# Regioselective Synthesis of Alcohols by Catalytic Transfer Hydrogenation of Epoxides

**DOI:** 10.1021/acs.joc.5c01342

**Published:** 2025-09-17

**Authors:** Sertaç Genç, Süleyman Gülcemal, Salih Günnaz, Bekir Çetinkaya, Jianliang Xiao, Derya Gülcemal

**Affiliations:** † Department of Chemistry, 37509 Ege University, Bornova, Izmir 35100, Turkiye; ‡ Department of Chemistry, 4591 University of Liverpool, Liverpool L69 7ZD, U.K.

## Abstract

Reductive ring opening of readily available epoxides provides easy access to synthetically important alcohols. However, controlling the regioselectivity of the reaction under user-friendly conditions remains challenging. Here, we present an efficient methodology for regioselective transfer hydrogenation (TH) of epoxides to synthesize Markovnikov and anti-Markovnikov alcohols using 2-propanol as the hydrogen source. An [IrCl­(cod)­(NHC)] (cod = 1,5-cyclooctadiene, NHC = N-heterocyclic carbene) complex as a precatalyst enables the formation of secondary and tertiary alcohols in good to excellent yields via selective TH of mono- and 2,2-disubstituted terminal epoxides. Remarkably, we found that an NHC–Ir & Pd/C cooperative catalysis approach can steer the regioselectivity of the reaction to afford anti-Markovnikov alcohols. This cooperative catalysis approach enables the transfer hydrogenative ring opening of mono- and 2,2-disubstituted terminal aryl epoxides to form linear alcohols and challenging internal epoxides to give branched alcohols.

## Graphical abstract

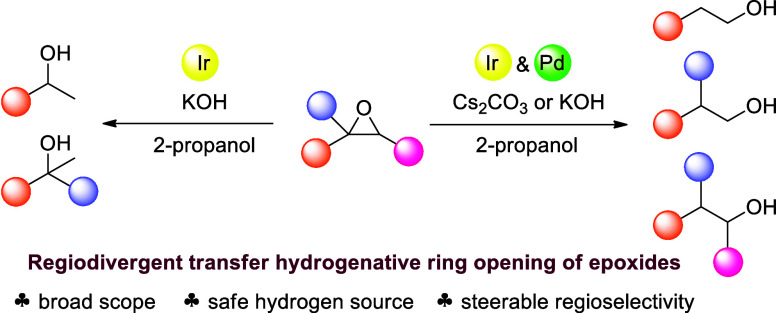

## 1 Introduction

Alcohols are versatile building blocks widely found in fine and bulk chemicals, pharmaceuticals, agrochemicals, and fragrances.^1^ As stoichiometric or catalytic functionalization of alcohols represents an attractive synthetic tool for constructing more complex molecules, the development of efficient protocols for the synthesis of alcohols is highly desirable.^2^ Among the numerous established procedures for the synthesis of alcohols, selective catalytic ring opening of epoxides, which are readily available from olefins, offers a highly attractive alternative.^3,4^ Due to the inherent high ring strain and strong polarization of the C–O bond, a key challenge for the reductive ring opening of nonsymmetrical epoxides is the control of regioselectivity to Markovnikov selective secondary alcohols or anti-Markovnikov selective primary alcohols (Scheme 1a).^3,4^ The steric nature of the substrates and the acidity or basicity of the catalytic systems play a crucial role in controlling the regioselectivity of these reactions.^4,5^ To this end, various catalytic systems have been developed in recent years for the selective synthesis of alcohols from epoxides (Scheme 1b). Among them, heterogeneous or homogeneous transition metal-catalyzed hydrogenation is one of the most employed methods for this transformation. Heterogeneous Pd-based catalysts were extensively studied for this reaction, where anti-Markovnikov alcohols are limited to aryl epoxides, while secondary alcohols are the major product in the case of alkyl epoxides.^6–13^ On the other hand, Ni-based heterogeneous catalysts selectively yield primary alcohols from both aryl and alkyl terminal epoxides.^14–16^ As demonstrated in previous studies, the regioselectivity of epoxide hydrogenation in the presence of homogeneous catalysts is substrate-controlled and is also strongly influenced by the acidity or basicity of the cocatalyst used in the reaction. Indeed, homogeneous ruthenium catalysts in combination with a strong base promote the selective formation of secondary alcohols from the hydrogenation of aryl and alkyl epoxides.^17–20^ Since 2019, significant progress has been made in the regioselective anti-Markovnikov hydrogenation of epoxides, where Lewis or Brønsted acids are used as cocatalysts together with homogeneous transition metal catalysts.^21–25^ In this regard, Ir/HOTf,^21^ Ti/Cr,^22^ Fe/TFA,^23^ Co/Zn­(OTf)_2_,^24^ and Fe/Al­(OTf)_3_^25^ catalyst systems selectively afforded anti-Markovnikov alcohols. The latter regiodivergent system is particularly interesting, because a simple change of the Lewis acid cocatalyst allows steering the regiocontrol toward either the primary or the secondary alcohols, but it suffers from a narrow substrate scope.^25^

**Scheme 1:**
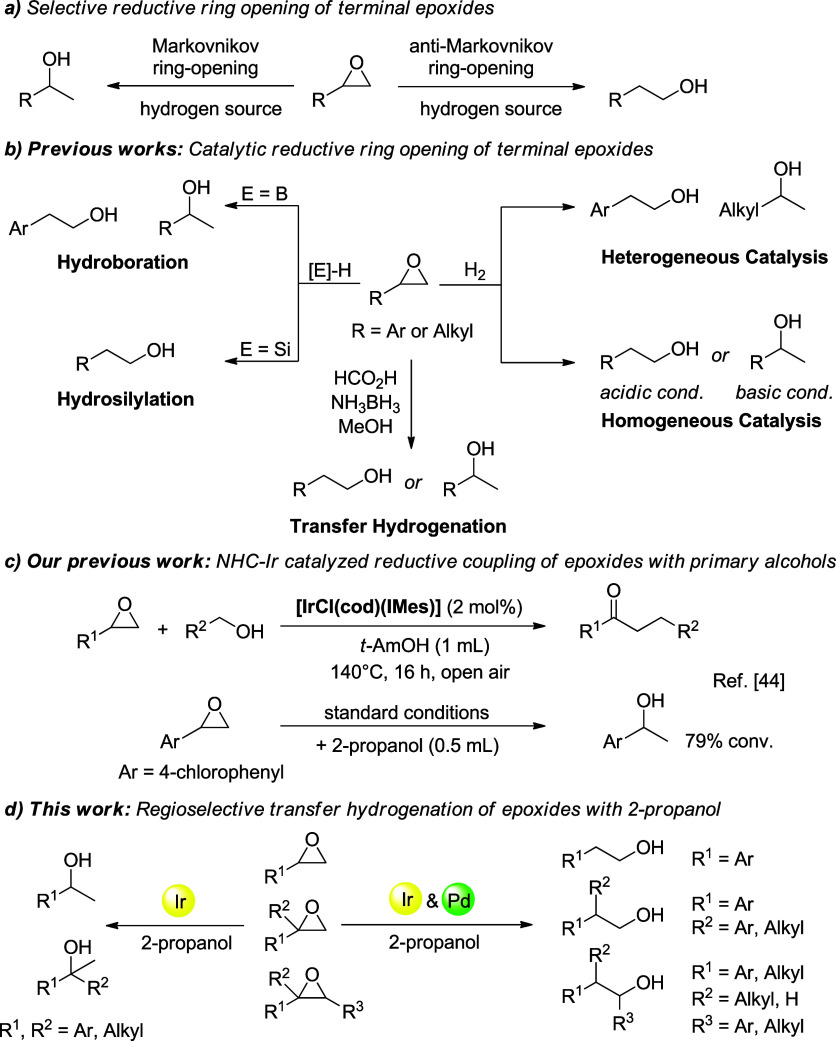
Catalytic Methods for Reductive Ring Opening of Epoxides

Due to the limitations associated with H_2_, such as flammability and high-pressure hazard, alternative approaches have also been developed for the ring opening of epoxides. Recently, catalytic hydroboration^26–31^ and hydrosilylation^32–34^ of epoxides have appeared as an alternative to the hydrogenation protocol. In 2020, Rueping and co-workers reported the MgBu_2_ catalyzed regiodivergent hydroboration of terminal epoxides to afford secondary alcohols. Here, replacing the MgBu_2_ catalyst with Mg­(NTf_2_)_2_ provided the primary alcohols when 2,2-disubstituted terminal aryl epoxides were tested.^26^ Although this study represents a remarkable advancement in tuning the regioselectivity of the reaction, this regiodivergence is limited only to 2,2-disubstituted terminal epoxides. Using other main-group metals,^27–29^ transition metals,^30^ and metal-free^31^ conditions in the hydroboration of epoxides also resulted in the formation of secondary alcohols. On the contrary, anti-Markovnikov selective primary alcohols were obtained by hydrosilylation of terminal epoxides.^32–34^ Although these hydroelementation processes employ milder reaction conditions, the complex workup procedures and the formation of considerable reaction waste because of the requirement of stoichiometric reagents limit their practicality in synthetic applications.

The limitations of direct hydrogenation and hydroelementation make it necessary to find safer and greener hydrogen donors. As an attractive alternative, TH using non-H_2_ hydrogen sources has become a rapidly growing method in synthetic chemistry.^35,36^ Zhou and co-workers reported the pioneering Pd-nanoparticle-catalyzed anti-Markovnikov reductive ring opening of terminal aryl epoxides using HCO_2_H/NEt_3_, in which a narrow substrate scope was explored.^37^ Very recently, Tian and co-workers developed an efficient catalytic system based on Fe­(BF_4_)_2_/tetraphos for the synthesis of primary alcohols from monosubstituted terminal aryl and alkyl epoxides using formic acid as the hydrogen source.^38^ The use of ammonia borane as the hydrogen source in the presence of PNP-Co/Er­(OTf)_3_ catalyst system selectively afforded anti-Markovnikov alcohols from both terminal aryl and alkyl epoxides.^39^ NHC–Mn/Zn­(OTf)_2_ catalyzed TH of terminal epoxides using ammonia borane also favored the formation of primary alcohols, but was limited to aryl epoxides, whereas Markovnikov-type alcohols were formed in the case of alkyl epoxides.^40^ Similar selectivity was achieved in the electroreduction of epoxides by using urea derivatives as hydrogen source.^41^ In contrast, photocatalytic reduction of terminal epoxides using alkene^42^ or methanol^5^ as hydrogen sources resulted in the selective formation of secondary alcohols. Among the various sacrificial hydrogen donors, 2-propanol is widely used in TH processes and has significant advantages, such as its low price, low toxicity, good production scale, easy recycling possibility and good solubilization capacity.^35,36^ However, as for the reduction of epoxides, there is only one single report using 2-propanol as the hydrogen source, and this photocatalytic Pt/TiO_2_ system suffers from a very limited scope.^43^

Product selectivity is crucial for the success of a chemical reaction. Therefore, regiodivergent ring opening of epoxides is of great importance as it determines the formation of specific alcohol products from a given epoxide substrate. Considering the current limitations associated with the catalytic regiodivergent ring opening of epoxides, we aimed to explore a general, safe, and user-friendly method for accessing either branched or linear alcohols. In 2021, we developed a new method for converting terminal epoxides and primary alcohols into α-alkylated ketones, which involves a one-pot Markovnikov TH of epoxide to a secondary alcohol and alkylation in the presence of an NHC–Ir (**Ir1**) catalyst.^44^ Subsequently, this method was adopted by other research groups, leading to various studies utilizing our approach.^45–47^ Here, control experiments proved that the corresponding secondary alcohol was obtained as the sole product from the ring opening of 4-chlorostyrene oxide in the presence of 2-propanol as the hydrogen source (Scheme 1c). In line with our interest in the development of reductive ring opening of epoxides, here we report an efficient NHC–Ir and NHC–Ir & Pd/C based catalytic system for the TH of epoxides that allows for tuning the product selectivity in terms of branched or linear alcohols with broad substrate scope (Scheme 1d). In addition, this user-friendly system uses safe and inexpensive 2-propanol as the reducing agent and does not require an inert atmosphere.

## 2 Results and Discussion

We began our study by screening the catalytic activities of several well-defined NHC–Ir complexes in the TH of 4-chlorostyrene oxide. The reaction was performed in the presence of an NHC–Ir complex (0.5 mol %) and KO^*t*^Bu (10 mol %) in 2-propanol (1 mL) at 82 °C (oil bath temperature) for 16 h under open air conditions. Besides commercially available **Ir1**, we tested a series of NHC–Ir complexes (**Ir2**–**Ir6**) prepared by our group (Table 1, entries 1–6).^48,49^ The electron-deficient complex **Ir6** showed the highest conversion and the selectivity to Markovnikov alcohol **4d** (entry 6). Besides KO^*t*^Bu, we also evaluated different bases (entries 7–10), and an improved conversion was achieved with KOH (entry 9). Increasing the reaction temperature to 95 °C resulted in the quantitative conversion of starting material and provided increased selectivity for the secondary alcohol (entry 9 vs entry 11). Furthermore, decreasing the amount of catalyst to 0.25 mol % resulted in a lower conversion (entry 12). Finally, control experiments demonstrated that both the base and the catalyst are essential to the reaction (entries 13 and 14). It should be noted that performing the reaction without the catalyst afforded a considerable amount of β-alkoxy ether byproduct (**4′d**) due to the activation of the epoxide with the base and nucleophilic attack of the 2-propanol (entry 14). Pleasingly, no oligomerization products were detected in the reaction mixture during the optimization studies.

**Table 1:**
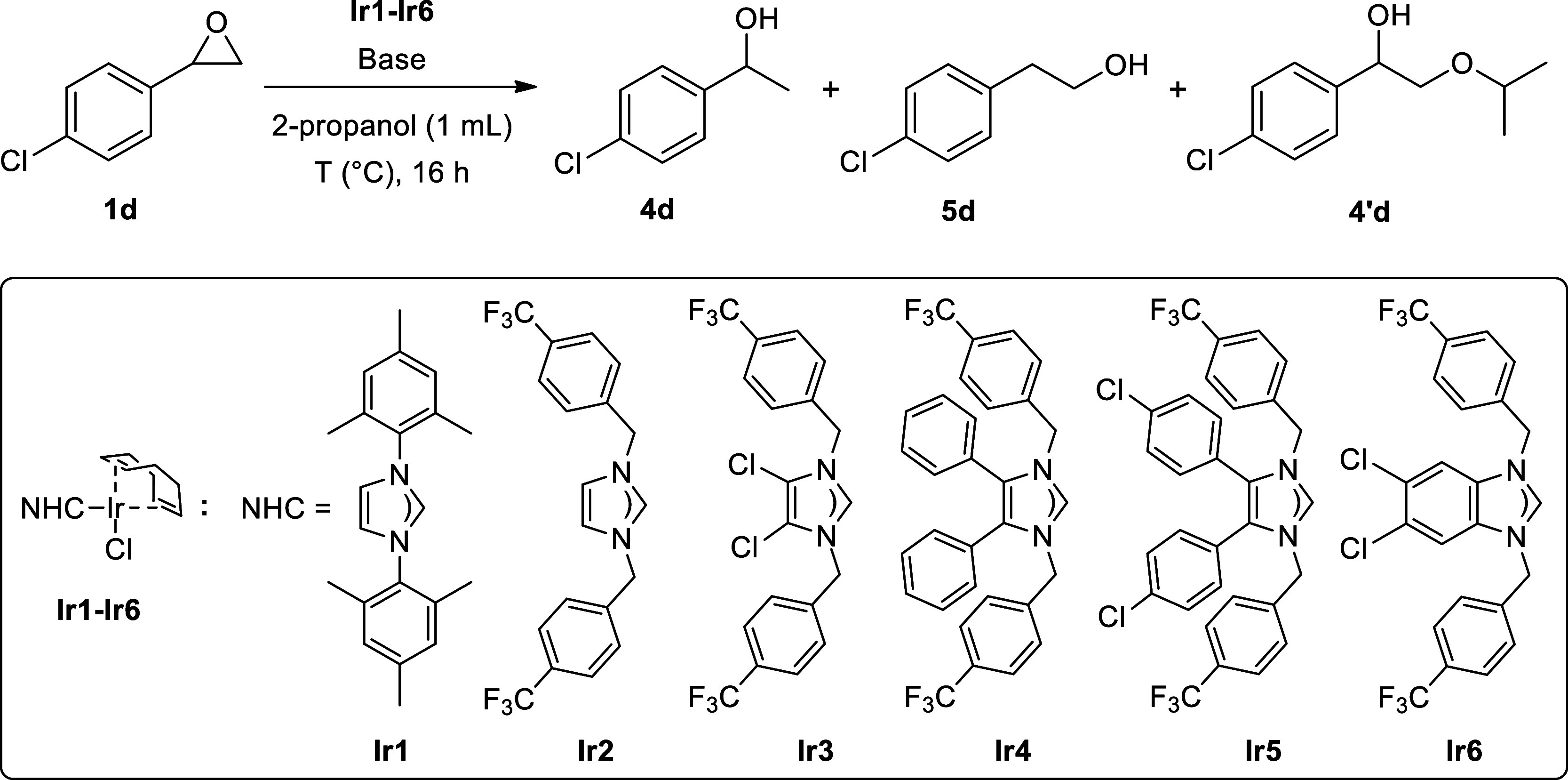
Optimization of the Reaction Conditions for Markovnikov Selective TH of Terminal Epoxides^a^

entry	cat. (mol %)	base (mol %)	*T* (°C)	conv. (%)^b^	**4d: 5d: 4′d** ratio^b^
1	**Ir1** (0.5)	KO^t^Bu (10)	82	32	31:0:69
2	**Ir2** (0.5)	KO^t^Bu (10)	82	28	42:4:54
3	**Ir3** (0.5)	KO^t^Bu (10)	82	49	78:6:16
4	**Ir4** (0.5)	KO^t^Bu (10)	82	46	71:7:22
5	**Ir5** (0.5)	KO^t^Bu (10)	82	54	81:4:15
6	**Ir6** (0.5)	KO^t^Bu (10)	82	66	86:8:6
7	**Ir6** (0.5)	NaO^t^Bu (10)	82	60	82:7:11
8	**Ir6** (0.5)	NaOH (10)	82	78	82:6:12
9	**Ir6** (0.5)	KOH (10)	82	84	89:5:6
10	**Ir6** (0.5)	Cs_2_CO_3_ (10)	82	26	81:8:11
11	**Ir6** (0.5)	KOH (10)	95	>99	97:3:0
12	**Ir6** (0.25)	KOH (10)	95	73	94:4:0
13	**Ir6** (0.5)		95	0	n.d.
14		KOH (10)	95	60	0:0:100

a Reaction conditions: **1d** (0.5 mmol), catalyst (0.1–0.5 mol %), base (5–10 mol %), 2-propanol (1 mL), T (82 or 95 °C, oil bath temperature), open to air.

b Determined from ^1^H NMR analysis of the crude reaction mixture using 1,3,5-trimethoxybenzene as the internal standard.

With the optimized conditions in hand, we explored the scope and limitations of the NHC–Ir catalyzed TH of different monosubstituted and 2,2-disubstituted terminal epoxides (**1** and **2**) to yield the corresponding Markovnikov alcohols (**4** and **6**) (Scheme 2). Monosubstituted styrene oxides were regioselectivitively converted to the corresponding secondary alcohols in high isolated yields (up to 95%), regardless of the electronic nature of the substituents at *para*-position of the phenyl ring in general. However, the highly electron-withdrawing nitro substituent on the phenyl ring led to the formation of the anti-Markovnikov alcohol 2-(4-nitrophenyl)­ethanol as the major product in 46% yield, probably due to the α position of the epoxide being made more electrophilic by the nitro moiety (vide infra).^50^ In addition, TH of styrene oxide enabled gram-scale synthesis of 1-phenylethanol (**4a**) with 87% yield. In a limited number of cases (**4j** and **4k**), higher catalyst and base loadings and temperatures were required to achieve moderate yields. However, when the sterically bulky 2-mesityloxirane (**1m**) was subjected to TH under similar conditions, no product formation was observed.

**Scheme 2:**
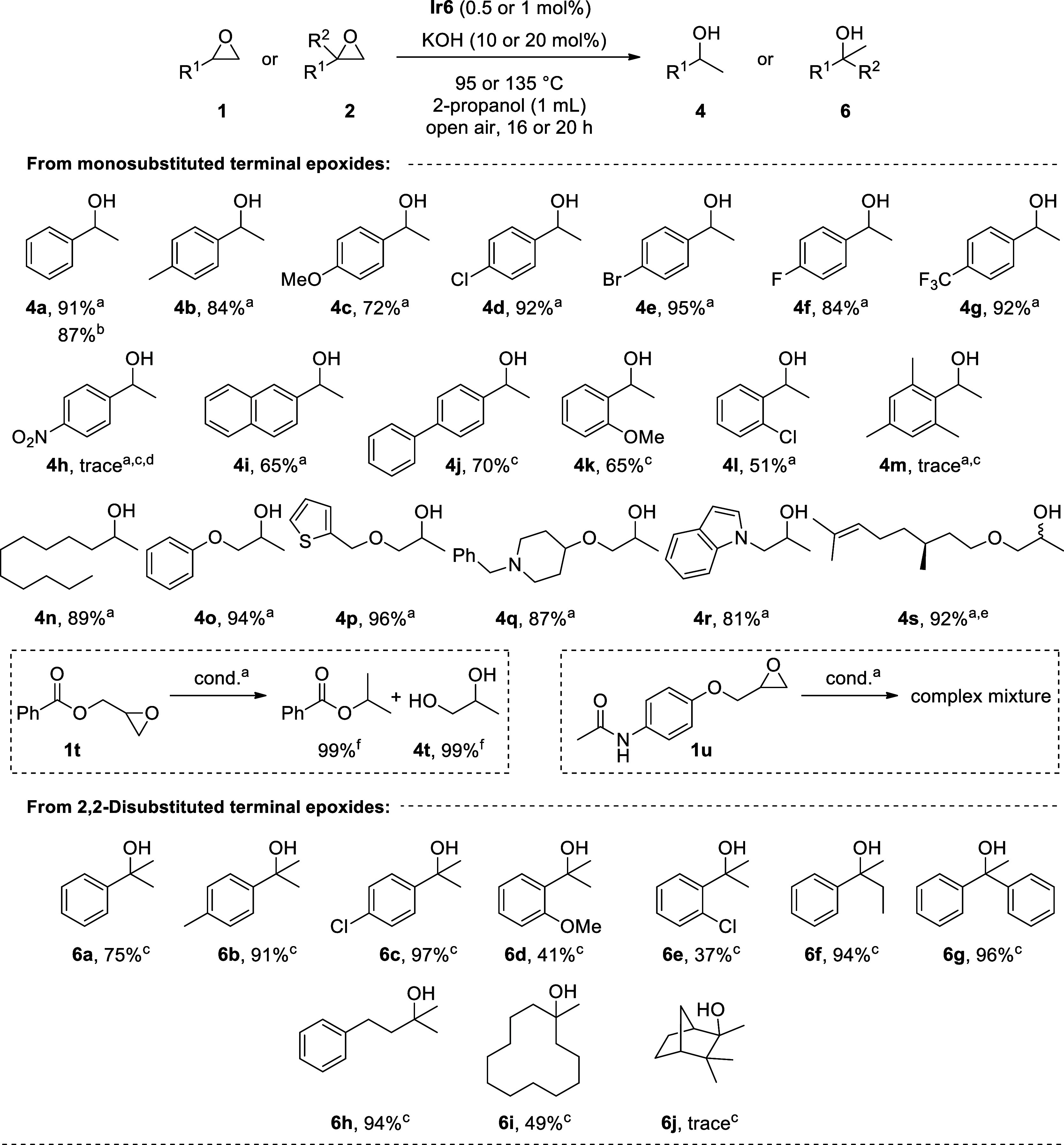
Scope of the NHC–Ir Catalyzed TH of Epoxides

a
**1** (0.5 mmol), **Ir6** (0.5 mol %), KOH (10 mol %), 2-propanol (1 mL), 95 °C (oil bath temperature), 16 h, open to air. Isolated yields.
b The reaction was carried out with 10 mmol of styrene oxide.
c
**1** or **2** (0.5 mmol), **Ir6** (1 mol %), KOH (20 mol %), 2-propanol (1 mL), 135 °C (oil bath temperature), 20 h, open to air. Isolated yields.
d The anti-Markovnikov selective 2-(4-nitrophenyl)­ethanol was obtained in 46% yield.
e Mixture of diastereoisomers (61:39).
f Yield determined by ^1^H NMR using 1,3,5-trimethoxybenzene as the internal standard.

We then turned our attention to alkyl-substituted epoxides. As shown in Scheme 2, secondary alcohols (**4n–4s**) were successfully obtained in high yields. Here, various functional groups are well-tolerated; epoxides bearing different functionalities such as ether, thiophene, piperidine, indole, and alkene afforded the respective secondary alcohols in excellent yields. On the other hand, one limitation of our method concerns the reactivity of ester or amide functionality. Thus, TH of oxiran-2-ylmethyl benzoate (**1t**) under standard conditions resulted in the formation of isopropyl benzoate and propane-1,2-diol (**4t**). This might be explained by the transesterification of **1t** with 2-propanol under basic conditions and subsequent TH of resulting oxiran-2-ylmethanol to give **4t**.^5^ TH of N-(4-(oxiran-2-ylmethoxy)­phenyl)­acetamide (**1u**) provided a complex mixture.

Encouraged by these results, the scope of the reaction was also extended to the more challenging 2,2-disubstituted terminal epoxides (**2a–2j**) to yield tertiary alcohols (**6a–6j**). Although they are synthetically important building blocks found in a variety of biologically active compounds,^51–53^ only one method is available for the catalytic reduction of epoxides to give tertiary alcohols.^26^ Pleasingly, epoxides with different steric and electronic properties on the aryl group and with different alkyl substituents, including macrocycles, were selectively converted to tertiary alcohols (**6a–6i**) in moderate to excellent yields. However, the sterically demanding norbornane epoxide afforded only a trace amount of **6j**.

Given the fact that hydrogenation of terminal aryl epoxides with heterogeneous Pd catalysts produces the anti-Markovnikov alcohols,^7–13,54^ we decided to explore whether introducing a commercially available Pd/C catalyst to our NHC–Ir catalytic system would allow for the selective C–O bond activation to give linear alcohols. We hypothesized that the activation of the epoxide by the palladium cocatalyst could result in anti-Markovnikov selective ring opening, leading to aldehydes via isomerization,^55–57^ which would then be reduced by NHC–Ir catalyzed TH to furnish linear alcohols. To our surprise somehow, the addition of Pd/C (2 mol % Pd) to the TH of epoxide **1a** indeed allowed preferential formation of the linear alcohol product (**4a:5a** ratio = 23:77, Table 2, entry 1). This result implies predominant formation of primary alcohol in the presence of Pd/C. To suppress branched alcohol formation, KOH was replaced with Cs_2_CO_3_ (Cs_2_CO_3_ provided only 21% branched alcohol in the NHC–Ir catalyzed TH reaction, see Table 1, entry 10). Delightfully, the reaction resulted in the 97% conversion of styrene oxide with a complete selectivity for the desired linear alcohol **5a** (Table 2, entry 2). Other weak bases showed full regioselectivity toward the linear alcohol, but with lower conversions (Table 2, entries 3–5). Lowering the amount of Pd/C or NHC–Ir led to a decrease in the conversion (Table 2, entries 6 and 7). Additionally, the TH of **1a** without an NHC–Ir catalyst produced only 15% linear alcohol (Table 2, entry 8), most likely via the in situ generated Pd–H intermediate in the presence of 2-propanol and the base.^58^ Performing the reaction without NHC–Ir and Pd/C catalysts gave 47% of β-alkoxy ether byproduct (**4′a)** due to the presence of the in situ generated isopropoxide nucleophile (Table 2, entry 9), as observed above (Table 1, entry 14). The reaction of **1a** under base-free conditions yielded only 13% of β-hydroxy ether product (**5′a),** and the unreacted epoxide was recovered after the reaction (Table 2, entry 10). This result shows that the isopropoxide nucleophile can be formed even under base-free conditions. Expanding the investigation, different heterogeneous or homogeneous Pd sources (Pd­(OH)_2_/C, Pd_2_(dba)_3_, Pd­(OAc)_2_) were also screened; however, these did not lead to better outcomes (Table 2, entries 11–13). Finally, Fe­(BF_4_)_2_, ZnCl_2_, CuCl_2_, and NiCl_2_(PPh_3_)_2_, were employed as first-row transition metal-based LAs (2 mol %), but they led to less than 10% conversion of epoxide **1a**.

**Table 2:**
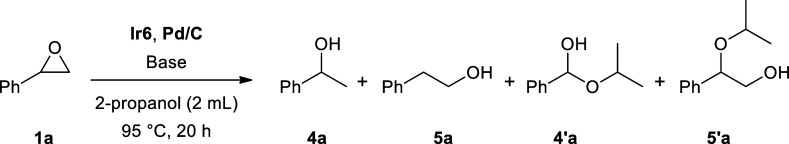
Optimization of the Reaction Conditions for anti-Markovnikov Selective TH of Terminal Epoxides^a^

entry	**Ir6** (mol %)	**Pd** (mol % Pd)	base (mol %)	conv. (%)^b^	**4a: 5a: 4′a: 5′a** ratio^b^
1	0.5	Pd/C (2)	KOH (10)	79	23:77:0:0
2	0.5	Pd/C (2)	Cs_2_CO_3_ (10)	97	0:100:0:0
3	0.5	Pd/C (2)	K_3_PO_4_ (10)	56	0:100:0:0
4	0.5	Pd/C (2)	K_2_CO_3_ (10)	32	0:100:0:0
5	0.5	Pd/C (2)	Na_2_CO_3_ (10)	22	0:100:0:0
6	0.5	Pd/C (0.4)	Cs_2_CO_3_ (10)	36	0:100:0:0
7	0.1	Pd/C (2)	Cs_2_CO_3_ (10)	54	0:100:0:0
8		Pd/C (2)	Cs_2_CO_3_ (10)	15	0:100:0:0
9			Cs_2_CO_3_ (10)	47	0:0:100:0
10	0.5	Pd/C (2)		13	0:0:0:100
11	0.5	Pd(OH)_2_/C (2)	Cs_2_CO_3_ (10)	76	0:100:0:0
12	0.5	**Pd**_**2**_ **(dba)**_**3**_ (2)	Cs_2_CO_3_ (10)	24	0:100:0:0
13	0.5	**Pd(OAc)**_**2**_ (2)	Cs_2_CO_3_ (10)	3	n.d.

a Reaction conditions: **1a** (0.5 mmol), **Ir6** (0.1–0.5 mol %), **Pd/C** (0.4–2 mol %), base (10 mol %), 2-propanol (2 mL), 95 °C (oil bath temperature), open to air.

b Determined from^1^H NMR analysis of the crude reaction mixture using 1,3,5-trimethoxybenzene as the internal standard.

Following this optimization, we probed the substrate scope of the NHC–Ir & Pd/C cocatalyzed TH of epoxides (Scheme 3). Terminal aryl epoxides with different electronic nature in the p*ara*-position afforded the corresponding primary alcohols (**5a–5g**) in good yields and high regioselectivities. Of note, the reaction of 4-chlorostyrene oxide (**1d**) to **5d** was accompanied by reductive dechlorination via Pd-catalyzed cleavage of the C–Cl bond, affording 2-phenylethanol (**5a**) as the byproduct in 10% yield.^59^ In addition, 2-(naphthalen-2-yl)­oxirane and epoxides with OCH_3_, Cl, or CH_3_ groups at the *ortho*-position(s) of the phenyl ring provided the respective primary alcohols (**5h–5k**) in high yields. Note that unlike **4m**, **5k** was obtained in a good yield, likely due to the presence of the palladium catalyst that alters the steric effect imposed on the attacking hydride in the case of the latter. Although this regioselectivity switch is very promising, it is limited to aromatic epoxides and shows a strong substrate dependence on regioselectivity. Thus, the regioselectivity was completely switched to Markovnikov alcohols when nonaryl-substituted terminal epoxides were used (**4n–4u**). This is in consistence with the requirement of a Lewis or Brønsted acid cocatalyst for the activation of aliphatic epoxide to give anti-Markovnikov selective alcohols.^21–25,33,34,38,39^ It should be noted that under the applied conditions the use of Cs_2_CO_3_ provided a considerable amount of β-alkoxy ether byproduct (**4′**), whereas Na_2_CO_3_ produced **4n** and **4o** in good yields. Pleasingly, the amide functionality of the paracetamol-derived epoxide substrate was unaffected under these weakly basic conditions, leading to the corresponding secondary alcohol **4u** in 94% yield.

**Scheme 3:**
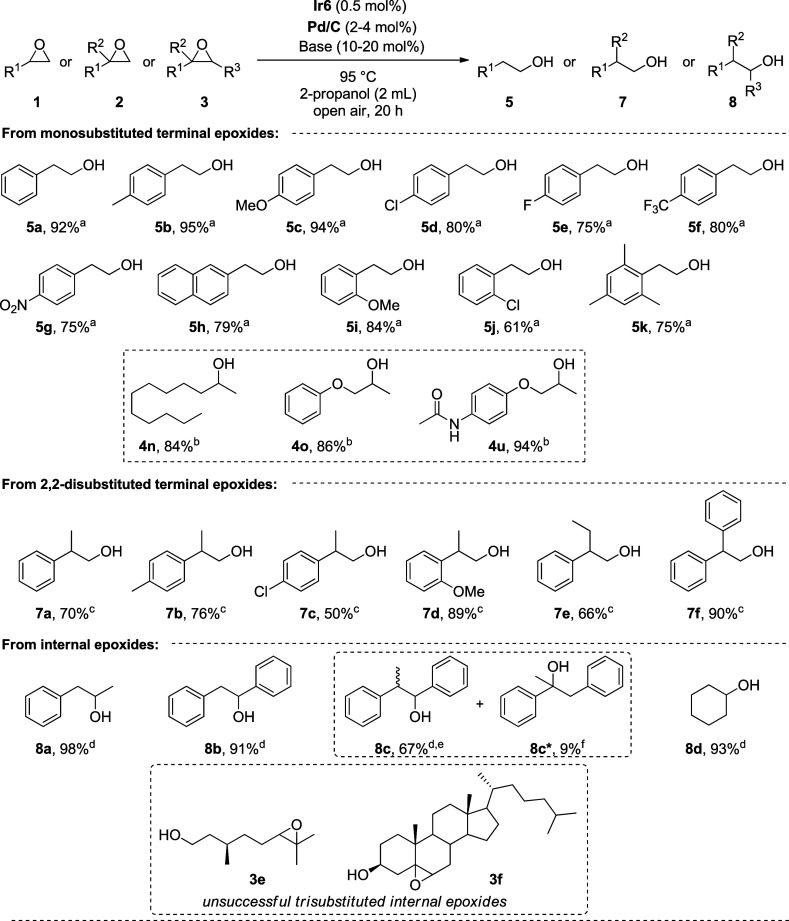
Scope of the NHC–Ir & Pd/C Catalyzed TH of Epoxides

a
**1** (0.5 mmol), **Ir6** (0.5 mol %), **Pd/C** (2 mol % of Pd), Cs_2_CO_3_ (10 mol %), 2-propanol (2 mL), 95 °C (oil bath temperature), 20 h, open to air. Isolated yields.
b Na_2_CO_3_ (10 mol %) was used instead of Cs_2_CO_3_.
c
**2** (0.5 mmol), **Ir6** (0.5 mol %), **Pd/C** (3 mol % of Pd), Cs_2_CO_3_ (10 mol %), 2-propanol (2 mL), 95 °C (oil bath temperature), 20 h, open to air. Isolated yields.
d
**3** (0.5 mmol), **Ir6** (0.5 mol %), **Pd/C** (4 mol % of Pd), KOH (20 mol %), 2-propanol (2 mL), 95 °C (oil bath temperature), 20 h, open to air. Isolated yields.
e Mixture of diastereoisomers (77:23).
f The side product 8c* was observed by the standard ^1^H and ^13^C NMR peaks of isolated product mixture.

To further expand the scope of the protocol, we turned our attention to 2,2-disubstituted terminal epoxides. Aromatic epoxides with different electronic and steric properties provided the corresponding primary alcohols (**7a–7f**) in good to excellent yields. Remarkably, with an increased Pd/C load (4 mol %) and 20 mol % KOH, di- and trisubstituted internal epoxides were successfully applied and yielded the desired secondary alcohols (**8a–8d**) in good yields. It should be noted that the reaction of the internal epoxide **3c** resulted in a mixture of a desired secondary alcohol **8c** (67%) the tertiary alcohol byproduct 8c* (9%), which were inseparable by column chromatography due to their similar polarities. However, applications of aliphatic trisubstituted epoxides derived from citronellol (**3e**) and cholesterol (**3f**) were not successful in conversion to the desired product, presumably due to steric and/or electronic effects that render the epoxides less active.

To better understand the regiodivergent character of the TH reaction, a set of mechanistic experiments was conducted for both catalytic systems. Our preliminary findings demonstrated that the transfer hydrogenative ring opening of epoxides proceeds mainly via an NHC–Ir catalyzed reduction pathway rather than the Meinwald rearrangement.^44^ We began our investigations to get a better insight into the possible involvement of the Meinwald rearrangement in the NHC–Ir catalyzed TH of terminal epoxides. First, kinetic studies using 4-chlorostyrene oxide (**1d**) under the optimized conditions (Table 1, entry 11) were performed. As seen in Figure 1, no formation of any carbonyl intermediate was observed during the entire course of the reaction, indicating that the isomerization process is less likely to be involved. Next, we carried out an isomerization experiment in the absence of 2-propanol. The reaction of **1a** in acetonitrile or *tert*-amyl alcohol did not produce any isomerization products, again ruling out the involvement of the Meinwald rearrangement (Scheme 4a).^18^ When an enantiopure (*R*)-styrene oxide was tested under the optimized conditions, racemic **4a** was obtained in 93% yield (Scheme 4b), indicating that the product racemization step should also be considered in the reaction mechanism.^19^ We then confirmed the dehydrogenation of **4a** in the absence of 2-propanol, where the reaction proceeded to afford acetophenone (**9**) in 20% yield after 16 h (Scheme 4c). The low yield could result from the reaction being reversible. In contrast, the TH of acetophenone under the standard reaction conditions resulted in the quantitative formation of **4a** in only 30 min (Scheme 4d). Finally, 1-deuterated analogue of **4a** (**4a–D**) was fully converted into **9** (4%) and **4a** (93%) under the standard conditions (Scheme 4e). This result suggests that alcohol dehydrogenation and ketone reduction occur through a reversible hydrogen transfer process and is consistent with the findings obtained in the racemization experiment.

**Figure 1:**
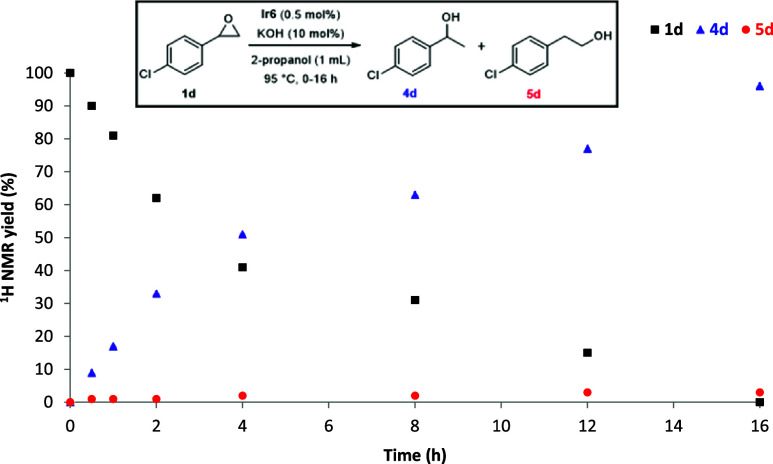
Time course of the NHC–Ir catalyzed TH of **1d**.

**Scheme 4:**
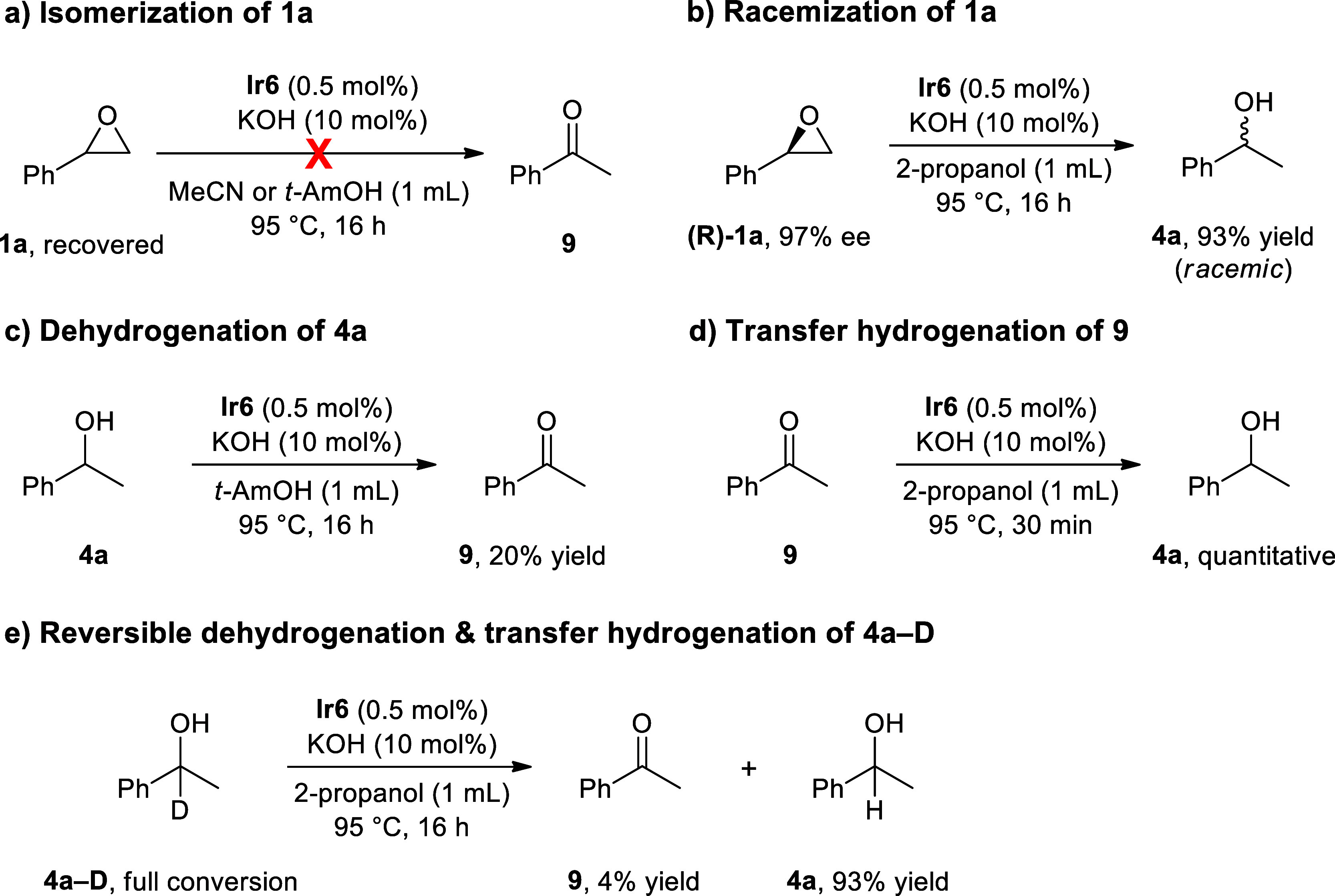
Control Experiments on the NHC–Ir Catalyzed TH of Epoxides

Next, we focused on performing control experiments on the NHC–Ir & Pd/C catalyzed TH of epoxides. Similarly, under the standard reaction conditions, no isomerization was observed in acetonitrile or *tert*-amyl alcohol (devoid of hydrogen source) (Scheme 5a). Performing the same reactions in the presence of 10 equiv of 2-propanol resulted in the formation of the linear alcohol product (**5a**) in 37% and 46% yields, respectively, and unreacted epoxide was recovered after the reactions. Furthermore, the TH of 2-phenylacetaldehyde under the standard reaction conditions resulted in only a trace amount of 2-phenylethanol, indicating that the primary alcohol was not produced via the TH of in situ generated aldehyde and ruling out the involvement of the isomerization process (Scheme 5b).^13^ However, a small amount of isomerization product (3–10%) was observed when **1a** was used as the substrate under base-free conditions in the presence of Pd/C catalyst (Scheme 5c). Remarkably, no isomerization was observed in the absence of Pd/C under these base-free conditions (Scheme 5c), indicating the necessity of Pd for C–O bond activation.

**Scheme 5:**
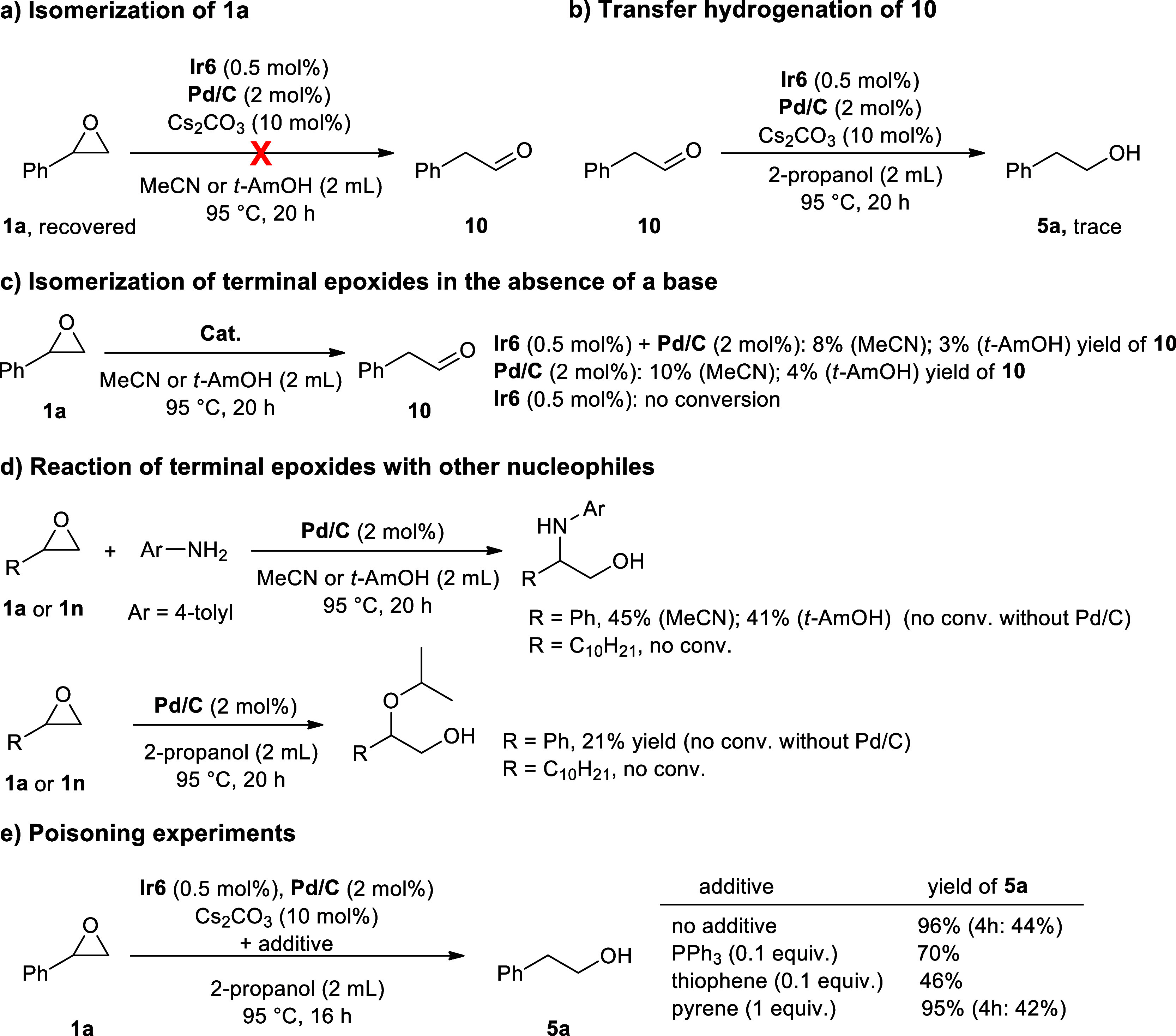
Control Experiments on the NHC–Ir & Pd/C Catalyzed TH of Epoxides

The difference in regioselectivity toward linear or branched alcohol in the reductive ring opening of aromatic and aliphatic epoxides is mainly attributed to the different interaction modes of these epoxides with the Pd surface.^12,13^ Adsorption of aromatic epoxides onto the electron deficient catalyst surface is possible through the π-electrons of the aromatic ring and the O atom of the epoxide, and the regioselectivity toward primary alcohols could be explained by the π-complexation which makes the α position of the epoxide more electron deficient and hence more susceptible to attack by nucleophiles, such as a hydride.^12,13,60,61^ This is somewhat reminiscent of the formation of the anti-Markovnikov alcohol in the case of 2-(4-nitrophenyl)­ethanol without Pd/C (Scheme 2). To verify the C–O bond cleavage being influenced by interactions with palladium, we performed reactions involving different nucleophiles with aromatic and aliphatic epoxides (Scheme 5d). The reaction of the aromatic epoxide (**1a**) with *p*-toluidine and 2-propanol proceeded in the presence of 2 mol % of Pd/C under base-free conditions and resulted in the formation of the corresponding β-amino alcohol (41–45%) and β-hydroxy ether (21%) products, respectively. Here, unreacted amine and epoxide were recovered after the reaction. Notably, no reactions were observed without Pd/C, indicating the role of palladium for epoxide activation which presumably results in weakening of the benzylic C–O bond of the epoxide. Addition of the nucleophile onto the electron deficient α-carbon should favor the formation of linear alcohols from aromatic epoxides. Meanwhile, the aliphatic epoxide (**1n**) remained unreacted under the same conditions. This may be expected, as the alkyl chain is much less likely to interact with the palladium surface.

The unique regioselectivity toward linear alcohol for aromatic epoxide hydrogenation has been attributed to interactions between the aromatic ring and the Pd surface, i.e. π-complexation. Thus, the selectivity toward linear alcohol is believed to increase with the number of active Pd sites.^12,60^ With this in mind, we investigated the impact of surface deactivation of Pd/C on the catalytic activity (Scheme 5e). The TH of styrene oxide in the presence of 0.1 equiv of PPh_3_ or thiophene led to significantly lower yields for the desired product **5a** (70% and 46%, respectively). These results support the notion that coordination of PPh_3_ or thiophene with Pd reduces the number of Pd atom ensembles necessary for interacting with the aromatic ring and thereby leads to the deactivation of Pd/C. Finally, we examined the potential poisoning effect of fused aromatic rings through their adsorption on the Pd/C surface via aromatic π–interactions. Anticipating that a fused aromatic ring would adsorb more strongly to the catalyst surface than styrene oxide and may inhibit catalytic activity, we carried out a reaction in the presence of 1.0 equiv pyrene (Scheme 5e). The addition of pyrene to the reaction under standard conditions did not cause any influence on catalytic activity and the regioselectivity, indicating that the activation of aromatic epoxides by Pd/C are not only promoted by noncovalent π–interactions of aryl groups on substrates, but may also involve the interaction of the oxygen atom of the epoxide with the palladium surface.^12,13,60^

We also performed a Hammett^62^ study to compare the electronic effects of styrene oxide substrates on the NHC–Ir or NHC–Ir & Pd/C catalyzed TH of epoxides. Thus, a Hammett plot of log­(*k*_X_/*k*_H_) against the substituent constant σ_p_ was constructed by using the initial rate for TH of a series of *para*-substituted (–CH_3_, –H, –Cl, –CF_3_) styrene oxides. Remarkably, as shown in Figure 2, an opposite trend was observed from the plots of log­(*k*_X_/*k*_H_) vs σ_p_ between these catalytic systems. A positive slope (0.88) was observed for the NHC–Ir catalyzed TH, indicating that the reaction accelerates when electron-withdrawing substituents in the *para* position of the phenyl ring are present and therefore, the transition state experiences negative charge buildup during the rate-determining ring opening step (Figure 2a).^63^ On the contrary, a negative slope (−0.45) appears to imply the buildup of a positive charge on the benzylic carbon of the epoxide in the rate-determination step of the NHC–Ir & Pd/C catalyzed TH reaction.^64^

**Figure 2:**
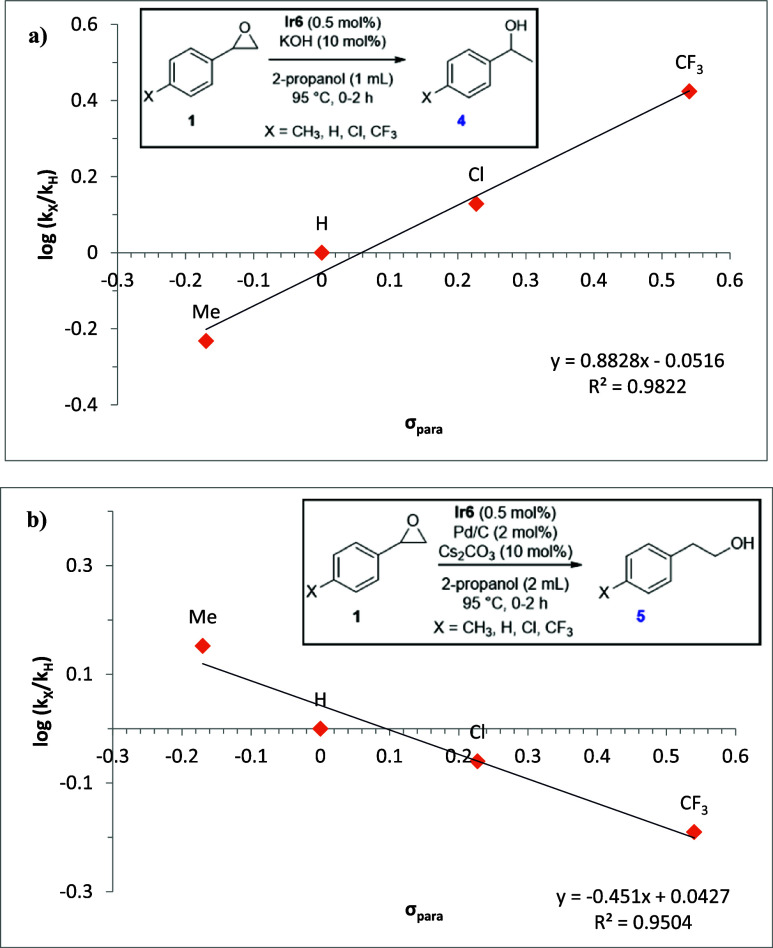
Hammett plots for (a) NHC–Ir and (b) NHC–Ir & Pd/C catalyzed TH of *para*-substituted styrene oxides obtained from noncompetitive experiments.

Based on experimental observations and previous reports,^12,13,20,48,60,61,65–67^ a plausible catalytic cycle for the transfer hydrogenative regiodivergent ring opening reaction is proposed (Scheme 6). The NHC–Ir catalyzed TH mechanism (Scheme 6a) starts with the generation of an iridium alkoxide species from complex **Ir6** and the 2-propanol in the presence of the base. Then, β-H elimination generates the transient iridium hydride.^48,65^ Next, an S_N2_-like attack of the iridium hydride on the less-hindered epoxide carbon gives the ion pair through a S_N2_-like transition state.^20,66^ This hydride transfer step is likely to be turnover limiting, which is in agreement with the Hammett studies (Figure 2a) that indicate that the α-carbon experiences negative charge accumulation. Finally, releasing the secondary alcohol from the ion pair regenerates the iridium alkoxide, completing the catalytic cycle. Meanwhile, it should be emphasized that while the formation of racemic alcohols from chiral epoxides (Scheme 4b) contradicts the expectation of a reaction proceeding through an S_N2_-like mechanism, the observed product racemization likely proceeds through reversible dehydrogenation and TH sequences (Scheme 4c–e).^67^

**Scheme 6:**
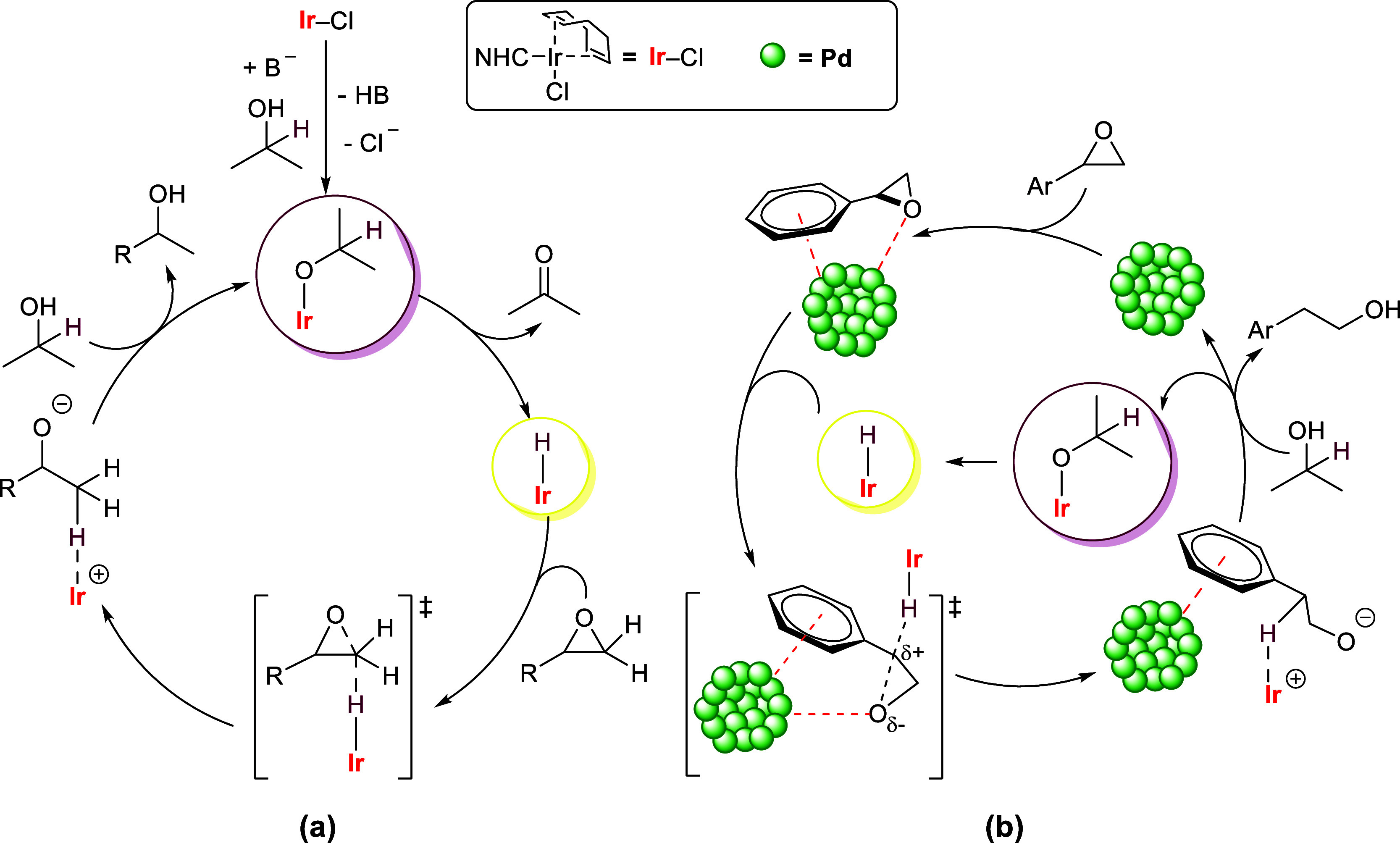
Proposed Mechanism for (a) NHC–Ir and (b) NHC–Ir & Pd/C Catalyzed TH of Epoxides

On the other hand, the complete regioselectivity switch in the NHC–Ir & Pd/C cocatalyzed TH reaction is likely driven by a different epoxide activation mode (Scheme 6b). We propose that the aryl group in aromatic epoxides interact with the electron-deficient surface atoms of palladium particles via its π-electrons, promoting the interaction of the epoxide moiety, including its oxygen atom, with and hence its activation by the palladium particles. This could make the benzylic position more electrophilic and lead to the weakening or ring opening of the C–O bond at this site due to the greater stability of the carbocation resulting from the resonance effect of the benzene ring.^12,13,60,61^ Subsequent reduction through an S_N1_ or S_N2_-like attack of the hydride at the electrophilic benzylic carbon gives rise to an anti-Markovnikov intermediate. The accumulation of positive charge in the transition state of the hydride attack is consistent with the results of Hammett experiments (Figure 2b). Finally, release of the primary alcohol regenerates the palladium species and iridium alkoxide, completing the catalytic cycle.

## 3 Conclusions

In summary, a new protocol has been developed for the regiodivergent transfer hydrogenative ring opening of a wide range of epoxides. The protocol uses safe, readily available and inexpensive 2-propanol as the hydrogen source and does not require an inert atmosphere. An NHC–Ir complex provides the corresponding secondary and tertiary alcohols in good to excellent yields via selective TH of mono- and 2,2-disubstituted terminal epoxides. Remarkably, introducing a commercially available Pd/C to the NHC–Ir system allows steering the selectivity of the reaction to anti-Markovnikov alcohols in the case of aromatic epoxides. This NHC–Ir & Pd/C based cooperative catalysis approach allows the formation of the primary alcohols from mono- and 2,2-disubstituted terminal aryl epoxides. Moreover, the latter system efficiently catalyzes the TH of challenging di- and trisubstituted internal epoxides toward branched alcohols. Initial mechanistic studies, including control experiments, kinetics, and Hammett studies, suggest that the observed regiodivergency is related with the different activation modes of epoxides. Whereas an S_N2_-like mechanism is proposed for the Markovnikov selective ring opening, introducing Pd/C facilitates the ring opening of aryl epoxides at the electrophilic benzylic carbon to furnish anti-Markovnikov products due to π-complexation of the epoxides.

## 4 Experimental Section

### 4.1 NHC–Ir Catalyzed TH of Monosubstituted Terminal Epoxides (**GP2a**)

Alcohols **4a–4s** were prepared according to the **GP2a**: To a 20 mL reaction tube with a condenser, epoxide (0.5 mmol), KOH (2.8 mg, 0.05 mmol; 10 mol %), **Ir6** (2.1 mg, 0.0025 mmol, 0.5 mol %) and 2-propanol (1.0 mL) were added under open air conditions. The reaction mixture was vigorously stirred under reflux in a preheated oil bath at 95 °C for 16 h. Thereafter, the reaction mixture was cooled to ambient temperature and the reaction mixture was diluted with 5 mL dichloromethane. After filtration, the solvent was evaporated, and the crude product was purified by column chromatography over silica gel.

### 4.2 NHC–Ir Catalyzed TH of 2,2-Disubstituted Terminal Epoxides (**GP2b**)

Alcohols **6a–6i** were prepared according to the **GP2b**: To a 20 mL reaction tube with a condenser, epoxide (0.5 mmol), KOH (5.6 mg, 0.1 mmol; 20 mol %), **Ir6** (4.2 mg, 0.005 mmol, 1 mol %) and 2-propanol (1.0 mL) were added under open air conditions. The reaction mixture was vigorously stirred under reflux in a preheated oil bath at 135 °C for 20 h. Thereafter, the reaction mixture was cooled to ambient temperature and the reaction mixture was diluted with 5 mL dichloromethane. After filtration, the solvent was evaporated, and the crude product was purified by column chromatography over silica gel.

### 4.3 NHC–Ir & Pd/C Catalyzed TH of Monosubstituted Terminal Aryl Epoxides (**GP2c**)

Alcohols **5a–5k** were prepared according to the **GP2c**: To a 20 mL reaction tube with a condenser, epoxide (0.5 mmol), Cs_2_CO_3_ (16.3 mg, 0.05 mmol; 10 mol %), **Ir6** (2.1 mg, 0.0025 mmol, 0.5 mol %), 10% **Pd/C** (10.6 mg, 0.01 mmol, 2 mol %) and 2-propanol (2.0 mL) were added under open air conditions. The reaction mixture was vigorously stirred under reflux in a preheated oil bath at 95 °C for 20 h. Thereafter, the reaction mixture was cooled to ambient temperature and the reaction mixture was diluted with 5 mL dichloromethane. After filtration, the solvent was evaporated, and the crude product was purified by column chromatography over silica gel.

### 4.4 NHC–Ir & Pd/C Catalyzed TH of Monosubstituted Terminal Alkyl Epoxides (**GP 2d)**

Alcohols **4n**, **4o** and **4u** were prepared according to the **GP 2d**: To a 20 mL reaction tube with a condenser, epoxide (0.5 mmol), Na_2_CO_3_ (5.3 mg, 0.05 mmol; 10 mol %), **Ir6** (2.1 mg, 0.0025 mmol, 0.5 mol %), 10% **Pd/C** (10.6 mg, 0.01 mmol, 2 mol %) and 2-propanol (2.0 mL) were added under open air conditions. The reaction mixture was vigorously stirred under reflux in a preheated oil bath at 95 °C for 20 h. Thereafter, the reaction mixture was cooled to ambient temperature and the reaction mixture was diluted with 5 mL dichloromethane. After filtration, the solvent was evaporated, and the crude product was purified by column chromatography over silica gel.

### 4.5 NHC–Ir & Pd/C Catalyzed TH of 2,2-Disubstituted Terminal Epoxides (**GP2e**)

Alcohols **7a–7f** were prepared according to the **GP2e**: To a 20 mL reaction tube with a condenser, epoxide (0.5 mmol), Cs_2_CO_3_ (16.3 mg, 0.05 mmol; 10 mol %), **Ir6** (2.1 mg, 0.0025 mmol, 0.5 mol %), **Pd/C** (15.9 mg, 0.015 mmol, 3 mol %) and 2-propanol (2.0 mL) were added under open air conditions. The reaction mixture was vigorously stirred under reflux in a preheated oil bath at 95 °C for 20 h. Thereafter, the reaction mixture was cooled to ambient temperature and the reaction mixture was diluted with 5 mL dichloromethane. After filtration, the solvent was evaporated, and the crude product was purified by column chromatography over silica gel.

### 4.6 NHC–Ir & Pd/C Catalyzed TH of Internal Epoxides (**GP 2f**)

Alcohols **8a–8d** were prepared according to the **GP 2f**: To a 20 mL reaction tube with a condenser, epoxide (0.5 mmol), KOH (5.6 mg, 0.1 mmol; 20 mol %), **Ir6** (2.1 mg, 0.0025 mmol, 0.5 mol %), **Pd/C** (21.3 mg, 0.02 mmol, 4 mol %) and 2-propanol (2.0 mL) were added under open air conditions. The reaction mixture was vigorously stirred under reflux in a preheated oil bath at 95 °C for 20 h. Thereafter, the reaction mixture was cooled to ambient temperature and the reaction mixture was diluted with 5 mL dichloromethane. After filtration, the solvent was evaporated, and the crude product was purified by column chromatography over silica gel.

## Supplementary Material



## Data Availability

The data underlying this study are available in the published article and its Supporting Information

## Notes

Detailed experimental section, characterization data and copies of NMR spectra (PDF)

## References

[1] Weissermel, K. ; Arpe, H.-J. Industrial Organic Chemistry, 4th ed.; Wiley–VCH: Hoboken, NJ, 2008.

[2] Cook A., Newman S. G. (2024). Alcohols as Substrates in Transition-Metal-Catalyzed
Arylation, Alkylation, and Related Reactions. Chem. Rev..

[3] Huang C.-Y., Doyle A. G. (2014). The Chemistry of Transition-Metals with Three-Membered
Ring Heterocycles. Chem. Rev..

[4] Thiyagarajan S., Gunanathan C. (2022). Catalytic Hydrogenation of Epoxides to Alcohols. Chem.–Asian J..

[5] Funk B. E., Pauze M., Lu Y.-C., Moser A. J., Wolf G., West J. G. (2023). Vitamin
B12 and Hydrogen Atom Transfer Cooperative
Catalysis as a Hydride Nucleophile Mimic in Epoxide Ring Opening. Cell Rep. Phys. Sci..

[6] Sajiki H., Hattori K., Hirota K. (1999). Pd/C­(en)-Catalyzed
Regioselective
Hydrogenolysis of Terminal Epoxides to Secondary Alcohols. Chem. Commun..

[7] Kirm I., Medina F., Rodríguez X., Cesteros Y., Salagre P. J., Sueiras E. (2005). Preparation of 2-Phenylethanol
by Catalytic Selective
Hydrogenation of Styrene Oxide Using Palladium Catalysts. J. Mol. Catal. A.

[8] Thiery E., Le Bras J., Muzart J. (2007). Palladium
Nanoparticles-Catalyzed
Regio- and Chemoselective Hydrogenolysis of Benzylic Epoxides in Water. Green Chem..

[9] Kwon M. S., Park I. S., Jang J. S., Lee J. S., Park J. (2007). Magnetically
Separable Pd Catalyst for Highly Selective Epoxide Hydrogenolysis
under Mild Conditions. Org. Lett..

[10] Dabbawala A. A., Sudheesh N., Bajaj H. C. (2012). Palladium Supported on Chitosan as
a Recyclable and Selective Catalyst for the Synthesis of 2-Phenyl
Ethanol. Dalton Trans..

[11] Nandi S., Patel P., Jakhar A., Khan N. H., Biradar A. V., Kureshy R. I., Bajaj H. C. (2017). Cucurbit­[6]­uril-Stabilized Palladium
Nanoparticles as a Highly Active Catalyst for Chemoselective Hydrogenation
of Various Reducible Groups in Aqueous Media. ChemistrySelect.

[12] Duval M., Deboos V., Hallonet A., Sagorin G., Denicourt-Nowicki A., Roucoux A. (2021). Selective Palladium
Nanoparticles-Catalyzed Hydrogenolysis
of Industrially Targeted Epoxides in Water. J. Catal..

[13] Zhou X., Wang Z., Chen Z.-N., Yang Y. (2024). Phosphine-Built-In
Porous Organic Cage Supported Ultrafine Pd Nanoclusters Enable Highly
Efficient and Regioselective Hydrogenation of Epoxides. CCS Chem..

[14] Newman M. S., Underwood G., Renoll M. (1949). The Reduction of Terminal Epoxides. J. Am. Chem. Soc..

[15] Unglaube F., Atia H., Bartling S., Kreyenschulte C. R., Mejía E. (2023). Hydrogenation of Epoxides to Anti-Markovnikov Alcohols
over a Nickel Heterogenous Catalyst Prepared from Biomass (Rice) Waste. Helv. Chim. Acta.

[16] Liu Y.-Y., Wu C.-D. (2024). Regioselective
Ring-Opening of Terminal Epoxides Catalyzed by a Porous
Metal Silicate Material. Inorg. Chem..

[17] Ito M., Hirakawa M., Osaku A., Ikariya T. (2003). Highly Efficient Chemoselective
Hydrogenolysis of Epoxides Catalyzed by a (η^5^-C5­(CH_3_)_5_)Ru Complex Bearing a 2-(Diphenylphosphino)­Ethylamine
Ligand. Organometallics.

[18] Thiyagarajan S., Gunanathan C. (2019). Ruthenium-Catalyzed Selective Hydrogenation of Epoxides
to Secondary Alcohols. Org. Lett..

[19] Kirlin F. L., Borden O. J., Head M. C., Kelly S. E., Chianese A. R. (2022). Epoxide
Hydrogenolysis Catalyzed by Ruthenium PNN and PNP Pincer Complexes. Organometallics.

[20] Borden O. J., Joseph B. T., Head M. C., Ammons O. A., Kim D. E., Bonino A. C., Keith J. M., Chianese A. R. (2024). Highly Enantiomerically
Enriched Secondary Alcohols via Epoxide Hydrogenolysis. Organometallics.

[21] Rainsberry A. N., Sage J. G., Scheuermann M. L. (2019). Iridium-Promoted Conversion of Terminal
Epoxides to Primary Alcohols under Acidic Conditions Using Hydrogen. Catal. Sci. Technol..

[22] Yao C., Dahmen T., Gansäuer A., Norton J. (2019). Anti-Markovnikov Alcohols
via Epoxide Hydrogenation through Cooperative Catalysis. Science.

[23] Liu W., Li W., Spannenberg A., Junge K., Beller M. (2019). Iron-Catalysed Regioselective
Hydrogenation of Terminal Epoxides to Alcohols under Mild Conditions. Nat. Catal..

[24] Liu W., Leischner T., Li W., Junge K., Beller M. (2020). A General
Regioselective Synthesis of Alcohols by Cobalt-Catalyzed Hydrogenation
of Epoxides. Angew. Chem., Int. Ed..

[25] Tadiello L., Gandini T., Stadler B. M., Tin S., Jiao H., Devries J. G., Pignataro L., Gennari C. (2022). Regiodivergent Reductive
Opening of Epoxides by Catalytic Hydrogenation Promoted by a (Cyclopentadienone)­Iron
Complex. ACS Catal..

[26] Magre M., Paffenholz E., Maity B., Cavallo L., Rueping M. (2020). Regiodivergent
Hydroborative Ring Opening of Epoxides via Selective C-O Bond Activation. J. Am. Chem. Soc..

[27] Zhang G., Zeng H., Zheng S., Neary M. C., Dub P. A. (2022). Markovnikov
Alcohols via Epoxide Hydroboration by Molecular Alkali Metal Catalysts. iScience.

[28] Wang J., Yao W., Hu D., Qi X., Zhang J.-Q., Ren H. (2022). NaOH/BEt_3_ Catalyzed Regioselective
Hydroboration of Epoxides to Secondary
Alcohols. Eur. J. Org Chem..

[29] Sarkar N., Sahoo R. K., Nembenna S. (2023). Aluminium-Catalyzed Selective Hydroboration
of Esters and Epoxides to Alcohols: C-O Bond Activation. Chem.—Eur. J..

[30] Zhang G., Zeng H., Tang Q., Ates S., Zheng S., Neary M. C. (2023). Vanadium-Catalysed Regioselective Hydroboration of
Epoxides for Synthesis of Secondary Alcohols. Dalton Trans..

[31] Sreejothi P., Sarkar P., Dutta S., Das A., Pati S. K., Mandal S. K. (2022). Regioselective Ring-Opening of Epoxides
Towards Markovnikov
Alcohols: a Metal-free Catalytic Approach using Abnormal N-Heterocyclic
Carbene. Chem. Commun..

[32] Wenz J., Wadepohl H., Gade L. H. (2017). Regioselective Hydrosilylation of
Epoxides Catalysed by Nickel­(II) Hydrido Complexes. Chem. Commun..

[33] Steiniger K. A., Lambert T. H. (2021). Primary
Alcohols via Nickel Pentacarboxycyclopentadienyl
Diamide Catalyzed Hydrosilylation of Terminal Epoxides. Org. Lett..

[34] Vayer M., Zhang S., Moran J., Leboeuf D. (2022). Rapid and Mild Metal-Free
Reduction of Epoxides to Primary Alcohols Mediated by HFIP. ACS Catal..

[35] Wang D., Astruc D. (2015). The Golden Age of Transfer
Hydrogenation. Chem. Rev..

[36] Kumar A., Bhardwaj R., Mandal S. K., Choudhury J. (2022). Transfer Hydrogenation
of CO_2_ and CO_2_ Derivatives using Alcohols as
Hydride Sources: Boosting an H_2_-Free Alternative Strategy. ACS Catal..

[37] Ley S. V., Mitchell C., Pears D., Ramarao C., Yu J.-Q., Zhou W. (2003). Recyclable Polyurea-Microencapsulated
Pd(0) Nanoparticles: an Efficient
Catalyst for Hydrogenolysis of Epoxides. Org.
Lett..

[38] Yao Y.-X., Zhang H.-W., Lu C.-B., Shang H.-Y., Tian Y.-Y. (2023). Highly
Selective and Practical Iron-Catalyzed Formal Hydrogenation of Epoxides
to Primary Alcohols Using Formic Acid. Eur.
J. Org Chem..

[39] Liu X., Longwitz L., Spiegelberg B., Tonjes J., Beweries T., Werner T. (2020). Erbium-Catalyzed Regioselective
Isomerization-Cobalt-Catalyzed
Transfer Hydrogenation Sequence for the Synthesis of Anti-Markovnikov
Alcohols from Epoxides under Mild Conditions. ACS Catal..

[40] Kumari S., Roy S., Ranjan Saha K., Kundu S. (2024). NHC)­Mn­(I) Complex Catalyzed Selective
Transfer Hydrogenation of Epoxides, Azoarenes and Nitroarenes Utilizing
Ammonia Borane. ChemCatChem.

[41] Huang C., Ma W., Zheng X., Xu M., Qi X., Lu Q. (2022). Epoxide Electroreduction. J. Am. Chem. Soc..

[42] Aida K., Hirao M., Funabashi A., Sugimura N., Ota E., Yamaguchi J. (2022). Catalytic Reductive Ring Opening of Epoxides Enabled
by Zirconocene and Photoredox Catalysis. Chem.

[43] Hirakawa H., Shiraishi Y., Sakamoto H., Ichikawa S., Tanaka S., Hirai T. (2015). Photocatalytic
Hydrogenolysis of Epoxides Using Alcohols as Reducing
Agents on TiO_2_ Loaded with Pt Nanoparticles. Chem. Commun..

[44] Genç S., Gülcemal S., Günnaz S., Çetinkaya B., Gülcemal D. (2021). Synthesis
of α-Alkylated Ketones via Selective
Epoxide Opening/Alkylation Reactions with Primary Alcohols. Org. Lett..

[45] Jana A., Chakraborty S., Sarkar K., Maji B. (2023). Ruthenium-Catalyzed
Reductive Coupling of Epoxides with Primary Alcohols via Hydrogen
Transfer Catalysis. J. Org. Chem..

[46] Nandi S., Borthakur I., Mandal A., Kundu S. (2024). Regioselective Ring
Opening of Epoxide with Alcohols: A Selective Route to α-Alkylated
Ketones and β-Alkylated Secondary Alcohols. ChemCatChem.

[47] Rong Q., Deng H.-B., Sun N., Che A.-Q., Hu X.-Q., Jin L.-Q. (2024). Mn-Catalyzed Ring-Opening
and Alkylation of Epoxides
with Alcohols for Accessing β-Alkylated Secondary Alcohols via
Borrowing Hydrogen. Org. Chem. Front..

[48] Genç S., Günnaz S., Çetinkaya B., Gülcemal S., Gülcemal D. (2018). Iridium­(I)-Catalyzed Alkylation Reactions to Form α-Alkylated
Ketones. J. Org. Chem..

[49] Genç S., Gülcemal S., Günnaz S., Çetinkaya B., Gülcemal D. (2020). Iridium-Catalyzed Alkylation of Secondary Alcohols
with Primary Alcohols: A Route to Access Branched Ketones and Alcohols. J. Org. Chem..

[50] Yang Y., Guo J., Ng H., Chen Z., Teo P. (2014). Formal Hydration of
non-Activated Terminal Olefins using Tandem Catalysts. Chem. Commun..

[51] Movassaghi M., Piizzi G., Siegel D. S., Piersanti G. (2006). Enantioselective
Total Synthesis of (−)-Acylfulvene and (−)-Irofulven. Angew. Chem., Int. Ed..

[52] Li K., Ou J., Gao S. (2016). Total Synthesis of Camptothecin and Related Natural
Products by a Flexible Strategy. Angew. Chem.,
Int. Ed..

[53] Liu Y.-L., Lin X.-T. (2019). Recent Advances
in Catalytic Asymmetric Synthesis of
Tertiary Alcohols via Nucleophilic Addition to Ketones. Adv. Synth. Catal..

[54] Thiery E., Le Bras J., Muzart J. (2009). Reactivity
Versus Stability of Oxiranes
under Palladium-Catalyzed Reductive Conditions. Eur. J. Org Chem..

[55] Jat J. L., Kumar G. (2019). Isomerization of Epoxides. Adv. Synth. Catal..

[56] Kulasegaram S., Kulawiec R. J. (1997). Palladium-Catalyzed Isomerization of Aryl-Substituted
Epoxides: A Selective Synthesis of Substituted Benzylic Aldehydes
and Ketones. J. Org. Chem..

[57] Vyas D. J., Larionov E., Besnard C., Guénée L., Mazet C. (2013). Isomerization of Terminal Epoxides by a [Pd–H] Catalyst: A
Combined Experimental and Theoretical Mechanistic Study. J. Am. Chem. Soc..

[58] Yang J., Yuan M., Xu D., Zhao H., Zhu Y., Fan M., Zhang F., Dong Z. (2018). Highly Dispersed Ultrafine Palladium
Nanoparticles Encapsulated in a Triazinyl Functionalized Porous Organic
Polymer as a Highly Efficient Catalyst for Transfer Hydrogenation
of Aldehydes. J. Mater. Chem. A.

[59] Yakabe S. (2010). One-Pot System
for Reduction of Epoxides Using NaBH_4_, PdCl_2_ Catalyst, and Moist Alumina. Synth. Commun..

[60] Viswanadhan M., Potdar A., Divakaran A., Badiger M., Rode C. (2016). Product Distribution
in Hydrogenation of Styrene Oxide over Pd/chitosan Catalyst. Res. Chem. Intermed..

[61] Muzart J. (2011). Pd-Mediated
Reactions of Epoxides. Eur. J. Org Chem..

[62] Hammett L. P. (1937). The Effect
of Structure upon the Reactions of Organic Compounds. Benzene Derivatives. J. Am. Chem. Soc..

[63] Kalow J. A., Doyle A. G. (2011). Mechanistic Investigations
of Cooperative Catalysis
in the Enantioselective Fluorination of Epoxides. J. Am. Chem. Soc..

[64] Tyagi A., Yadav N., Khan J., Mondal S., Hazra C. K. (2022). Brønsted
Acid-Catalysed Epoxide Ring-Opening Using Amine Nucleophiles: A Facile
Access to β-Amino Alcohols. Chem.–Asian
J..

[65] Jiménez M. V., Fernández-Tornos J., Modrego F. J., Pérez-Torrente J. J., Oro L. A. (2015). Oxidation and β-Alkylation
of Alcohols Catalysed
by Iridium­(I) Complexes with Functionalised N-Heterocyclic Carbene
Ligands. Chem.—Eur. J..

[66] Head M. C., Joseph B. T., Keith J. M., Chianese A. R. (2023). The Mechanism
of
Markovnikov-Selective Epoxide Hydrogenolysis Catalyzed by Ruthenium
PNN and PNP Pincer Complexes. Organometallics.

[67] Putignano E., Bossi G., Rigo P., Baratta W. (2012). MCl_2_(ampy)­(dppf)
(M = Ru, Os): Multitasking Catalysts for Carbonyl Compound/Alcohol
Interconversion Reactions. Organometallics.

