# Supplementary material for: Regioselective Synthesis of Alcohols by Catalytic Transfer Hydrogenation of Epoxides
Source: J Org Chem. 2025 Sep 17;90(38):13508–19. doi: 10.1021/acs.joc.5c01342 (PMC12481575; doi:10.1021/acs.joc.5c01342)
Supplement: Supplementary file 1 [file jo5c01342_si_001.pdf]

Supporting Information

Regioselective Synthesis of Alcohols by Catalytic Transfer Hydrogenation of Epoxides

Sertaç Genç,[a,b] Süleyman Gülcemal,[a] Salih Günnaz,[a] Bekir Çetinkaya,[a] Jianliang Xiao,[b] Derya Gülcemal*[a]

[a] Department of Chemistry, Ege University, 35100 Bornova, Izmir, Türkiye
[b] Department of Chemistry, University of Liverpool, Liverpool L69 7ZD, U.K.

* E-mail: derya.gulcemal@ege.edu.tr

Table of Contents

1. General Considerations……………………………………………………………..S2

2. General Procedure: Synthesis of Substrates……………………………………………..S2

3. General Procedure: Transfer Hydrogenation (TH) of Epoxides…………………… S14

4. Mechanistic Control Experiments……………………………………………………...S38

5. References……………………………...…………….………………………….......S42

6. Traces of ¹H and ¹³C NMR spectra of epoxides…………………………………..….S49

7. Traces of ¹H and ¹³C NMR spectra of isolated products……………………………...S64

1. General Considerations

Unless otherwise noted, all the reagents and solvents were commercially obtained and used without further purification. The [IrCl(cod)(NHC)] complexes **Ir1**,[1] **Ir2**,[2] and **Ir3-Ir6**[3] were synthesized according to the previous reports. 10 wt. % Pd/C was purchased from Sigma-Aldrich (catalog #205699). Epoxides (**1a**, **1d**, **1f**, **1j**, **1n**, **1o**, **2a**, and **3d**) were obtained from commercial sources and were used without further purification. The ^{1}H and $^{13}C\{^{1}H\}$ NMR spectra were recorded on a Varian AS 400 Mercury NMR spectrometer at 298 K. Chemical shifts are reported in units of parts per million (ppm) relative to tetramethyl silane (δ = 0 ppm), $CDCl_3$ (δ = 7.26 ppm for ^{1}H NMR and δ = 77.0 ppm for $^{13}C\{^{1}H\}$ NMR). Coupling constants (*J*) are reported in Hertz (Hz). The following abbreviations are used for the multiplicities: (s): singlet, (bs): broad singlet, (d): doublet, (dd): doublet of doublets, (t): triplet, (q): quartet, (m): multiplet. Melting points were measured on a Gallenkamp electrothermal melting-point apparatus without correction. Gas chromatography (GC) analyses were performed on an Agilent 6890N gas chromatograph with a HP-5 Agilent 19091J-413 column.

2. General Procedure: Synthesis of Substrates

2.1. Corey-Chaykovsky epoxidation of ketones and aldehydes (GP1a)

To a 50 mL Schlenk flask was added trimethylsulfonium iodide (1.6 equiv) and dry dimethyl sulfoxide/tetrahydrofuran (15 mL, 2:1) and then NaH (60% in mineral oil, 1.5 equiv) was added to the above mixture at 0 °C under argon. The reaction mixture was allowed to stir for 30 min at 25 °C and then ketone or aldehyde (15 mmol, 1.0 equiv) in dry tetrahydrofuran was added at 0 °C. The reaction mixture was stirred at 0 °C for 1 h and then allowed to stir at 25 °C for 24 h. After the reaction, the crude was quenched by water then was extracted with diethyl ether. The combined organic layers were washed with brine and dried over anhydrous Na_2SO_4. The solvent was concentrated under reduced pressure and the crude product was purified by vacuum distillation or column chromatography (SiO_2, hexane:ethyl acetate with Et_3N).

2.2. Epoxidation of alkenes using *m*-CPBA (GP1b)

To a 50 mL two-necked Schlenk flask was added alkene (10 mmol, 1.0 equiv), $NaHCO_3$ (1.3 equiv) and dichloromethane (20 mL) under argon atmosphere. The *m*-CPBA solution (73%, 1.2 equiv.) in 20 mL dichloromethane was then added dropwise through a dropping funnel over 20 minutes at 0 ºC. The reaction was then stirred for an additional 1 h and then allowed to warm to 25 °C. After completion (TLC monitoring), the reaction was quenched by aqueous $Na_2S_2O_3$ and extracted with dichloromethane. The combined organic layers were washed with a saturated solution of $NaHCO_3$ and brine, then dried over anhydrous Na_2SO_4. The solvent was concentrated under reduced pressure and the crude product was purified by column chromatography (SiO_2, hexane:ethyl acetate with Et_3N).

2.3. Characterization data of substrates

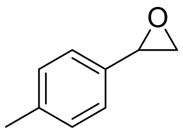

2-(p-tolyl)oxirane (1b).[4] Following the general procedure (GP1a), the title product was isolated by vacuum distillation: yield 1.45 g, 72%. Colorless oil. ^{1}H NMR ($CDCl_3$, 400 MHz): δ (ppm) 7.20-7.15 (m, 4H), 3.84 (dd, J_1 = 4.0 Hz, J_2 = 2.4 Hz, 1H), 3.13 (dd, J_1 = 5.2 Hz, J_2 = 4.0 Hz, 1H), 2.80 (dd, J_1 = 5.6 Hz, J_2 = 2.4 Hz, 1H), 2.35 (s, 3H).

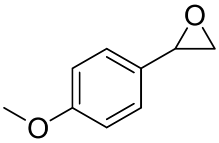

2-(4-methoxyphenyl)oxirane (1c).[4] Following the general procedure (GP1a), the title product was isolated by using silica gel column chromatography eluting with hexane:ethyl acetate (80:20) with 1.0% Et_3N: yield 1.51 g, 67%. Colorless oil. ^{1}H NMR ($CDCl_3$, 400 MHz): δ (ppm)

7.20 (d, J = 8.4 Hz, 2H), 6.88 (d, J = 8.4 Hz, 2H), 3.85-3.78 (m, 1H), 3.80 (s, 3H), 3.12 (dd, J_1 = 5.2 Hz, J_2 = 4.0 Hz, 1H), 2.80 (dd, J_1 = 5.6 Hz, J_2 = 2.8 Hz, 1H).

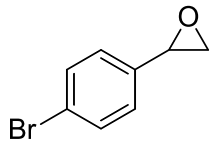

2-(4-bromophenyl)oxirane (1e).[4] Following the general procedure (GP1a), the title product was isolated by using silica gel column chromatography eluting with hexane:ethyl acetate (95:5) with 1.0% Et_3N: yield 2.09 g, 69%. White solid (m.p.: 98-100 °C). ^{1}H NMR ($CDCl_3$, 400 MHz): δ (ppm) 7.46 (d, J = 8.4 Hz, 2H), 7.15 (d, J= 8.0 Hz, 2H), 3.82 (t, J = 3.2 Hz, 1H), 3.14 (dd, J_1 = 5.6 Hz, J_2 = 4.0 Hz, 1H), 2.74 (dd, J_1 = 5.6 Hz, J_2 = 2.8 Hz, 1H).

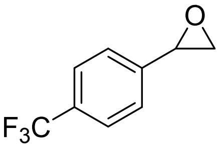

2-(4-(trifluoromethyl)phenyl)oxirane (1g).[4,5] Following the general procedure (GP1a), the title product was isolated by using silica gel column chromatography eluting with hexane:ethyl acetate (95:5) with 1.0% Et_3N: yield 1.47 g, 52%. Colorless oil. ^{1}H NMR ($CDCl_3$, 400 MHz): δ (ppm) 7.60 (d, J = 8.0 Hz, 2H), 7.39 (d, J= 8.0 Hz, 2H), 3.91 (dd, J_1 = 4.0 Hz, J_2 = 2.4 Hz, 1H), 3.19 (dd, J_1 = 5.2 Hz, J_2 = 4.4 Hz, 1H), 2.77 (dd, J_1 = 5.6 Hz, J_2 = 2.4 Hz, 1H).

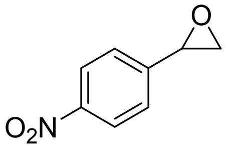

2-(4-nitrophenyl)oxirane (1h).[6] Following the general procedure (GP1b), the title product was isolated by using silica gel column chromatography eluting hexane:ethyl acetate (90:10) with 1.0% Et_3N: yield 1.80 g, 73%. Yellow solid (m.p.: 84-86 °C). ^{1}H NMR ($CDCl_3$, 400 MHz): δ

(ppm) 8.19 (d, J= 8.8 Hz, 2H), 7.44 (d, J= 8.8 Hz, 2H), 3.95 (dd, J_1 = 4.4 Hz, J_2 = 2.4 Hz, 1H), 3.22 (dd, J_1 = 5.6 Hz, J_2 = 4.0 Hz, 1H), 2.77 (dd, J_1 = 5.6 Hz, J_2 = 2.4 Hz, 1H).

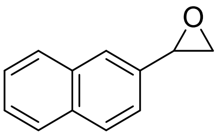

2-(naphthalen-2-yl)oxirane (1i).[4,5] Following the general procedure (GP1a), the title product was isolated by using silica gel column chromatography eluting with hexane:ethyl acetate (95:5) with 1.0% Et_3N: yield 2.01 g, 79%. White solid (m.p.: 56-58 °C). ^1H NMR ($CDCl_3$, 400 MHz): δ (ppm) 7.85-7.81 (m, 4H), 7.52-7.46 (m, 2H), 7.34 (dd, J_1 = 8.4 Hz, J_2 = 2.0 Hz, 1H), 4.04 (dd, J_1 = 4.0 Hz, J_2 = 2.4 Hz, 1H), 3.23 (dd, J_1 = 5.2 Hz, J_2 = 4.0 Hz, 1H), 2.91 (dd, J_1 = 5.2 Hz, J_2 = 2.8 Hz, 1H).

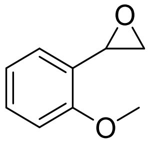

2-(2-methoxyphenyl)oxirane (1k).[7] Following the general procedure (GP1a), the title product was isolated by using silica gel column chromatography eluting with hexane:ethyl acetate (90:10) with 1.0% Et_3N: yield 1.52 g, 68%. Colorless oil. ^1H NMR ($CDCl_3$, 400 MHz): δ (ppm) 7.26 (t, J = 4.0 Hz, 1H), 7.15 (d, J = 7.6 Hz, 1H), 6.96-6.87 (m, 2H), 4.20 (dd, J_1 = 4.0 Hz, J_2 = 2.8 Hz, 1H), 3.87 (s, 3H), 3.14 (dd, J_1 = 5.6 Hz, J_2 = 4.4 Hz, 1H), 2.70 (dd, J_1 = 5.6 Hz, J_2 = 2.4 Hz, 1H).

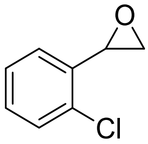

2-(2-chlorophenyl)oxirane (1l).[7] Following the general procedure (GP1a), the title product was isolated by using silica gel column chromatography eluting with hexane:ethyl acetate

(95:5) with 1.0% Et_3N: yield 1.87 g, 81%. Colorless oil. ^{1}H NMR ($CDCl_3$, 400 MHz): δ (ppm) 7.37-7.34 (m, 1H), 7.27-7.21 (m, 3H), 4.21 (dd, J_1 = 4.4 Hz, J_2 = 2.4 Hz, 1H), 3.19 (dd, J_1 = 6.0 Hz, J_2 = 4.0 Hz, 1H), 2.66 (dd, J_1 = 5.6 Hz, J_2 = 2.4 Hz, 1H).

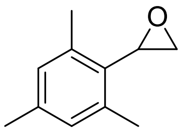

2-mesityloxirane (1m).[8] Following the general procedure (GP1a), the title product was isolated by using silica gel column chromatography eluting with hexane:ethyl acetate (90:10) with 1.0% Et_3N: yield 1.48 g, 61%. Colorless oil. ^{1}H NMR ($CDCl_3$, 400 MHz): δ (ppm) 6.85 (s, 2H), 3.92 (t, J= 3.2 Hz, 1H), 3.18 (dd, J_1 = 5.6 Hz, J_2 = 4.0 Hz, 1H), 2.76 (dd, J_1 = 5.6 Hz, J_2 = 2.8 Hz, 1H).

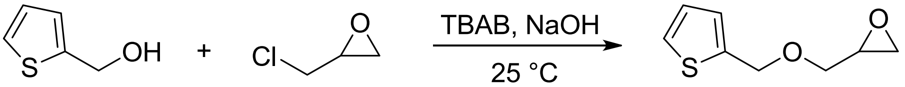

1p

2-((thiophen-2-ylmethoxy)methyl)oxirane (1p).[9] Epichlorohydrin (60 mmol) was slowly added to a cold mixture of 40% w/w aqueous NaOH (25 mL), thiophen-2-ylmethanol (15 mmol) and tetrabutylammonium bromide (0.75 mmol). The reaction was allowed to warm up to 25 °C and after completion of the reaction (TLC monitoring), the mixture was extracted with Et_2O. The combined organic layers were dried over anhydrous Na_2SO_4. The solvent was concentrated under reduced pressure and the crude product was purified by vacuum distillation: yield 2.34 g, 92%. Colorless oil. ^{1}H NMR ($CDCl_3$, 400 MHz): δ (ppm) 7.29 (dd, J_1 = 5.2 Hz, J_2 = 1.2 Hz, 1H), 7.02-7.00 (m, 1H), 6.98-6.95 (m, 1H), 4.74 (q, J = 12.4 Hz, 2H), 3.76 (dd, J_1 = 11.2 Hz, J_2 = 3.2 Hz, 1H), 3.44 (dd, J_1 = 11.6 Hz, J_2 = 5.6 Hz, 1H), 3.19-3.14 (m, 1H), 2.79 (t, J = 4.8 Hz, 1H), 2.61 (dd, J_1 = 5.2 Hz, J_2 = 2.4 Hz, 1H).

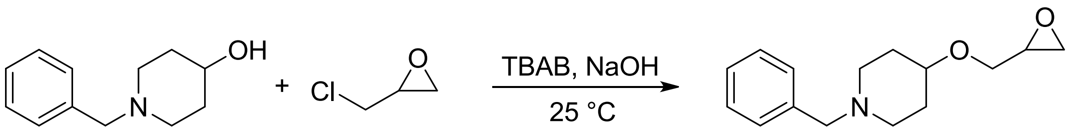

1q

1-benzyl-4-(oxiran-2-ylmethoxy)piperidine (1q).[10] Epichlorohydrin (75 mmol) was slowly added to a cold mixture of 12 N aqueous NaOH (10 mL), 1-benzylpiperidin-4-ol (15 mmol) and tetrabutylammonium bromide (0.75 mmol). The reaction was allowed to warm up to 25 °C and was stirred for 48 h. After completion of the reaction, the mixture was extracted with dichloromethane. The combined organic layers were dried over anhydrous Na_2SO_4. The solvent was concentrated under reduced pressure and the crude product was purified by silica gel column chromatography eluting with hexane:ethyl acetate (50:50) with 1.0% Et_3N: yield 2.22 g, 60%. Colorless oil. ^{1}H NMR ($CDCl_3$, 400 MHz): δ (ppm) 7.31-7.21 (m, 5H), 3.70 (dd, J_1 = 11.2 Hz, J_2 = 3.2 Hz, 1H), 3.48 (s, 2H), 3.45-3.35 (m, 2H), 3.15-3.11 (m, 1H), 2.78 (t, J = 4.0 Hz, 1H), 2.75-2.72 (m, 2H), 2.60 (dd, J_1 = 5.2 Hz, J_2 = 2.8 Hz, 1H), 2.13 (t, J = 10.4 Hz, 2H), 1.90-1.86 (m, 2H), 1.67-1.56 (m, 2H).

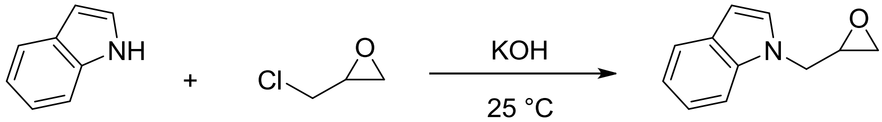

1r

1-(oxiran-2-ylmethyl)-1H-indole (1r). [11] To a 50 mL three-necked flask equipped with a reflux condenser was added indole (15 mmol) and then epichlorohydrin (75 mmol) was slowly added under argon atmosphere. The reaction was allowed to stir at 60 °C for 30 min and then KOH (22.5 mmol) was added and stirred for an additional 10 h. After completion of the reaction, the mixture was filtered and washed with dichloromethane. Subsequently, the organic layer was extracted with water and the combined organic layers were dried over anhydrous Na_2SO_4. The solvent was concentrated under reduced pressure and the crude product was purified by silica gel column chromatography eluting with hexane:ethyl acetate (95:5) with 1.0% Et_3N: yield 1.76

g, 68%. Dark yellow oil. ^{1}H NMR ($CDCl_3$, 400 MHz): δ (ppm) 7.67 (d, J = 8.0 Hz, 1H), 7.41 (d, J = 8.4 Hz, 1H), 7.26 (t, J = 7.2 Hz, 1H), 7.18-7.14 (m, 2H), 6.56 (d, J = 2.8 Hz, 1H), 4.43 (dd, J_1 = 15.6 Hz, J_2 = 3.2 Hz, 1H), 4.19 (dd, J_1 = 15.2 Hz, J_2 = 5.2 Hz, 1H), 3.31-3.27 (m, 1H), 2.81 (t, J = 4.0 Hz, 1H), 2.47 (dd, J_1 = 5.2 Hz, J_2 = 2.8 Hz, 1H).

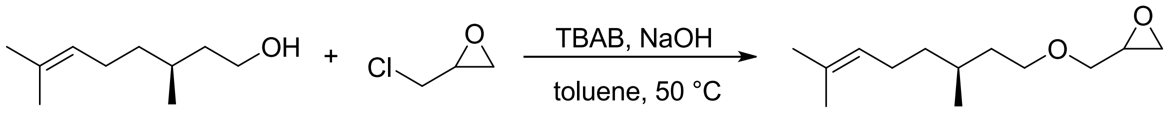

1s

2-((((S)-3,7-dimethyloct-6-en-1-yl)oxy)methyl)oxirane (1s).[12] Tetrabutylammonium bromide (1.6 mmol) and 48% w/w aqueous NaOH (8 mL) were added to a solution of citronellol (32 mmol) in toluene (32 mL). Epichlorohydrin (64 mmol) was then added to the solution at 25 °C and the reaction mixture was stirred at 50 °C for 6 h. After the reaction, the crude product was diluted with dichloromethane and washed with water. The organic layer was dried over anhydrous Na_2SO_4. The solvent was concentrated under reduced pressure and the crude product was purified by silica gel column chromatography eluting with hexane:ethyl acetate (80:20) with 1.0% Et_3N: yield 5,91 g, 87%. Colorless oil. ^{1}H NMR ($CDCl_3$, 400 MHz): δ (ppm) 5.08 (t, J = 6.8 Hz, 1H), 3.71-3.67 (m, 1H), 3.57-3.45 (m, 2H), 3.39-3.35 (m, 1H), 3.15-3.11 (m, 1H), 2.78 (t, J = 5.2 Hz, 1H), 2.59 (dd, J_1 = 5.2 Hz, J_2 = 2.8 Hz, 1H), 2.04-1.89 (m, 2H), 1.69-1.51 (m, 9H), 1.43-1.28 (m, 2H), 1.20-1.11 (m, 1H), 0.88 (d, J = 6.4 Hz, 3H).

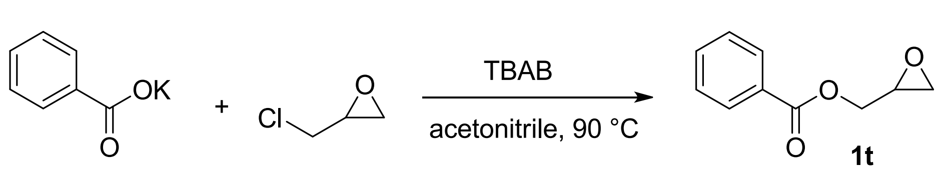

1t

phenyl 2-(oxiran-2-yl)acetate (1t).[13] To a 100 mL three-necked flask equipped with a reflux condenser was added epichlorohydrin (38 mmol), potassium benzoate (25 mmol), tetrabutylammonium bromide (0.84 mmol) and acetonitrile (20 mL). The reaction was allowed to stir at 90 °C for 5 h. After completion of the reaction, the solvent was removed under reduced

pressure and the residue was extracted with ethyl acetate and water. The organic layer was then washed with washed with saturated solution of $NaHCO_3$ and water. The combined organic layers were dried over anhydrous Na_2SO_4. The solvent was concentrated under reduced pressure and the crude product was purified by silica gel column chromatography eluting with hexane:ethyl acetate (90:10) with 1.0% Et_3N: yield 2.49 g, 56%. Colorless oil. ^{1}H NMR ($CDCl_3$, 400 MHz): δ (ppm) 8.08-7.99 (m, 2H), 7.59-7.51 (m, 1H), 7.46-7.38 (m, 2H), 4.65 (dd, J_1 = 12.4 Hz, J_2 = 3.2 Hz, 1H), 4.17 (dd, J_1 = 12.4 Hz, J_2 = 6.0 Hz, 1H), 3.36-3.32 (m, 1H), 2.89 (t, J = 4.0 Hz, 1H), 2.73 (dd, J_1 = 4.8 Hz, J_2 = 2.2 Hz, 1H).

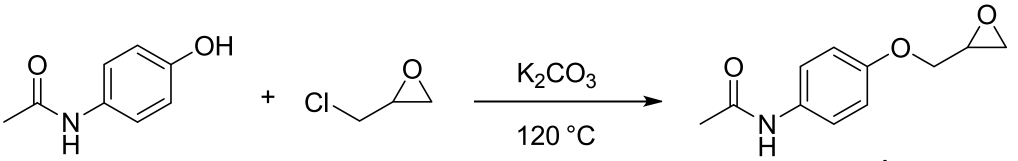

N-(4-(oxiran-2-ylmethoxy)phenyl)acetamide (1u).[14] A mixture of paracetamol (15 mmol), K_2CO_3 (18 mmol) and epichlorohydrin (75 mmol) was stirred at 120 °C for 5h. After completion of the reaction, the mixture was filtered. The filtrate was diluted with water (50 mL) and extracted with ethyl acetate. The combined organic layers were dried over anhydrous Na_2SO_4. The solvent was concentrated under reduced pressure and the crude product was purified by silica gel column chromatography eluting with dichloromethane:methanol (90:10) with 1.0% Et_3N: yield 2.17 g, 70%. White solid (m.p.: 85-87 °C). ^{1}H NMR ($CDCl_3$, 400 MHz): δ (ppm) 7.70 (bs, 1H), 7.37 (d, J = 8.8 Hz, 2H), 6.82 (d, J = 9.2 Hz, 2H), 4.18 (dd, J_1 = 10.8 Hz, J_2 = 3.2 Hz, 1H), 3.88 (dd, J_1 = 10.8 Hz, J_2 = 6.0 Hz, 1H), 3.34-3.31 (m, 1H), 2.88 (t, J = 4.4 Hz, 1H), 2.74 (dd, J_1 = 4.8 Hz, J_2 = 2.4 Hz, 1H), 2.11 (s, 3H).

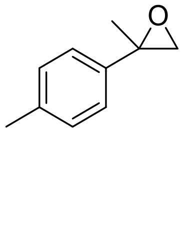

2-methyl-2-(p-tolyl)oxirane (2b).[17] Following the general procedure (GP1a), the title product was isolated by vacuum distillation: yield 1.82 g, 82%. Colorless oil. ^{1}H NMR ($CDCl_3$, 400 MHz): δ (ppm) 7.27 (d, J = 7.6 Hz, 2H), 7.16 (d, J = 8.0 Hz, 2H), 2.96 (d, J = 5.2 Hz, 1H), 2.80 (d, J = 5.2 Hz, 1H), 2.35 (s, 3H), 1.71 (s, 3H).

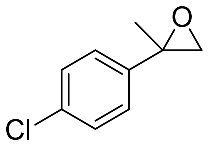

2-(4-chlorophenyl)-2-methyloxirane (2c).[15,17] Following the general procedure (GP1a), the title product was isolated by vacuum distillation: yield 2.11 g, 84%. Colorless oil. ^{1}H NMR ($CDCl_3$, 400 MHz): δ (ppm) 7.29 (s, 4H), 2.97 (d, J = 5.6 Hz, 1H), 2.75 (d, J = 5.6 Hz, 1H), 1.69 (s, 3H).

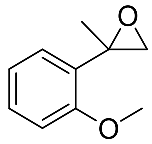

2-(2-methoxyphenyl)-2-methyloxirane (2d).[17] Following the general procedure (GP1a), the title product was isolated by vacuum distillation: yield 1.67 g, 68%. Colorless oil. ^{1}H NMR ($CDCl_3$, 400 MHz): δ (ppm) 7.37 (d, J = 7.6 Hz, 1H), 7.26 (t, J = 8.4 Hz, 1H), 6.93 (t, J = 7.6 Hz, 1H), 6.87 (d, J = 8.4 Hz, 1H), 3.87 (s, 3H), 2.93 (d, J = 5.2 Hz, 1H), 2.76 (d, J = 5.2 Hz, 1H), 1.63 (s, 3H).

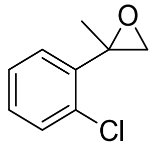

2-(2-chlorophenyl)-2-methyloxirane (2e).[16] Following the general procedure (GP1a), the title product was isolated by vacuum distillation: yield 1.81 g, 72%. Colorless oil. ^{1}H NMR ($CDCl_3$,

400 MHz): δ (ppm) 7.49-7.47 (m, 1H), 7.34-7.32 (m, 1H), 7.28-7.20 (m, 2H), 3.00 (d, J = 5.2 Hz, 1H), 2.83 (d, J = 4.8 Hz, 1H), 1.65 (s, 3H).

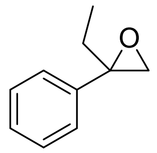

2-ethyl-2-phenyloxirane (2f).[15,16] Following the general procedure (GP1a), the title product was isolated by vacuum distillation: yield 1.75 g, 79%. Colorless oil. ^{1}H NMR ($CDCl_3$, 400 MHz): δ (ppm) 7.39-7.26 (m, 5H), 2.98 (d, J = 5.6 Hz, 1H), 2.75 (d, J = 5.6 Hz, 1H), 2.24-2.15 (m, 1H), 1.86-1.77 (m, 1H), 0.94 (t, J = 7.6 Hz, 3H).

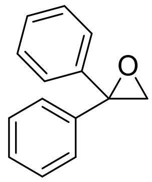

2,2-diphenyloxirane (2g).[15] Following the general procedure (GP1a), the title product was isolated by using silica gel column chromatography eluting with hexane:ethyl acetate (95:5) with 1.0% Et_3N: yield 2.26 g, 77%. White solid (m.p.: 54-56 °C). ^{1}H NMR ($CDCl_3$, 400 MHz): δ (ppm) 7.39-7.31 (m, 10H), 3.30 (s. 2H).

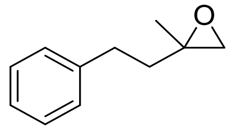

2-methyl-2-phenethyloxirane (2h).[15] Following the general procedure (GP1a), the title product was isolated by using silica gel column chromatography eluting with hexane:ethyl acetate (90:10) with 1.0% Et_3N: yield 1.45 g, 60%. Colorless oil. ^{1}H NMR ($CDCl_3$, 400 MHz): δ (ppm) 7.31-7.18 (m, 5H), 2.73 (t, J = 7.6 Hz, 2H), 2.59 (dd, J_1 = 11.2 Hz, J_2 = 4.8 Hz, 2H). 1.98-1.80 (m, 2H), 1.39 (s, 3H).

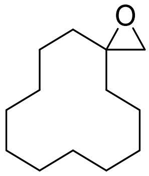

1-oxaspiro[2.11]tetradecane (2i).[18] Following the general procedure (GP1a), the title product was isolated by using silica gel column chromatography eluting with hexane:ethyl acetate (99:1) with 1.0% Et_3N: yield 2.52 g, 86%. Colorless oil. ^{1}H NMR ($CDCl_3$, 400 MHz): δ (ppm) 2.58 (s, 2H), 1.71-1.63 (m, 2H), 1.54-1.37 (m, 20H).

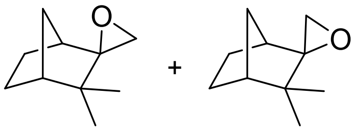

endo-**2j** exo-**2j**

(1:2)

3,3-dimethylspiro[bicyclo[2.2.1]heptane-2,2'-oxirane] (2j).[5] Following the general procedure (GP1b), the title product was isolated by vacuum distillation: yield 1.19 g, 78%. White solid (m.p.: 86-88 °C).^{1}H NMR ($CDCl_3$, 400 MHz): δ (ppm) 2.79 (d, J = 4.8 Hz, 0.25H), 2.76 (d, J = 4.9 Hz, 0.25H), 2.70 (d, J = 4.6 Hz, 0.50H), 2.68 (d, J = 4.6 Hz, 0.50H), 2.04–1.56 (m, 5H), 1.51–1.18 (m, 3H), 0.97 (s, 0.75H), 0.91 (s, 3H), 0.82 (s, 0.75H).

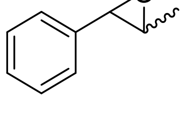

2-methyl-3-phenyloxirane (3a).[15,19] Following the general procedure (GP1b), the title product was isolated by using silica gel column chromatography eluting with hexane:ethyl acetate (95:5) with 1.0% Et_3N: yield 1.09 g, 82%. Colorless oil. ^{1}H NMR ($CDCl_3$, 400 MHz): δ (ppm) 7.37-7.27 (m, 5H), 4.06 (d, J = 4.4 Hz, 1H), 3.37-3.32 (m, 1H), 1.09 (d, J = 5.6 Hz, 3H).

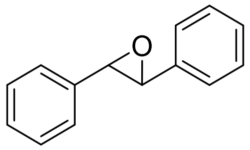

2,3-diphenyloxirane (3b).[20] Following the general procedure (GP1b), the title product was isolated by using silica gel column chromatography eluting with hexane:ethyl acetate (95:5) with 1.0% Et_3N: yield 1.84 g, 94%. White solid (m.p.: 78-80 °C). ^1H NMR ($CDCl_3$, 400 MHz): δ (ppm) 7.41-7.32 (m, 10H), 3.88 (s, 3H).

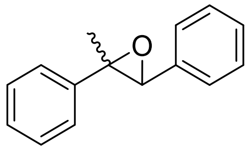

2-methyl-2,3-diphenyloxirane (3c).[17] Following the general procedure (GP1b), the title product was isolated by using silica gel column chromatography eluting with hexane:ethyl acetate (90:10) with 1.0% Et_3N: yield 1.21 g, 58%. White solid (m.p.: 46-48 °C). ^1H NMR ($CDCl_3$, 400 MHz): δ (ppm) 7.48-7.30 (m, 10H), 3.98 (s, 1H), 1.47 (s, 3H).

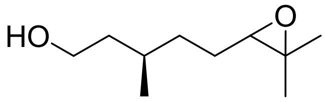

(3S)-5-(3,3-dimethyloxiran-2-yl)-3-methylpentan-1-ol (3e).[21] Following the general procedure (GP1b), the title product was isolated by vacuum distillation: yield 1.38 g, 80%. Colorless oil. ^1H NMR ($CDCl_3$, 400 MHz): δ (ppm) 3.67-3.56 (m, 2H), 2.61 (t, J = 6.0 Hz, 1H), 2.34 (bs, 1H), 1.59-1.33 (m, 7H), 1.25 (s, 3H), 1.22 (s, 3H), 0.87 (d, J = 7.6 Hz, 3H).

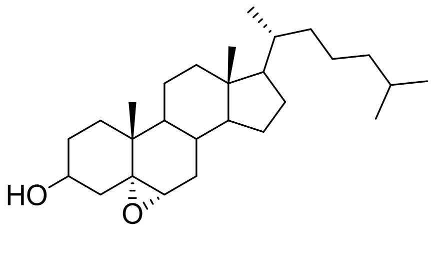

(9aR,11bR)-9a,11b-dimethyl-9-((R)-6-methylheptan-2-yl)hexadecahydrocyclopenta[1,2] phenanthro[8a,9-b]oxiren-3-ol (3f).[22,23] To a solution of cholesterol (2.6 mmol) in chloroform (100 mL), *m*-CPBA (2.8 mmol) was added at 25 °C and the mixture was stirred for 18 h under argon atmosphere. After completion, the reaction was quenched by aqueous $Na_2S_2O_3$ and extracted with chloroform. The combined organic layers were washed with water and then dried over anhydrous Na_2SO_4. The solvent was concentrated under reduced pressure and the crude solid was recrystallized from ethyl acetate and hexane (1:5): yield 0.87 g, 84%. White solid (m.p.: 142-144 °C). ^{1}H NMR ($CDCl_3$, 400 MHz): δ (ppm) 3.90 (m, 1H), 2.89 (d, *J* = 4.4 Hz, 1H), 2.06 (t, *J* = 11.6 Hz, 1H), 1.95-1.88 (m, 4H), 1.69-1.25 (m, 21H), 1.12-0.98 (m, 5H), 0.89-0.84 (m, 10H), 0.60 (s, 3H).

3. General Procedure: Transfer Hydrogenation (TH) of Epoxides

3. 1. NHC−Ir catalyzed TH of monosubstituted terminal epoxides (GP2a)

Alcohols **4a**-**4s** were prepared according to the **GP2a**: To a 20 mL reaction tube with a condenser, epoxide (0.5 mmol), KOH (2.8 mg, 0.05 mmol; 10 mol%), **Ir6** (2.1 mg, 0.0025 mmol, 0.5 mol%) and 2-propanol (1.0 mL) were added under open air conditions. The reaction mixture was vigorously stirred under reflux in a preheated oil bath at 95 °C for 16 h. Thereafter, the reaction mixture was cooled to ambient temperature and the reaction mixture was diluted with 5 mL dichloromethane. After filtration, the solvent was evaporated, and the crude product was purified by column chromatography over silica gel.

3. 2. NHC−Ir catalyzed TH of 2,2-disubstituted terminal epoxides (GP2b)

Alcohols **6a**-**6i** were prepared according to the **GP2b**: To a 20 mL reaction tube with a condenser, epoxide (0.5 mmol), KOH (5.6 mg, 0.1 mmol; 20 mol%), **Ir6** (4.2 mg, 0.005 mmol, 1 mol%) and 2-propanol (1.0 mL) were added under open air conditions. The reaction mixture was vigorously stirred under reflux in a preheated oil bath at 135 °C for 20 h. Thereafter, the

reaction mixture was cooled to ambient temperature and the reaction mixture was diluted with 5 mL dichloromethane. After filtration, the solvent was evaporated, and the crude product was purified by column chromatography over silica gel.

3. 3. NHC−Ir & Pd/C catalyzed TH of monosubstituted terminal aryl epoxides (GP2c)

Alcohols **5a**-**5k** were prepared according to the **GP2c**: To a 20 mL reaction tube with a condenser, epoxide (0.5 mmol), Cs_2CO_3 (16.3 mg, 0.05 mmol; 10 mol%), **Ir6** (2.1 mg, 0. 0025 mmol, 0.5 mol%), 10% **Pd/C** (10.6 mg, 0.01 mmol, 2 mol%) and 2-propanol (2.0 mL) were added under open air conditions. The reaction mixture was vigorously stirred under reflux in a preheated oil bath at 95 °C for 20 h. Thereafter, the reaction mixture was cooled to ambient temperature and the reaction mixture was diluted with 5 mL dichloromethane. After filtration, the solvent was evaporated, and the crude product was purified by column chromatography over silica gel.

3. 4. NHC−Ir & Pd/C catalyzed TH of monosubstituted terminal alkyl epoxides (GP2d)

Alcohols **4n**, **4o** and **4u** were prepared according to the **GP2d**: To a 20 mL reaction tube with a condenser, epoxide (0.5 mmol), Na_2CO_3 (5.3 mg, 0.05 mmol; 10 mol%), **Ir6** (2.1 mg, 0. 0025 mmol, 0.5 mol%), 10% **Pd/C** (10.6 mg, 0.01 mmol, 2 mol%) and 2-propanol (2.0 mL) were added under open air conditions. The reaction mixture was vigorously stirred under reflux in a preheated oil bath at 95 °C for 20 h. Thereafter, the reaction mixture was cooled to ambient temperature and the reaction mixture was diluted with 5 mL dichloromethane. After filtration, the solvent was evaporated, and the crude product was purified by column chromatography over silica gel.

3. 5. NHC−Ir & Pd/C catalyzed TH of 2,2-disubstituted terminal epoxides (GP2e)

Alcohols **7a**-**7f** were prepared according to the **GP2e**: To a 20 mL reaction tube with a condenser, epoxide (0.5 mmol), Cs_2CO_3 (16.3 mg, 0.05 mmol; 10 mol%), **Ir6** (2.1 mg, 0. 0025

mmol, 0.5 mol%), **Pd/C** (15.9 mg, 0.015 mmol, 3 mol%) and 2-propanol (2.0 mL) were added under open air conditions. The reaction mixture was vigorously stirred under reflux in a preheated oil bath at 95 °C for 20 h. Thereafter, the reaction mixture was cooled to ambient temperature and the reaction mixture was diluted with 5 mL dichloromethane. After filtration, the solvent was evaporated, and the crude product was purified by column chromatography over silica gel.

3. 6. NHC−Ir & Pd/C catalyzed TH of internal epoxides (GP2f)

Alcohols **8a**-**8d** were prepared according to the **GP2f**: To a 20 mL reaction tube with a condenser, epoxide (0.5 mmol), KOH (5.6 mg, 0.1 mmol; 20 mol%), **Ir6** (2.1 mg, 0. 0025 mmol, 0.5 mol%), **Pd/C** (21.3 mg, 0.02 mmol, 4 mol%) and 2-propanol (2.0 mL) were added under open air conditions. The reaction mixture was vigorously stirred under reflux in a preheated oil bath at 95 °C for 20 h. Thereafter, the reaction mixture was cooled to ambient temperature and the reaction mixture was diluted with 5 mL dichloromethane. After filtration, the solvent was evaporated, and the crude product was purified by column chromatography over silica gel.

3.7. Characterization data of products

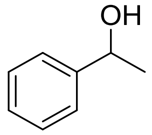

1-phenylethanol (4a).[24] Following the general procedure (GP2a), the title product **4a** (56 mg, 91% yield) was isolated by using silica gel column chromatography eluting with hexane and ethyl acetate (8:2) mixture, starting from 2-phenyloxirane (60 mg, 0.5 mmol), KOH (2.8 mg, 0.05 mmol, 10 mol%) and **Ir6** (2.1 mg, 0.0025 mmol, 0.5 mol%) in 2-propanol (1 mL) at 95 ºC. Colorless oil. ^{1}H NMR ($CDCl_3$, 400 MHz): δ (ppm) 7.38-7.26 (m, 4H), 4.86 (q, J = 6.4 Hz,

1H), 2.34 (bs, 1H), 1.48 (d, J = 6.8 Hz, 3H); $^{13}C\{^1H\}$ NMR ($CDCl_3$, 100 MHz): δ (ppm) 145.9, 128.5, 127.4, 125.4, 70.3, 25.1.

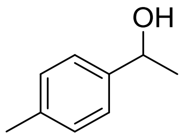

1-(p-tolyl)ethanol (4b).[24,25] Following the general procedure (GP2a), the title product **4b** (57 mg, 84% yield) was isolated by using silica gel column chromatography eluting with hexane and ethyl acetate (8:2) mixture, starting from 2-(p-tolyl)oxirane (67 mg, 0.5 mmol), KOH (2.8 mg, 0.05 mmol, 10 mol%) and **Ir6** (2.1 mg, 0.0025 mmol, 0.5 mol%) in 2-propanol (1 mL) at 95 ºC. Colorless oil. ^1H NMR ($CDCl_3$, 400 MHz): δ (ppm) 7.29 (d, J = 8.0 Hz, 2H), 7.20 (d, J = 7,6 Hz, 2H), 4.89 (q, J = 6.4 Hz, 1H), 2.37 (s, 3H), 1.88 (bs, 1H), 1.51 (d, J = 6.4 Hz, 3H); $^{13}C\{^1H\}$ NMR ($CDCl_3$, 100 MHz): δ (ppm) 142.9, 137.1, 129.2, 125.4, 70.3, 25.1, 21.1.

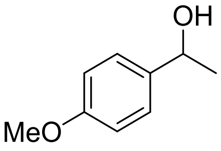

1-(4-methoxyphenyl)ethanol (4c).[26] Following the general procedure (GP2a), the title product **4c** (55 mg, 72% yield) was isolated by using silica gel column chromatography eluting with hexane and ethyl acetate (7:3) mixture, starting from 2-(4-methoxyphenyl)oxirane (75 mg, 0.5 mmol), KOH (2.8 mg, 0.05 mmol, 10 mol%) and **Ir6** (2.1 mg, 0.0025 mmol, 0.5 mol%) in 2-propanol (1 mL) at 95 ºC. Colorless oil. ^1H NMR ($CDCl_3$, 400 MHz): δ (ppm) 7.32 (d, J = 8.8 Hz, 2H), 6.91 (d, J = 8,8 Hz, 2H), 4.88 (q, J = 6.4 Hz, 1H), 3.83 (s, 3H), 1.88 (bs, 1H), 1.50 (d, J = 6.4 Hz, 3H); $^{13}C\{^1H\}$ NMR ($CDCl_3$, 100 MHz): δ (ppm) 158.9, 138.0, 126.7, 113.9, 69.9, 55.3, 25.0.

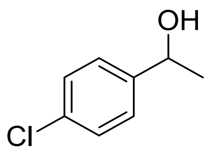

1-(4-chlorophenyl)ethanol (4d).[25,26] Following the general procedure (GP2a), the title product **4d** (72 mg, 92% yield) was isolated by using silica gel column chromatography eluting with hexane and ethyl acetate (8:2) mixture, starting from 2-(4-chlorophenyl)oxirane (77 mg, 0.5 mmol), KOH (2.8 mg, 0.05 mmol, 10 mol%) and **Ir6** (2.1 mg, 0.0025 mmol, 0.5 mol%) in 2-propanol (1 mL) at 95 ºC. Colorless oil. ^{1}H NMR ($CDCl_3$, 400 MHz): δ (ppm) 7.35-7.30 (m, 4H), 4.89 (q, J = 6.4 Hz, 1H), 1.92 (bs, 1H), 1.49 (d, J = 6.4 Hz, 3H); ^{13}C{^{1}H} NMR ($CDCl_3$, 100 MHz): δ (ppm) 144.2, 133.1, 128.6, 126.8, 69.7, 25.3.

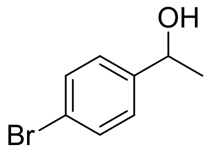

1-(4-bromophenyl)ethanol (4e).[25,27] Following the general procedure (GP2a), the title product **4e** (95 mg, 95% yield) was isolated by using silica gel column chromatography eluting with hexane and ethyl acetate (8:2) mixture, starting from 2-(4-bromophenyl)oxirane (99 mg, 0.5 mmol), KOH (2.8 mg, 0.05 mmol, 10 mol%) and **Ir6** (2.1 mg, 0.0025 mmol, 0.5 mol%) in 2-propanol (1 mL) at 95 ºC. White solid (m.p.: 40-42 ºC). ^{1}H NMR ($CDCl_3$, 400 MHz): δ (ppm) 7.49 (d, J = 8.4 Hz, 2H), 7.27 (d, J = 8.4 Hz, 2H), 4.89 (q, J = 6.4 Hz, 1H), 1.86 (bs, 1H), 1.49 (d, J = 6.8 Hz, 3H); ^{13}C{^{1}H} NMR ($CDCl_3$, 100 MHz): δ (ppm) 144.8, 131.6, 127.2, 121.2, 69.8, 25.3.

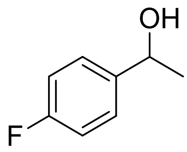

1-(4-fluorophenyl)ethanol (4f).[24,25] Following the general procedure (GP2a), the title product **4f** (59 mg, 84% yield) was isolated by using silica gel column chromatography eluting with hexane and ethyl acetate (8:2) mixture, starting from 2-(4-fluorophenyl)oxirane (69 mg, 0.5 mmol), KOH (2.8 mg, 0.05 mmol, 10 mol%) and **Ir6** (2.1 mg, 0.0025 mmol, 0.5 mol%) in 2-propanol (1 mL) at 95 ºC. Colorless oil. ^{1}H NMR ($CDCl_3$, 400 MHz): δ (ppm) 7.38-7.33 (m, 2H), 7.05 (t, J = 8.8 Hz, 2H), 4.90 (q, J = 6.4 Hz, 1H), 1.96 (bs, 1H), 1.50 (d, J = 6.4 Hz, 3H); ^{13}C{^{1}H} NMR ($CDCl_3$, 100 MHz): δ (ppm) 163.3, 160.9, 141.5, 141.5, 127.1, 127.0, 115.4, 115.3, 69.8, 25.3.

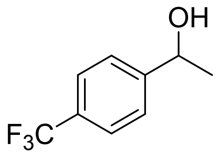

1-(4-(trifluoromethyl)phenyl)ethanol (4g).[27] Following the general procedure (GP2a), the title product **4g** (87 mg, 92% yield) was isolated by using silica gel column chromatography eluting with hexane and ethyl acetate (8:2) mixture, starting from 2-(4-(trifluoromethyl)phenyl)oxirane (94 mg, 0.5 mmol), KOH (2.8 mg, 0.05 mmol, 10 mol%) and **Ir6** (2.1 mg, 0.0025 mmol, 0.5 mol%) in 2-propanol (1 mL) at 95 ºC. Colorless oil. ^{1}H NMR ($CDCl_3$, 400 MHz): δ (ppm) 7.63 (d, J = 8.4 Hz, 2H), 7.51 (d, J = 8.4 Hz, 2H), 5.00 (q, J = 6.4 Hz, 1H), 1.84 (bs, 1H), 1.53 (d, J = 6.4 Hz, 3H); ^{13}C{^{1}H} NMR ($CDCl_3$, 100 MHz): δ (ppm) 149.7, 130.1, 129.7 (q, J = 32.2 Hz), 125.6, 125.4 (q, J = 4.4 Hz), 125.5 (q, J = 357.0 Hz), 69.8, 25.4.

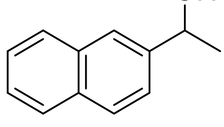

1-(naphthalen-2-yl)ethanol (4i).[27] Following the general procedure (GP2a), the title product **4i** (56 mg, 65% yield) was isolated by using silica gel column chromatography eluting with hexane and ethyl acetate (9:1) mixture, starting from 2-(naphthalen-2-yl)oxirane (85 mg, 0.5 mmol), KOH (2.8 mg, 0.05 mmol, 10 mol%) and **Ir6** (2.1 mg, 0.0025 mmol, 0.5 mol%) in 2-propanol (1 mL) at 95 ºC. White solid (m.p.: 72-74 ºC). ^{1}H NMR ($CDCl_3$, 400 MHz): δ (ppm) 7.87-7.83 (m, 4H), 7.54-7.49 (m, 3H), 5.09 (q, J = 6.4 Hz, 1H), 2.01 (bs, 1H), 1.61 (d, J = 6.4 Hz, 3H); $^{13}C\{^1H\}$ NMR ($CDCl_3$, 100 MHz): δ (ppm) 143.2, 133.3, 132.9, 128.3, 127.9, 127.6, 126.2, 125.8, 123.8, 123.8, 70.5, 25.2.

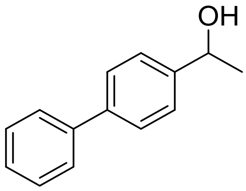

1-([1,1'-biphenyl]-4-yl)ethanol (4j).[28] Following the general procedure (GP2b), the title product **4j** (69 mg, 70% yield) was isolated by using silica gel column chromatography eluting with hexane and ethyl acetate (9:1) mixture, starting from 2-([1,1'-biphenyl]-4-yl)oxirane (99 mg, 0.5 mmol), KOH (5.6 mg, 0.1 mmol, 20 mol%) and **Ir6** (4.2 mg, 0.005 mmol, 1 mol%) in 2-propanol (1 mL) at 135 ºC. White solid (m.p.: 95-97 ºC). ^{1}H NMR ($CDCl_3$, 400 MHz): δ (ppm) 7.63-7.60 (m, 4H), 7.49-7.45 (m, 4H), 7.38 (t, J = 7.2 Hz, 1H), 4.99 (m, 1H), 1.90 (bs, 1H), 1.57 (d, J = 6.4 Hz, 3H); $^{13}C\{^1H\}$ NMR ($CDCl_3$, 100 MHz): δ (ppm) 144.8, 140.9, 140.5, 128.8, 127.3, 127.1, 125.9, 70.2, 25.2.

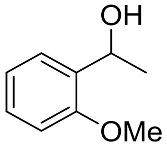

1-(2-methoxyphenyl)ethanol (4k).[29] Following the general procedure (GP2b), the title product **4k** (49 mg, 65% yield) was isolated by using silica gel column chromatography eluting with hexane and ethyl acetate (8:2) mixture, starting from 2-(2-methoxyphenyl)oxirane (75 mg, 0.5

mmol), KOH (5.6 mg, 0.1 mmol, 20 mol%) and **Ir6** (4.2 mg, 0.005 mmol, 1 mol%) in 2-propanol (1 mL) at 135 ºC. Colorless oil. ^{1}H NMR (CDCl$_3$, 400 MHz): δ (ppm) 7.35 (d, J = 7.6 Hz, 1H), 7.27-7.23 (m, 1H), 6.97 (t, J = 7.6 Hz, 1H), 6.88 (d, J = 8.0 Hz, 1H), 5.10 (m, 1H), 3.86 (s, 3H), 2.77 (bs, 1H), 1.51 (d, J = 6.4 Hz, 3H); ^{13}C{^{1}H} NMR (CDCl$_3$, 100 MHz): δ (ppm) 156.5, 133.5, 128.2, 126.1, 120.8, 110.4, 66.4, 55.2, 22.9.

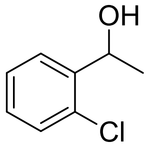

1-(2-chlorophenyl)ethanol (4l).[29] Following the general procedure (GP2b), the title product **4l** (39 mg, 51% yield) was isolated by using silica gel column chromatography eluting with hexane and ethyl acetate (9:1) mixture, starting from 2-(2-chlorophenyl)oxirane (77 mg, 0.5 mmol), KOH (5.6 mg, 0.1 mmol, 20 mol%) and **Ir6** (4.2 mg, 0.005 mmol, 1 mol%) in 2-propanol (1 mL) at 135 ºC. Colorless oil. ^{1}H NMR (CDCl$_3$, 400 MHz): δ (ppm) 7.61 (d, J = 7.6 Hz, 1H), 7.36-7.28 (m, 2H), 7.22 (t, J = 7.6 Hz, 1H), 5.31 (q, J = 6.4 Hz, 1H), 1.99 (bs, 1H), 1.52 (d, J = 6.4 Hz, 3H); ^{13}C{^{1}H} NMR (CDCl$_3$, 100 MHz): δ (ppm) 143.0, 131.6, 129.4, 128.4, 127.2, 126.4, 66.9, 23.5.

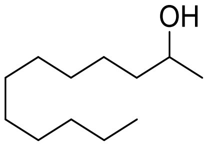

dodecan-2-ol (4n).[24,25] Following the general procedure (GP2a), the title product **4n** (83 mg, 89% yield) was isolated by using silica gel column chromatography eluting with hexane and ethyl acetate (95:5) mixture, starting from 2-decyloxirane (92 mg, 0.5 mmol), KOH (2.8 mg, 0.05 mmol, 10 mol%) and **Ir6** (2.1 mg, 0.0025 mmol, 0.5 mol%) in 2-propanol (1 mL) at 95 ºC. Colorless oil. ^{1}H NMR (CDCl$_3$, 400 MHz): δ (ppm) 3.85-3.77 (m, 1H), 1.50-1.23 (m, 18H),

1.20 (d, J = 6.4 Hz, 3H), 0.89 (t, J = 6.4 Hz, 3H); $^{13}C\{^1H\}$ NMR ($CDCl_3$, 100 MHz): δ (ppm) 68.2, 39.4, 31.9, 29.7, 29.6, 29.3, 25.8, 23.5, 22.7, 14.1.

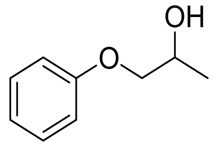

1-phenoxypropan-2-ol (4o).[25] Following the general procedure (GP2a), the title product **4o** (71 mg, 94% yield) was isolated by using silica gel column chromatography eluting with hexane and ethyl acetate (8:2) mixture, starting from 2-(phenoxymethyl)oxirane (75 mg, 0.5 mmol), KOH (2.8 mg, 0.05 mmol, 10 mol%) and **Ir6** (2.1 mg, 0.0025 mmol, 0.5 mol%) in 2-propanol (1 mL) at 95 ºC. Colorless oil. ^1H NMR ($CDCl_3$, 400 MHz): δ (ppm) 7.35-7.32 (m, 2H), 7.00 (t, J = 7.6 Hz, 1H), 6.94 (d, J = 7.6 Hz, 2H), 4.27-4.19 (m, 1H), 3.97 (dd, J_1 = 9.4 Hz, J_2 = 3.2 Hz, 1H), 3.82 (dd, J_1 = 9.2 Hz, J_2 = 7.6 Hz, 1H), 3.02 (bs, 1H), 1.32 (d, J = 6.4 Hz, 3H); $^{13}C\{^1H\}$ NMR ($CDCl_3$, 100 MHz): δ (ppm) 158.5, 129.5, 121.2, 114.6, 73.2, 66.3, 18.7.

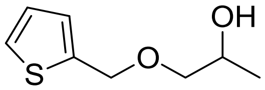

1-(thiophen-2-ylmethoxy)propan-2-ol (4p). Following the general procedure (GP2a), the title product **4p** (83 mg, 96% yield) was isolated by using silica gel column chromatography eluting with hexane and ethyl acetate (8:2) mixture, starting from 2-((thiophen-2-ylmethoxy)methyl)oxirane (85 mg, 0.5 mmol), KOH (2.8 mg, 0.05 mmol, 10 mol%) and **Ir6** (2.1 mg, 0.0025 mmol, 0.5 mol%) in 2-propanol (1 mL) at 95 ºC. Colorless oil. ^1H NMR ($CDCl_3$, 400 MHz): δ (ppm) 7.30 (d, J = 5.2 Hz, 1H), 7.01-6.97 (m, 2H), 4.72 (s, 2H), 4.01-3.94 (m, 1H), 3.48 (dd, J_1 = 9.6 Hz, J_2 = 3.2 Hz, 1H), 3.28 (dd, J_1 = 9.6 Hz, J_2 = 8.4 Hz, 1H), 2.36 (bs, 1H), 1.14 (d, J = 6.4 Hz, 3H); $^{13}C\{^1H\}$ NMR ($CDCl_3$, 100 MHz): δ (ppm) 140.7, 126.7, 126.5, 125.9, 75.4, 67.7, 66.4, 18.6. HRMS (ESI): m/z: $[M]^+$ calcd for $C_8H_{12}O_2S$ 172.0558; found: 172.0563, Δ = + 3.1 ppm.

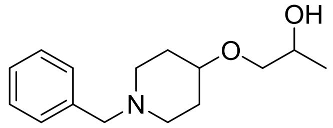

1-((1-benzylpiperidin-4-yl)oxy)propan-2-ol (4q). Following the general procedure (GP2a), the title product **4q** (108 mg, 87% yield) was isolated by using silica gel column chromatography eluting with hexane and ethyl acetate (5:5) mixture, starting from 1-benzyl-4-(oxiran-2-ylmethoxy)piperidine (123 mg, 0.5 mmol), KOH (2.8 mg, 0.05 mmol, 10 mol%) and **Ir6** (2.1 mg, 0.0025 mmol, 0.5 mol%) in 2-propanol (1 mL) at 95 ºC. Colorless oil. ^{1}H NMR ($CDCl_3$, 400 MHz): δ (ppm) 7.31-7.22 (m, 5H), 3.95-3.87 (m, 1H), 3.51 (s, 2H), 3.42 (dd, J_1 = 9.2 Hz, J_2 = 3.2 Hz, 1H), 3.38-3.32 (m, 1H), 3.20 (t, J = 8.0 Hz, 1H), 2.87 (bs, 1H), 2.74-2.71 (m, 2H), 2.18 (t, J = 9.6 Hz, 2H), 1.91-1.87 (m, 2H), 1.66-1.59 (m, 2H), 1.13 (d, J = 6.4 Hz, 3H); ^{13}C{^{1}H} NMR ($CDCl_3$, 100 MHz): δ (ppm) 137.8, 129.2, 128.2, 127.1, 73.4, 66.5, 62.9, 50.8, 31.1, 30.9, 18.7. HRMS (ESI): m/z: $[M]^+$ calcd for $C_{15}H_{23}NO_2$ 249.1729; found: 249.1727, Δ = – 0.7 ppm.

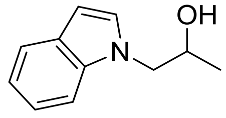

1-(1*H*-indol-1-yl)propan-2-ol (4r).[30] Following the general procedure (GP2a), the title product **4r** (71 mg, 81% yield) was isolated by using silica gel column chromatography eluting with hexane and ethyl acetate (7:3) mixture, starting from 1-(oxiran-2-ylmethyl)-1H-indole (86 mg, 0.5 mmol), KOH (2.8 mg, 0.05 mmol, 10 mol%) and **Ir6** (2.1 mg, 0.0025 mmol, 0.5 mol%) in 2-propanol (1 mL) at 95 ºC. Colorless oil. ^{1}H NMR ($CDCl_3$, 400 MHz): δ (ppm) 7.64 (d, J = 8.0 Hz, 1H), 7.38 (d, J = 8.0 Hz, 1H), 7.24-7.20 (m, 1H), 7.14-7.11 (m, 2H), 6.53 (d, J = 3.2 Hz, 1H), 4.22-4.13 (m, 2H), 4.05-3.99 (m, 1H), 1.73 (bs, 1H), 1.25 (d, J = 5.6 Hz, 3H); ^{13}C{^{1}H} NMR ($CDCl_3$, 100 MHz): δ (ppm) 136.3, 128.6, 128.5, 121.7, 121.0, 119.5, 109.5, 101.6, 67.3, 53.8, 20.5.

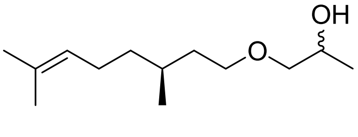

1-((3,7-dimethyloct-6-en-1-yl)oxy)propan-2-ol (4s). Following the general procedure (GP2a), the title product **4s** (98 mg, 92% yield) was isolated by using silica gel column chromatography eluting with hexane and ethyl acetate (8:2) mixture, starting from 2-((((S)-3,7-dimethyloct-6-en-1-yl)oxy)methyl)oxirane (106 mg, 0.5 mmol), KOH (2.8 mg, 0.05 mmol, 10 mol%) and **Ir6** (2.1 mg, 0.0025 mmol, 0.5 mol%) in 2-propanol (1 mL) at 95 ºC. Colorless oil. Mixture of diastereoisomers (61:39). ^{1}H NMR ($CDCl_3$, 400 MHz): δ (ppm) 5.06 (d, J = 8.0 Hz, 1H), 3.96-3.88 (m, 1H), 3.53-3.42 (m, 2H), 3.39-3.35 (m, 1H), 3.19 (dd, J_1 = 8.0 Hz, J_2 = 4.8 Hz, 1H), 2.53 (bs, 1H), 2.01-1.89 (m, 2H), 1.65 (s, 3H), 1.63-1.50 (m, 6H), 1.42-1.27 (m, 2H), 1.11 (d, J = 6.4 Hz, 3H), 0.87 (d, J = 6.4 Hz, 3H); ^{13}C{^{1}H} NMR ($CDCl_3$, 100 MHz): δ (ppm) 131.1, 124.7, 76.3, 71.9, 69.6, 69.3, 66.3, 39.2, 37.2, 36.5, 29.8, 29.5, 27.9, 25.7, 25.4, 24.6, 22.7, 22.6, 21.9 19.6, 19.5, 18.6, 17.6. HRMS (ESI): m/z: $[M]^+$ calcd for $C_{13}H_{26}O_2$ 214.1933; found: 214.1931, Δ = – 0.8 ppm.

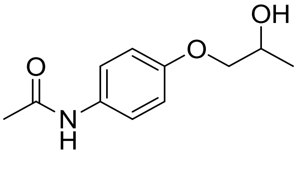

N-(4-(2-hydroxypropoxy)phenyl)acetamide (4u).[31] Following the general procedure (GP2d), the title product **4u** (98 mg, 94% yield) was isolated by using silica gel column chromatography eluting with dichloromethane and methanol (8:2) mixture, starting from N-(4-(oxiran-2-ylmethoxy)phenyl)acetamide (103 mg, 0.5 mmol), Na_2CO_3 (5.3 mg, 0.05 mmol; 10 mol%), 10% **Pd/C** complex (10.6 mg, 0.01 mmol, 2 mol%) and **Ir6** (2.1 mg, 0.0025 mmol, 0.5 mol%) in 2-propanol (1 mL) at 95 ºC. White solid (m.p.: 132-134 ºC). ^{1}H NMR (DMSO-d_6, 400 MHz): δ (ppm) 9.73 (bs, 1H-NH), 7.44 (d, J = 9.2 Hz, 2H), 6.83 (d, J = 9.2 Hz, 2H), 4.81 (d, J = 4.8 Hz, 1H), 3.93-3.87 (m, 1H), 3.76 (dd, J_1 = 9.6 Hz, J_2 = 3.2 Hz, 1H), 1.98 (s, 3H), 1.12 (d, J =

6.4 Hz, 3H); $^{13}C\{^1H\}$ NMR (DMSO-d_6, 100 MHz): δ (ppm) 194.4, 168.1, 154.9, 132.9, 120.9, 114.9, 73.8, 64.9, 24.2, 20.6.

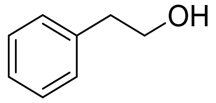

2-phenylethanol (5a).[5,6] Following the general procedure (GP2c), the title product **5a** (56 mg, 92% yield) was isolated by using silica gel column chromatography eluting with hexane and ethyl acetate (8:2) mixture, starting from 2-phenyloxirane (60 mg, 0.5 mmol), Cs_2CO_3 (16.3 mg, 0.05 mmol; 10 mol%), 10% **Pd/C** (10.6 mg, 0.01 mmol, 2 mol%) and **Ir6** (2.1 mg, 0.0025 mmol, 0.5 mol%) in 2-propanol (2 mL) at 95 ºC. Colorless oil. ^1H NMR ($CDCl_3$, 400 MHz): δ (ppm) 7.37-7.33 (m, 2H), 7.28-7.25 (m, 3H), 3.88 (t, J = 6.8 Hz, 2H), 2.89 (t, J = 6.8 Hz, 2H), 1.66 (bs, 1H); $^{13}C\{^1H\}$ NMR ($CDCl_3$, 100 MHz): δ (ppm) 138.5, 129.0, 128.6, 126.5, 63.7, 39.2.

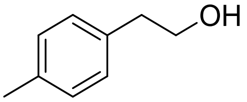

2-(p-tolyl)ethanol (5b).[32] Following the general procedure (GP2c), the title product **5b** (65 mg, 95% yield) was isolated by using silica gel column chromatography eluting with hexane and ethyl acetate (8:2) mixture, starting from 2-(p-tolyl)oxirane (67 mg, 0.5 mmol), Cs_2CO_3 (16.3 mg, 0.05 mmol; 10 mol%), 10% **Pd/C** (10.6 mg, 0.01 mmol, 2 mol%) and **Ir6** (2.1 mg, 0.0025 mmol, 0.5 mol%) in 2-propanol (2 mL) at 95 ºC. Colorless oil. ^1H NMR ($CDCl_3$, 400 MHz): δ (ppm) 7.13 (s, 4H), 3.83 (t, J = 6.4 Hz, 2H), 2.83 (t, J = 6.8 Hz, 2H), 2.34 (s, 3H), 1.63 (bs, 1H); $^{13}C\{^1H\}$ NMR ($CDCl_3$, 100 MHz): δ (ppm) 136.0, 135.3, 129.3, 128.9, 63.8, 38.7, 21.0.

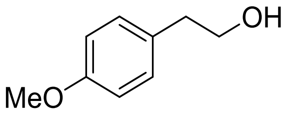

2-(4-methoxyphenyl)ethanol (5c).[32] Following the general procedure (GP2c), the title product **5c** (71 mg, 94% yield) was isolated by using silica gel column chromatography eluting with hexane and ethyl acetate (7:3) mixture, starting from 2-(4-methoxyphenyl)oxirane (75 mg, 0.5 mmol), Cs_2CO_3 (16.3 mg, 0.05 mmol; 10 mol%), 10% **Pd/C** (10.6 mg, 0.01 mmol, 2 mol%) and **Ir6** (2.1 mg, 0.0025 mmol, 0.5 mol%) in 2-propanol (2 mL) at 95 ºC. Colorless oil. ^{1}H NMR ($CDCl_3$, 400 MHz): δ (ppm) 7.15 (d, J = 8.4 Hz, 2H), 6.86 (d, J = 8.4 Hz, 2H), 3.83 (t, J = 6.4 Hz, 2H), 2.82 (t, J = 6.4 Hz, 2H); $^{13}C\{^1H\}$ NMR ($CDCl_3$, 100 MHz): δ (ppm) 158.3, 130.4, 129.9, 114.0, 63.8, 55.3, 38.3.

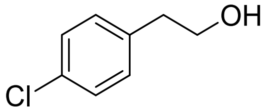

2-(4-chlorophenyl)ethanol (5d).[5] Following the general procedure (GP2c), the title product **5d** (62 mg, 80% yield) was isolated by using silica gel column chromatography eluting with hexane and ethyl acetate (8:2) mixture, starting from 2-(4-chlorophenyl)oxirane (77 mg, 0.5 mmol), Cs_2CO_3 (16.3 mg, 0.05 mmol; 10 mol%), 10% **Pd/C** (10.6 mg, 0.01 mmol, 2 mol%) and **Ir6** (2.1 mg, 0.0025 mmol, 0.5 mol%) in 2-propanol (2 mL) at 95 ºC. Colorless oil. ^{1}H NMR ($CDCl_3$, 400 MHz): δ (ppm) 7.30 (d, J = 8.4 Hz, 2H), 7.18 (d, J = 8.4 Hz, 2H), 3.85 (t, J = 6.8 Hz, 2H), 2.85 (t, J = 6.8 Hz, 2H), 1.66 (bs, 1H); $^{13}C\{^1H\}$ NMR ($CDCl_3$, 100 MHz): δ (ppm) 137.0, 132.3, 130.4, 128.7, 63.4, 38.5.

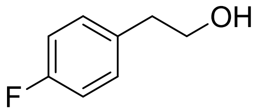

2-(4-fluorophenyl)ethanol (5e).[5,32] Following the general procedure (GP2c), the title product **5e** (53 mg, 75% yield) was isolated by using silica gel column chromatography eluting with

hexane and ethyl acetate (8:2) mixture, starting from 2-(4-fluorophenyl)oxirane (69 mg, 0.5 mmol), Cs_2CO_3 (16.3 mg, 0.05 mmol; 10 mol%), 10% **Pd/C** (10.6 mg, 0.01 mmol, 2 mol%) and **Ir6** (2.1 mg, 0.0025 mmol, 0.5 mol%) in 2-propanol (2 mL) at 95 ºC. Colorless oil. ^1H NMR ($CDCl_3$, 400 MHz): δ (ppm) 7.22-7.17 (m, 2H), 7.00 (t, J = 8.8 Hz, 2H), 3.85 (t, J = 6.8 Hz, 2H), 2.85 (t, J = 6.8 Hz, 2H), 1.57 (bs, 1H); $^{13}C\{^1H\}$ NMR ($CDCl_3$, 100 MHz): δ (ppm) 161.6 (d, J_{C-F} = 245.2 Hz), 161.6 (d, J_{C-F} = 245.2 Hz), 134.1 (d, J_{C-F} = 3.2 Hz), 130.4 (d, J_{C-F} = 7.8 Hz), 115.3 (d, J_{C-F} = 21.2 Hz), 63.6, 38.6. ^{19}F NMR (376 MHz, $CDCl_3$, TMS, 25ºC, ppm): δ = -116.8

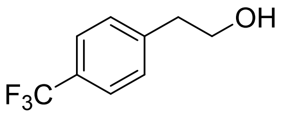

2-(4-(trifluoromethyl)phenyl)ethanol (5f).[5] Following the general procedure (GP2c), the title product **5f** (76 mg, 80% yield) was isolated by using silica gel column chromatography eluting with hexane and ethyl acetate (8:2) mixture, starting from 2-(4-(trifluoromethyl)phenyl)oxirane (94 mg, 0.5 mmol), Cs_2CO_3 (16.3 mg, 0.05 mmol; 10 mol%), 10% **Pd/C** (10.6 mg, 0.01 mmol, 2 mol%) and **Ir6** (2.1 mg, 0.0025 mmol, 0.5 mol%) in 2-propanol (2 mL) at 95 ºC. Colorless oil. ^1H NMR ($CDCl_3$, 400 MHz): δ (ppm) 7.56 (d, J = 8.4 Hz, 2H), 7.34 (d, J = 8.8 Hz, 2H), 3.87 (t, J = 6.4 Hz, 2H), 2.91 (t, J = 6.4 Hz, 2H), 2.08 (bs, 1H); $^{13}C\{^1H\}$ NMR ($CDCl_3$, 100 MHz): δ (ppm) 142.8, 129.3, 128.8 (q, J = 32.5 Hz), 125.4 (q, J = 3.8 Hz), 124.2 (q, J = 272.7 Hz), 63.2, 38.9. ^{19}F NMR (376 MHz, $CDCl_3$, TMS, 25ºC, ppm): δ = -62.5

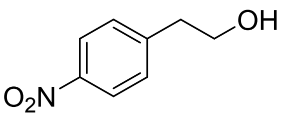

2-(4-nitrophenyl)ethanol (5g).[6] Following the general procedure (GP2c), the title product **5g** (63 mg, 75% yield) was isolated by using silica gel column chromatography eluting with hexane and ethyl acetate (9:1) mixture, starting from 2-(4-nitrophenyl)oxirane (82 mg, 0.5 mmol),

Cs_2CO_3 (16.3 mg, 0.05 mmol; 10 mol%), 10% **Pd/C** (10.6 mg, 0.01 mmol, 2 mol%) and **Ir6** (2.1 mg, 0.0025 mmol, 0.5 mol%) in 2-propanol (2 mL) at 95 ºC. Orange solid (m.p.: 60-62 ºC). ^{1}H NMR ($CDCl_3$, 400 MHz): δ (ppm) 8.16 (d, J = 8.4 Hz, 2H), 7.40 (d, J = 8.4 Hz, 2H), 3.92 (t, J = 6.4 Hz, 2H), 2.97 (t, J = 6.4 Hz, 2H); $^{13}C\{^1H\}$ NMR ($CDCl_3$, 100 MHz): δ (ppm) 146.7, 129.8, 123.7, 62.9, 38.9.

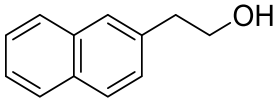

2-(naphthalen-2-yl)ethanol (5h).[5] Following the general procedure (GP2c), the title product **5h** (68 mg, 79% yield) was isolated by using silica gel column chromatography eluting with hexane and ethyl acetate (8:2) mixture, starting from 2-(naphthalen-2-yl)oxirane (85 mg, 0.5 mmol), Cs_2CO_3 (16.3 mg, 0.05 mmol; 10 mol%), 10% **Pd/C** (10.6 mg, 0.01 mmol, 2 mol%) and **Ir6** (2.1 mg, 0.0025 mmol, 0.5 mol%) in 2-propanol (2 mL) at 95 ºC. White solid (m.p.: 63-65 ºC). ^{1}H NMR ($CDCl_3$, 400 MHz): δ (ppm) 7.84-7.80 (m, 3H), 7.69 (s, 1H), 7.51-7.44 (m, 2H), 7.36 (d, J = 8.4 Hz, 1H), 3.93 (t, J = 6.8 Hz, 2H), 3.03 (t, J = 6.8 Hz, 2H), 1.75 (bs, 1H); $^{13}C\{^1H\}$ NMR ($CDCl_3$, 100 MHz): δ (ppm) 135.9, 133.6, 132.3, 128.2, 127.7, 127.5, 127.5, 127.4, 63.5, 39.3.

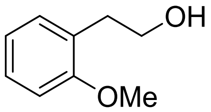

2-(2-methoxyphenyl)ethanol (5i).[33] Following the general procedure (GP2c), the title product **5i** (64 mg, 84% yield) was isolated by using silica gel column chromatography eluting with hexane and ethyl acetate (7:3) mixture, starting from 2-(2-methoxyphenyl)oxirane (75 mg, 0.5 mmol), Cs_2CO_3 (16.3 mg, 0.05 mmol; 10 mol%), 10% **Pd/C** (10.6 mg, 0.01 mmol, 2 mol%) and **Ir6** (2.1 mg, 0.0025 mmol, 0.5 mol%) in 2-propanol (2 mL) at 95 ºC. Colorless oil. ^{1}H NMR ($CDCl_3$, 400 MHz): δ (ppm) 7.24-7.16 (m, 2H), 6.93-6.86 (m, 2H), 3.84-3.81 (m, 5H),

2.91 (t, J = 6.4 Hz, 2H), 1.81 (bs, 1H); $^{13}C\{^1H\}$ NMR ($CDCl_3$, 100 MHz): δ (ppm) 157.6, 130.9, 127.8, 127.0, 120.6, 110.4, 62.8, 55.3, 34.1.

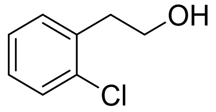

2-(2-chlorophenyl)ethanol (5j).[32] Following the general procedure (GP2c), the title product **5j** (48 mg, 61% yield) was isolated by using silica gel column chromatography eluting with hexane and ethyl acetate (8:2) mixture, starting from 2-(2-chlorophenyl)oxirane (77 mg, 0.5 mmol), Cs_2CO_3 (16.3 mg, 0.05 mmol; 10 mol%), 10% **Pd/C** (10.6 mg, 0.01 mmol, 2 mol%) and **Ir6** (2.1 mg, 0.0025 mmol, 0.5 mol%) in 2-propanol (2 mL) at 95 ºC. Colorless oil. ^1H NMR ($CDCl_3$, 400 MHz): δ (ppm) 7.38-7.36 (m, 1H), 7.29-7.26 (m, 1H), 7.23-7.16 (m, 2H), 3.89 (q, J = 6.8 Hz, 2H), 3.03 (t, J = 6.8 Hz, 2H), 1.37 (bs, 1H); $^{13}C\{^1H\}$ NMR ($CDCl_3$, 100 MHz): δ (ppm) 136.1, 134.2, 131.3, 129.6, 127.9, 126.8, 62.0, 36.9.

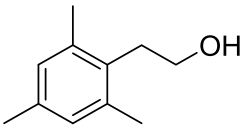

2-mesitylethanol (5k).[34] Following the general procedure (GP2c), the title product **5k** (62 mg, 75% yield) was isolated by using silica gel column chromatography eluting with hexane and ethyl acetate (8:2) mixture, starting from 2-mesityloxirane (81 mg, 0.5 mmol), Cs_2CO_3 (16.3 mg, 0.05 mmol; 10 mol%) 10% **Pd/C** (10.6 mg, 0.01 mmol, 2 mol%) and **Ir6** (2.1 mg, 0.0025 mmol, 0.5 mol%) in 2-propanol (2 mL) at 95 ºC. White solid (m.p.: 76-78 ºC). ^1H NMR ($CDCl_3$, 400 MHz): δ (ppm) 6.85 (s, 2H), 3.75 (t, J = 7.6 Hz, 2H), 2.93 (t, J = 7.6 Hz, 2H), 1.26 (bs, 1H); $^{13}C\{^1H\}$ NMR ($CDCl_3$, 100 MHz): δ (ppm) 136.8, 135.7, 131.4, 129.0, 61.8, 32.6, 20.8, 19.9.

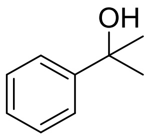

2-phenylpropan-2-ol (6a).[24,25] Following the general procedure (GP2b), the title product **6a** (51 mg, 75% yield) was isolated by using silica gel column chromatography eluting with hexane and ethyl acetate (9:1) mixture, starting from 2-methyl-2-phenyloxirane (67 mg, 0.5 mmol), KOH (5.6 mg, 0.1 mmol, 20 mol%) and **Ir6** (4.2 mg, 0.005 mmol, 1 mol%) in 2-propanol (1 mL) at 135 ºC. Colorless oil. ^{1}H NMR ($CDCl_3$, 400 MHz): δ (ppm) 7.54-7.51 (m, 2H), 7.38 (t, J = 7.2 Hz, 2H), 7.29-7.26 (m, 1H), 1.84 (bs, 1H), 1.62 (s, 6H); ^{13}C{^{1}H} NMR ($CDCl_3$, 100 MHz): δ (ppm) 149.1, 128.2, 126.7, 124.4, 72.5, 31.7.

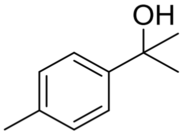

2-(p-tolyl)propan-2-ol (6b).[35] Following the general procedure (GP2b), the title product **6b** (68 mg, 91% yield) was isolated by using silica gel column chromatography eluting with hexane and ethyl acetate (9:1) mixture, starting from 2-methyl-2-(p-tolyl)oxirane (74 mg, 0.5 mmol), KOH (5.6 mg, 0.1 mmol, 20 mol%) and **Ir6** (4.2 mg, 0.005 mmol, 1 mol%) in 2-propanol (1 mL) at 135 ºC. Colorless oil. ^{1}H NMR ($CDCl_3$, 400 MHz): δ (ppm) 7.39 (d, J = 8.0 Hz, 2H), 7.39 (d, J = 8.0 Hz, 2H), 7.16 (d, J = 8.0 Hz, 2H), 2.35 (s, 3H), 1.58 (s, 6H); ^{13}C{^{1}H} NMR ($CDCl_3$, 100 MHz): δ (ppm) 146.2, 136.2, 128.9, 124.3, 72.4, 31.7, 20.9.

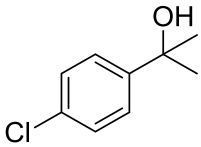

2-(4-chlorophenyl)propan-2-ol (6c).[35] Following the general procedure (GP2b), the title product **6c** (82 mg, 97% yield) was isolated by using silica gel column chromatography eluting with hexane and ethyl acetate (9:1) mixture, starting from 2-(4-chlorophenyl)-2-methyloxirane

(84 mg, 0.5 mmol), KOH (5.6 mg, 0.1 mmol, 20 mol%) and **Ir6** (4.2 mg, 0.005 mmol, 1 mol%) in 2-propanol (1 mL) at 135 ºC. Colorless oil. White solid (m.p.: 43-45 ºC). ^{1}H NMR (CDCl$_3$, 400 MHz): δ (ppm) 7.42 (d, *J* = 8.8 Hz, 2H), 7.29 (d, *J* = 8.8 Hz, 2H), 1.77 (bs, 1H), 1.56 (s, 6H); ^{13}C{^{1}H} NMR (CDCl$_3$, 100 MHz): δ (ppm) 147.6, 132.4, 128.2, 125.9, 72.2, 31.8.

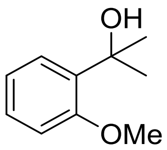

2-(2-methoxyphenyl)propan-2-ol (6d).[36] Following the general procedure (GP2b), the title product **6d** (34 mg, 41% yield) was isolated by using silica gel column chromatography eluting with hexane and ethyl acetate (9:1) mixture, starting from 2-(2-methoxyphenyl)-2-methyloxirane (82 mg, 0.5 mmol), KOH (5.6 mg, 0.1 mmol, 20 mol%) and **Ir6** (4.2 mg, 0.005 mmol, 1 mol%) in 2-propanol (1 mL) at 135 ºC. Colorless oil. ^{1}H NMR (CDCl$_3$, 400 MHz): δ (ppm) 7.33-7.31 (m, 1H), 7.26-7.22 (m, 1H), 6.97-6.92 (m, 2H), 4.20 (bs, 1H), 3.92 (s, 3H), 1.61 (s, 6H); ^{13}C{^{1}H} NMR (CDCl$_3$, 100 MHz): δ (ppm) 156.9, 135.8, 128.1, 125.8, 120.9, 111.3, 72.6, 55.3, 29.7.

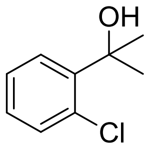

2-(2-chlorophenyl)propan-2-ol (6e).[37] Following the general procedure (GP2b), the title product **6e** (31 mg, 37% yield) was isolated by using silica gel column chromatography eluting with hexane and ethyl acetate (9:1) mixture, starting from 2-(2-chlorophenyl)-2-methyloxirane (84 mg, 0.5 mmol), KOH (5.6 mg, 0.1 mmol, 20 mol%) and **Ir6** (4.2 mg, 0.005 mmol, 1 mol%) in 2-propanol (1 mL) at 135 ºC. Colorless oil. ^{1}H NMR (CDCl$_3$, 400 MHz): δ (ppm) 7.66 (d, *J* = 8.0 Hz, 1H), 7.36 (d, *J* = 8.0 Hz, 1H), 7.27-7.17 (m, 2H), 2.68 (bs, 1H), 1.73 (s, 6H); ^{13}C{^{1}H} NMR (CDCl$_3$, 100 MHz): δ (ppm) 144.7, 131.4, 131.3, 128.3, 127.0, 126.9, 73.1, 29.4.

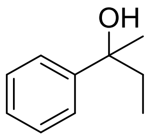

2-phenylbutan-2-ol (6f).[38] Following the general procedure (GP2b), the title product **6f** (71 mg, 94% yield) was isolated by using silica gel column chromatography eluting with hexane and ethyl acetate (9:1) mixture, starting from 2-ethyl-2-phenyloxirane (74 mg, 0.5 mmol), KOH (5.6 mg, 0.1 mmol, 20 mol%) and **Ir6** (4.2 mg, 0.005 mmol, 1 mol%) in 2-propanol (1 mL) at 135 ºC. Colorless oil. ^{1}H NMR ($CDCl_3$, 400 MHz): δ (ppm) 7.45-7.43 (m, 2H), 7.35 (t, *J* = 7.2 Hz, 2H), 7.24 (t, *J* = 7.6 Hz, 1H), 1.88-1.79 (m, 2H - bs, 1H), 1.56 (s, 3H), 0,80 (t, *J* = 7.2 Hz, 3H); $^{13}C\{^1H\}$ NMR ($CDCl_3$, 100 MHz): δ (ppm) 147.7, 128.1, 126.5, 124.9, 74.9, 36.6, 29.6, 8.3.

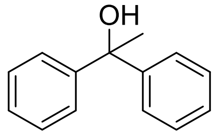

1,1-diphenylethanol (6g).[24] Following the general procedure (GP2b), the title product **6g** (95 mg, 96% yield) was isolated by using silica gel column chromatography eluting with hexane and ethyl acetate (9:1) mixture, starting from 2,2-diphenyloxirane (98 mg, 0.5 mmol), KOH (5.6 mg, 0.1 mmol, 20 mol%) and **Ir6** (4.2 mg, 0.005 mmol, 1 mol%) in 2-propanol (1 mL) at 135 ºC. White solid (m.p.: 80-82 ºC). ^{1}H NMR ($CDCl_3$, 400 MHz): δ (ppm) 7.44-7.41 (m, 4H), 7.35-7.31 (m, 4H), 7.27-7.23 (m, 2H), 2.24 (bs, 1H), 1.96 (s, 3H); $^{13}C\{^1H\}$ NMR ($CDCl_3$, 100 MHz): δ (ppm) 147.9, 128.2, 126.9, 125.8, 76.2, 30.8.

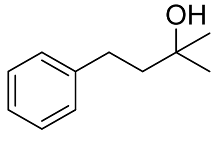

2-methyl-4-phenylbutan-2-ol (6h).[39] Following the general procedure (GP2b), the title product **6h** (77 mg, 94% yield) was isolated by using silica gel column chromatography eluting

with hexane and ethyl acetate (9:1) mixture, starting from 2-methyl-2-phenethyloxirane (81 mg, 0.5 mmol), KOH (5.6 mg, 0.1 mmol, 20 mol%) and **Ir6** (4.2 mg, 0.005 mmol, 1 mol%) in 2-propanol (1 mL) at 135 °C. Colorless oil. ^{1}H NMR ($CDCl_3$, 400 MHz): δ (ppm) 7.31-7.16 (m, 5H), 2.73-7.68 (m, 2H), 1.82-1.76 (m, 2H), 1.30 (s, 6H); $^{13}C\{^1H\}$ NMR ($CDCl_3$, 100 MHz): δ (ppm) 142.5, 128.4, 128.3, 125.7, 70.9, 45.7, 30.7, 29.3.

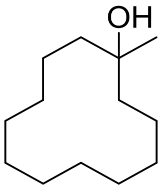

1-methylcyclododecanol (6i).[24] Following the general procedure (GP2b), the title product **6h** (48 mg, 49% yield) was isolated by using silica gel column chromatography eluting with hexane and ethyl acetate (95:5) mixture, starting from 1-oxaspiro[2.11]tetradecane (98 mg, 0.5 mmol), KOH (5.6 mg, 0.1 mmol, 20 mol%) and **Ir6** (4.2 mg, 0.005 mmol, 1 mol%) in 2-propanol (1 mL) at 135 °C. White solid (m.p.: 84-86 °C). ^{1}H NMR ($CDCl_3$, 400 MHz): δ (ppm) 1.58-1.52 (m, 2H), 1.45-1.35 (m, 20H), 1.25 (bs, 1H), 1.17 (s, 3H); $^{13}C\{^1H\}$ NMR ($CDCl_3$, 100 MHz): δ (ppm) 73.7, 36.2, 29.0, 26.4, 26.0, 22.5, 22.1, 19.9.

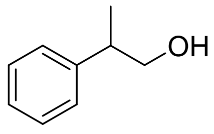

2-phenylpropan-1-ol (7a).[5,24] Following the general procedure (GP2e), the title product **7a** (47 mg, 70% yield) was isolated by using silica gel column chromatography eluting with hexane and ethyl acetate (9:1) mixture, starting from 2-methyl-2-phenyloxirane (67 mg, 0.5 mmol), Cs_2CO_3 (16.3 mg, 0.05 mmol; 10 mol%), 10% **Pd/C** (15.9 mg, 0.015 mmol, 3 mol%) and **Ir6** (2.1 mg, 0.0025 mmol, 0.5 mol%) in 2-propanol (2 mL) at 95 °C. Colorless oil. ^{1}H NMR ($CDCl_3$, 400 MHz): δ (ppm) 7.38-7.25 (m, 5H), 3.72 (d, J = 6.8 Hz, 2H), 3.02-2.93 (m, 1H),

1.56 (bs, 1H), 1.31 (d, J = 7.2 Hz, 3H); $^{13}C\{^1H\}$ NMR ($CDCl_3$, 100 MHz): δ (ppm) 143.7, 128.6, 127.5, 126.7, 68.7, 42.5, 17.6.

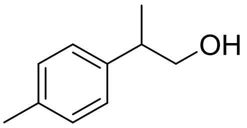

2-(p-tolyl)propan-1-ol (7b).[40] Following the general procedure (GP2e), the title product **7b** (57 mg, 76% yield) was isolated by using silica gel column chromatography eluting with hexane and ethyl acetate (9:1) mixture, starting from 2-methyl-2-(p-tolyl)oxirane (74 mg, 0.5 mmol), Cs_2CO_3 (16.3 mg, 0.05 mmol; 10 mol%), 10% **Pd/C** (15.9 mg, 0.015 mmol, 3 mol%) and **Ir6** (2.1 mg, 0.0025 mmol, 0.5 mol%) in 2-propanol (2 mL) at 95 ºC. Colorless oil. ^1H NMR ($CDCl_3$, 400 MHz): δ (ppm) 7.18-7.14 (m, 4H), 3.67 (d, J = 7.2 Hz, 2H), 2.96-2.88 (m, 1H), 2.35 (s, 3H), 1.27 (d, J = 6.8 Hz, 3H); $^{13}C\{^1H\}$ NMR ($CDCl_3$, 100 MHz): δ (ppm) 140.6, 136.0, 129.2, 127.2, 68.5, 41.9, 20.9, 17.6.

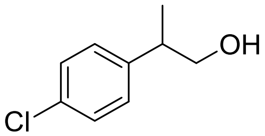

2-(4-chlorophenyl)propan-1-ol (7c).[40] Following the general procedure (GP2e), the title product **7c** (42 mg, 50% yield) was isolated by using silica gel column chromatography eluting with hexane and ethyl acetate (9:1) mixture, starting from 2-(4-chlorophenyl)-2-methyloxirane (84 mg, 0.5 mmol), Cs_2CO_3 (16.3 mg, 0.05 mmol; 10 mol%), 10% **Pd/C** (15.9 mg, 0.015 mmol, 3 mol%) and **Ir6** (2.1 mg, 0.0025 mmol, 0.5 mol%) in 2-propanol (2 mL) at 95 ºC. Colorless oil. ^1H NMR ($CDCl_3$, 400 MHz): δ (ppm) 7.29 (d, J = 8.4 Hz, 2H), 7.17 (d, J = 8.0 Hz, 2H), 3.67 (d, J = 6.8 Hz, 2H), 2.97-2.88 (m, 1H), 1.43 (bs, 1H), 1.25 (d, J = 6.8 Hz, 3H); $^{13}C\{^1H\}$ NMR ($CDCl_3$, 100 MHz): δ (ppm) 142.2, 132.3, 128.8, 128.7, 68.5, 41.8, 17.5.

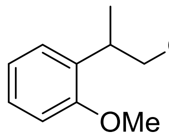

2-(2-methoxyphenyl)propan-1-ol (7d).[41] Following the general procedure (GP2e), the title product **7d** (73 mg, 89% yield) was isolated by using silica gel column chromatography eluting with hexane and ethyl acetate (8:2) mixture, starting from 2-(2-methoxyphenyl)-2-methyloxirane (82 mg, 0.5 mmol), Cs_2CO_3 (16.3 mg, 0.05 mmol; 10 mol%), 10% **Pd/C** (15.9 mg, 0.015 mmol, 3 mol%) and **Ir6** (2.1 mg, 0.0025 mmol, 0.5 mol%) in 2-propanol (2 mL) at 95 ºC. Colorless oil. ^{1}H NMR ($CDCl_3$, 400 MHz): δ (ppm) 7.23-7.19 (m, 2H), 6.95 (t, J = 7.6 Hz, 1H), 6.88 (d, J = 8.8 Hz, 1H), 3.83 (s, 3H), 3.75-3.66 (m, 2H), 3.48-2.39 (m, 1H), 1.26 (d, J = 6.8 Hz, 3H); $^{13}C\{^1H\}$ NMR ($CDCl_3$, 100 MHz): δ (ppm) 157.3, 127.4, 127.3, 120.8, 110.6, 67.8, 55.4, 35.2, 16.5.

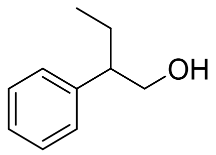

2-phenylbutan-1-ol (7e).[24] Following the general procedure (GP2e), the title product **7e** (50 mg, 66% yield) was isolated by using silica gel column chromatography eluting with hexane and ethyl acetate (9:1) mixture, starting from 2-ethyl-2-phenyloxirane (74 mg, 0.5 mmol), Cs_2CO_3 (16.3 mg, 0.05 mmol; 10 mol%), 10% **Pd/C** (15.9 mg, 0.015 mmol, 3 mol%) and **Ir6** (2.1 mg, 0.0025 mmol, 0.5 mol%) in 2-propanol (2 mL) at 95 ºC. Colorless oil. ^{1}H NMR ($CDCl_3$, 400 MHz): δ (ppm) 7.37-7.31 (m, 2H), 7.24-7.19 (m, 3H), 3.81-3.71 (m, 2H), 2.73-2.66 (m, 1H), 1.81-1.71 (m, 1H), 1.65-1.53 (m, 1H), 0.84 (t, J = 7.2 Hz, 3H); $^{13}C\{^1H\}$ NMR ($CDCl_3$, 100 MHz): δ (ppm) 142.2, 128.6, 128.1, 126.7, 67.3, 50.5, 24.9, 11.9.

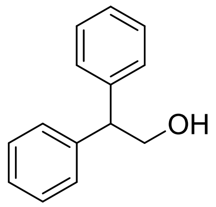

2,2-diphenylethanol (7f).[6,24] Following the general procedure (GP2e), the title product **7f** (89 mg, 90% yield) was isolated by using silica gel column chromatography eluting with hexane and ethyl acetate (9:1) mixture, starting from 2,2-diphenyloxirane (98 mg, 0.5 mmol), Cs_2CO_3 (16.3 mg, 0.05 mmol; 10 mol%), 10% **Pd/C** (15.9 mg, 0.015 mmol, 3 mol%) and **Ir6** (2.1 mg, 0.0025 mmol, 0.5 mol%) in 2-propanol (2 mL) at 95 ºC. White solid (m.p.: 55-57 ºC). ^{1}H NMR ($CDCl_3$, 400 MHz): δ (ppm) 7.35-7.22 (m, 10H), 4.24-4.17 (m, 3H), 1.49 (bs, 1H); ^{13}C{^{1}H} NMR ($CDCl_3$, 100 MHz): δ (ppm) 141.3, 128.7, 128.3, 126.8, 66.1, 53.6.

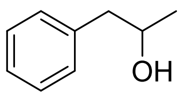

1-phenylpropan-2-ol (8a).[25] Following the general procedure (GP2f), the title product **8a** (66 mg, 98% yield) was isolated by using silica gel column chromatography eluting with hexane and ethyl acetate (95:5) mixture, starting from 2-methyl-3-phenyloxirane (67 mg, 0.5 mmol), KOH (5.6 mg, 0.1 mmol; 20 mol%), 10% **Pd/C** 21.3 mg, 0.02 mmol, 4 mol%) and **Ir6** (2.1 mg, 0.0025 mmol, 0.5 mol%) in 2-propanol (2 mL) at 95 ºC. Colorless oil. ^{1}H NMR ($CDCl_3$, 400 MHz): δ (ppm) 7.36-7.23 (m, 5H), 4.09-4.01 (m, 1H), 2.82 (dd, J_1 = 13.4 Hz, J_2 = 5.2 Hz, 1H), 2.72 (dd, J_1 = 13.4 Hz, J_2 = 8.0 Hz, 1H), 1.70 (bs, 1H), 1.27 (d, J = 6.0 Hz, 3H); ^{13}C{^{1}H} NMR ($CDCl_3$, 100 MHz): δ (ppm) 138.5, 129.4, 128.6, 126.5, 68.9, 45.8, 22.8.

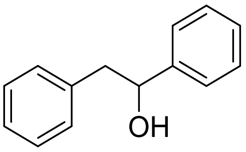

1,2-diphenylethanol (8b).[24] Following the general procedure (GP2f), the title product **8b** (90 mg, 91% yield) was isolated by using silica gel column chromatography eluting with hexane and ethyl acetate (95:5) mixture, starting from 2,3-diphenyloxirane (98 mg, 0.5 mmol), KOH (5.6 mg, 0.1 mmol; 20 mol%) 10% **Pd/C** 21.3 mg, 0.02 mmol, 4 mol%) and **Ir6** (2.1 mg, 0.0025 mmol, 0.5 mol%) in 2-propanol (2 mL) at 95 ºC. White solid (m.p.: 63-65 ºC). ^{1}H NMR ($CDCl_3$, 400 MHz): δ (ppm) 7.37-7.20 (m, 10H), 4.90 (t, *J* = 6.8 Hz, 1H), 3.08-2.97 (m, 2H), 2.03 (bs, 1H); ^{13}C{^{1}H} NMR ($CDCl_3$, 100 MHz): δ (ppm) 143.8, 138.0, 129.5, 128.5, 128.4, 127.6, 126.6, 125.9, 75.3, 46.1.

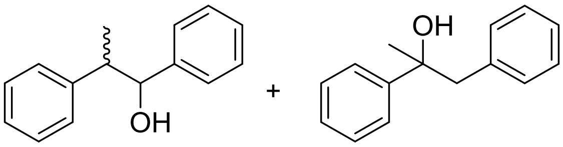

1,2-diphenylpropan-1-ol (8c)[42] + **1,2-diphenylpropan-2-ol (8c*).**[43] Following the general procedure (GP2f), a mixture of product (80 mg, 76% yield) **8c** (67%) and **8c*** (9%) was isolated by using silica gel column chromatography eluting with hexane and ethyl acetate (95:5) mixture, starting from 2-methyl-2,3-diphenyloxirane (105 mg, 0.5 mmol), KOH (5.6 mg, 0.1 mmol; 20 mol%), 10% **Pd/C** 21.3 mg, 0.02 mmol, 4 mol%) and **Ir6** (2.1 mg, 0.0025 mmol, 0.5 mol%) in 2-propanol (2 mL) at 95 ºC. White solid (m.p.: 70-72 ºC). **8c**: Mixture of diastereoisomers (77:23 *anti*:*syn*). ^{1}H NMR ($CDCl_3$, 400 MHz): δ (ppm) 7.40-7.23 (m, 10H, *syn* and *anti*), 4.81 (d, *J* = 8.8 Hz, 1H, *syn*), 4.68 (d, *J* = 8.8 Hz, 1H, *anti*), 3.17-3.09 (m, 1H, *syn*), 3.08-3.01 (m, 1H, *anti*), 1.98 (bs, 1H, *syn*), 1.93 (bs, 1H, *anti*), 1.33 (d, *J* = 6.8 Hz, 3H, *syn*), 1.33 (d, *J* = 6.8 Hz, 3H, *syn*), 1.10 (d, *J* = 6.8 Hz, 3H, *anti*); ^{13}C{^{1}H} NMR ($CDCl_3$, 100

MHz): δ (ppm) 143.5, 143.4, 142.8, 142.5, 130.6, 128.6, 128.2, 128.2, 128.0, 128.0, 127.9, 127.7, 127.1, 126.9, 126.9, 126.3, 126.2, 79.6, 78.6, 48.1, 47.2, 18.3, 14.9.

The side product **8c***: ^{1}H NMR ($CDCl_3$, 400 MHz): δ (ppm) δ 7.47-7.16 (m, 8H), 7.03-7.00 (m, 2H), 3.15 (d, J = 13.2 Hz, 1H), 3.11 (d, J = 13.6 Hz, 1H), 1.91 (s, 1H), 1.58 (s,3H). $^{13}C\{^1H\}$ NMR ($CDCl_3$, 100 MHz): δ (ppm) 147.5, 136.7, 130.6, 128.0, 126.6, 126.6, 124.9, 74.4, 50.4, 29.3.

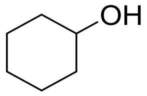

cyclohexanol (8d).[24,27] Following the general procedure (GP2f), the title product **8d** (46 mg, 93% yield) was isolated by using silica gel column chromatography eluting with hexane and ethyl acetate (95:5) mixture, starting from 7-oxabicyclo[4.1.0]heptane (49 mg, 0.5 mmol), KOH (5.6 mg, 0.1 mmol; 20 mol%) 10% **Pd/C** 21.3 mg, 0.02 mmol, 4 mol%) and **Ir6** (2.1 mg, 0.0025 mmol, 0.5 mol%) in 2-propanol (2 mL) at 95 ºC. Colorless oil. ^{1}H NMR ($CDCl_3$, 400 MHz): δ (ppm) 3.65-3.58 (m, 1H), 1.92-1.88 (m, 2H), 1.77-1.70 (m, 3H), 1.55 (bs, 1H), 1.35-1.12 (m, 5H); $^{13}C\{^1H\}$ NMR ($CDCl_3$, 100 MHz): 70.3, 35.5, 25.5, 24.1.

4. Mechanistic Control Experiments

4.1. Procedure for the time profile of the NHC-Ir catalyzed TH of 1d

Seven identical reactions were performed in different reaction tubes for a specified time (0.5, 1, 2, 4, 8, 12, and 16 h). To a 20 mL reaction tube with a condenser, 2-(4-chlorophenyl)oxirane (77 mg, 0.5 mmol), KOH (2.8 mg, 0.05 mmol, 10 mol%) and **Ir6** (2.1 mg, 0.0025 mmol, 0.5 mol%) in 2-propanol (1.0 mL) were added under open air conditions. The reaction mixture was vigorously stirred under reflux in a preheated oil bath at 95 °C for a specified time. After cooling to ambient temperature, internal standard (1,3,5-trimethoxybenzene) was added into the reaction mixture and the yields were calculated through ^{1}H NMR analysis.

4.2. Procedure for control experiments on the NHC−Ir catalyzed TH of epoxides

4.2.a. Isomerization of 1a: To a 20 mL reaction tube with a condenser, 2-phenyloxirane (60 mg, 0.5 mmol), KOH (2.8 mg, 0.05 mmol; 10 mol%), **Ir6** (2.1 mg, 0.0025 mmol, 0.5 mol%) and *tert*-amyl alcohol or acetonitrile (1.0 mL) were added under open air conditions. The reaction mixture was vigorously stirred in a preheated oil bath at 95 °C for 16 h. After cooling to ambient temperature, internal standard (1,3,5-trimethoxybenzene) was added into the reaction mixture and the yields were calculated through ^{1}H NMR analysis.

4.2.b. Racemization of 1a: To a 20 mL reaction tube with a condenser, (R)-2-phenyloxirane (60 mg, 0.5 mmol), KOH (2.8 mg, 0.05 mmol; 10 mol%), **Ir6** (2.1 mg, 0.0025 mmol, 0.5 mol%) and 2-propanol (1.0 mL) were added under open air conditions. The reaction mixture was vigorously stirred under reflux in a preheated oil bath at 95 °C for 16 h. After cooling to room temperature, the solvent was evaporated under reduced pressure and the resulting mixture was purified by using silica gel column chromatography eluting with hexane and ethyl acetate (8:2) mixture. $[\alpha]_D^{25} = 0.0$ (c 1.0, CH_2Cl_2)

4.2.c. Dehydrogenation of 4a: To a 20 mL reaction tube with a condenser, 1-phenylethanol (61 mg, 0.5 mmol), KOH (2.8 mg, 0.05 mmol; 10 mol%), **Ir6** (2.1 mg, 0.0025 mmol, 0.5 mol%) and *tert*-amyl alcohol (1.0 mL) were added under open air conditions. The reaction mixture was vigorously stirred in a preheated oil bath at 95 °C for 16 h. After cooling to ambient temperature, internal standard (1,3,5-trimethoxybenzene) was added into the reaction mixture and the yield was calculated through ^{1}H NMR analysis.

4.2.d. Transfer hydrogenation of 9: To a 20 mL reaction tube with a condenser, acetophenone (60 mg, 0.5 mmol), KOH (2.8 mg, 0.05 mmol; 10 mol%), **Ir6** (2.1 mg, 0.0025 mmol, 0.5 mol%) and 2-propanol (1.0 mL) were added under open air conditions. The reaction mixture was vigorously stirred under reflux in a preheated oil bath at 95 °C for 30 min. After cooling to

ambient temperature, internal standard (1,3,5-trimethoxybenzene) was added into the reaction mixture and the yield was calculated through ^{1}H NMR analysis.

4.2.e. Reversible dehydrogenation & transfer hydrogenation of 4a-D: To a 20 mL reaction tube with a condenser, 1-deuterio-1-phenylethanol (61 mg, 0.5 mmol), KOH (2.8 mg, 0.05 mmol; 10 mol%), **Ir6** (2.1 mg, 0.0025 mmol, 0.5 mol%) and 2-propanol (1.0 mL) were added under open air conditions. The reaction mixture was vigorously stirred under reflux in a preheated oil bath at 95 °C for 16 h. After cooling to ambient temperature, internal standard (1,3,5-trimethoxybenzene) was added into the reaction mixture and the yields were calculated through ^{1}H NMR analysis.

4.3. Procedure for control experiments on the NHC−Ir & Pd/C catalyzed TH of epoxides

4.3.a. Isomerization of 1a: To a 20 mL reaction tube with a condenser, 2-phenyloxirane (0.5 mmol), Cs_2CO_3 (16.3 mg, 0.05 mmol; 10 mol%), **Ir6** (2.1 mg, 0. 0025 mmol, 0.5 mol%), 10% **Pd/C** (10.6 mg, 0.01 mmol, 2 mol%) and acetonitrile (2.0 mL) were added under open air conditions. The reaction mixture was vigorously stirred under reflux in a preheated oil bath at 95 °C for 20 h. After cooling to ambient temperature, internal standard (1,3,5-trimethoxybenzene) was added into the reaction mixture and the yield was calculated through ^{1}H NMR analysis.

4.3.b. Transfer hydrogenation of 10: To a 20 mL reaction tube with a condenser, 2-phenylacetaldehyde (0.5 mmol), Cs_2CO_3 (16.3 mg, 0.05 mmol; 10 mol%), **Ir6** (2.1 mg, 0. 0025 mmol, 0.5 mol%) 10% **Pd/C** (10.6 mg, 0.01 mmol, 2 mol%) and 2-propanol (2.0 mL) were added under open air conditions. The reaction mixture was vigorously stirred under reflux in a preheated oil bath at 95 °C for 20 h. After cooling to ambient temperature, internal standard (1,3,5-trimethoxybenzene) was added into the reaction mixture and the yield was calculated through ^{1}H NMR analysis.

4.3.c. Isomerization of terminal epoxides in the absence of a base: To a 20 mL reaction tube with a condenser, 2-phenyloxirane (0.5 mmol), **Ir6** (2.1 mg, 0. 0025 mmol, 0.5 mol%) and/or 10% **Pd/C** (10.6 mg, 0.01 mmol, 2 mol%) and acetonitrile (2.0 mL) were added under open air conditions. The reaction mixture was vigorously stirred under reflux in a preheated oil bath at 95 °C for 20 h. After cooling to ambient temperature, internal standard (1,3,5-trimethoxybenzene) was added into the reaction mixture and the yields were calculated through ^{1}H NMR analysis.

4.3.d. Reaction of terminal epoxides with other nucleophiles: To a 20 mL reaction tube with a condenser; *for amine as a nucleophile:* 2-phenyloxirane or 2-decyloxirane (0.5 mmol), 4-tolyl amine (0.5 mmol), 10% **Pd/C** (10.6 mg, 0.01 mmol, 2 mol%) and acetonitrile (2.0 mL), *for alcohol as a nucleophile:* 2-phenyloxirane or 2-decyloxirane (0.5 mmol), 10% **Pd/C** (10.6 mg, 0.01 mmol, 2 mol%) and 2-propanol (2.0 mL), were added under open air conditions. The reaction mixture was vigorously stirred under reflux in a preheated oil bath at 95 °C for 20 h. After cooling to ambient temperature, internal standard (1,3,5-trimethoxybenzene) was added into the reaction mixture and the yields were calculated through ^{1}H NMR analysis.

4.3.e. Poisoning experiments: To a 20 mL reaction tube with a condenser, 2-phenyloxirane (0.5 mmol), Cs_2CO_3 (16.3 mg, 0.05 mmol; 10 mol%), **Ir6** (2.1 mg, 0. 0025 mmol, 0.5 mol%), 10% **Pd/C** (10.6 mg, 0.01 mmol, 2 mol%), additive (PPh_3, thiophene or pyrene) (0.1 or 1 equiv.) and 2-propanol (2.0 mL) were added under open air conditions. The reaction mixture was vigorously stirred under reflux in a preheated oil bath at 95 °C for 4 or 16 h. After cooling to ambient temperature, internal standard (1,3,5-trimethoxybenzene) was added into the reaction mixture and the yields were calculated through ^{1}H NMR analysis.

4.4. Procedure for the Hammett studies

Four identical reactions were performed in different reaction tubes for a specified time (0.5, 1, 1.5, and 2 h) to calculate the initial rate of the reaction. To a 20 mL reaction tube with a

condenser; *for NHC−Ir catalyzed TH of epoxides:* 2-phenyloxirane (0.5 mmol), KOH (2.8 mg, 0.05 mmol; 10 mol%), **Ir6** (2.1 mg, 0.0025 mmol, 0.5 mol%) and 2-propanol (1.0 mL), *for NHC−Ir & Pd/C catalyzed TH of epoxides:* 2-phenyloxirane (0.5 mmol), Cs_2CO_3 (16.3 mg, 0.05 mmol; 10 mol%), **Ir6** (2.1 mg, 0.0025 mmol, 0.5 mol%) and 2-propanol (2.0 mL) were added under open air conditions. The reaction mixture was vigorously stirred under reflux in a preheated oil bath at 95 °C for a specified time. After cooling to ambient temperature, internal standard (1,3,5-trimethoxybenzene) was added into the reaction mixture and the yields were calculated through ^{1}H NMR analysis. This procedure was repeated for other *para*-substituted ($-CH_3$, −Cl, $-CF_3$) 2-phenyloxiranes. The graphs of the logarithm of the substrate concentration vs time were created. The slopes of the curves for each experiment equal the initial rates of the reaction under the corresponding conditions.

The k_{rel} ($\log(k_X/k_H)$) values were calculated as follows:

$$k_{rel} = k_R/k_H = (\ln(R_f/R_i))/(\ln(H_f/H_i))$$

R_f and R_i are final and initial concentration of *para*-substituted 2-phenyloxirane respectively; H_f and H_i are final and initial concentration of 2-phenyloxirane respectively.

Using the initial rate for each TH reaction, a Hammett plot of $\log(k_X/k_H)$ (k_{rel}) against the substituent constant σ_p could be constructed.

5. References

[1] Genç, S.; Gülcemal, S.; Gülcemal, D.; Günnaz, S.; Çetinkaya, B. Synthesis of α-Alkylated Ketones via Selective Epoxide Opening/Alkylation Reactions with Primary Alcohols. *Org. Lett.* **2021**, *23*, 5229–5234.

[2] Genç, S.; Günnaz, S.; Çetinkaya, B.; Gülcemal, S.; Gülcemal, D. Iridium(I)-Catalyzed Alkylation Reactions to Form α-Alkylated Ketones. *J. Org. Chem.* **2018**, *83*, 2875−2881.

[3] Genç, S.; Gülcemal, S.; Günnaz, S.; Çetinkaya, B.; Gülcemal, D. Iridium-Catalyzed Alkylation of Secondary Alcohols with Primary Alcohols: A Route to Access Branched Ketones and Alcohols. *J. Org. Chem.* **2020**, *85*, 9139−9152.

[4] Li, S.; Shi, Y.; Li, P.; Xu, J. Nucleophilic Organic Base DABCO-Mediated Chemospecific Meinwald Rearrangement of Terminal Epoxides into Methyl Ketones. *J. Org. Chem.* **2019**, *84*, 4443−4450.

[5] Liu, W.; Li, W.; Spannenberg, A.; Junge, K.; Beller, M. Iron-Catalysed Regioselective Hydrogenation of Terminal Epoxides to Alcohols under Mild Conditions. *Nat. Catal.* **2019**, *2*, 523−528.

[6] Huang, C.; Ma, W.; Zheng, X.; Xu, M.; Qi, X.; Lu, Q. Epoxide Electroreduction. *J. Am. Chem. Soc.* **2022**, *144*, 1389−1395.

[7] Kavanagh, S. A.; Piccinini, A.; Fleming, E. M.; Connon, S. J. Urea Derivatives Are Highly Active Catalysts for the Base-Mediated Generation of Terminal Epoxides from Aldehydes and Trimethylsulfonium Iodide. *Org. Biomol. Chem.* **2008**, *6*, 1339–1343.

[8] Pandey, A. K.; Banerjee, P. One-Pot Synthesis of Oxazolidine Derivatives by [3+2]-Annulation Reactions of 1-Tosyl-2-phenyl/alkylaziridines with Aryl Epoxides. *Asian J. Org. Chem.* **2016**, *5*, 360–366.

[9] De Almeida, C. G.; Reis, S. G.; de Almeida, A. M.; Diniz, C. G.; da Silva, V. L.; Le Hyaric, M. Synthesis and Antibacterial Activity of Aromatic and Heteroaromatic Amino Alcohols. *Chem. Biol. Drug Des.* **2011**, *78*, 876–880.

[10] Barbe, G.; Charette, A. B. Highly Chemoselective Metal-Free Reduction of Tertiary Amides. *J. Am. Chem. Soc.* **2008**, *130*, 18–19.

[11] Zhou, L.; Zhou, L.; Xiang, S.; Zhang, K.; Chen, M. Dual-Responsive Epoxy Thermosets with Controlled Degradation and High Performance Based on Serial Connection of Double Dynamic Bonds. *Polymer* **2023**, *283*, 126234.

[12] Yook, J.; Jeong, D.; Lee, C. J. Synthesis of Citronellol-Derived Antibacterial Polymers and Effect of Thioether, Sulfoxide, Sulfone, and Ether Functional Groups on Their Bactericidal Activity. *Macromolecules* **2023**, *56*, 3406–3420.

[13] Araujo, Y. J. K.; Avvari, N. P.; Paiva, D. R.; de Lima, D. P.; Beatriz, A. Synthesis and Enzymatic Resolution of Racemic 2,3-Epoxy Propyl Esters Obtained from Glycerol. *Tetrahedron Lett.* **2015**, *56*, 1696–1698.

[14] Apparu, M.; Ben Tiba, Y.; Léo, P.-M.; Hamman, S.; Coulombeau, C. Determination of the Enantiomeric Purity and the Configuration of β-aminoalcohols using (R)-2-Fluorophenylacetic Acid (AFPA) and Fluorine-19 NMR: Application to β-blockers. *Tetrahedron: Asymmetry* **2000**, *11*, 2885–2898.

[15] Vyas, D. J.; Larionov, E.; Besnard, C.; Guénee, L.; Mazet, C. Isomerization of Terminal Epoxides by a [Pd−H] Catalyst: A Combined Experimental and Theoretical Mechanistic Study. *J. Am. Chem. Soc.* **2013**, *135*, 6177–6183.

[16] Sone, T.; Yamaguchi, A.; Matsunaga, S.; Shibasaki, M. Catalytic Asymmetric Synthesis of 2,2-Disubstituted Terminal Epoxides via Dimethyloxosulfonium Methylide Addition to Ketones. *J. Am. Chem. Soc.* **2008**, *130*, 10078–10079.

[17] Cabré, A.; Cabezas-Giménez, J.; Sciortino, G.; Ujaque, G.; Verdaguer, X.; Lledós, A.; Riera, A. Mild Iridium-Catalysed Isomerization of Epoxides. Computational Insights and Application to the Synthesis of β-Alkyl Amines. *Adv. Synth. Catal.* **2019**, *361*, 3624–3631.

[18] Aida, K.; Hirao, M.; Funabashi, A.; Sugimura, N.; Ota, E.; Yamaguchi, J. Catalytic Reductive Ring Opening of Epoxides Enabled by Zirconocene and Photoredox Catalysis. *Chem* **2022**, *8*, 1762–1774.

[19] Liu, W.; Leischner, T.; Li, W.; Junge, K.; Beller, M. A General Regioselective Synthesis of Alcohols by Cobalt-Catalyzed Hydrogenation of Epoxides. *Angew. Chem., Int. Ed.* **2020**, *59*, 11321−11324.

[20] Zhang, Z.-W.; Li, H.-B.; Li, J.; Wang, C.-C.; Feng, J.; Yang, Y.-H.; Liu, S. Synthesis of Epoxides from Alkyl Bromides and Alcohols with in Situ Generation of Dimethyl Sulfonium Ylide in DMSO Oxidations. *J. Org. Chem.* **2020**, *85*, 537–547.

[21] Davis, C. E.; Bailey, J. L.; Lockner, J. W.; Coates, R. M. Regio- and Stereoselectivity of Diethylaluminum Azide Opening of Trisubstituted Epoxides and Conversion of the 3° Azidohydrin Adducts to Isoprenoid Aziridines. *J. Org. Chem.* **2003**, *68*, 75–82.

[22] Carvalho, J. F. S.; Silva, M. M. C.; Melo, M. L. S. Highly Efficient Epoxidation of Unsaturated Steroids Using Magnesium Bis(Monoperoxyphthalate) Hexahydrate. *Tetrahedron* **2009**, *65*, 2773–2781.

[23] Ma, E.; Kim, H.; Kim, E. Epoxidation and Reduction of Cholesterol, 1,4,6-Cholestatrien-3-One and 4,6-Cholestadien-3β-ol. *Steroids* **2005**, *70*, 245–250.

[24] Magre, M.; Paffenholz, E.; Maity, B.; Cavallo, L.; Rueping, M. Regiodivergent Hydroborative Ring Opening of Epoxides via Selective C–O Bond Activation. *J. Am. Chem. Soc.* **2020**, *142*, 14286–14294.

[25] Thiyagarajan, S.; Gunanathan, C. Ruthenium-Catalyzed Selective Hydrogenation of Epoxides to Secondary Alcohols. *Org. Lett.* **2019**, *21*, 9774–9778.

[26] Chen, S.; Yan, D.; Xue, M.; Hong, Y.; Yao, Y.; Shen, Q. Tris(cyclopentadienyl)lanthanide Complexes as Catalysts for Hydroboration Reaction toward Aldehydes and Ketones. *Org. Lett.* **2017**, *19*, 3382–3385.

[27] Bruneau-Voisine, A.; Wang, D.; Dorcet, V.; Roisnel, T.; Darcel, C.; Sortais, J.-B. Transfer Hydrogenation of Carbonyl Derivatives Catalyzed by an Inexpensive Phosphine-Free Manganese Precatalyst. *Org. Lett.* **2017**, *19*, 3656–3659.

[28] Andrade, L. H.; Barcellos, T. Lipase-Catalyzed Highly Enantioselective Kinetic Resolution of Boron-Containing Chiral Alcohol. *Org. Lett.* **2009**, *11*, 3052–3055.

[29] Liu, J.-T.; Yang, S.; Tang, W.; Yang, Z.; Xu, J. Iridium-Catalyzed Efficient Reduction of Ketones in Water with Formic Acid as a Hydride Donor at Low Catalyst Loading. *Green Chem.* **2018**, *20*, 2118–2124.

[30] Funk, B. E.; Pauze, M.; Lu, Y.-C.; Moser, A. J.; Wolf, G.; West, J. G. Vitamin B12 and Hydrogen Atom Transfer Cooperative Catalysis as a Hydride Nucleophile Mimic in Epoxide Ring Opening. *Cell Rep. Phys. Sci.* **2023**, *4*, 101372.

[31] Harfenist, M.; Thom, E. Smiles Rearrangement on Borohydride Reduction of a Nitrophenoxy Ester. *J. Org. Chem.* **1971**, *36 (9)*, 1204–1207.

[32] Unglaube, F.; Atia, H.; Bartling, S.; Kreyenschulte, C. R.; Mejía, E. Hydrogenation of Epoxides to Anti-Markovnikov Alcohols over a Nickel Heterogeneous Catalyst Prepared from Biomass (Rice) Waste. *Helv. Chim. Acta* **2023**, *106*, e202200167.

[33] Li, X.; Wang, K.; Li, Y.-G.; Zhao, Q.; Ma, Y.-N.; Chen, X. A Borenium–Borane Composite for Exhaustive Reduction of Oxo-Chemicals. *J. Am. Chem. Soc.* **2025**, *147*, 1893–1902.

[34] Li, L.; Herzon, S. B. Temporal Separation of Catalytic Activities Allows Anti-Markovnikov Reductive Functionalization of Terminal Alkynes. *Nat. Chem.* **2014**, *6*, 22–27.

[35] Matsumoto, S.; Naito, M.; Oseki, T.; Akazome, M.; Otani, Y. Selective Reaction of Benzyl Alcohols with HI Gas: Iodination, Reduction, and Indane Ring Formations. *Tetrahedron* **2017**, *73*, 7254–7259.

[36] Gu, J.; Yang, H.; Deng, J.; Jiang, D.; Lv, K.; Wang, T.; Yao, Q. A General Photoinduced Oxidative Strategy with Molecular Oxygen in Water. *Org. Chem. Front.* **2024**, *11*, 1910–1916.

[37] Arnodo, D.; Ramos-Martín, M.; Cicco, L.; Capriati, V.; Ríos-Lombardía, N.; González-Sabín, J.; Presa Soto, A.; García-Álvarez, J. From Oximes to Tertiary Alcohols in Water, at Room Temperature and under Air: A Hybrid One-Pot Tandem Assembly of Enzymatic Deoximation and RLi/RMgX Reagents. *Org. Biomol. Chem.* **2023**, *21*, 4414–4421

[38] Luo, W.; Zhang, L.-M.; Zhang, Z.-M.; Zhang, J. Synthesis of W-Phos Ligand and Its Application in the Copper-Catalyzed Enantioselective Addition of Linear Grignard Reagents to Ketones. *Angew. Chem. Int. Ed.* **2022**, *61*, e202204443.

[39] Hamada, S.; Sugimoto, K.; Elboray, E. E.; Kawabata, T.; Furuta, T. Chemoselective Oxidation of p-Methoxybenzyl Ethers by an Electronically Tuned Nitroxyl Radical Catalyst. *Org. Lett.* **2020**, *22*, 5486–5490.

[40] Zhou, X.; Wang, Z.; Chen, Z.-N.; Yang, Y. Phosphine-Built-In Porous Organic Cage Supported Ultrafine Pd Nanoclusters Enable Highly Efficient and Regioselective Hydrogenation of Epoxides. *CCS Chem.* **2024**, *6*, 2476–2488.

[41] Nandi, P. G.; Jasra, R. V.; Kumar, A. Pincer–Ruthenium-Catalyzed β-Methylation of Alcohols. *Organometallics* **2023**, *42*, 3138–3152.

[42] Henriques, D. S. G.; Zimmer, K.; Klare, S.; Meyer, A.; Rojo-Wiechel, E.; Bauer, M.; Sure, R.; Grimme, S.; Schiemann, O.; Flowers, R. A., II; Gansäuer, A. Highly Active Titanocene Catalysts for Epoxide Hydrosilylation: Synthesis, Theory, Kinetics, EPR Spectroscopy. *Angew. Chem., Int. Ed.* **2016**, 55, 7671−7675.

[43] Li, C.-C.; Dai, X.-J.; Wang, H.; Zhu, D.; Gao, J.; Li, C.-J. Iron-Catalyzed Nucleophilic Addition Reaction of Organic Carbanion Equivalents via Hydrazones. *Org. Lett.* **2018**, *20*, 3801–3805.

6. Traces of ^{1}H and ^{13}C NMR spectra of epoxides

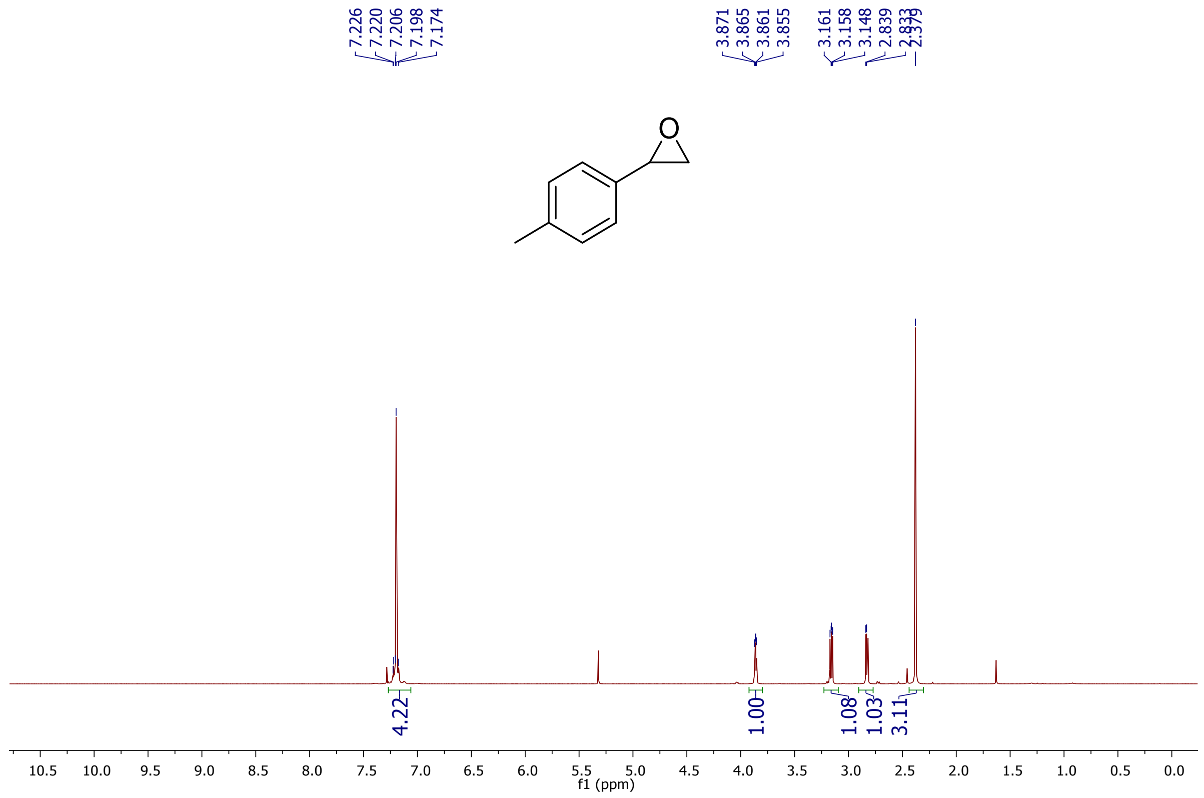

Figure S1. ^{1}H (400 MHz, $CDCl_3$) NMR spectra of **1b**

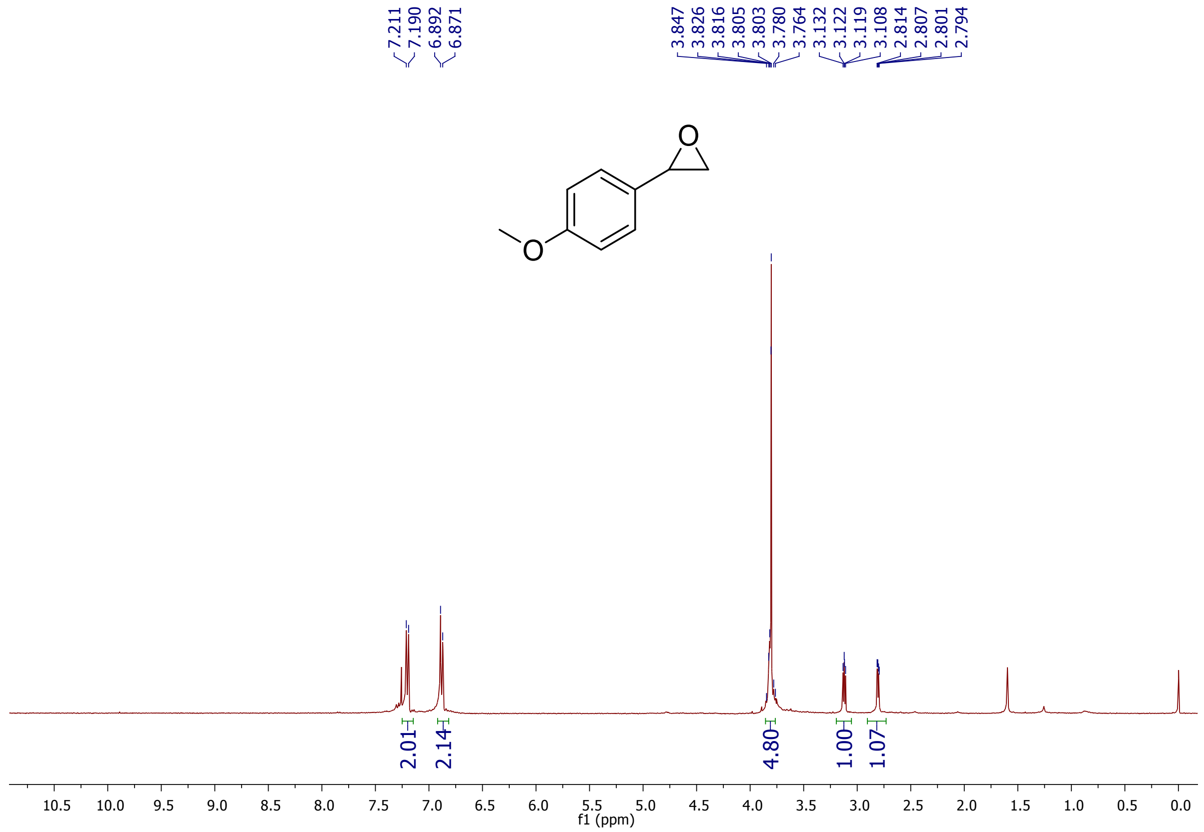

Figure S2. ^{1}H (400 MHz, $CDCl_3$) NMR spectra of **1c**

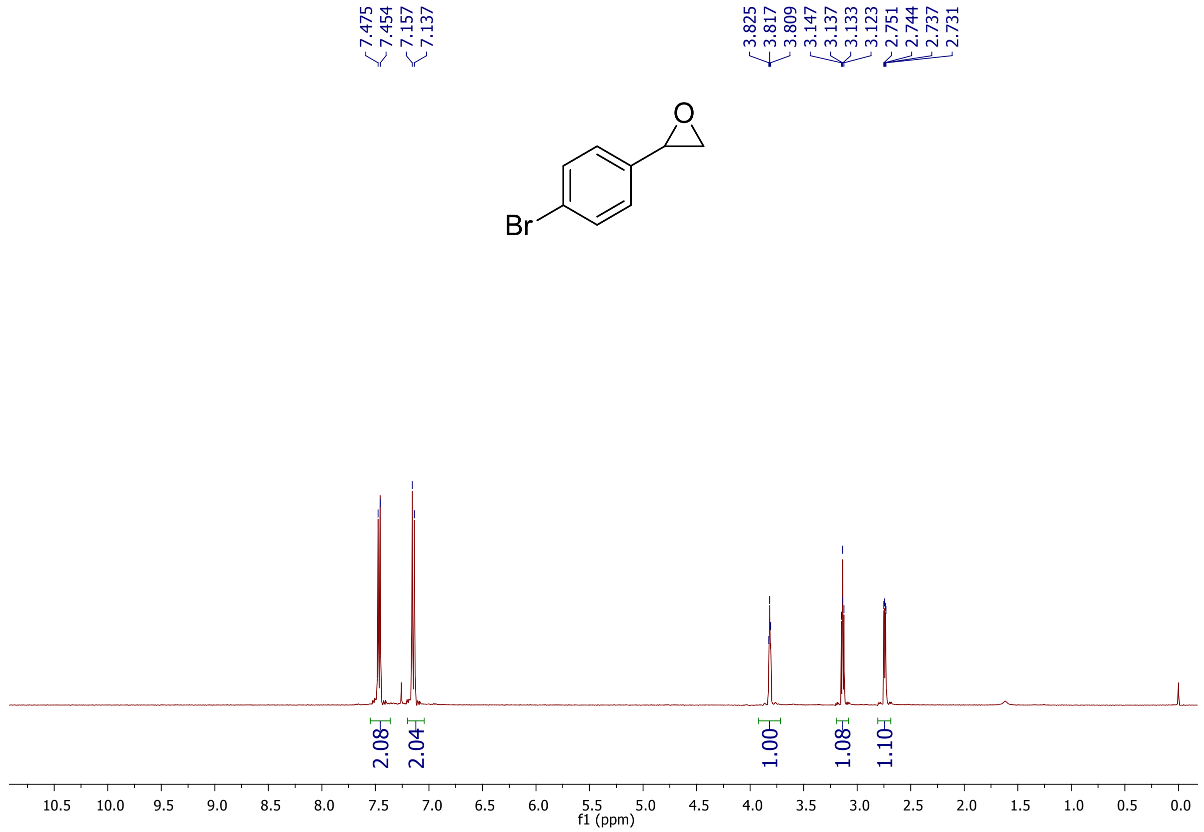

Figure S3. ^{1}H (400 MHz, $CDCl_3$) NMR spectra of **1e**

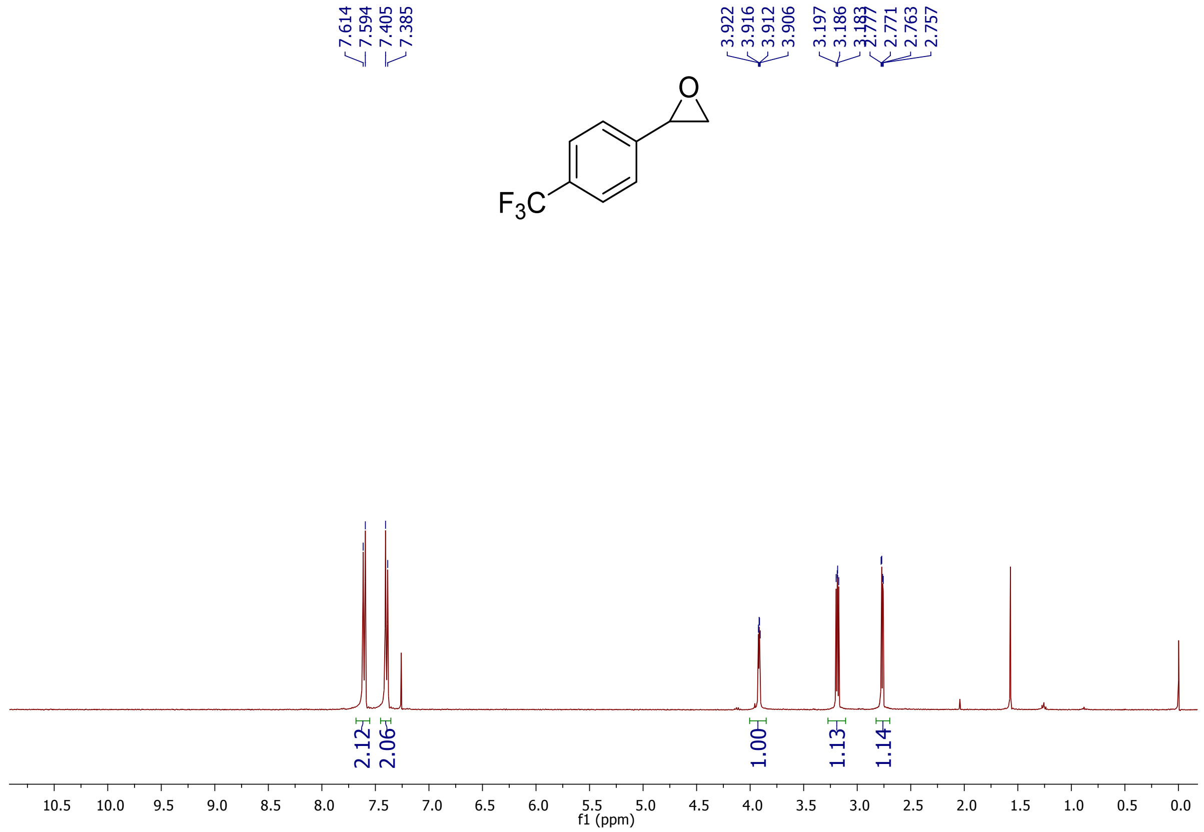

Figure S4. ^{1}H (400 MHz, $CDCl_3$) NMR spectra of **1g**

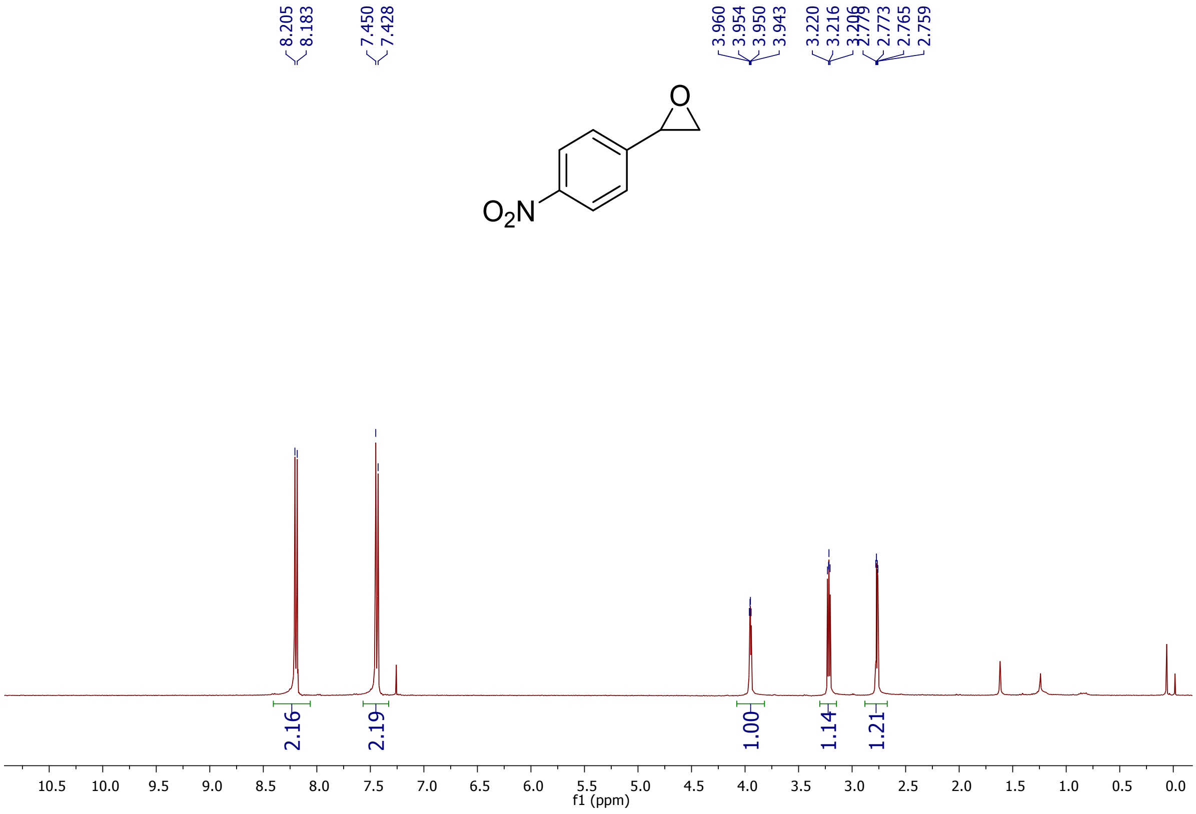

Figure S5. ^{1}H (400 MHz, $CDCl_3$) NMR spectra of **1h**

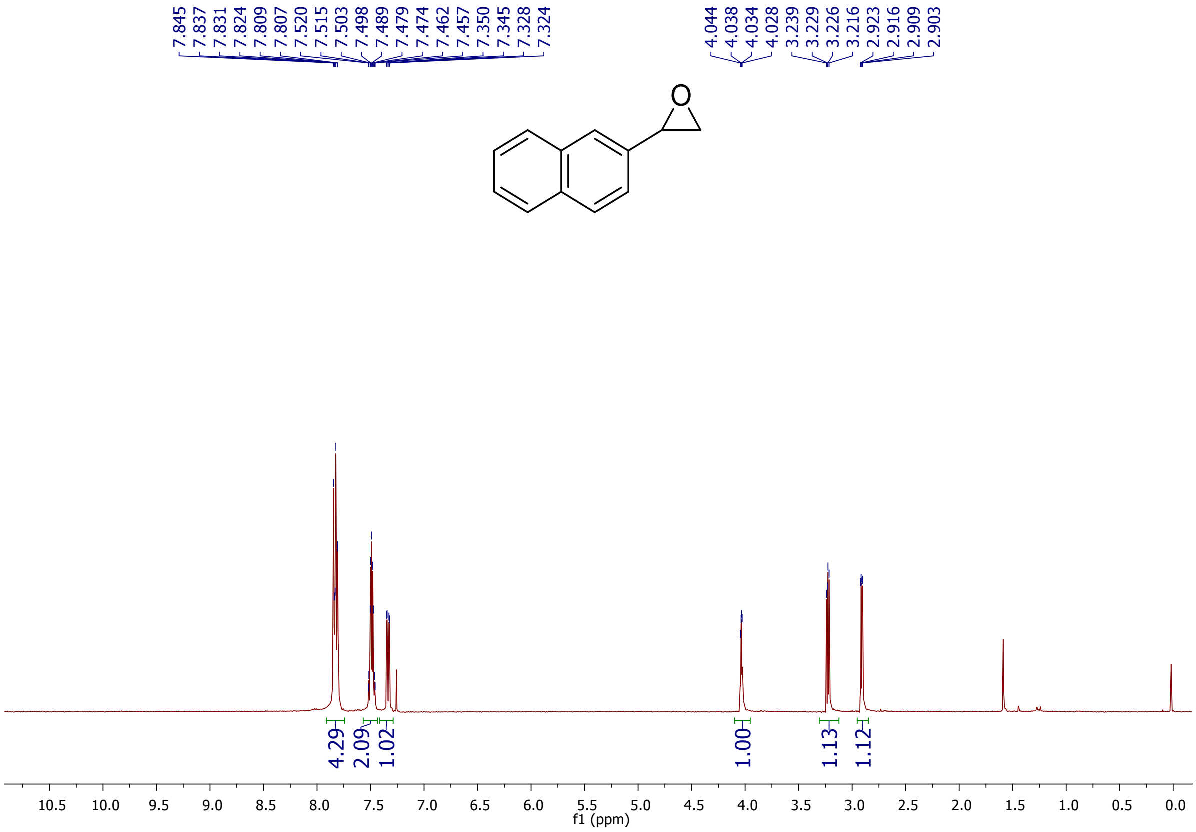

Figure S6. ^{1}H (400 MHz, $CDCl_3$) NMR spectra of **1i**

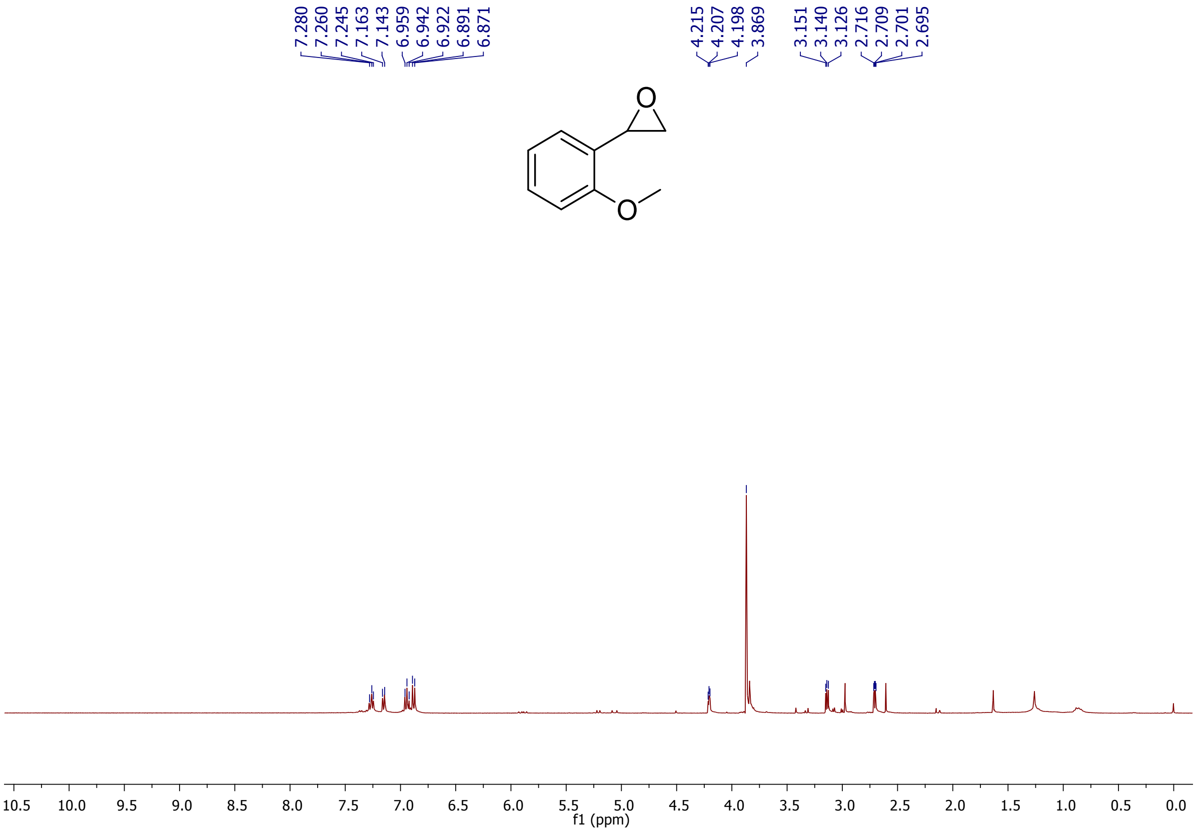

Figure S7. ^{1}H (400 MHz, $CDCl_3$) NMR spectra of **1k**

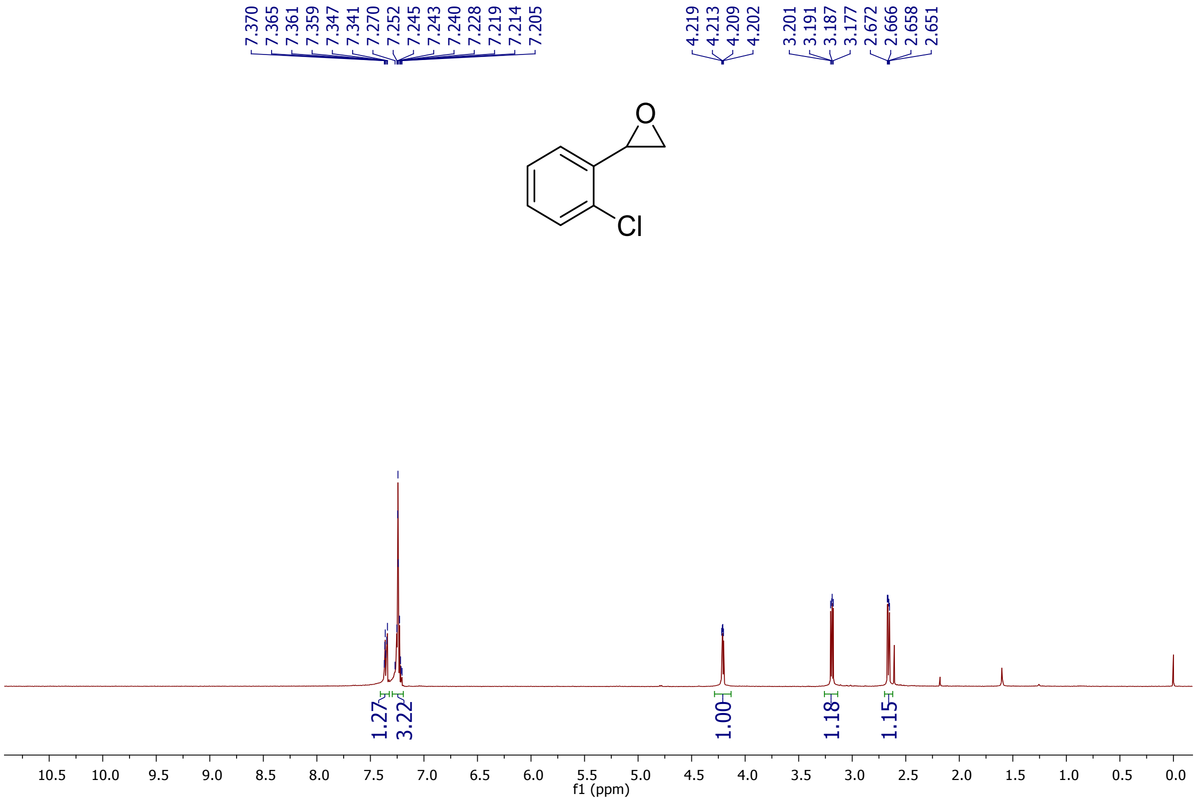

Figure S8. ^{1}H (400 MHz, $CDCl_3$) NMR spectra of **1l**

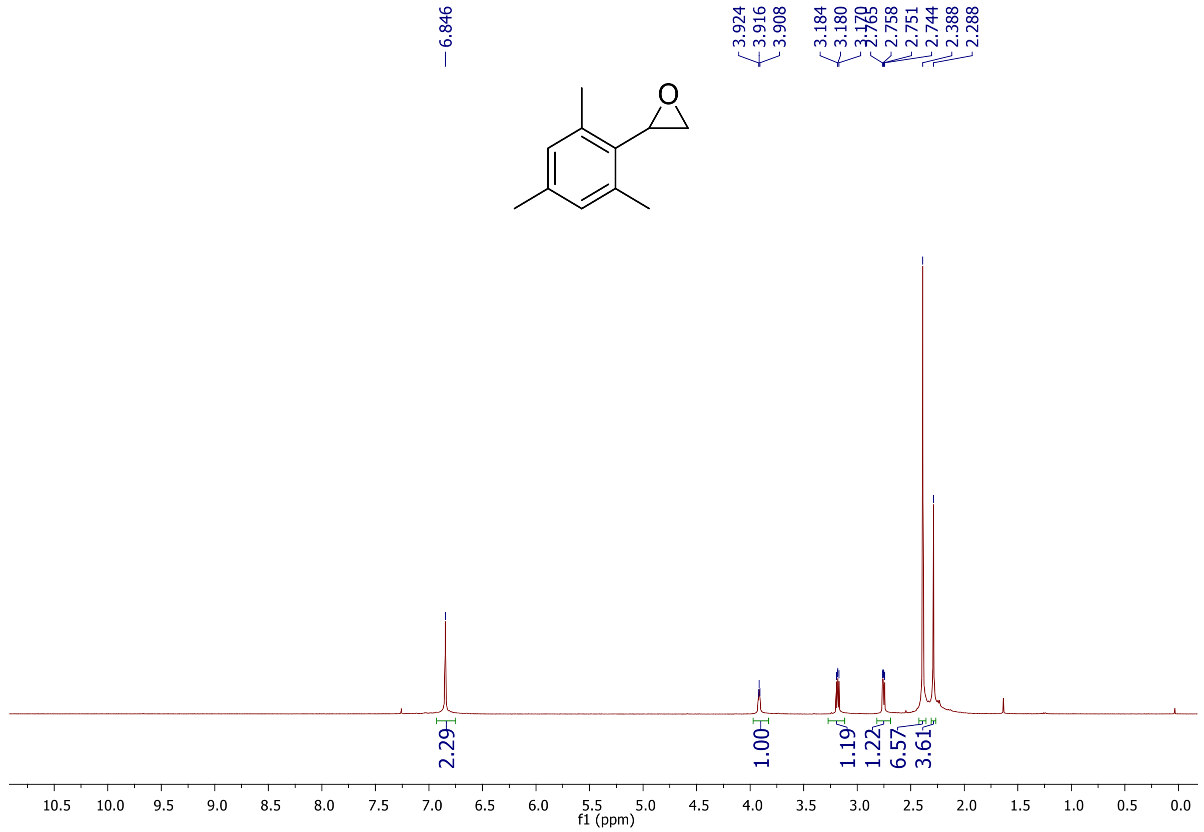

Figure S9. ^{1}H (400 MHz, $CDCl_3$) NMR spectra of **1m**

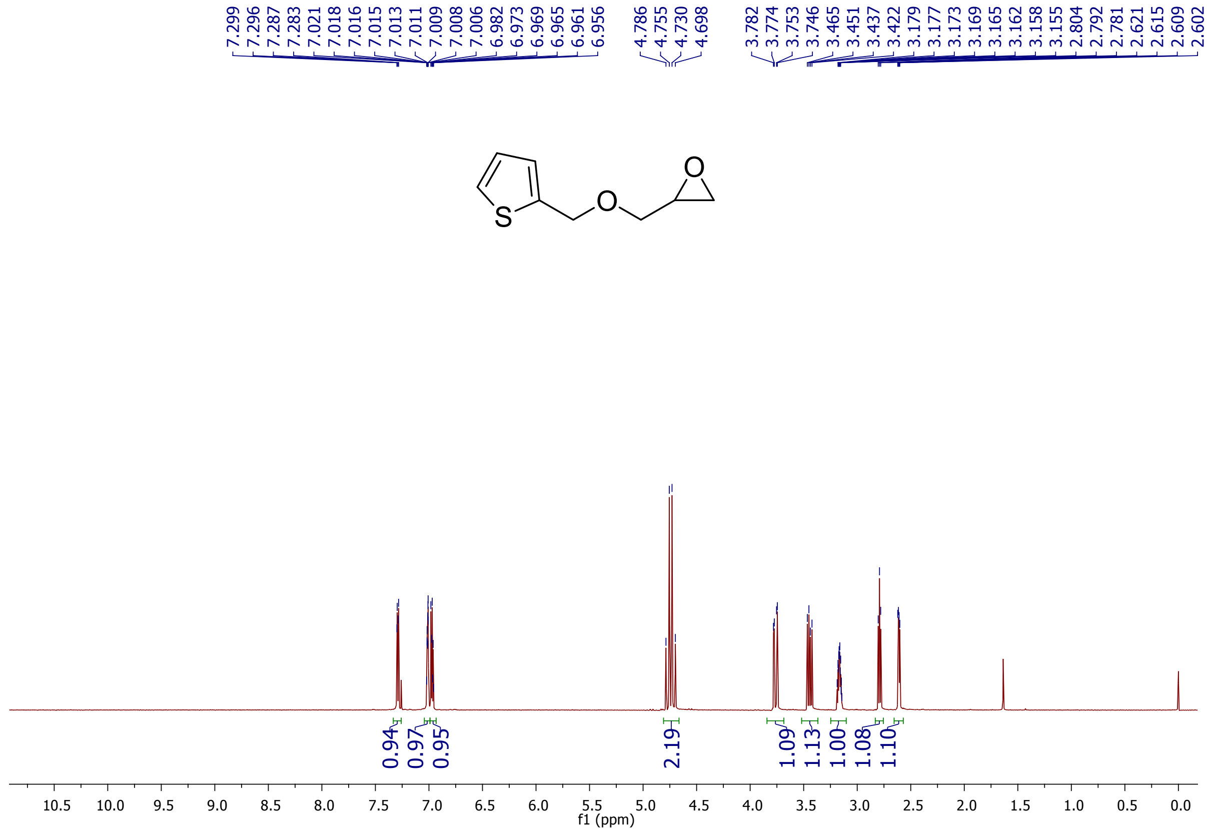

Figure S10. ^{1}H (400 MHz, $CDCl_3$) NMR spectra of **1p**

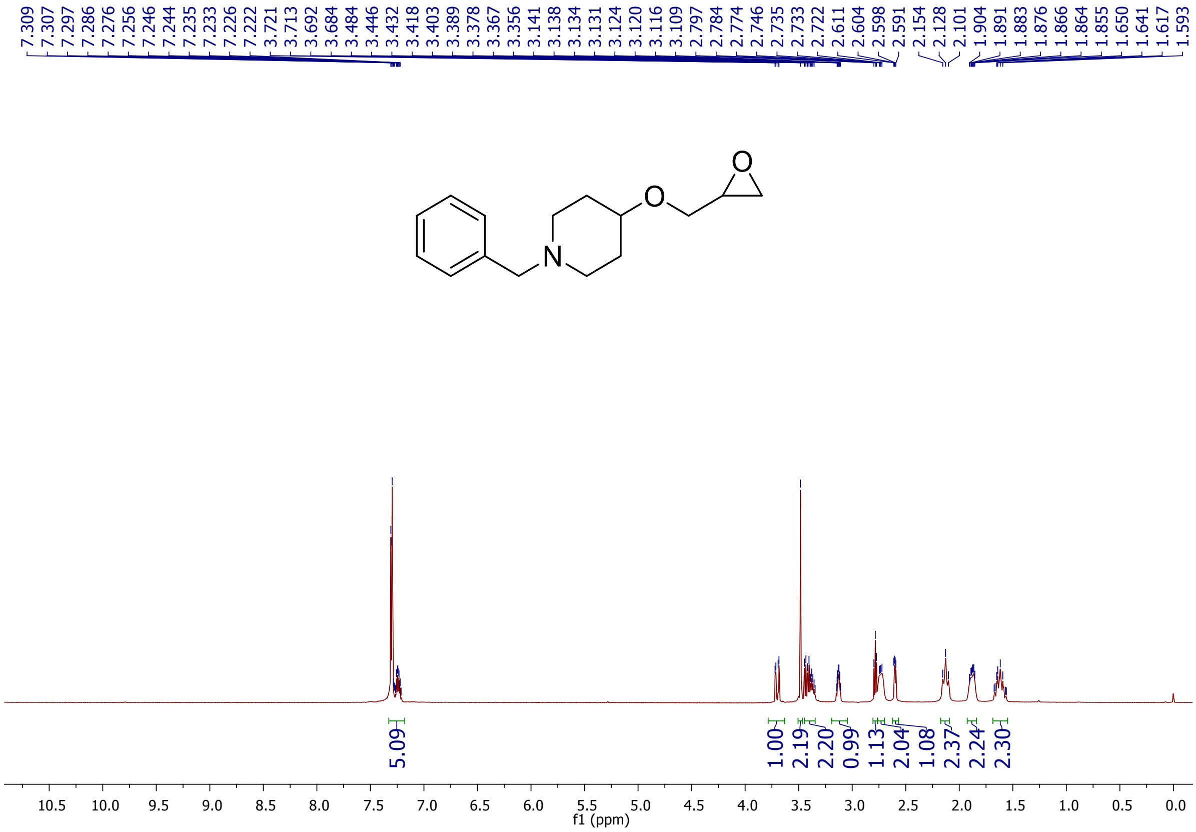

Figure S11. ^1H (400 MHz, $CDCl_3$) NMR spectra of **1q**

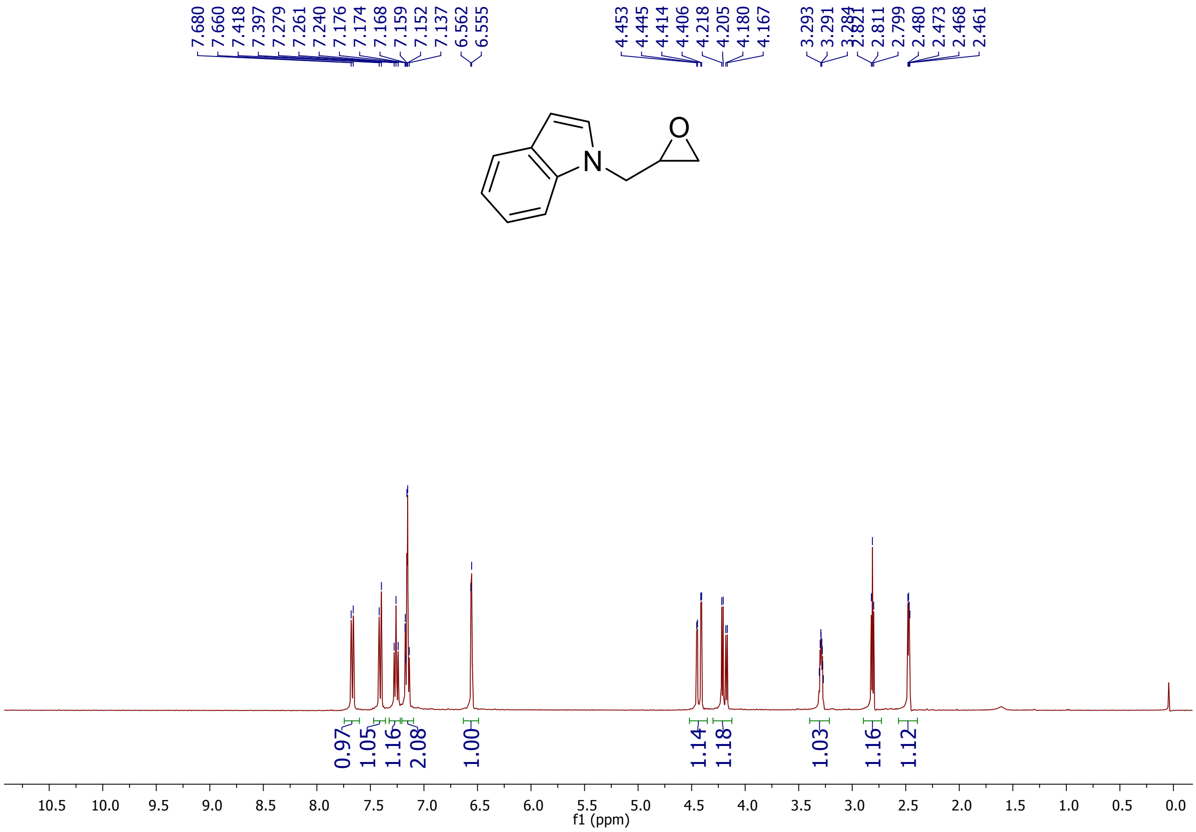

Figure S12. ^1H (400 MHz, $CDCl_3$) NMR spectra of **1r**

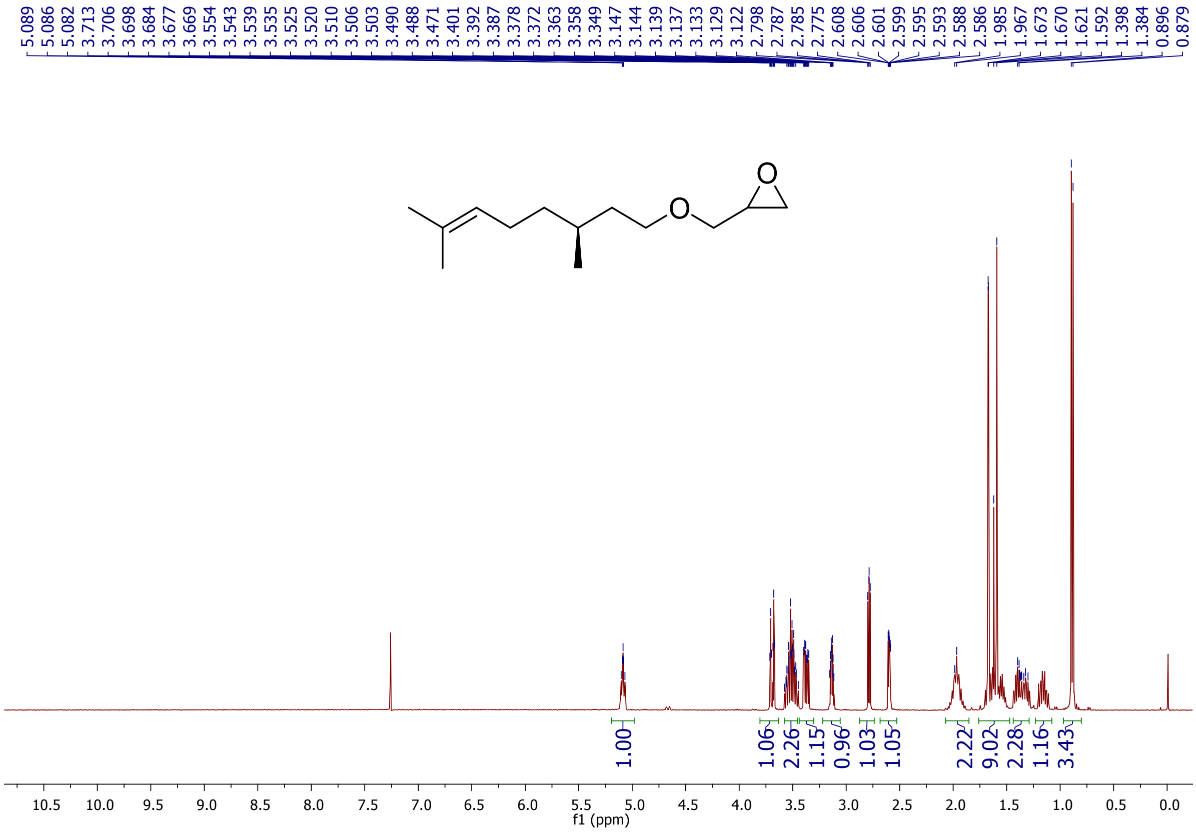

Figure S13. ^{1}H (400 MHz, $CDCl_3$) NMR spectra of **1s**

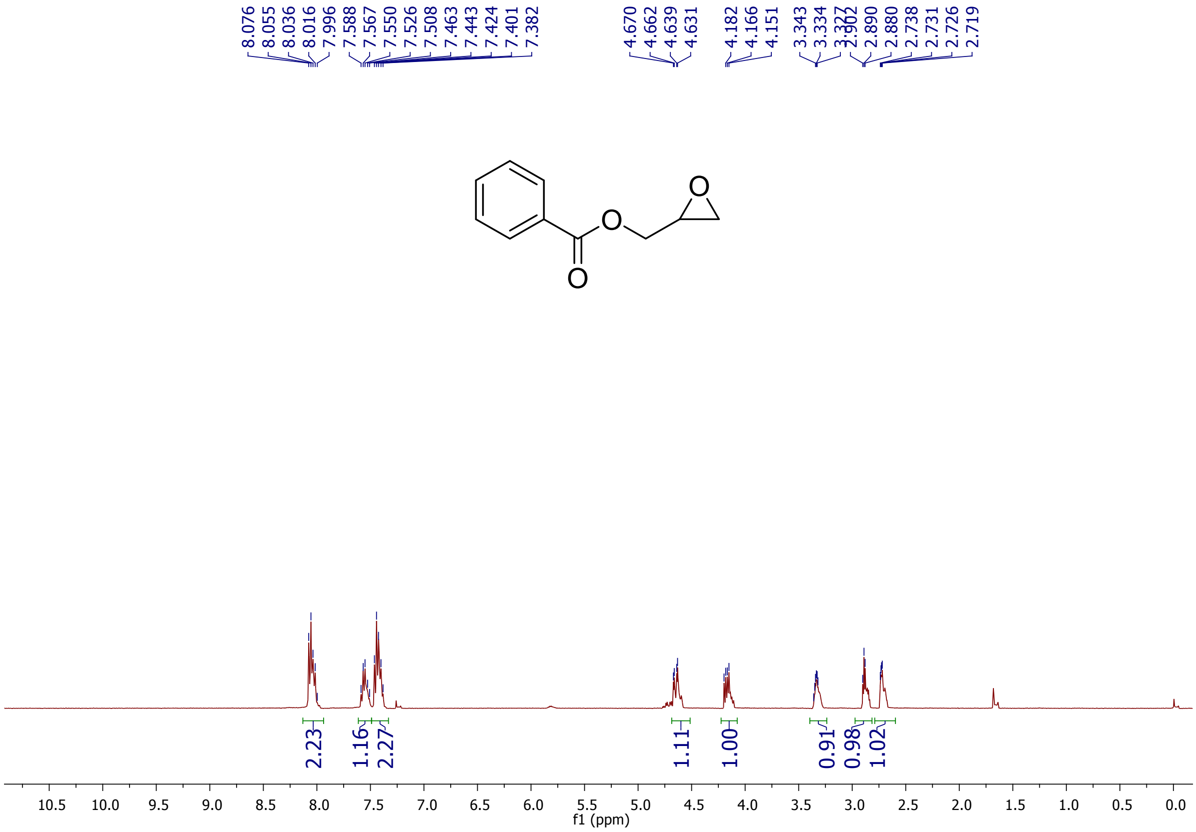

Figure S14. ^{1}H (400 MHz, $CDCl_3$) NMR spectra of **1t**

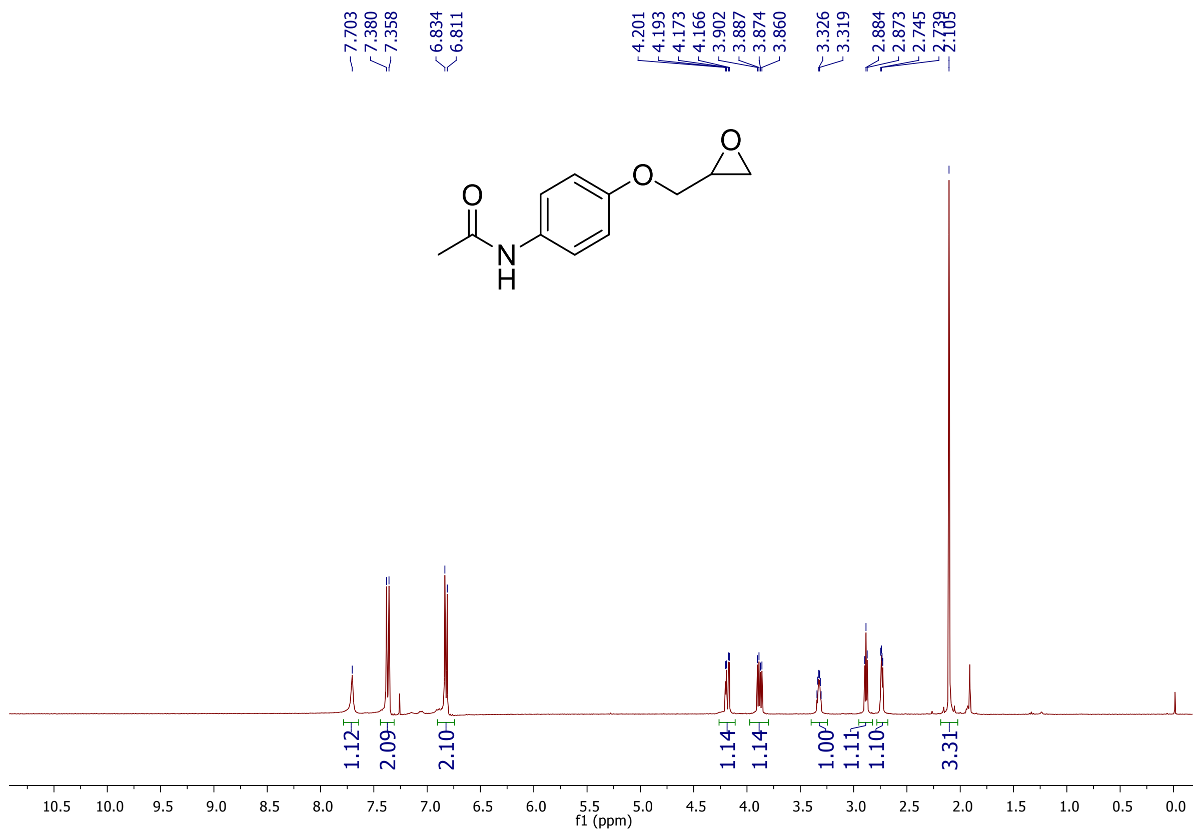

Figure S15. ^{1}H (400 MHz, $CDCl_3$) NMR spectra of **1u**

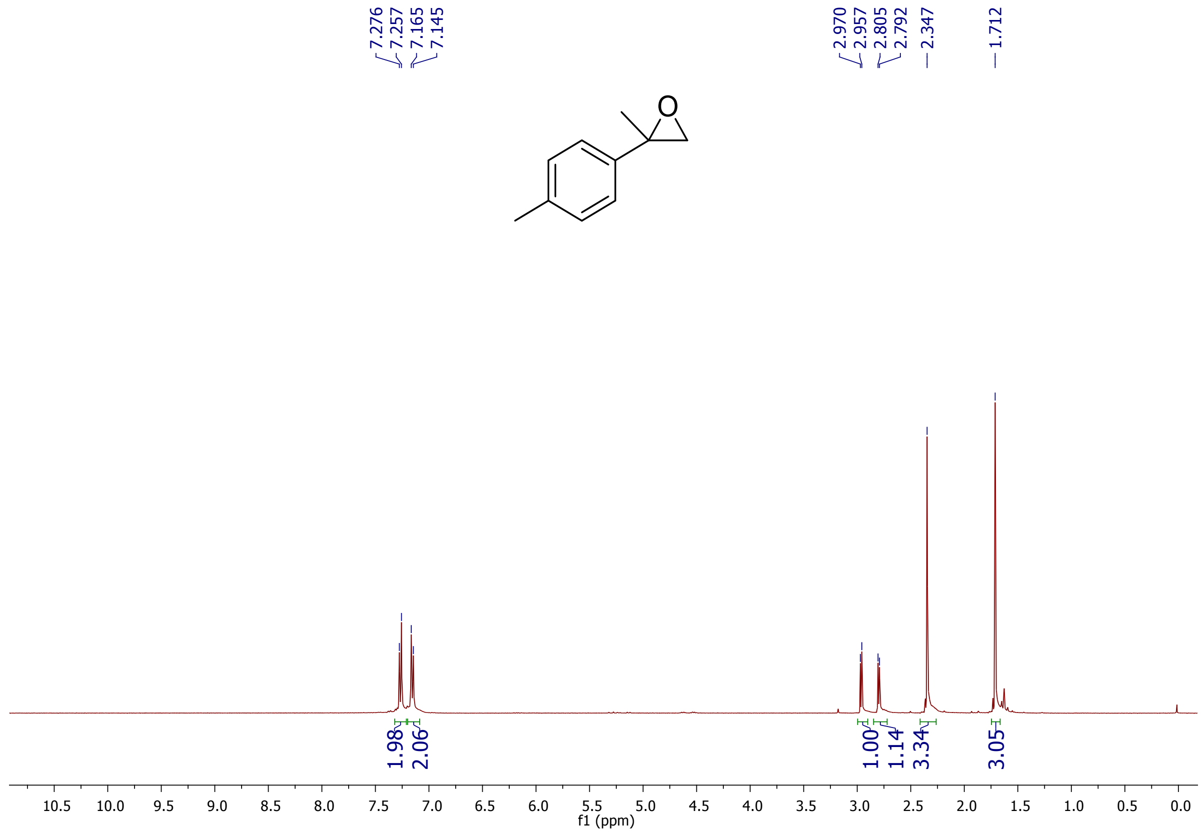

Figure S16. ^{1}H (400 MHz, $CDCl_3$) NMR spectra of **2b**

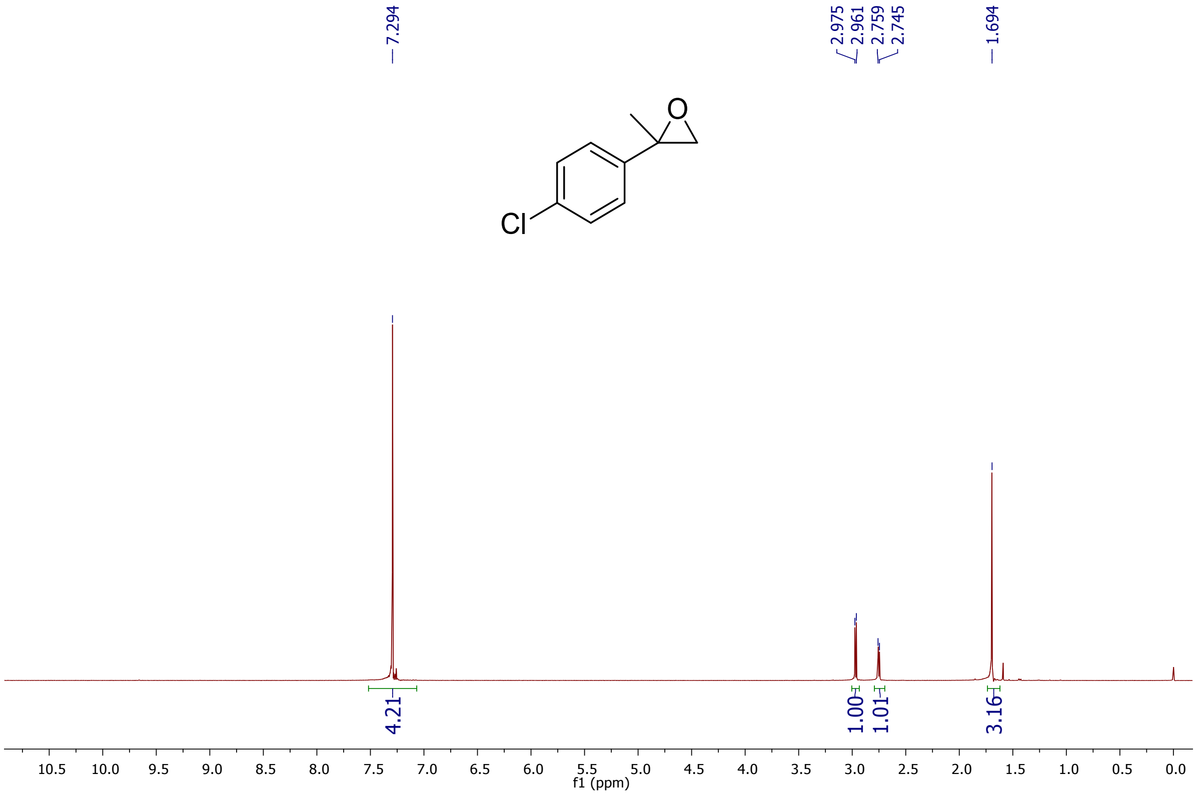

Figure S17. ^{1}H (400 MHz, $CDCl_3$) NMR spectra of **2c**

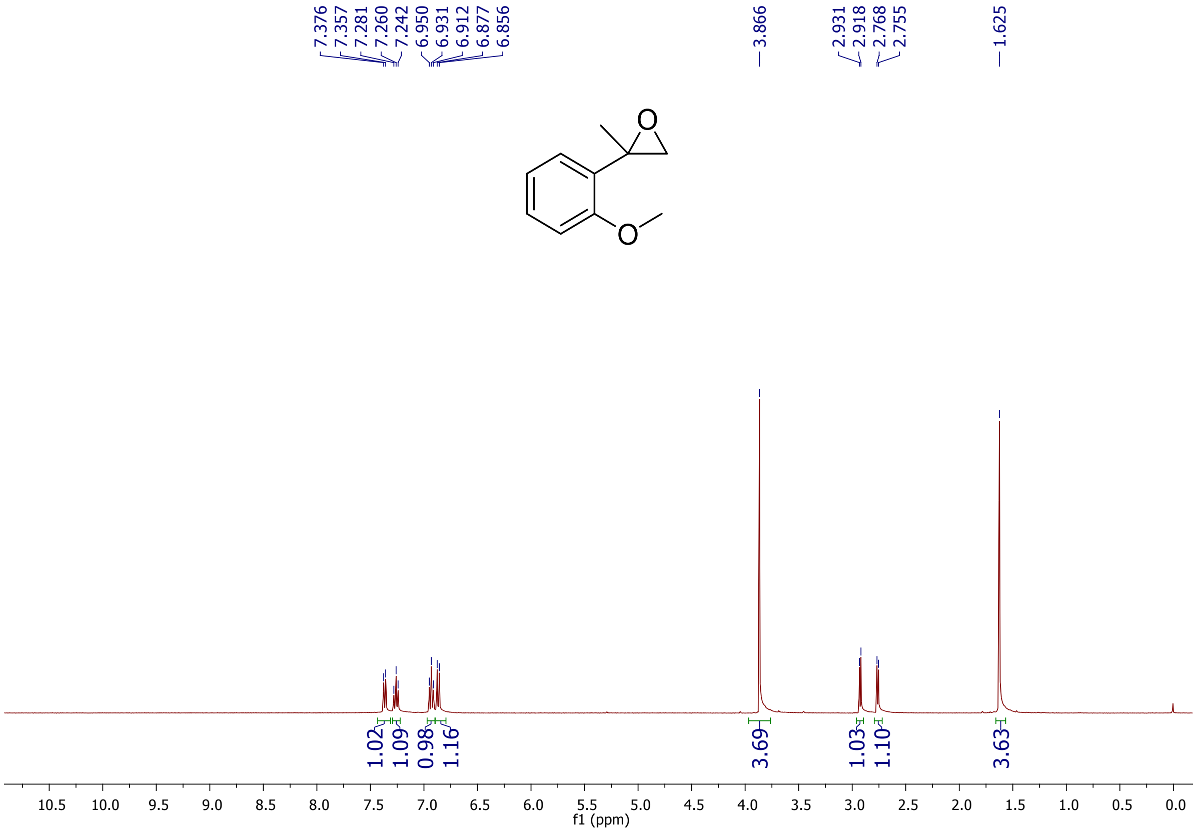

Figure S18. ^{1}H (400 MHz, $CDCl_3$) NMR spectra of **2d**

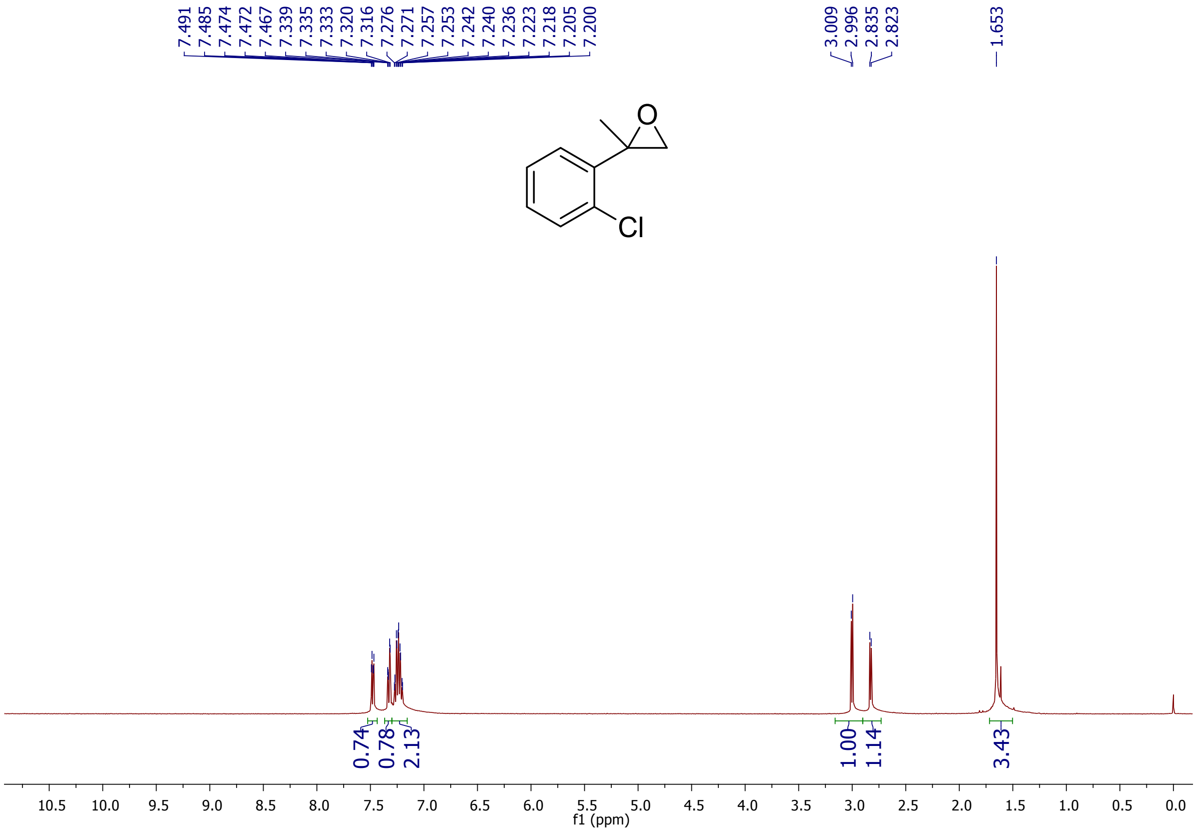

Figure S19. ^{1}H (400 MHz, $CDCl_3$) NMR spectra of **2e**

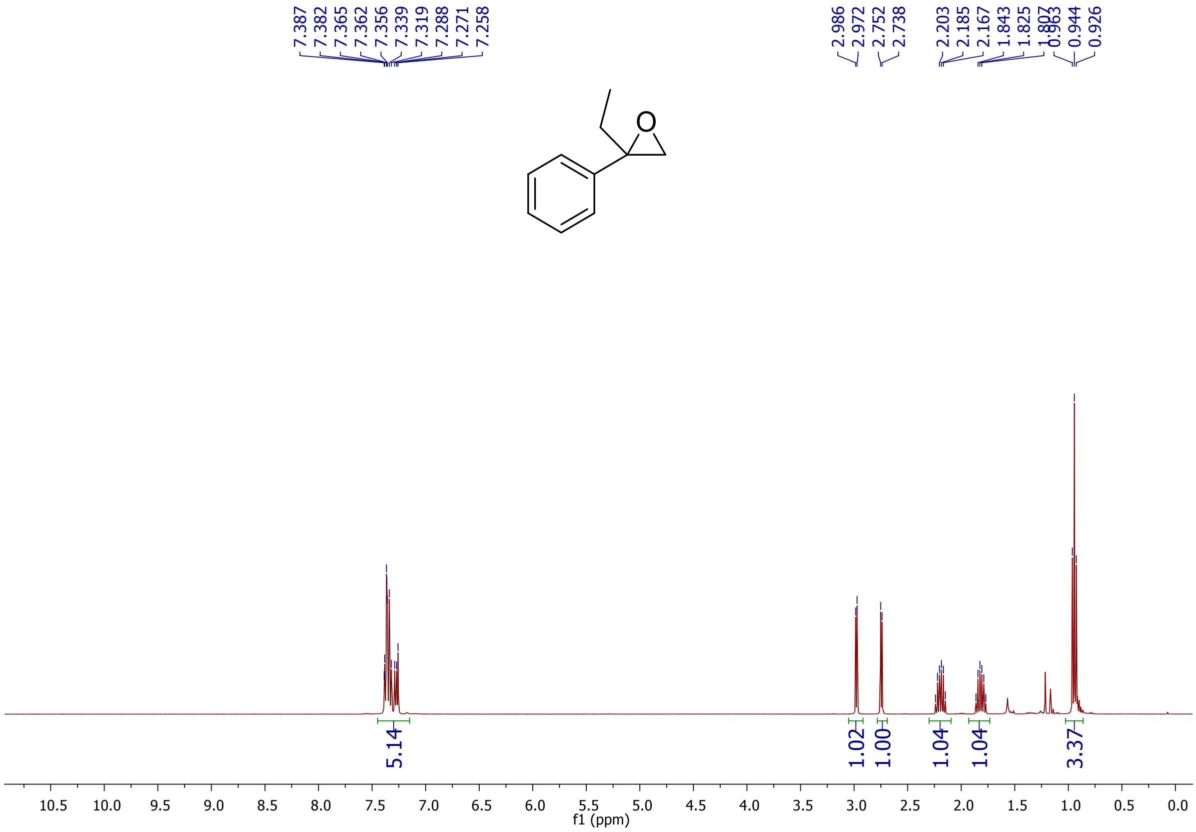

Figure S20. ^{1}H (400 MHz, $CDCl_3$) NMR spectra of **2f**

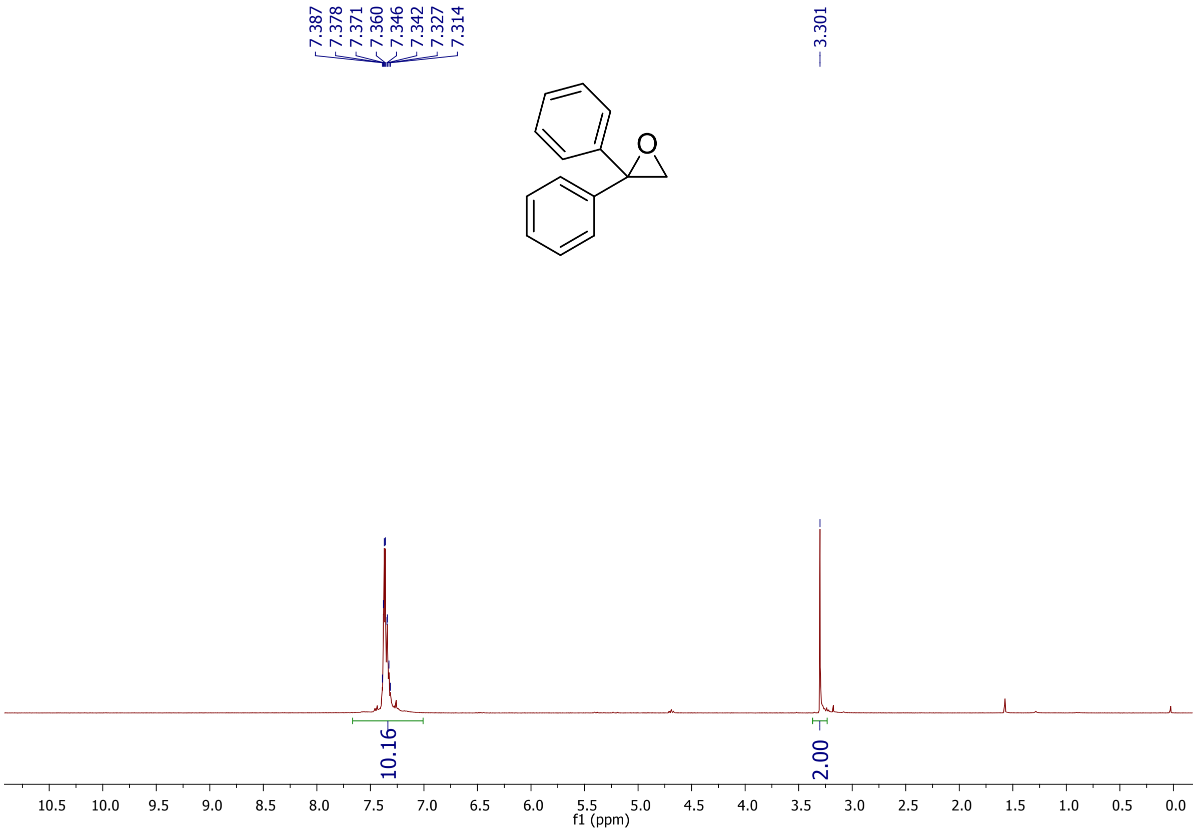

Figure S21. ^{1}H (400 MHz, $CDCl_3$) NMR spectra of **2g**

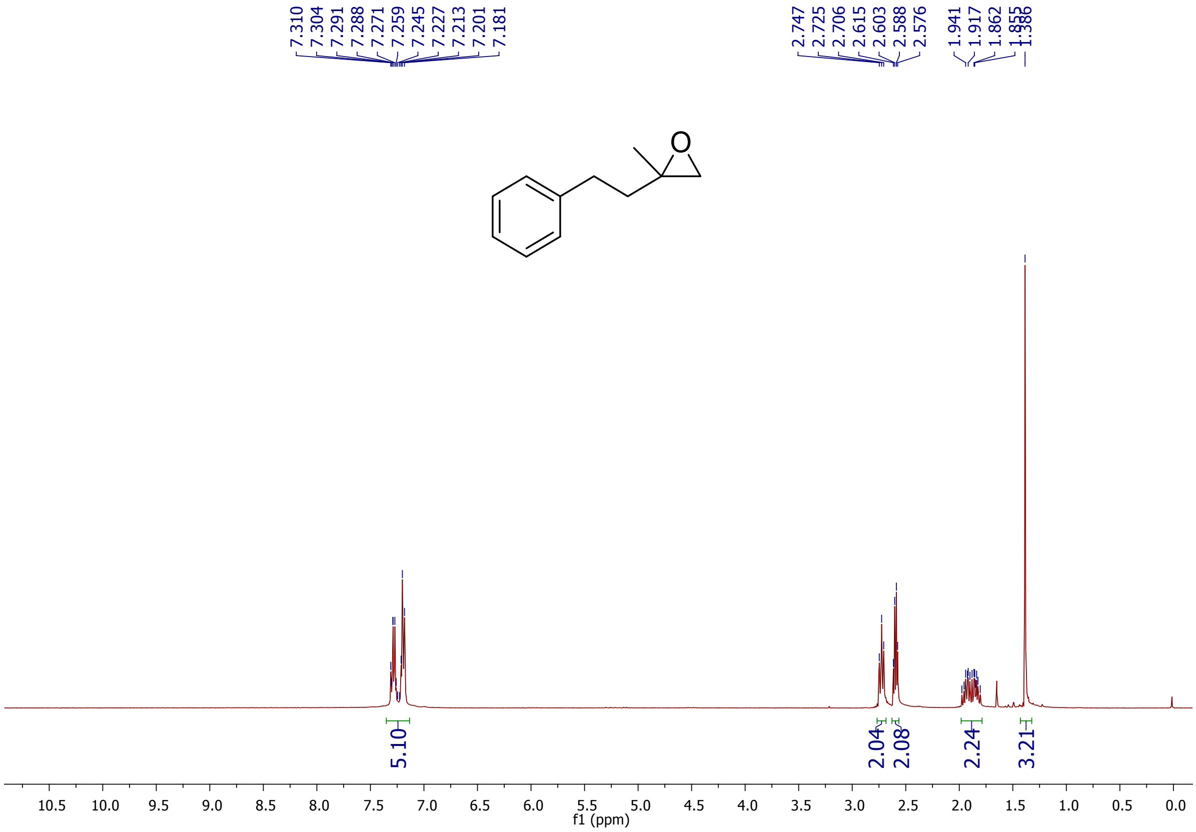

Figure S22. ^{1}H (400 MHz, $CDCl_3$) NMR spectra of **2h**

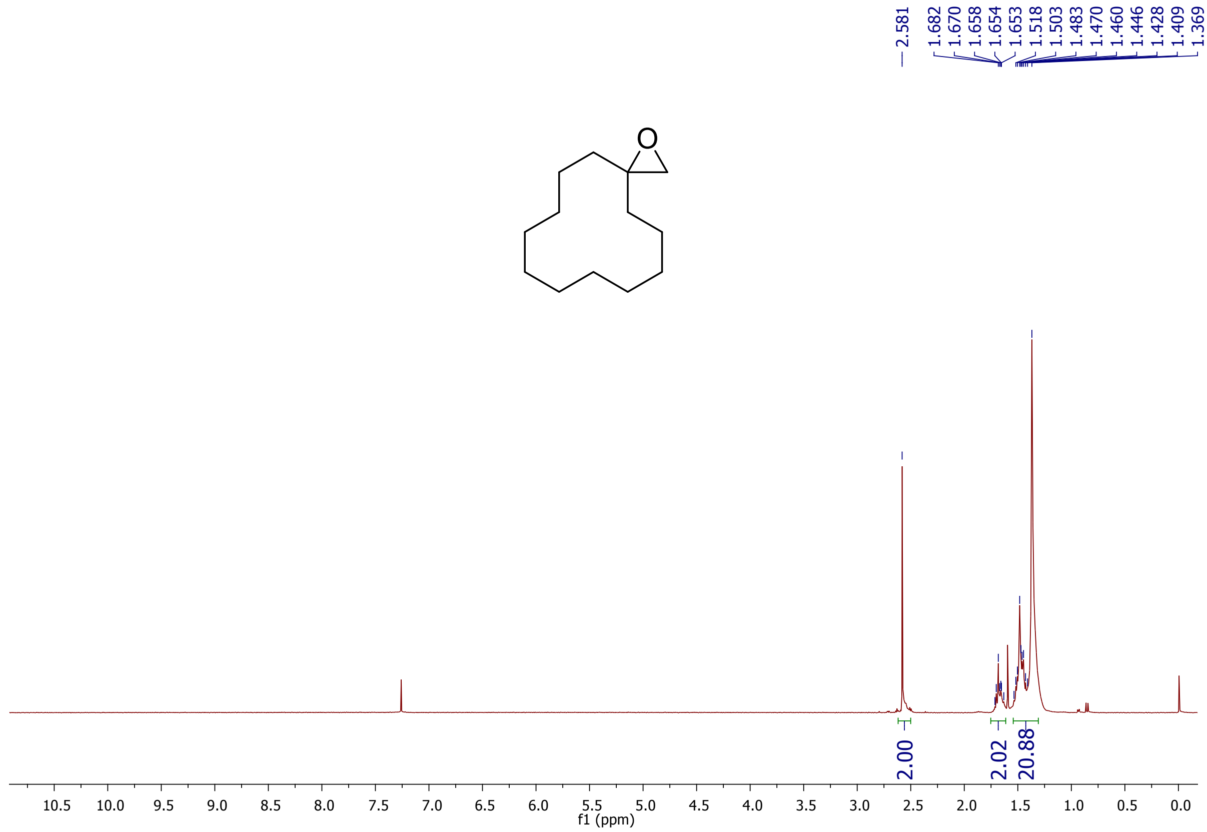

Figure S23. ^{1}H (400 MHz, $CDCl_3$) NMR spectra of **2i**

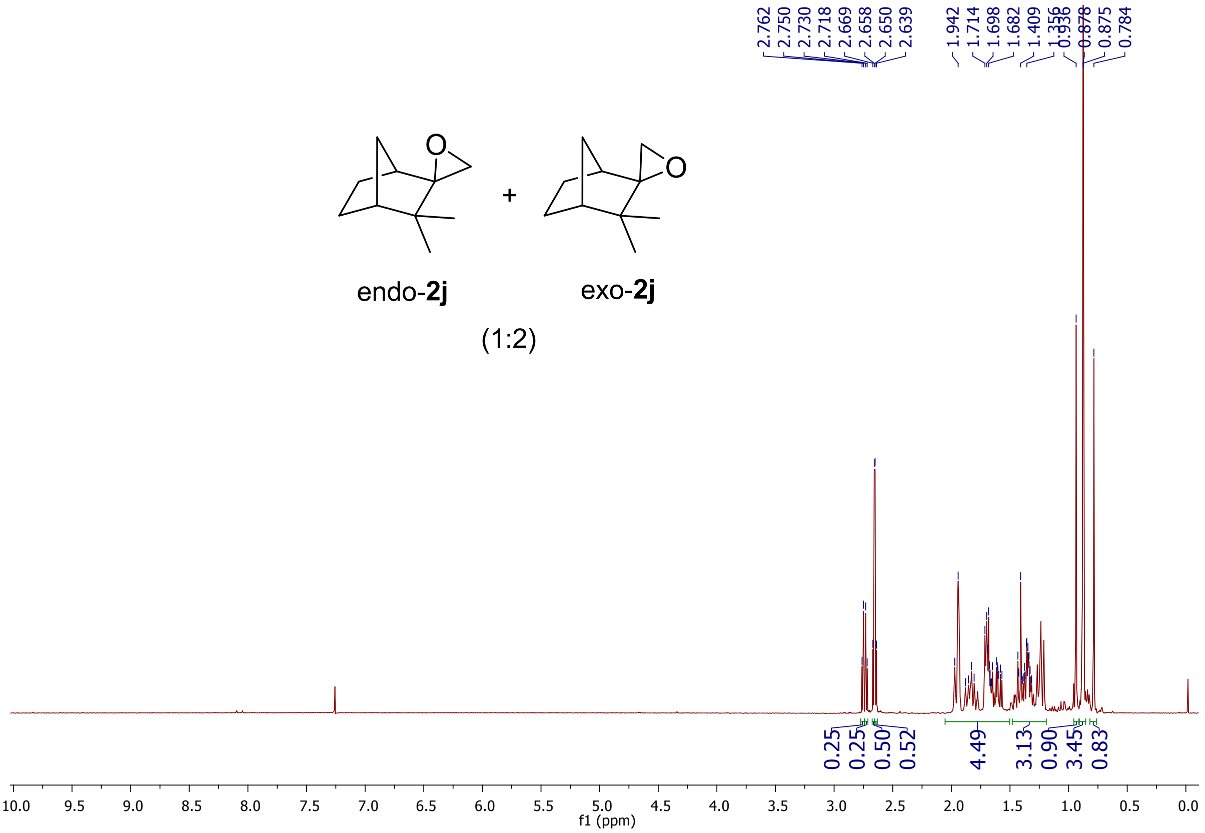

Figure S24. ^{1}H (400 MHz, $CDCl_3$) NMR spectra of **2j**

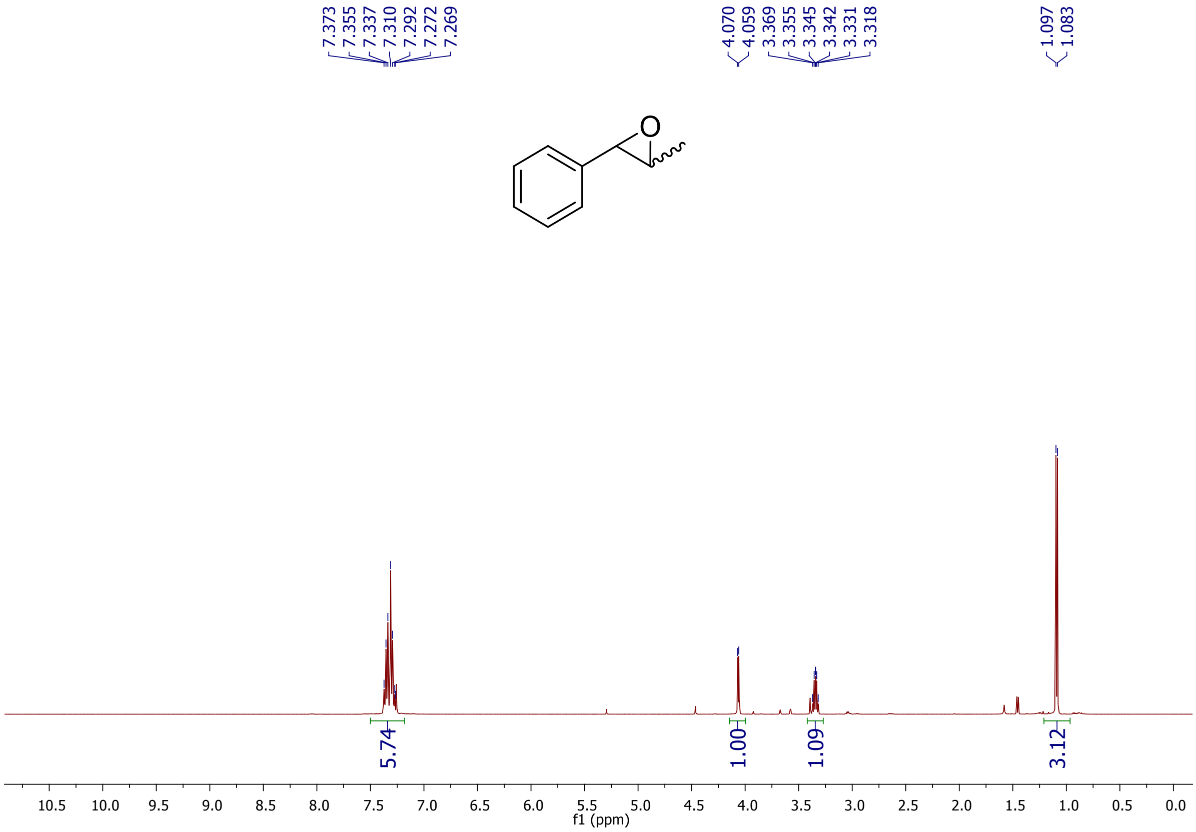

Figure S25. ^{1}H (400 MHz, $CDCl_3$) NMR spectra of **3a**

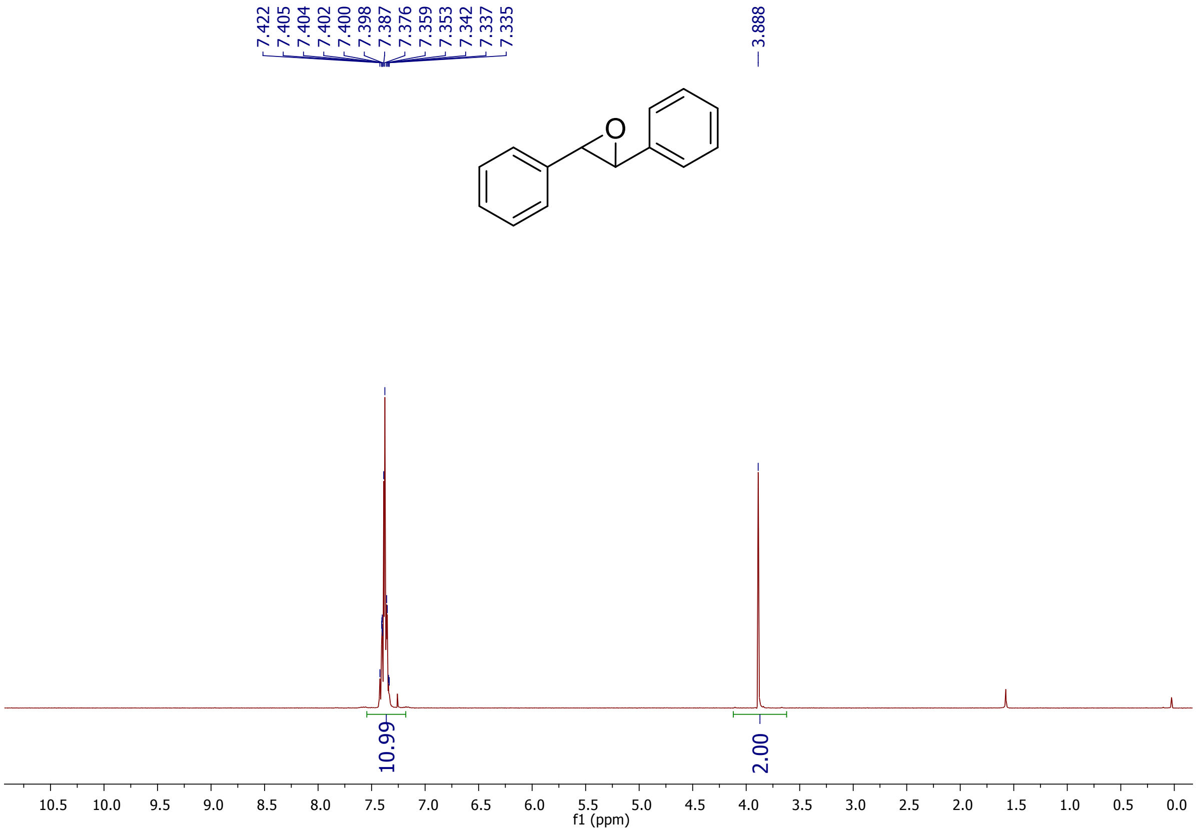

Figure S26. ^{1}H (400 MHz, $CDCl_3$) NMR spectra of **3b**

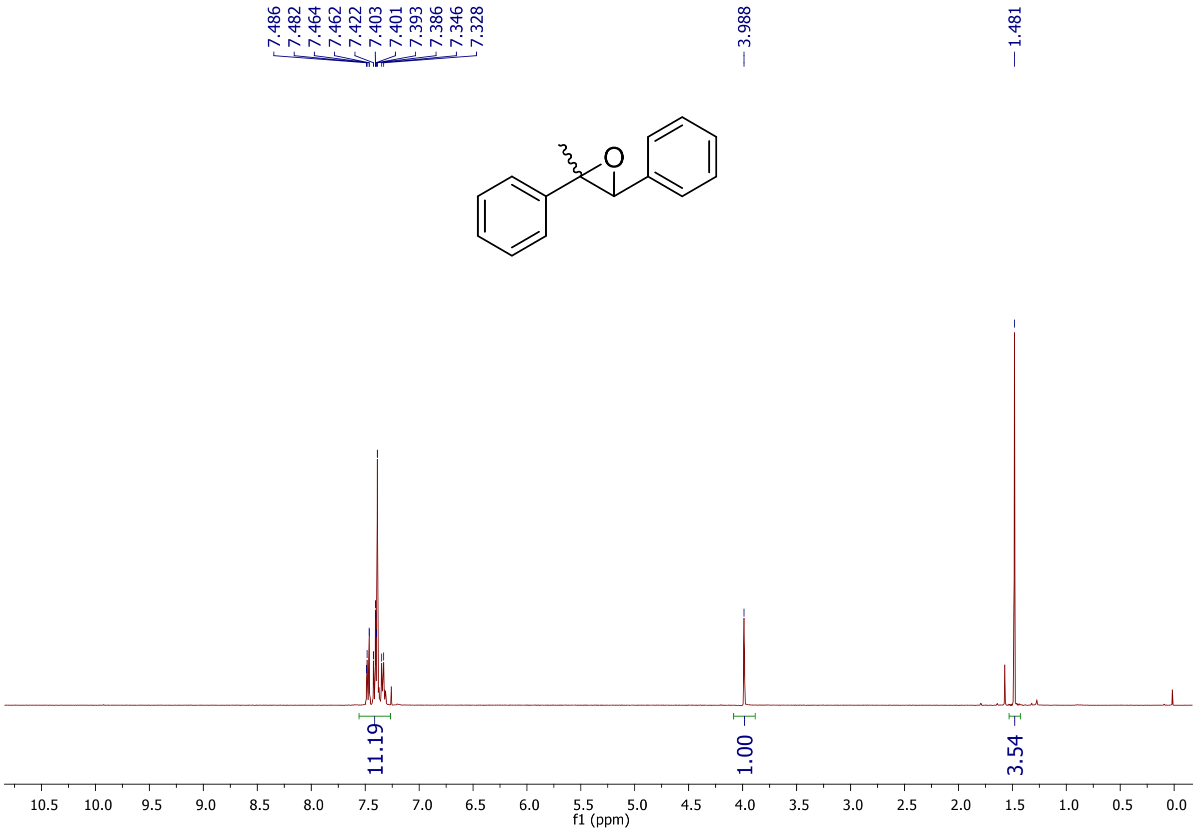

Figure S27. ^{1}H (400 MHz, $CDCl_3$) NMR spectra of **3c**

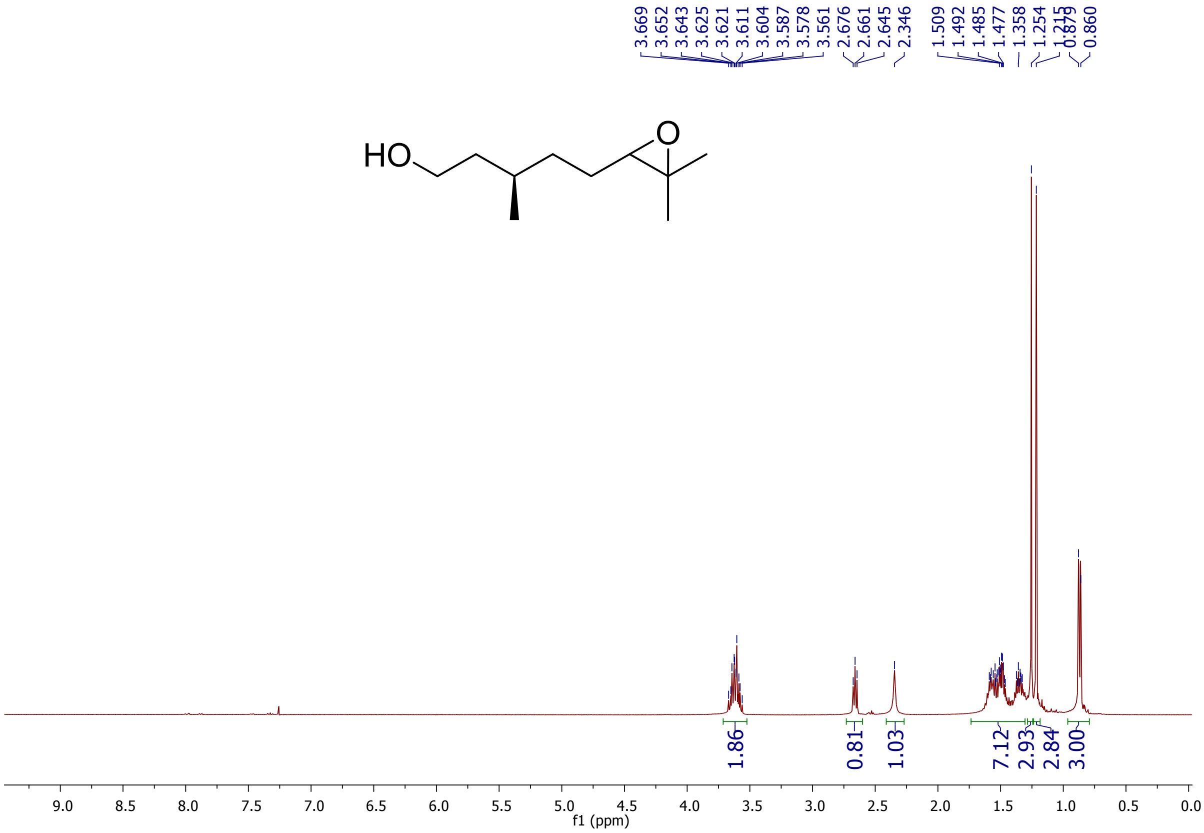

Figure S28. ^{1}H (400 MHz, $CDCl_3$) NMR spectra of **3e**

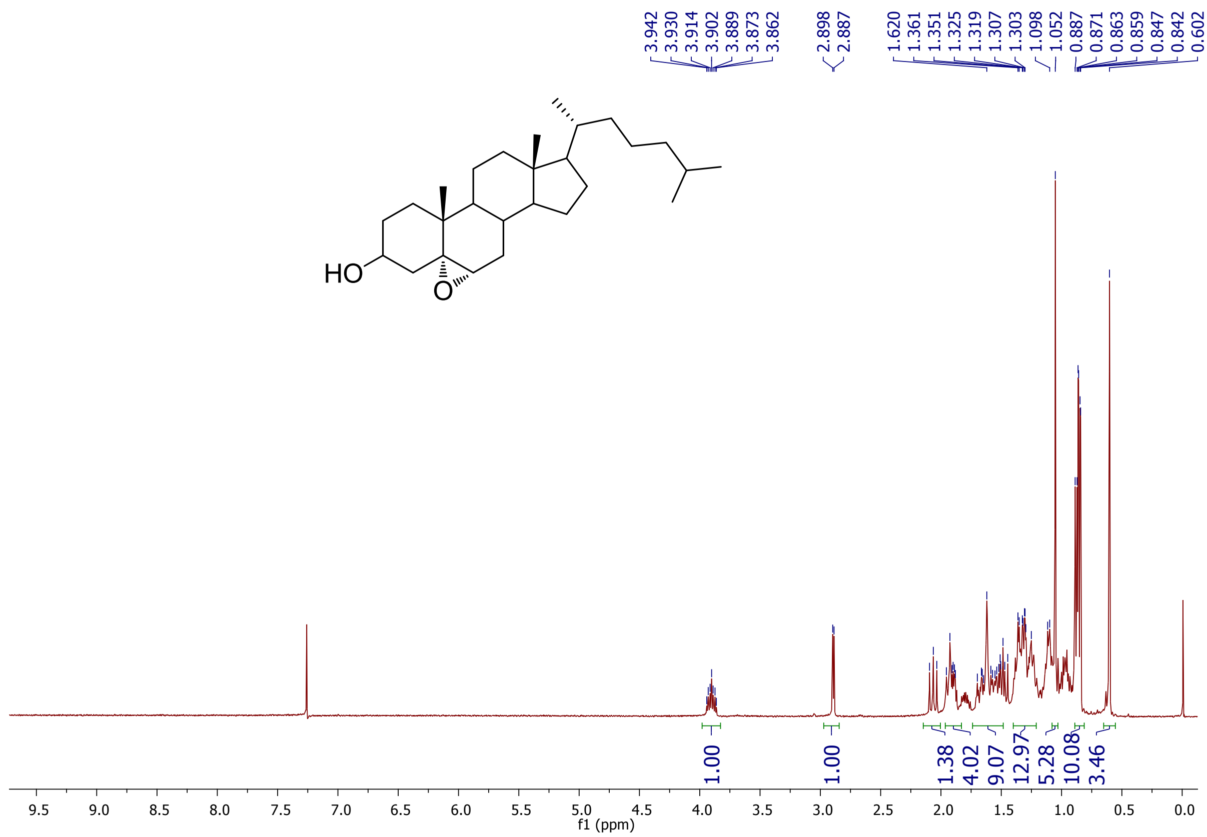

Figure S29. ^{1}H (400 MHz, $CDCl_3$) NMR spectra of **3f**

7. Traces of ^{1}H and ^{13}C NMR spectra of isolated products

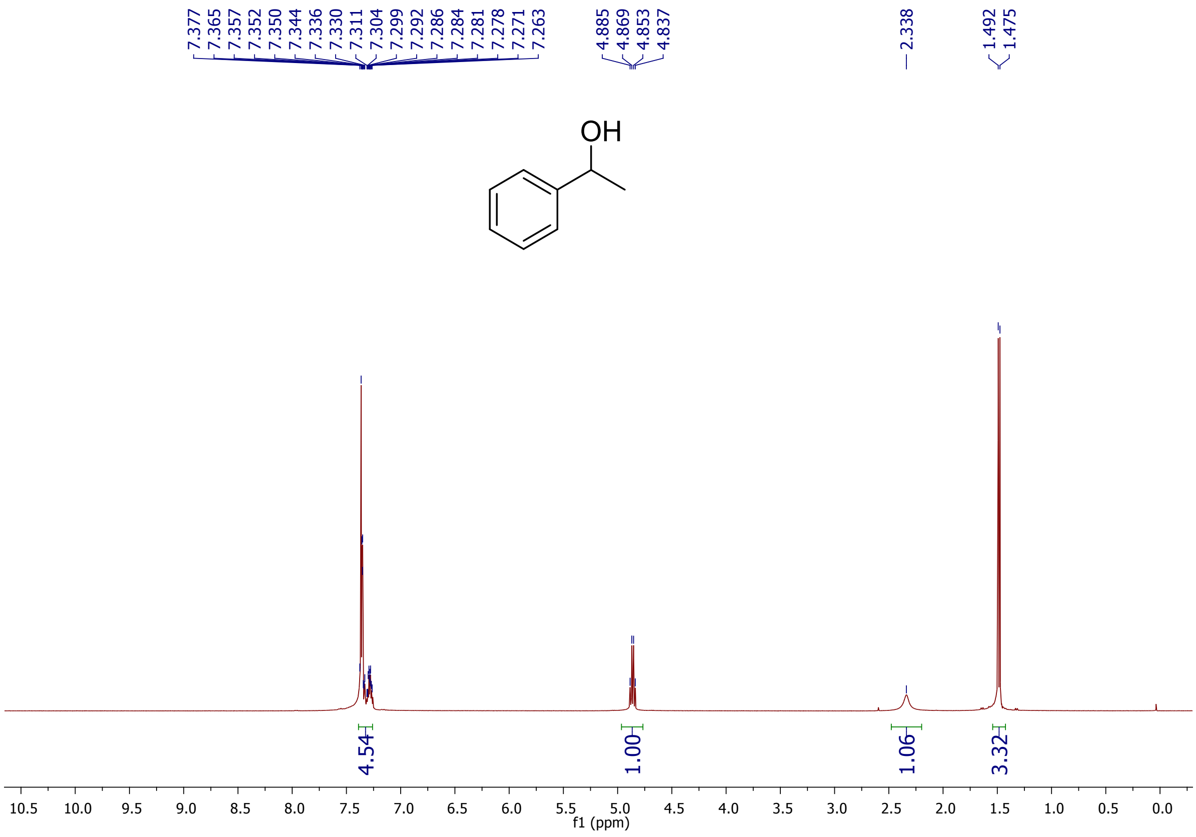

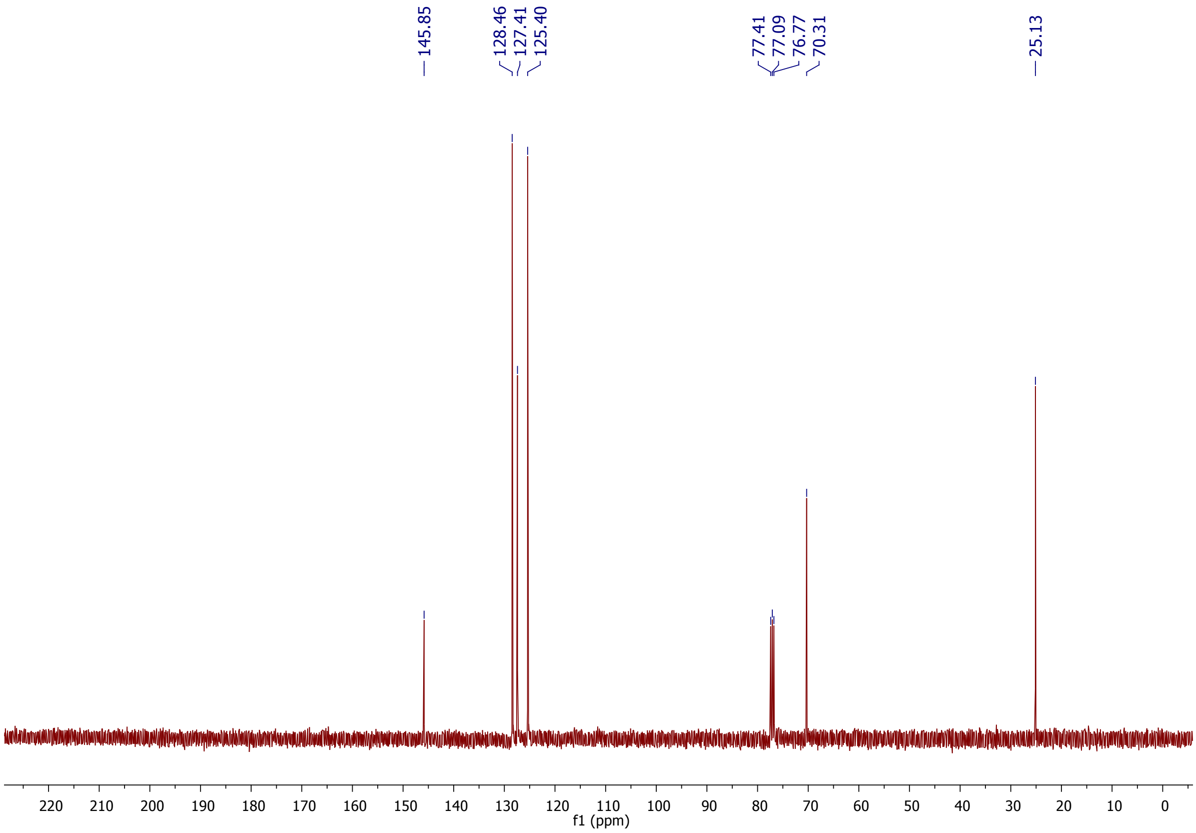

Figure S30. ^{1}H (400 MHz, $CDCl_3$) and $^{13}C\{^1H\}$ (100.6 MHz, $CDCl_3$) NMR spectra of **4a**

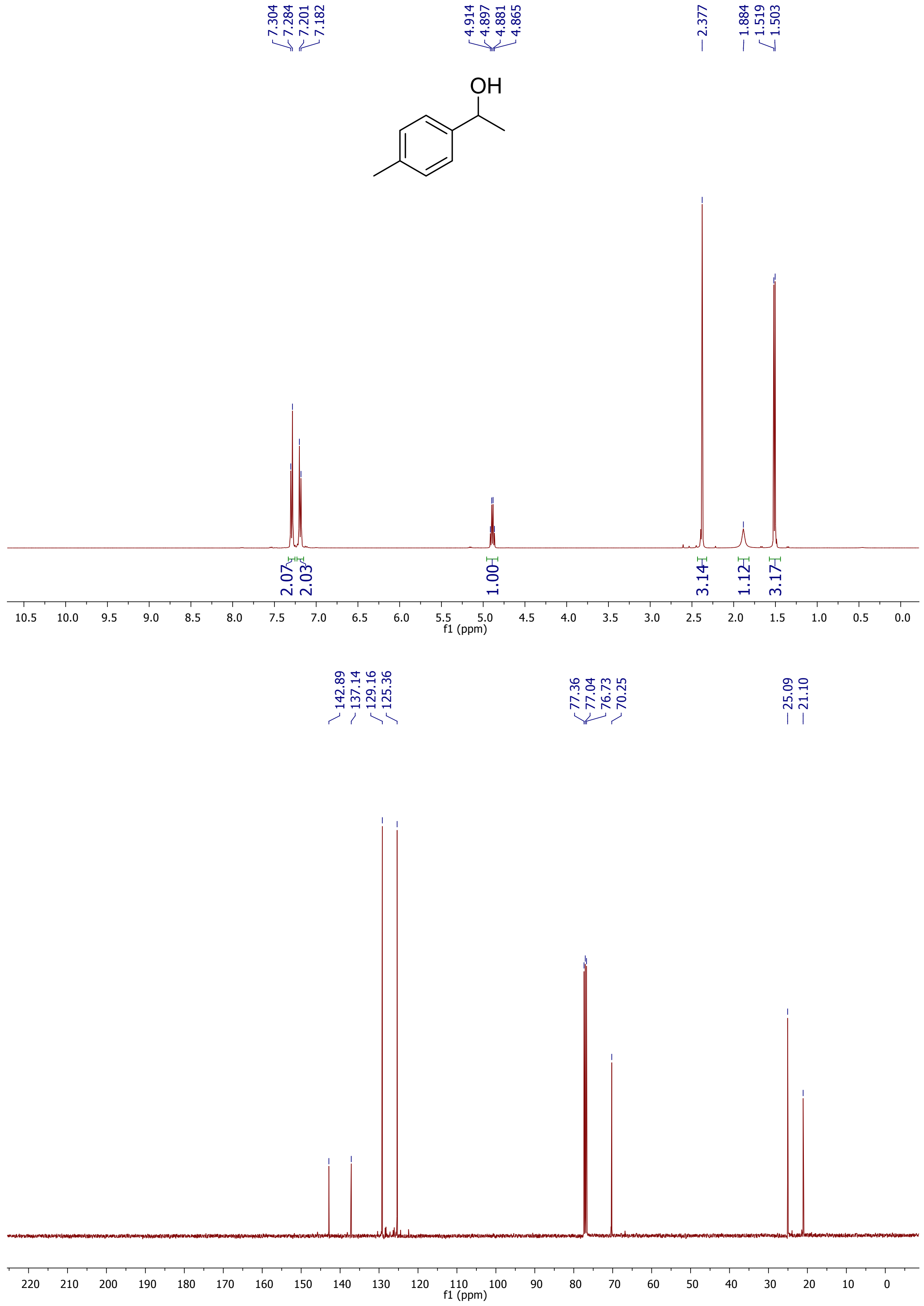

Figure S31. ^{1}H (400 MHz, $CDCl_3$) and $^{13}C\{^{1}H\}$ (100.6 MHz, $CDCl_3$) NMR spectra of **4b**

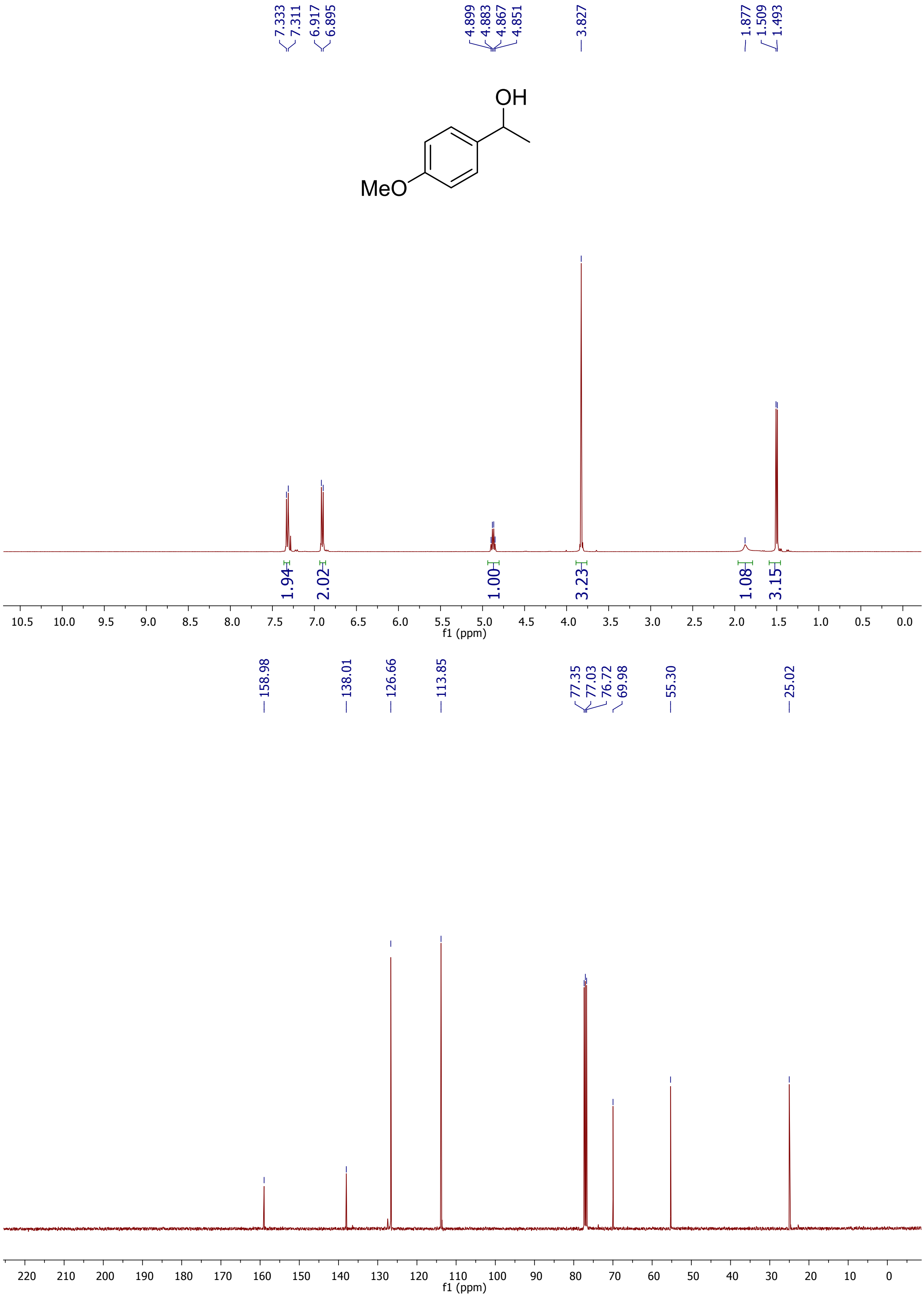

Figure S32. ^{1}H (400 MHz, $CDCl_3$) and $^{13}C\{^{1}H\}$ (100.6 MHz, $CDCl_3$) NMR spectra of **4c**

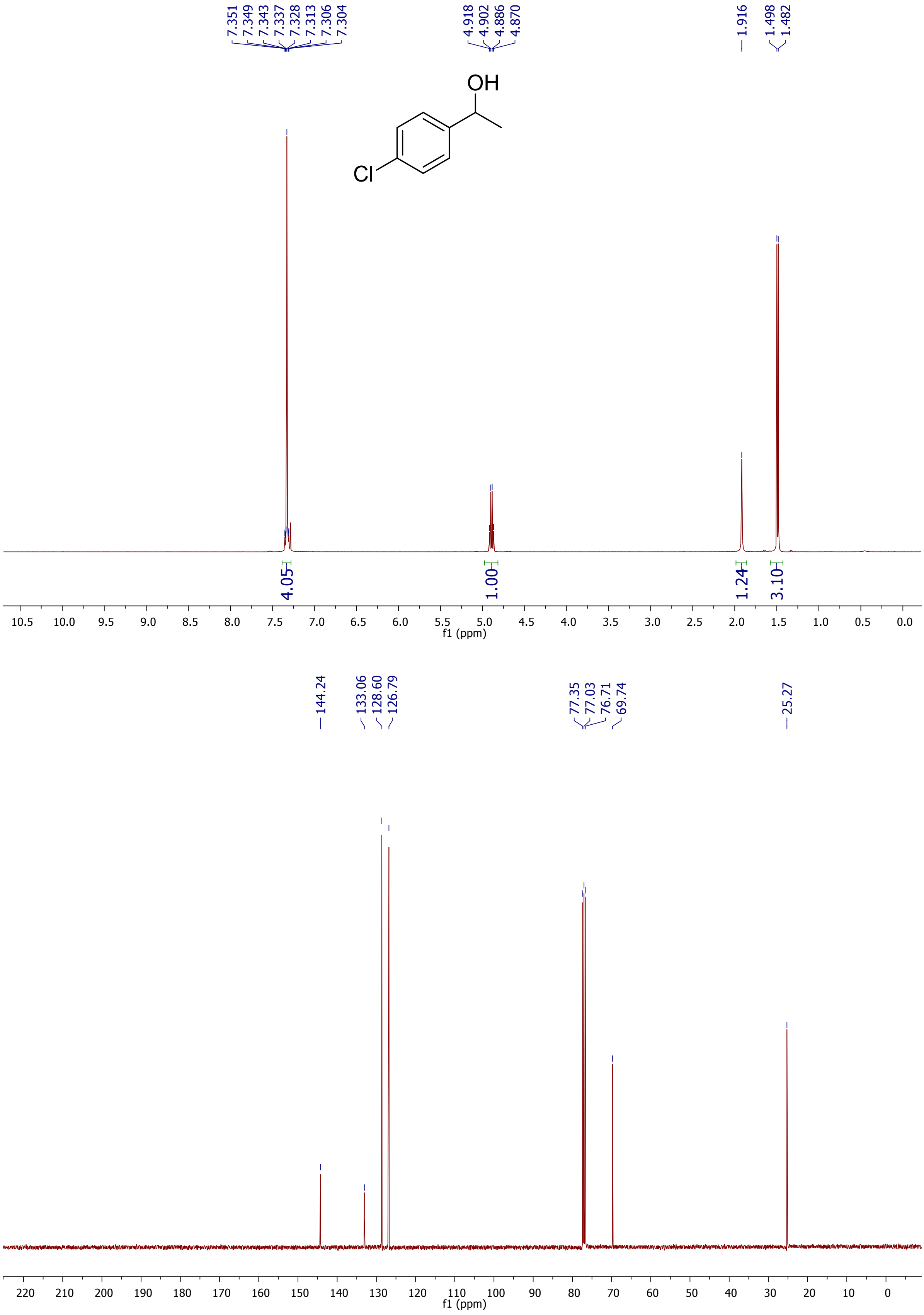

Figure S33. ^{1}H (400 MHz, $CDCl_3$) and $^{13}C\{^{1}H\}$ (100.6 MHz, $CDCl_3$) NMR spectra of **4d**

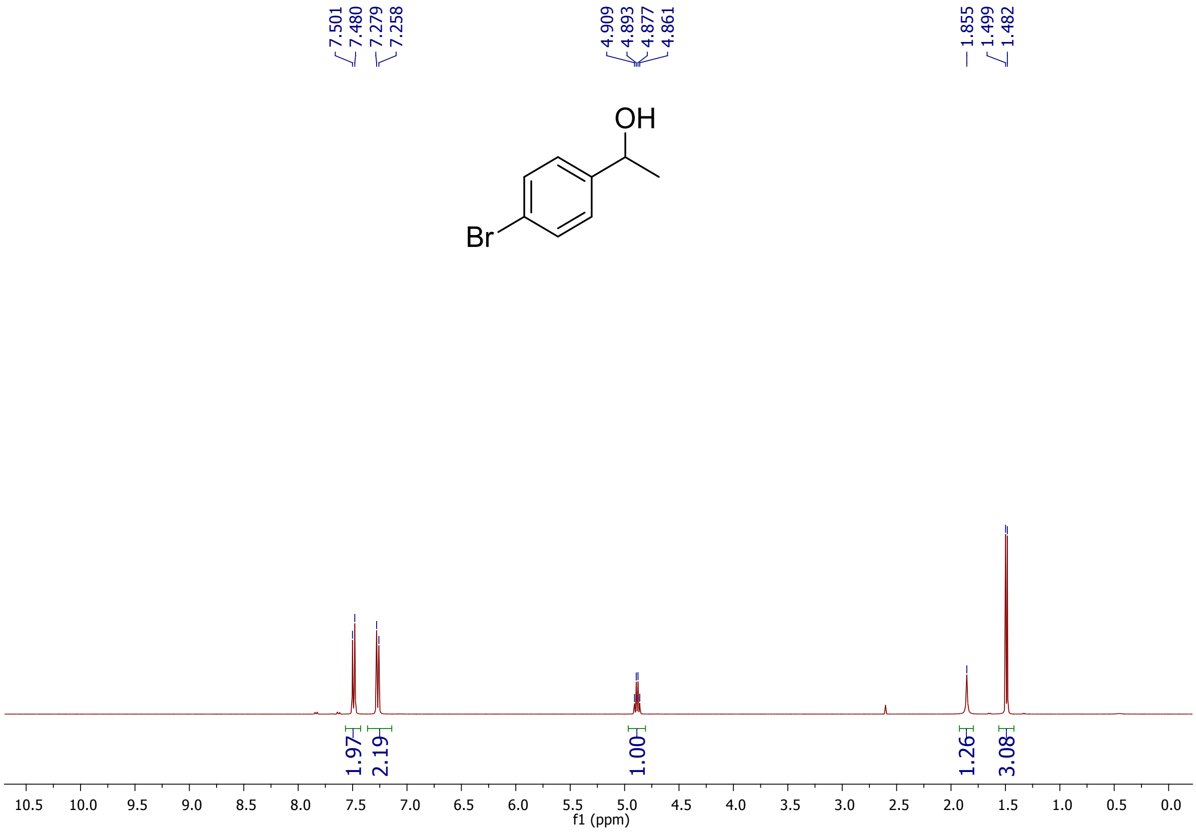

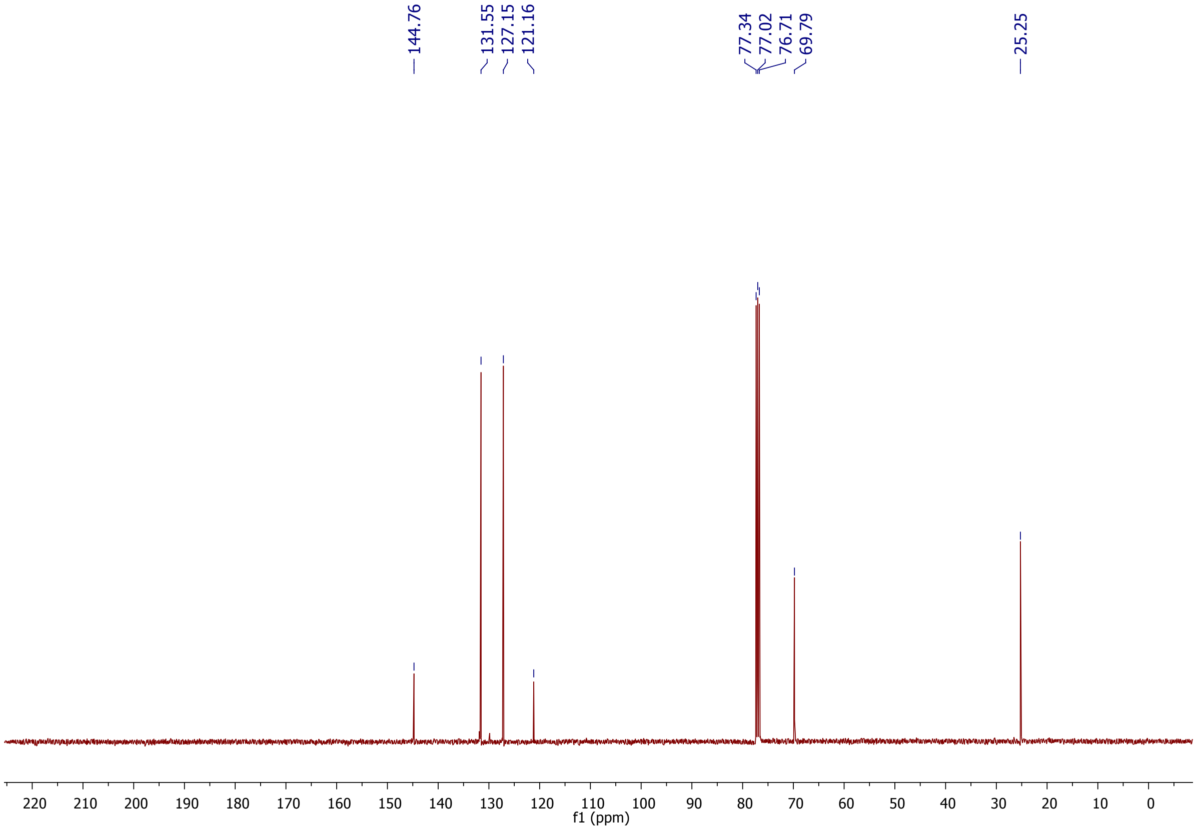

Figure S34. ^{1}H (400 MHz, $CDCl_3$) and $^{13}C\{^{1}H\}$ (100.6 MHz, $CDCl_3$) NMR spectra of **4e**

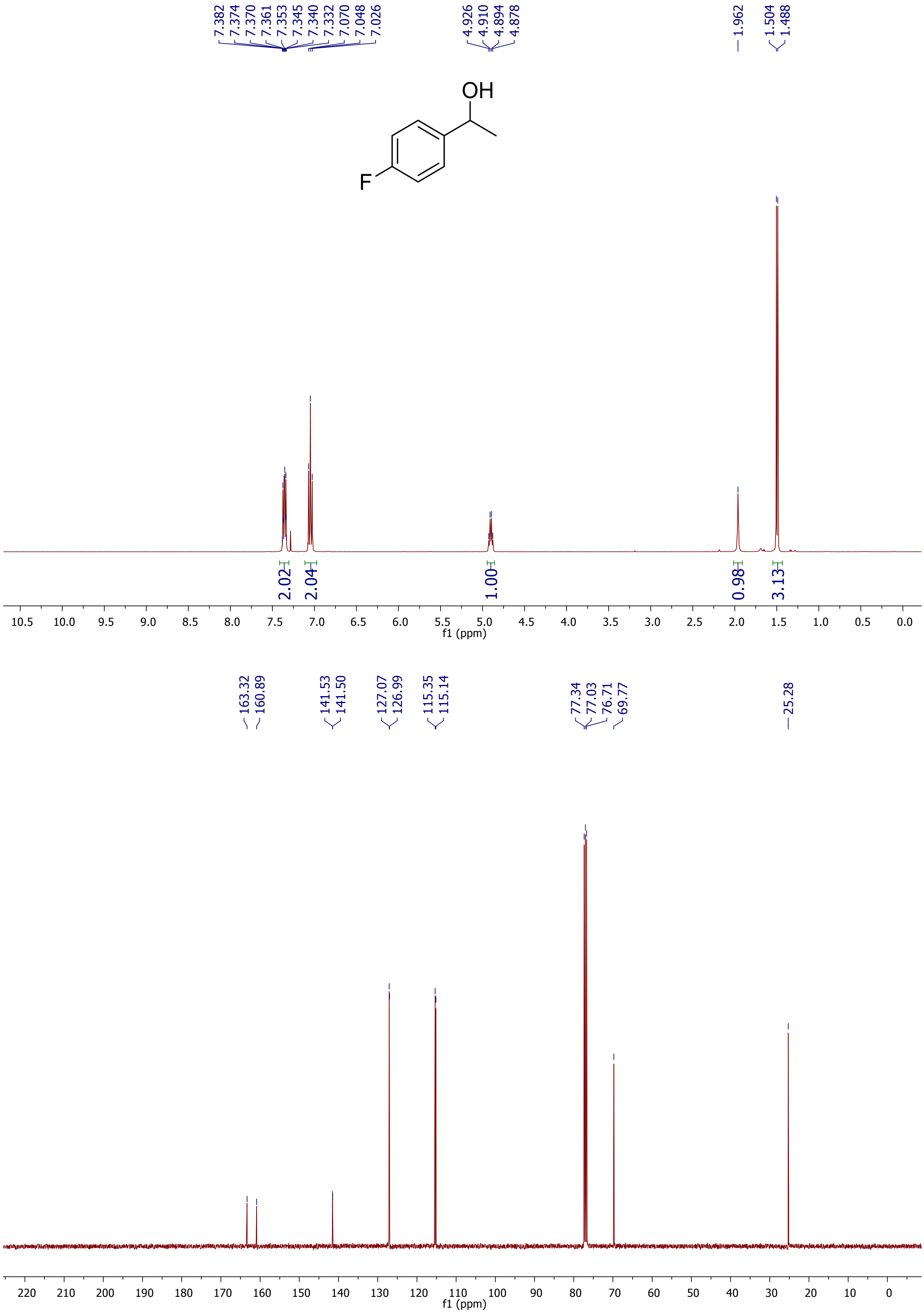

Figure S35. ^{1}H (400 MHz, $CDCl_3$) and $^{13}C\{^{1}H\}$ (100.6 MHz, $CDCl_3$) NMR spectra of **4f**

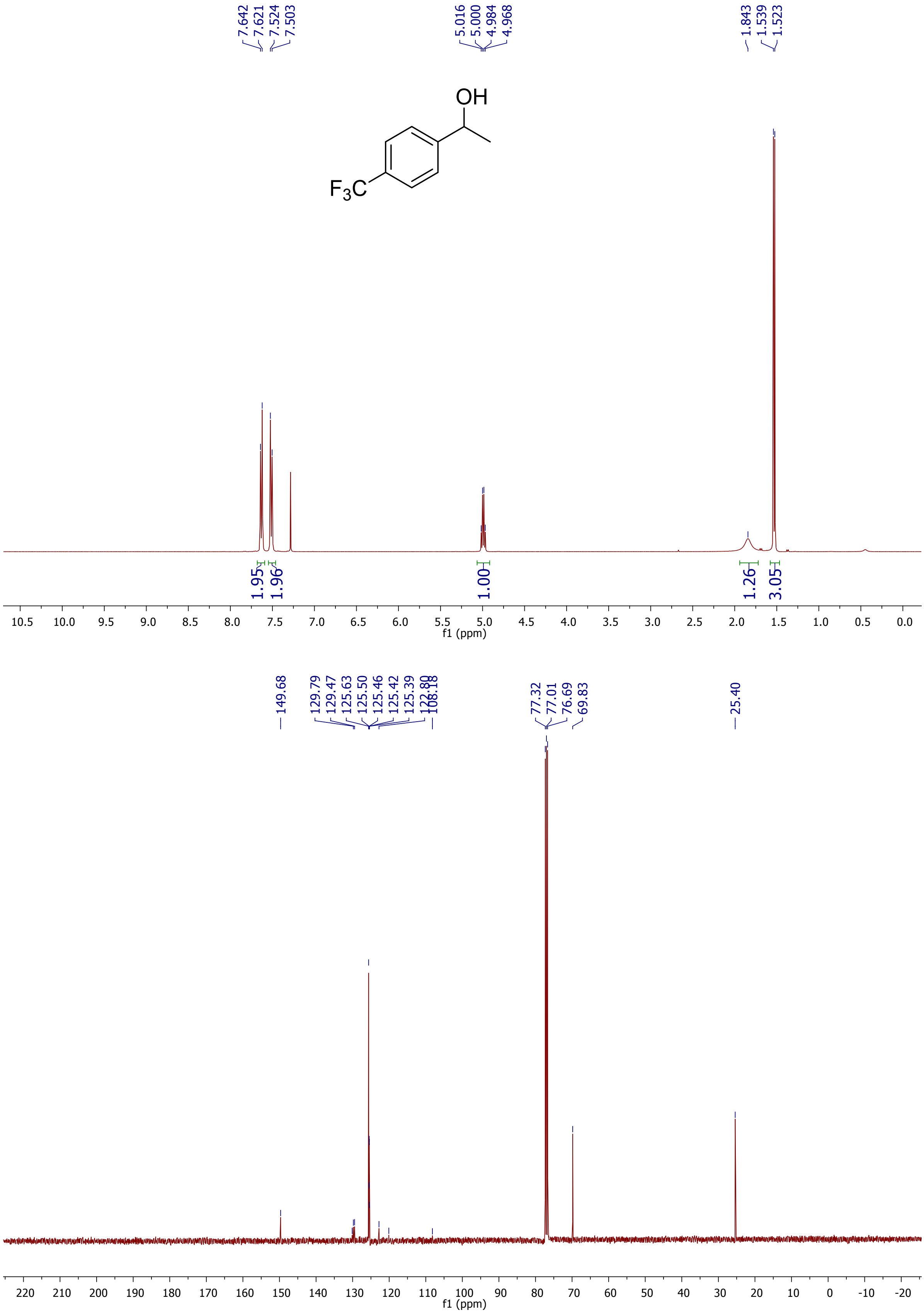

Figure S36. ^{1}H (400 MHz, $CDCl_3$) and $^{13}C\{^{1}H\}$ (100.6 MHz, $CDCl_3$) NMR spectra of **4g**

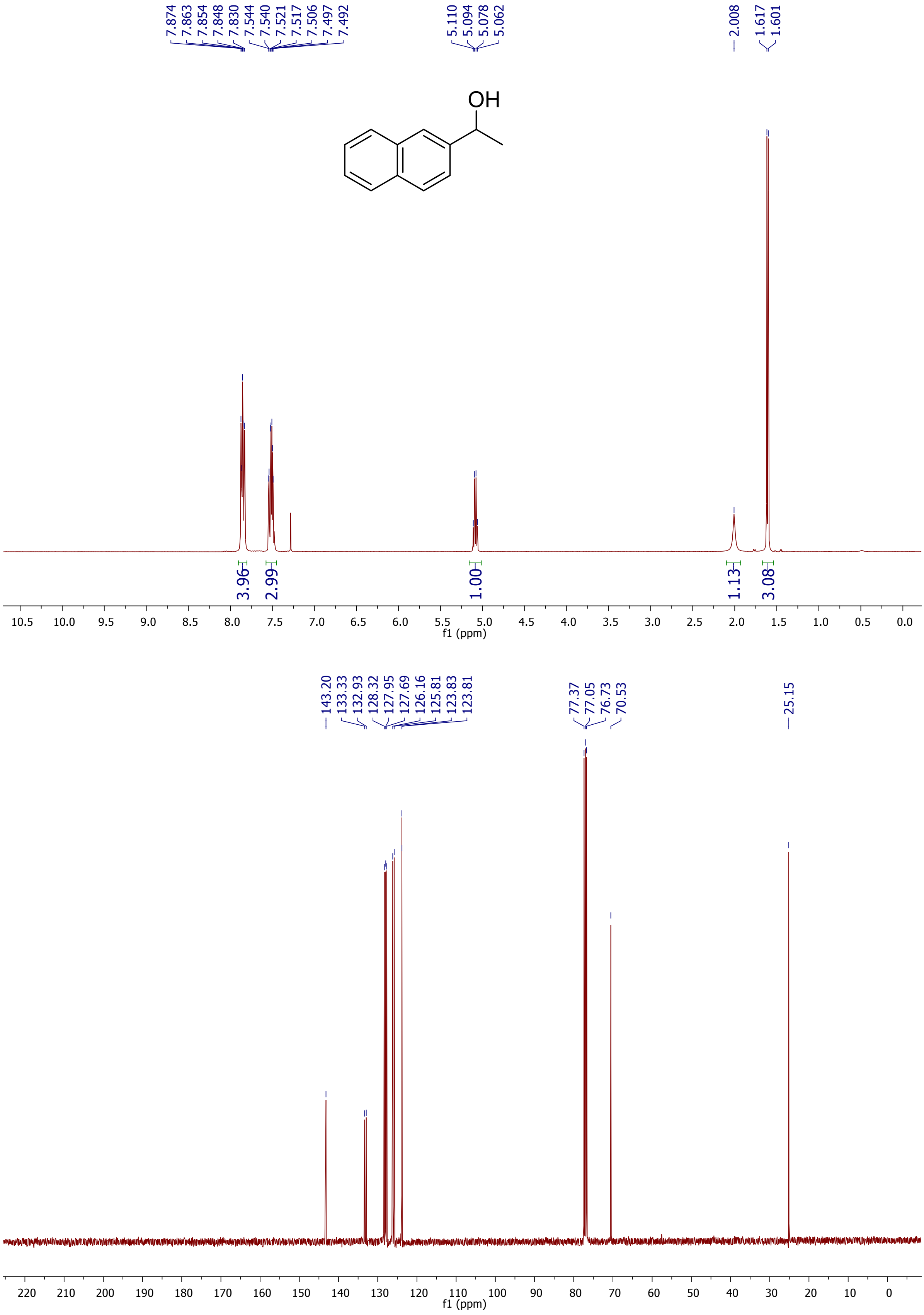

Figure S37. ^{1}H (400 MHz, $CDCl_3$) and $^{13}C\{^{1}H\}$ (100.6 MHz, $CDCl_3$) NMR spectra of **4i**

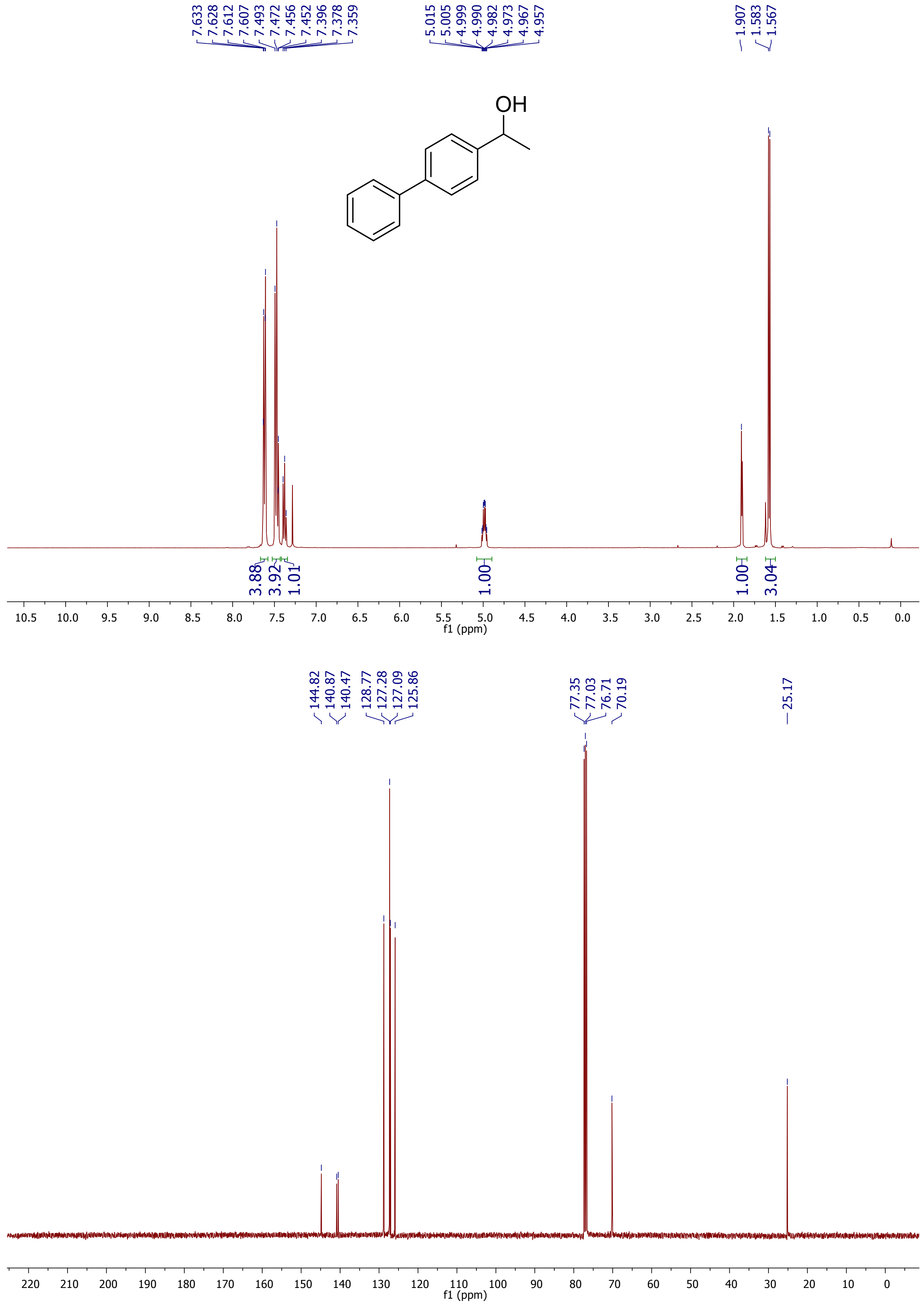

Figure S38. ^{1}H (400 MHz, $CDCl_3$) and $^{13}C\{^{1}H\}$ (100.6 MHz, $CDCl_3$) NMR spectra of **4j**

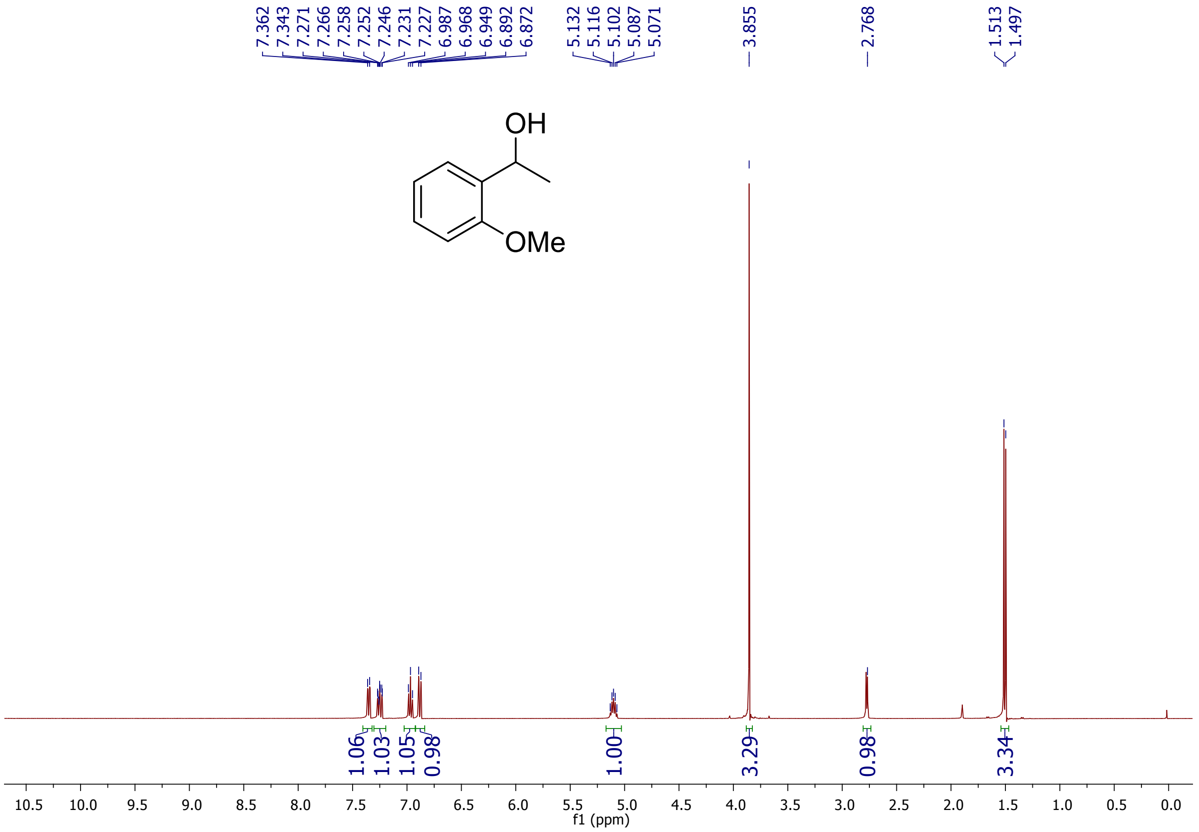

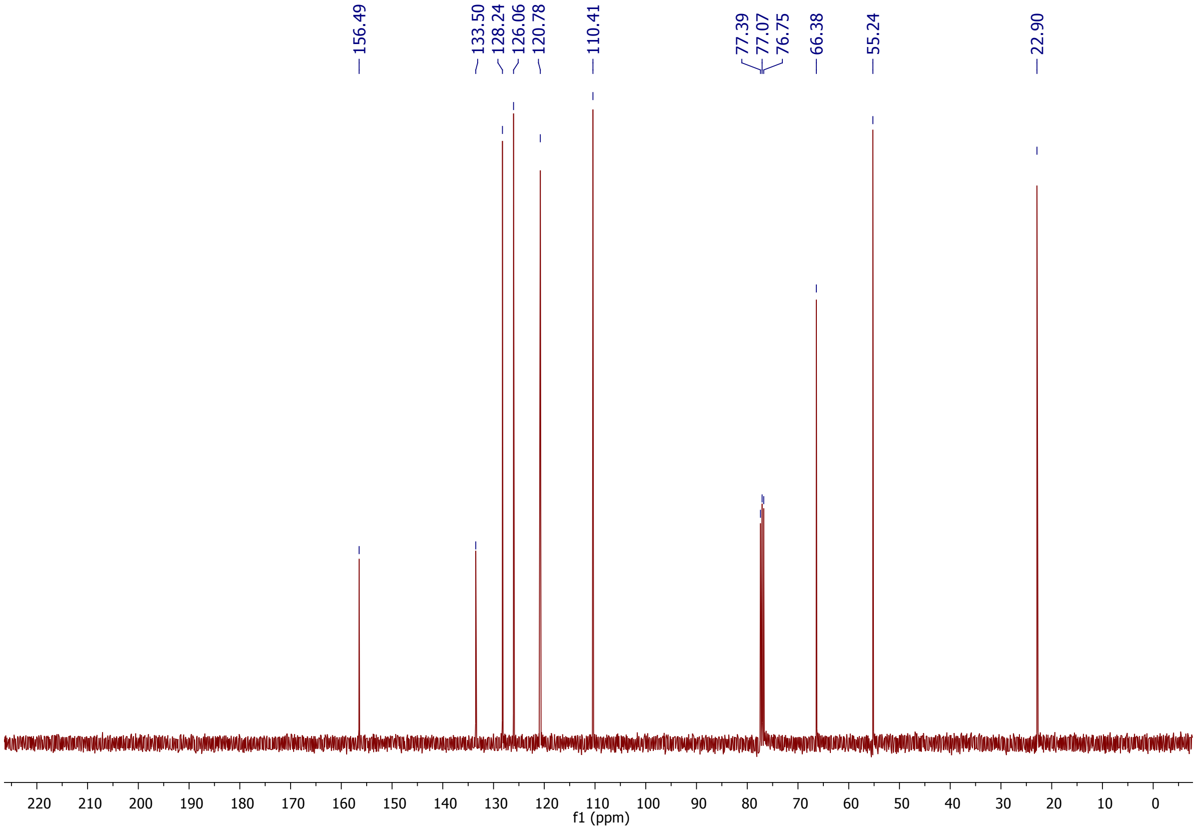

Figure S39. ^{1}H (400 MHz, $CDCl_3$) and $^{13}C\{^{1}H\}$ (100.6 MHz, $CDCl_3$) NMR spectra of **4k**

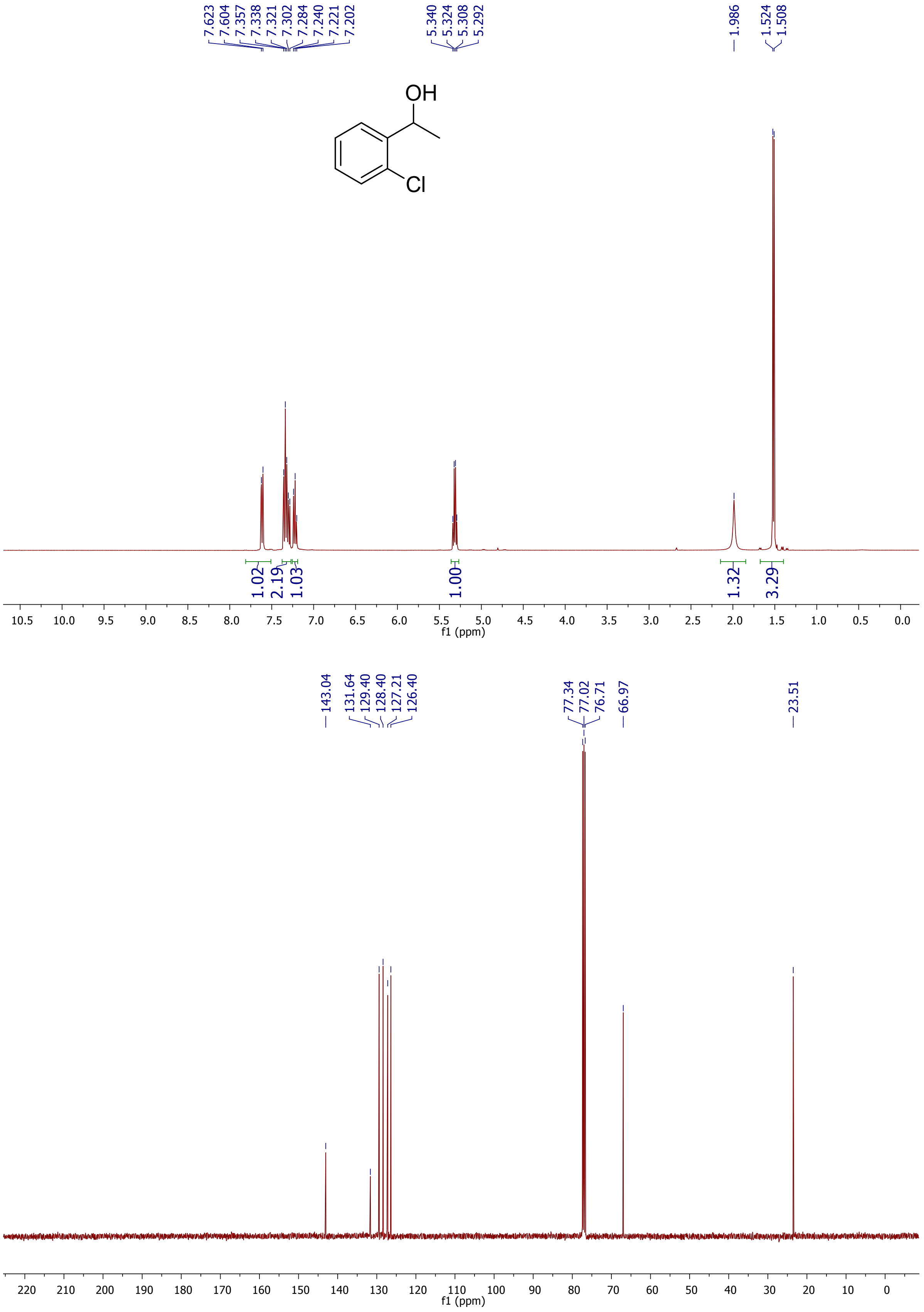

Figure S40. ^{1}H (400 MHz, $CDCl_3$) and $^{13}C\{^{1}H\}$ (100.6 MHz, $CDCl_3$) NMR spectra of **4l**

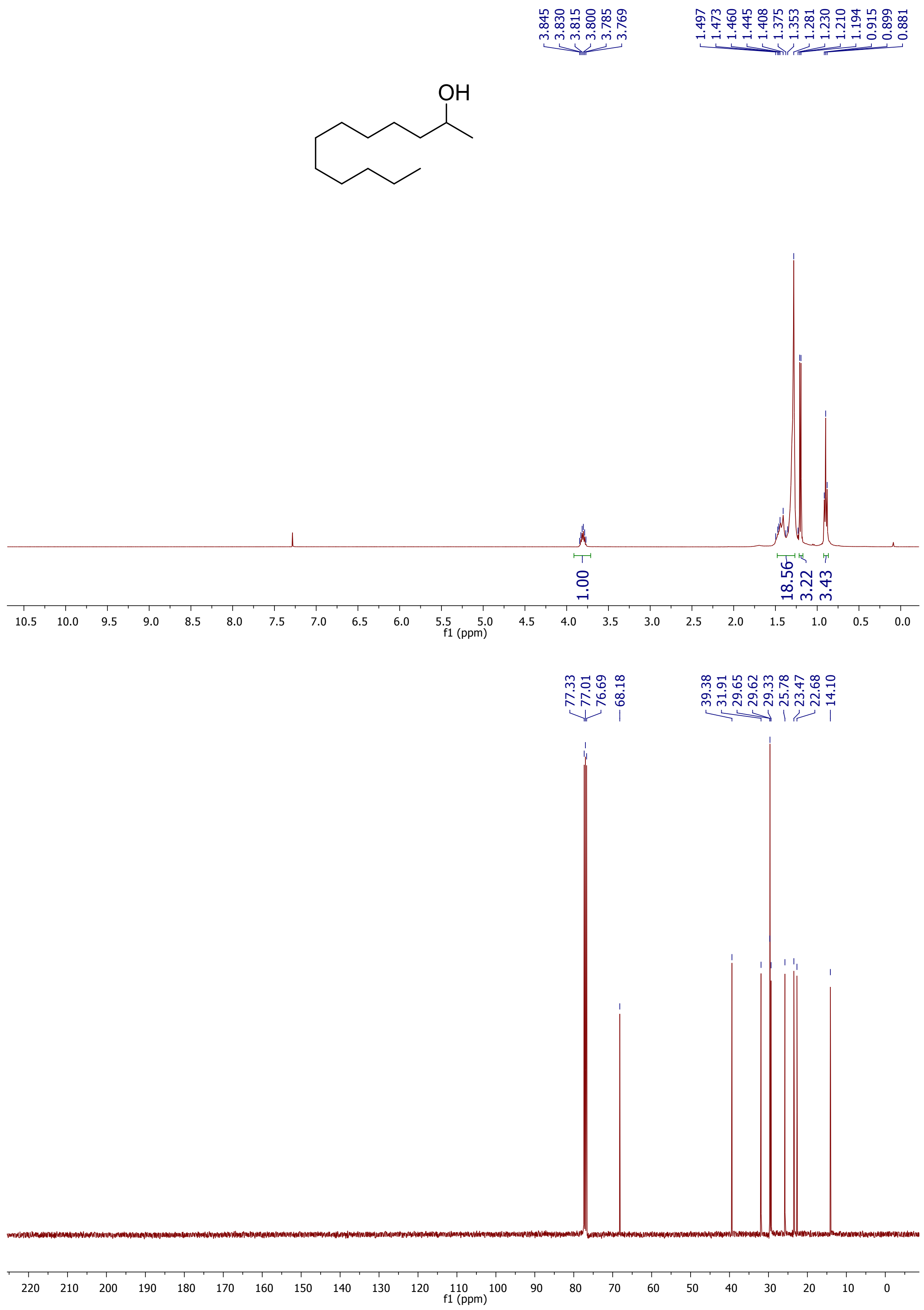

Figure S41. ^{1}H (400 MHz, $CDCl_3$) and $^{13}C\{^{1}H\}$ (100.6 MHz, $CDCl_3$) NMR spectra of **4n**

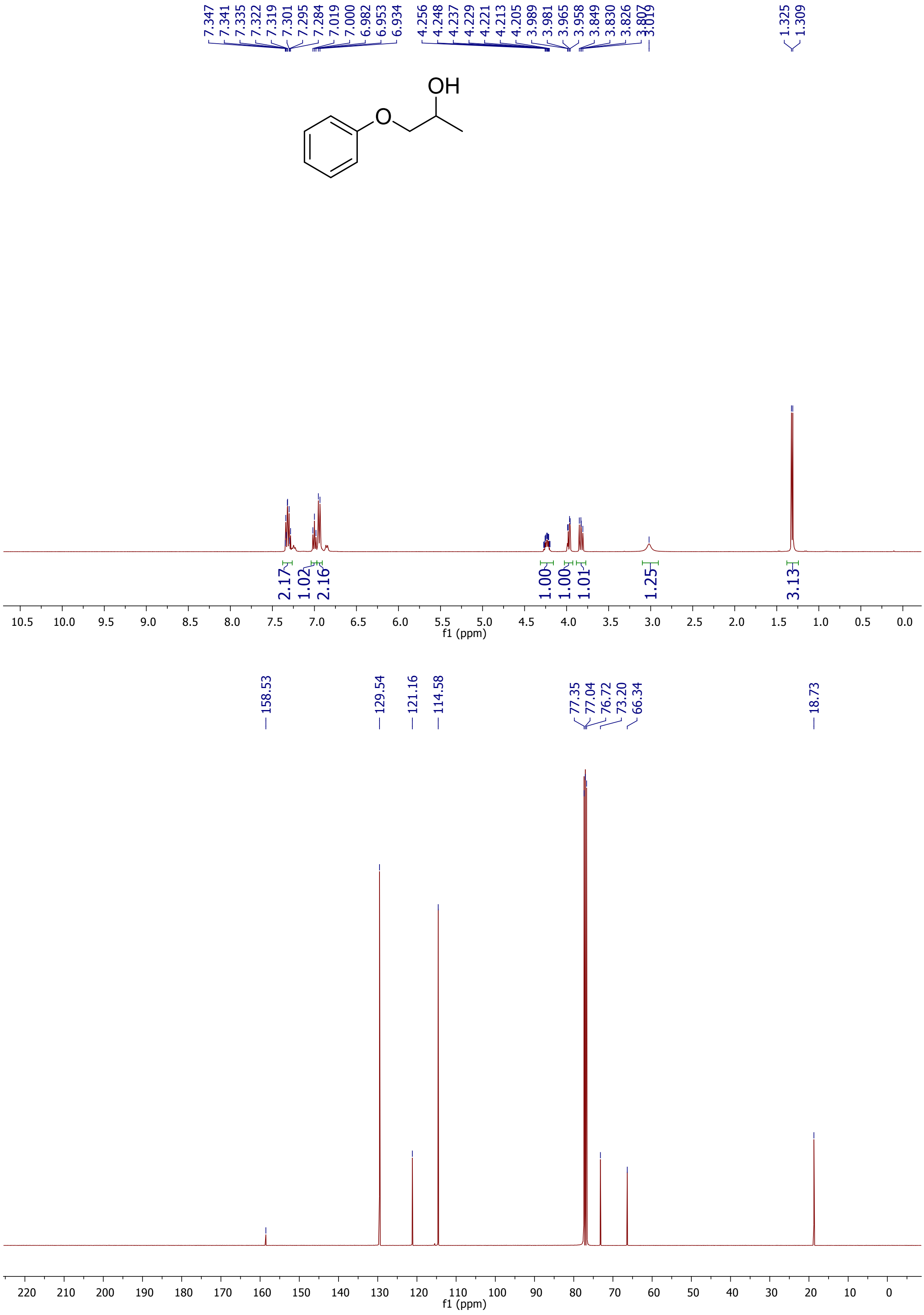

Figure S42. ^{1}H (400 MHz, $CDCl_3$) and $^{13}C\{^{1}H\}$ (100.6 MHz, $CDCl_3$) NMR spectra of **4o**

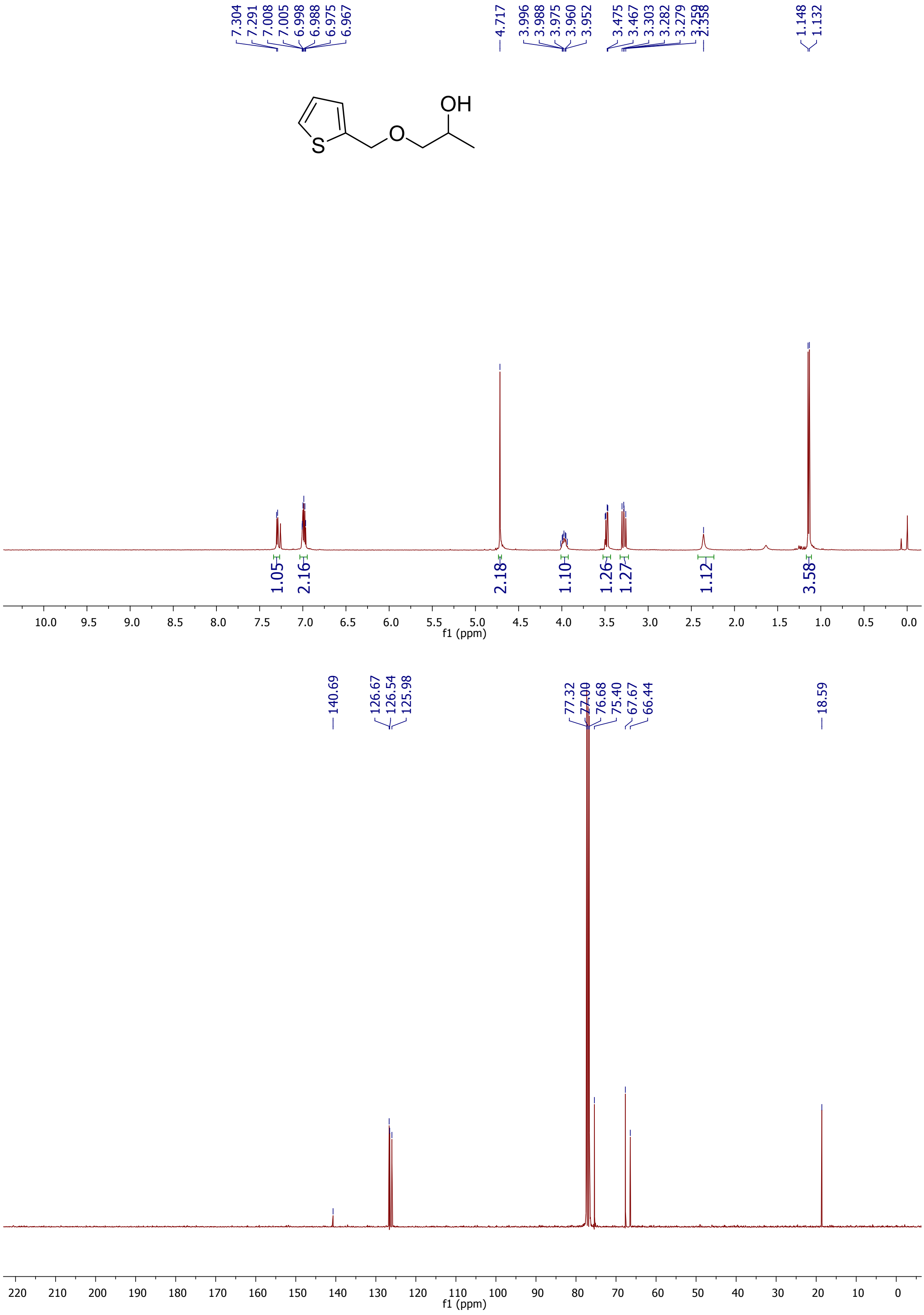

Figure S43. ^{1}H (400 MHz, $CDCl_3$) and $^{13}C\{^{1}H\}$ (100.6 MHz, $CDCl_3$) NMR spectra of **4p**

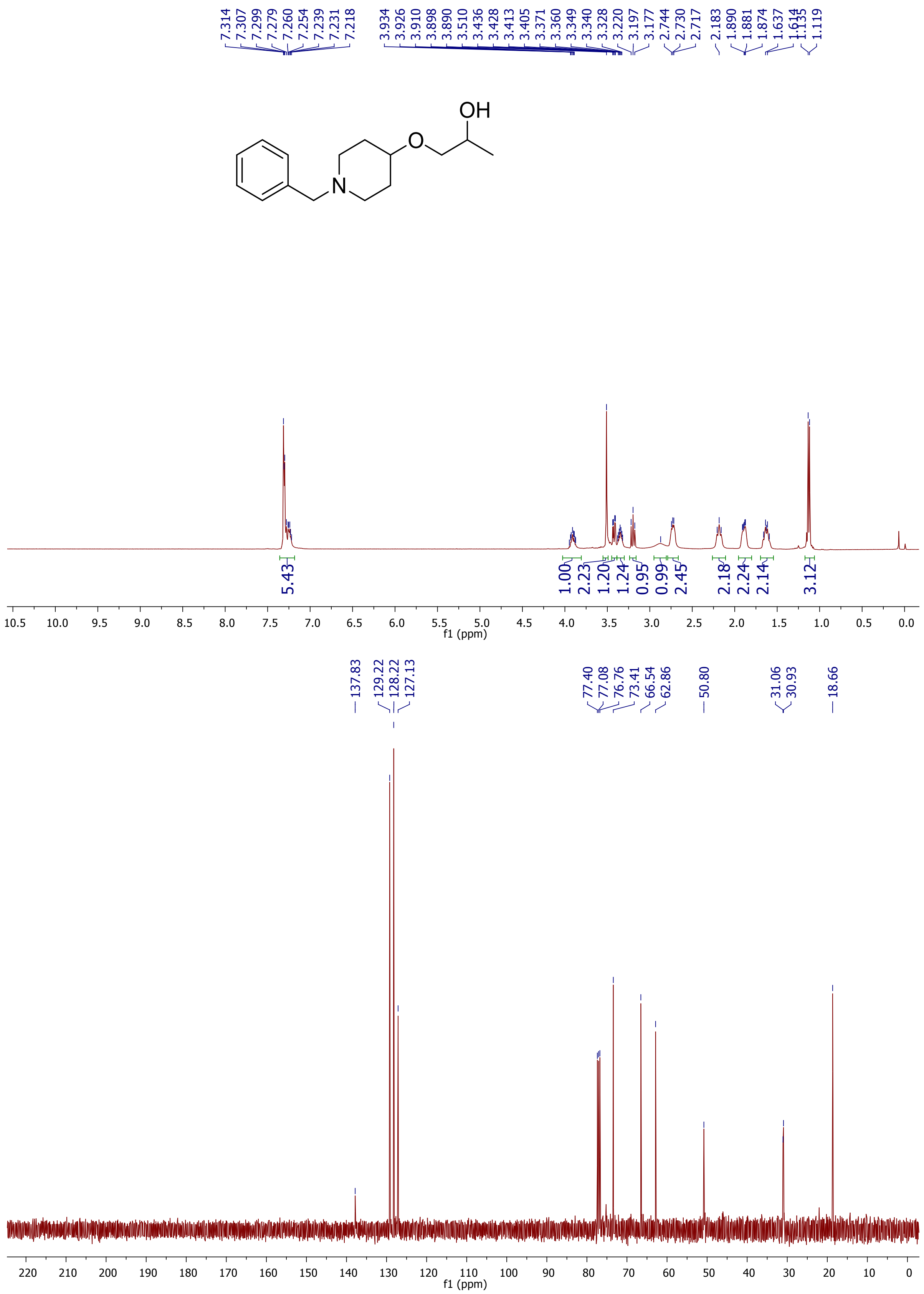

Figure S44. ^{1}H (400 MHz, $CDCl_3$) and $^{13}C\{^{1}H\}$ (100.6 MHz, $CDCl_3$) NMR spectra of **4q**

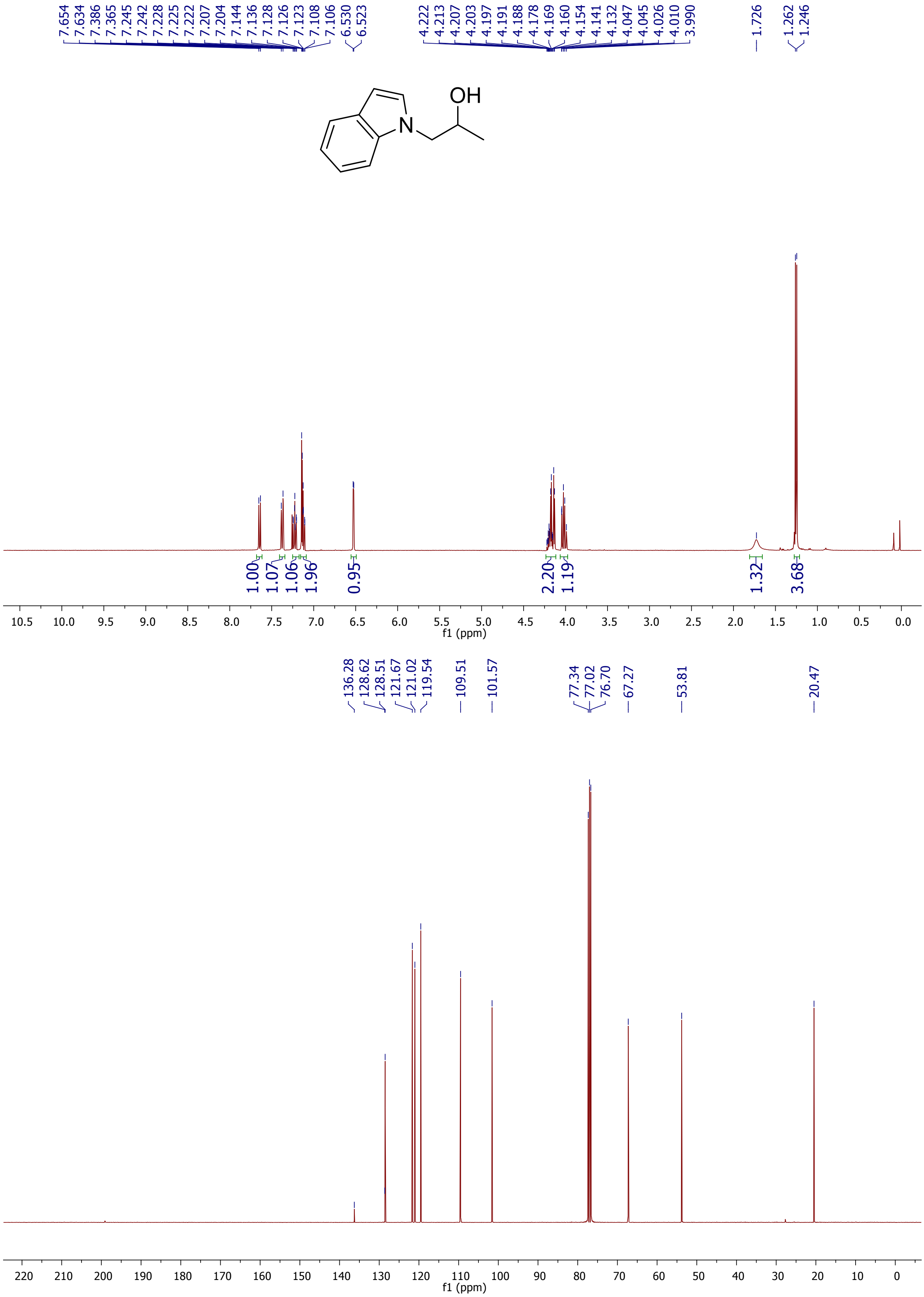

Figure S45. ^{1}H (400 MHz, $CDCl_3$) and $^{13}C\{^1H\}$ (100.6 MHz, $CDCl_3$) NMR spectra of **4r**

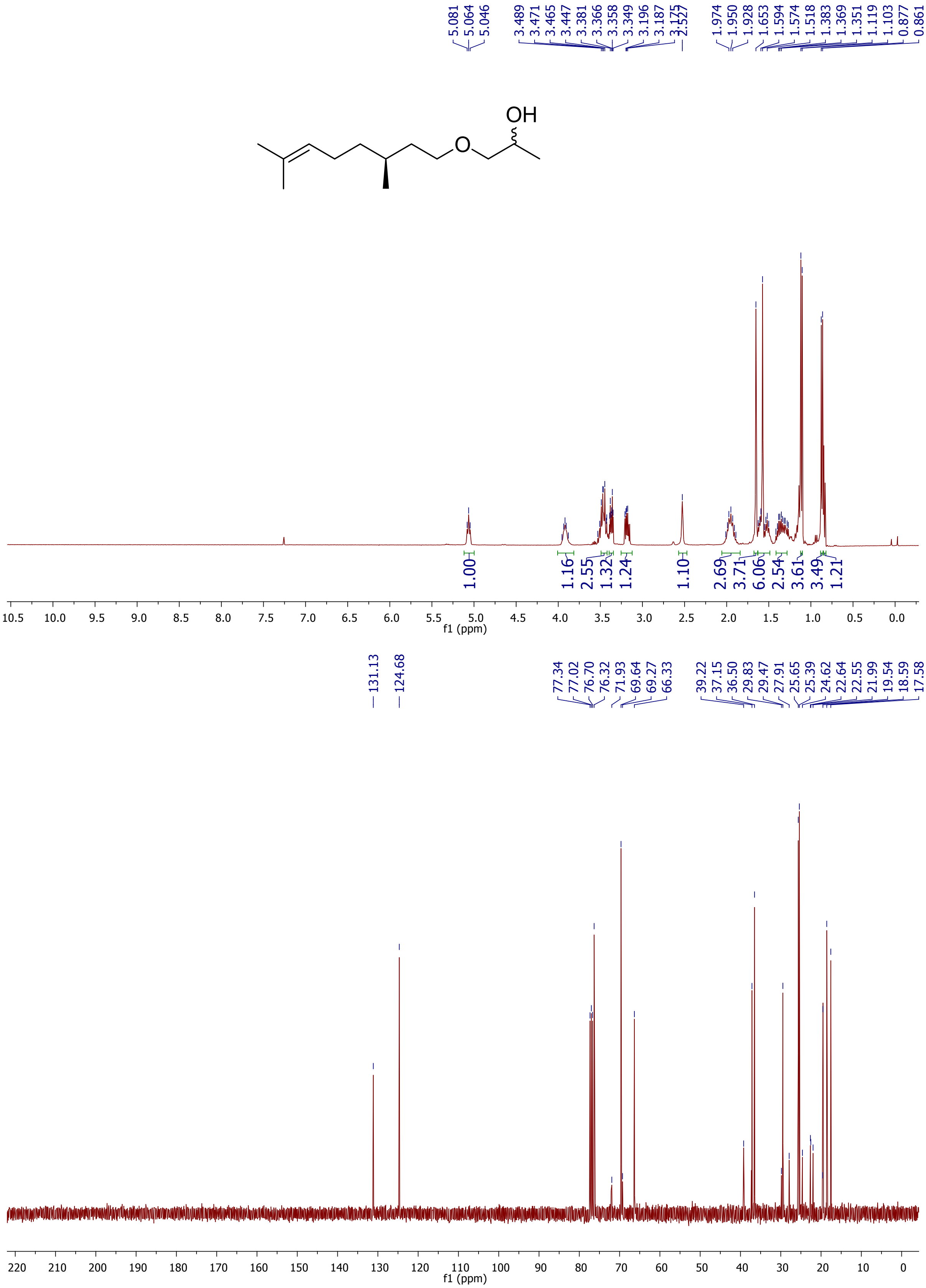

Figure S46. ^{1}H (400 MHz, $CDCl_3$) and $^{13}C\{^{1}H\}$ (100.6 MHz, $CDCl_3$) NMR spectra of **4s**

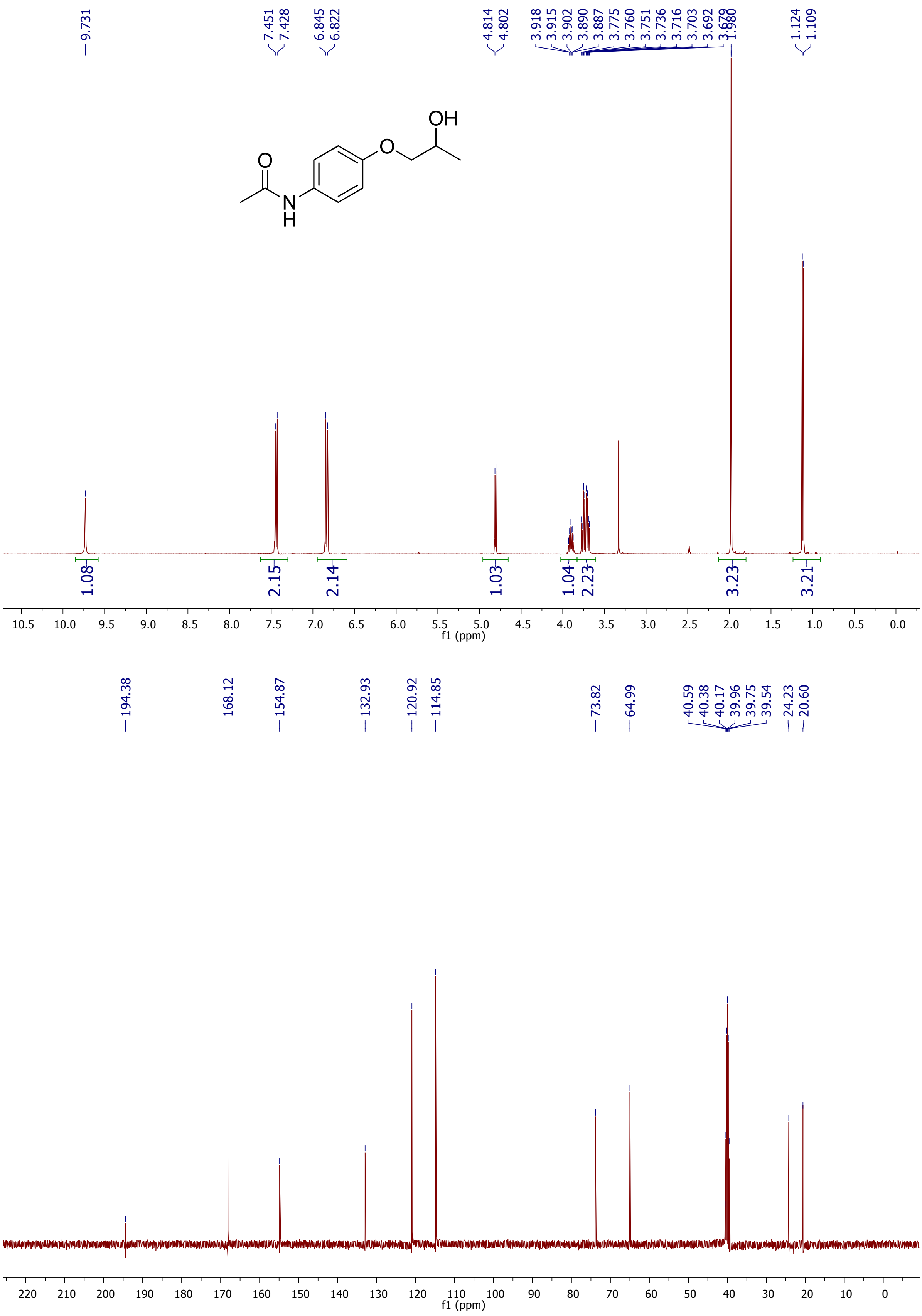

Figure S47. ^{1}H (400 MHz, DMSO-d_6) and $^{13}C\{^{1}H\}$ (100.6 MHz, DMSO-d_6) NMR spectra of **4u**

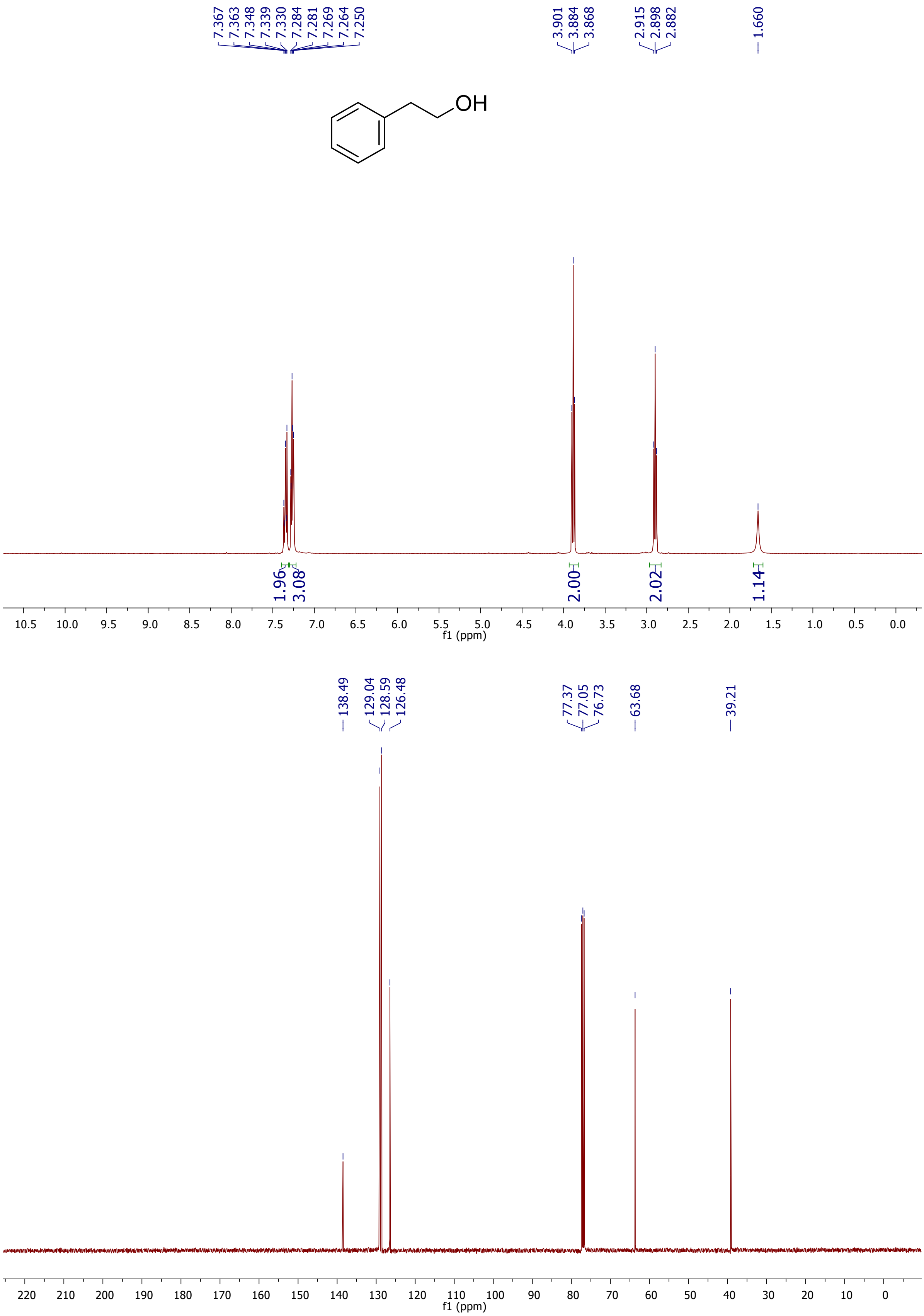

Figure S48. ^{1}H (400 MHz, $CDCl_3$) and $^{13}C\{^{1}H\}$ (100.6 MHz, $CDCl_3$) NMR spectra of **5a**

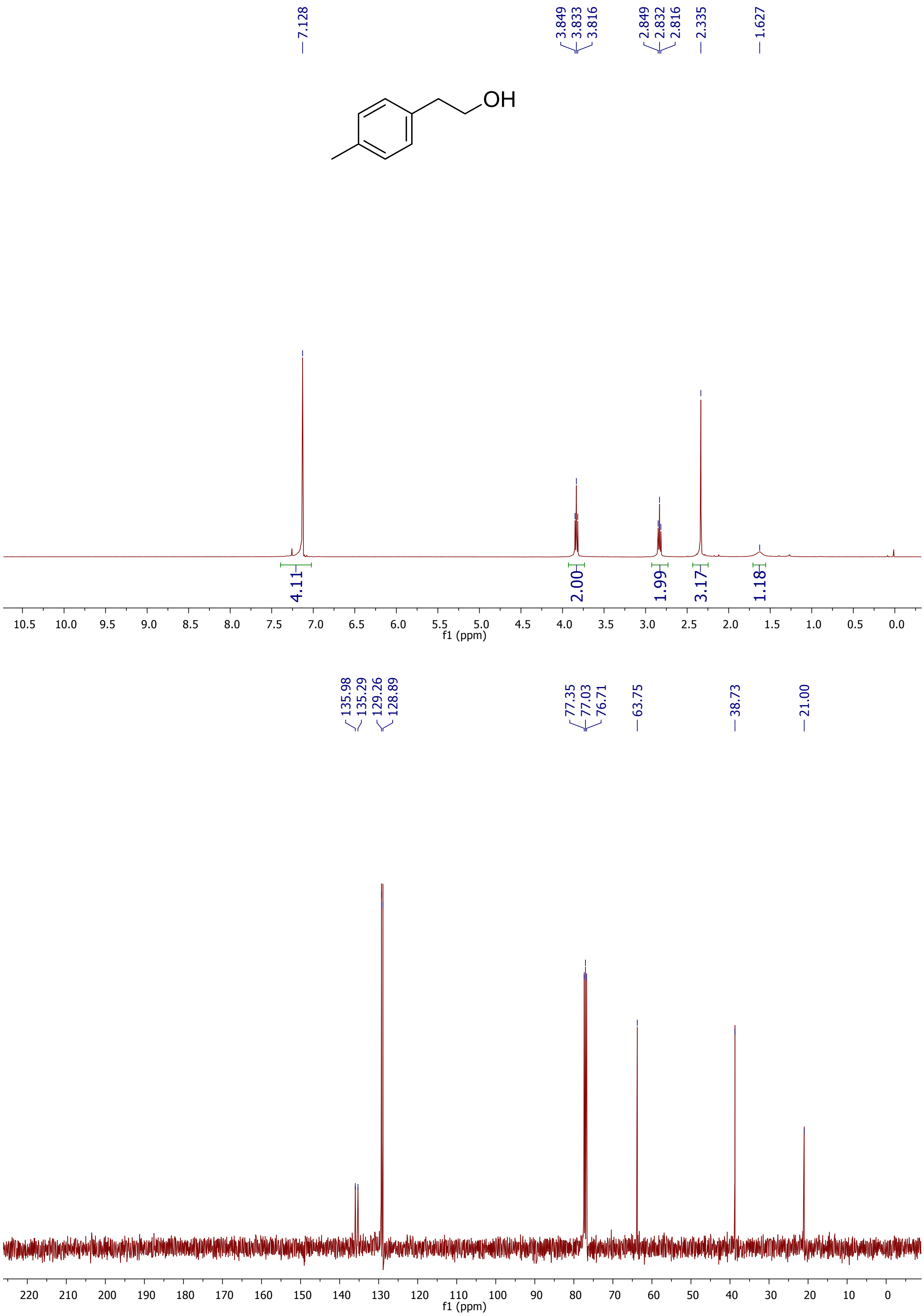

Figure S49. ^{1}H (400 MHz, $CDCl_3$) and $^{13}C\{^{1}H\}$ (100.6 MHz, $CDCl_3$) NMR spectra of **5b**

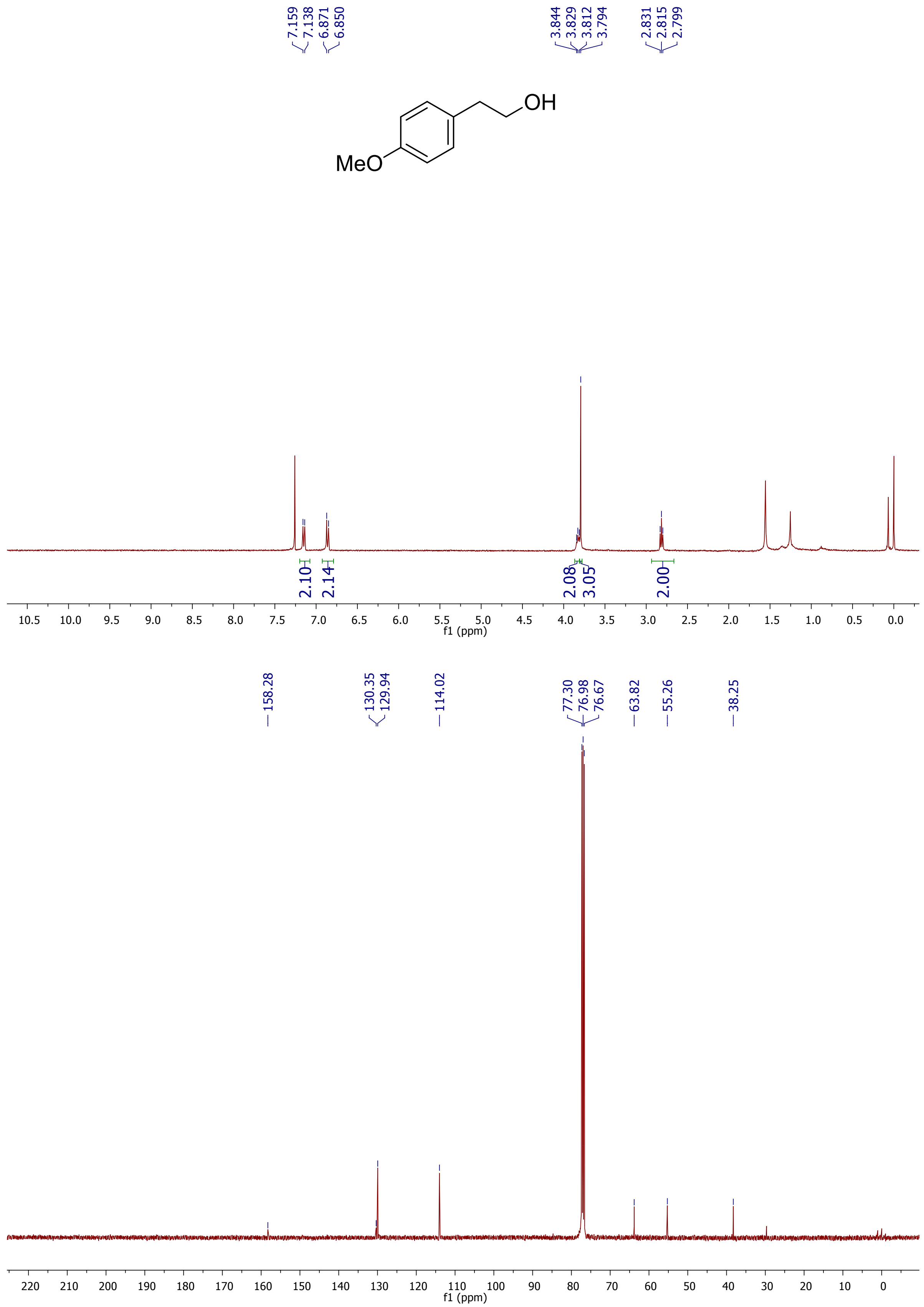

Figure S50. ^{1}H (400 MHz, $CDCl_3$) and ^{13}C{^{1}H} (100.6 MHz, $CDCl_3$) NMR spectra of **5c**

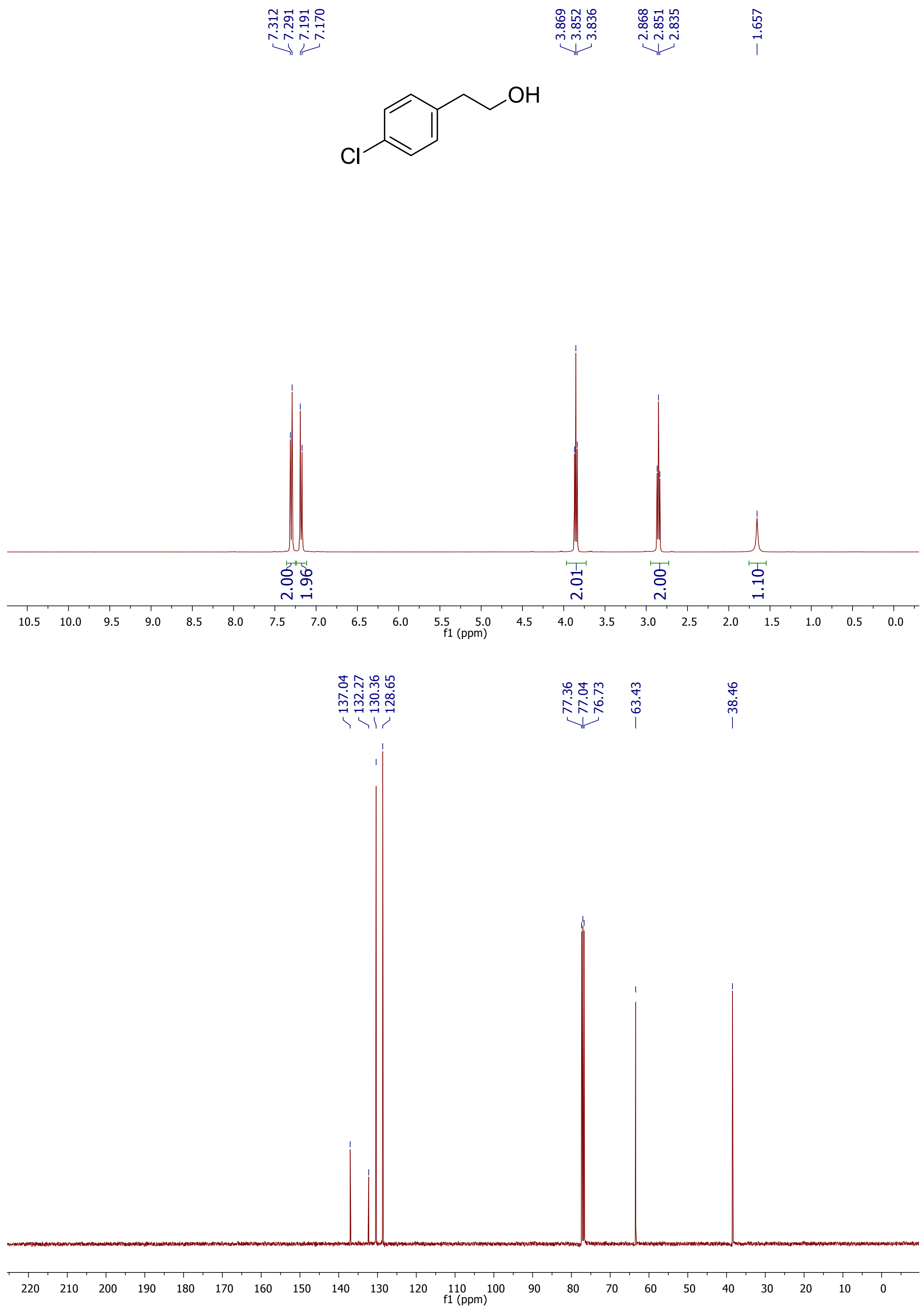

Figure S51. ^{1}H (400 MHz, $CDCl_3$) and $^{13}C\{^{1}H\}$ (100.6 MHz, $CDCl_3$) NMR spectra of **5d**

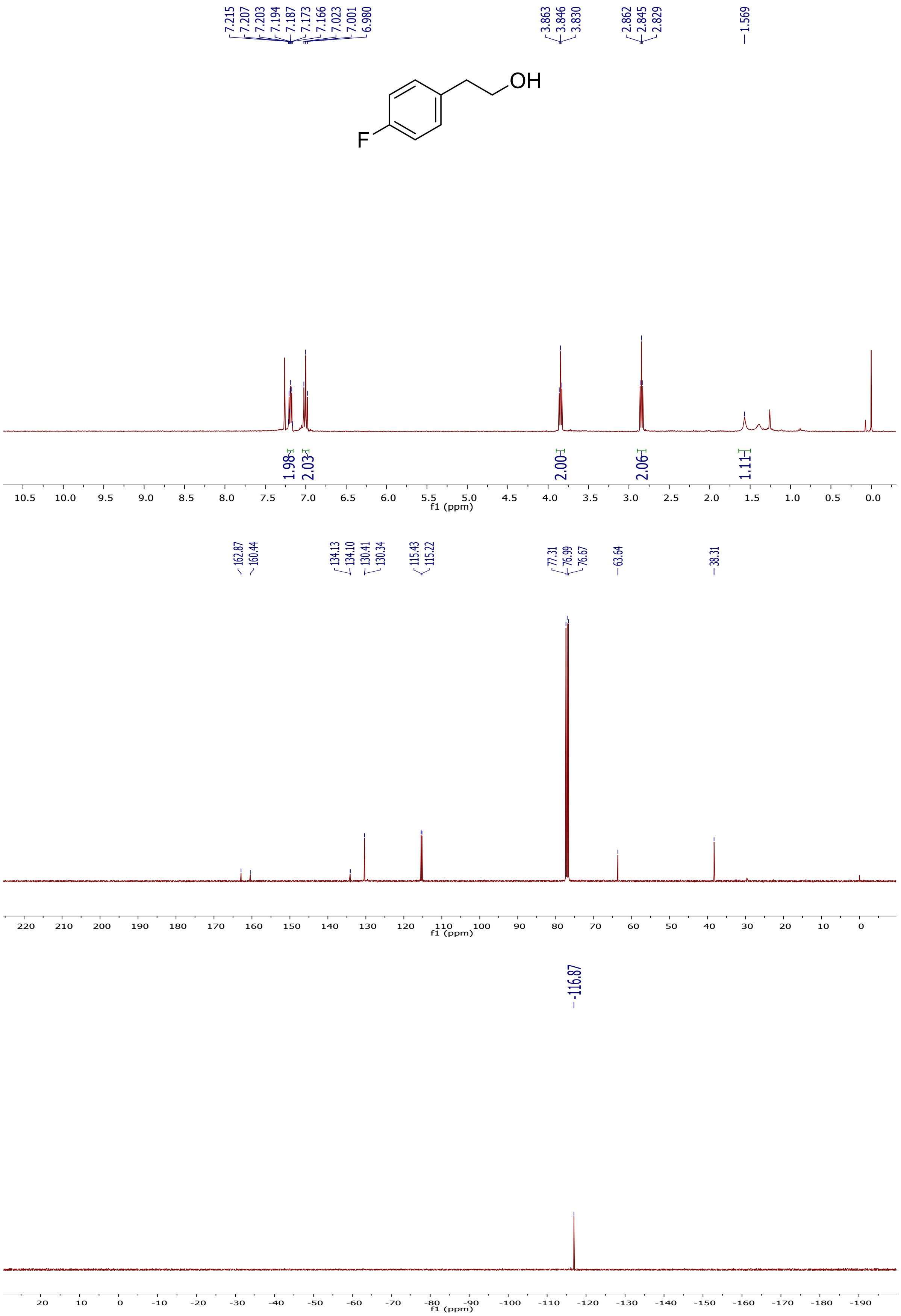

Figure S52. ^{1}H (400 MHz, $CDCl_3$), $^{13}C\{^{1}H\}$ (100.6 MHz, $CDCl_3$) and ^{19}F (376 MHz, $CDCl_3$) NMR spectra of **5e**

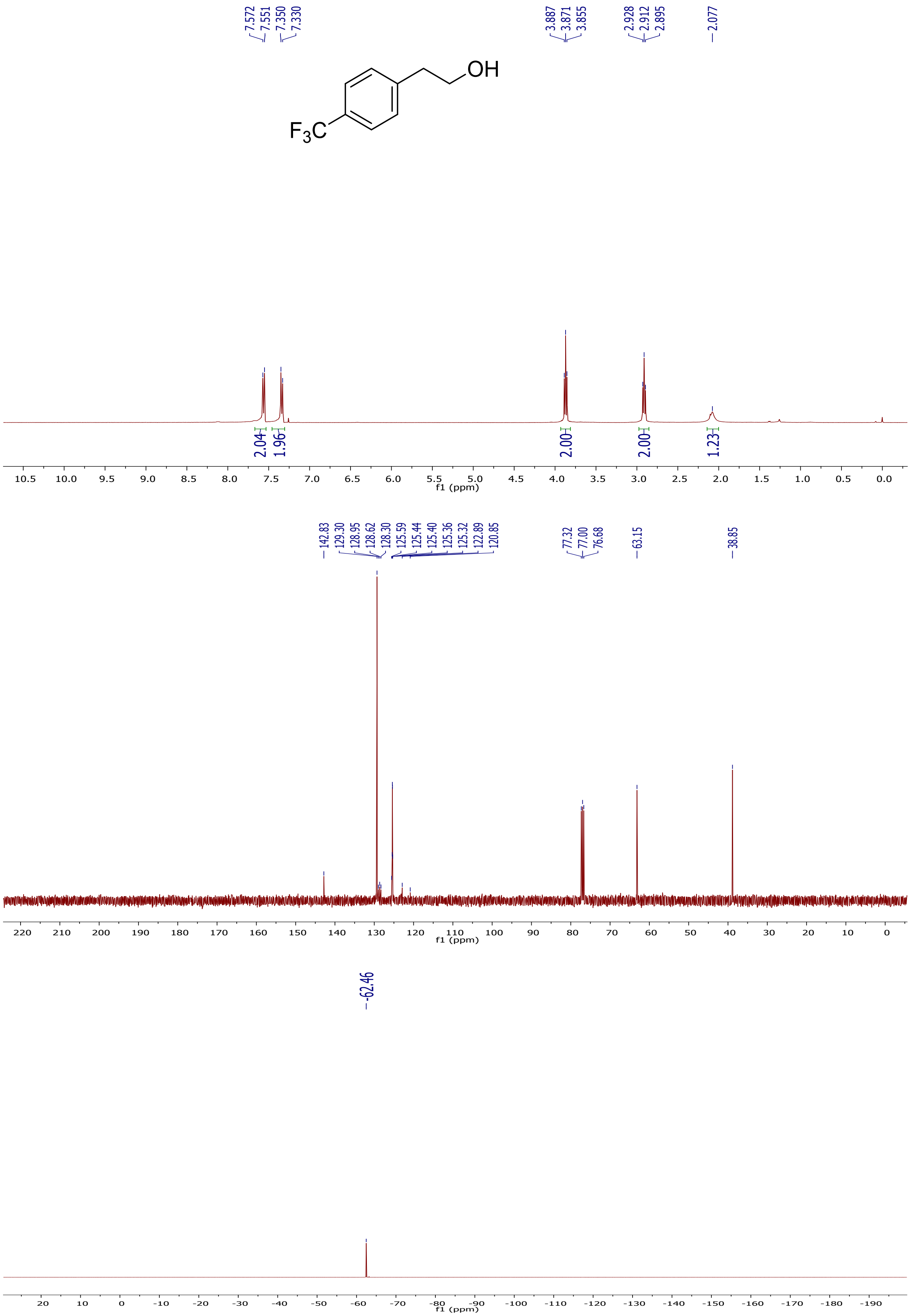

Figure S53. ^{1}H (400 MHz, $CDCl_3$), $^{13}C\{^{1}H\}$ (100.6 MHz, $CDCl_3$) and ^{19}F (376 MHz, $CDCl_3$) NMR spectra of **5f**

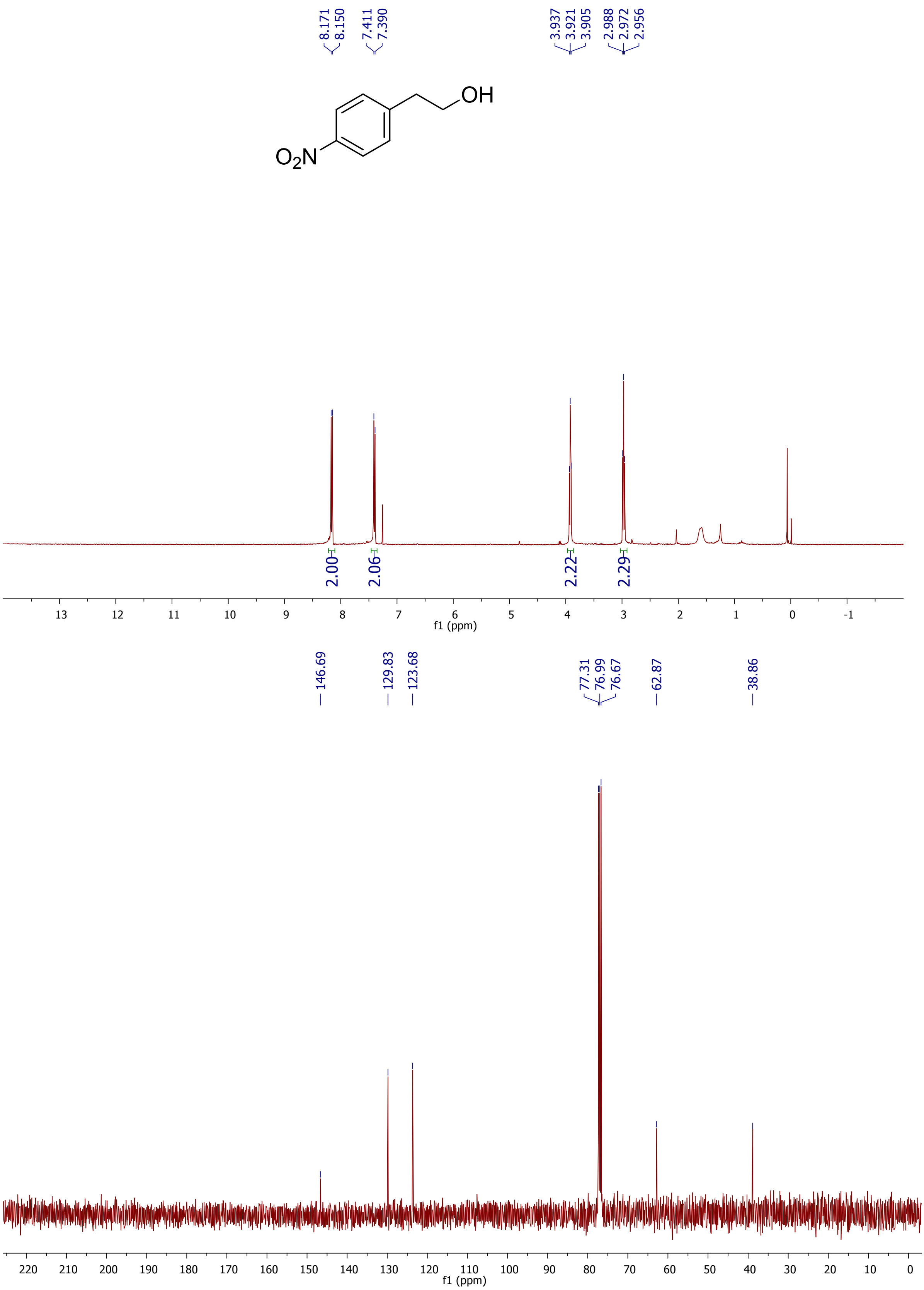

Figure S54. ^{1}H (400 MHz, $CDCl_3$) and $^{13}C\{^{1}H\}$ (100.6 MHz, $CDCl_3$) NMR spectra of **5g**

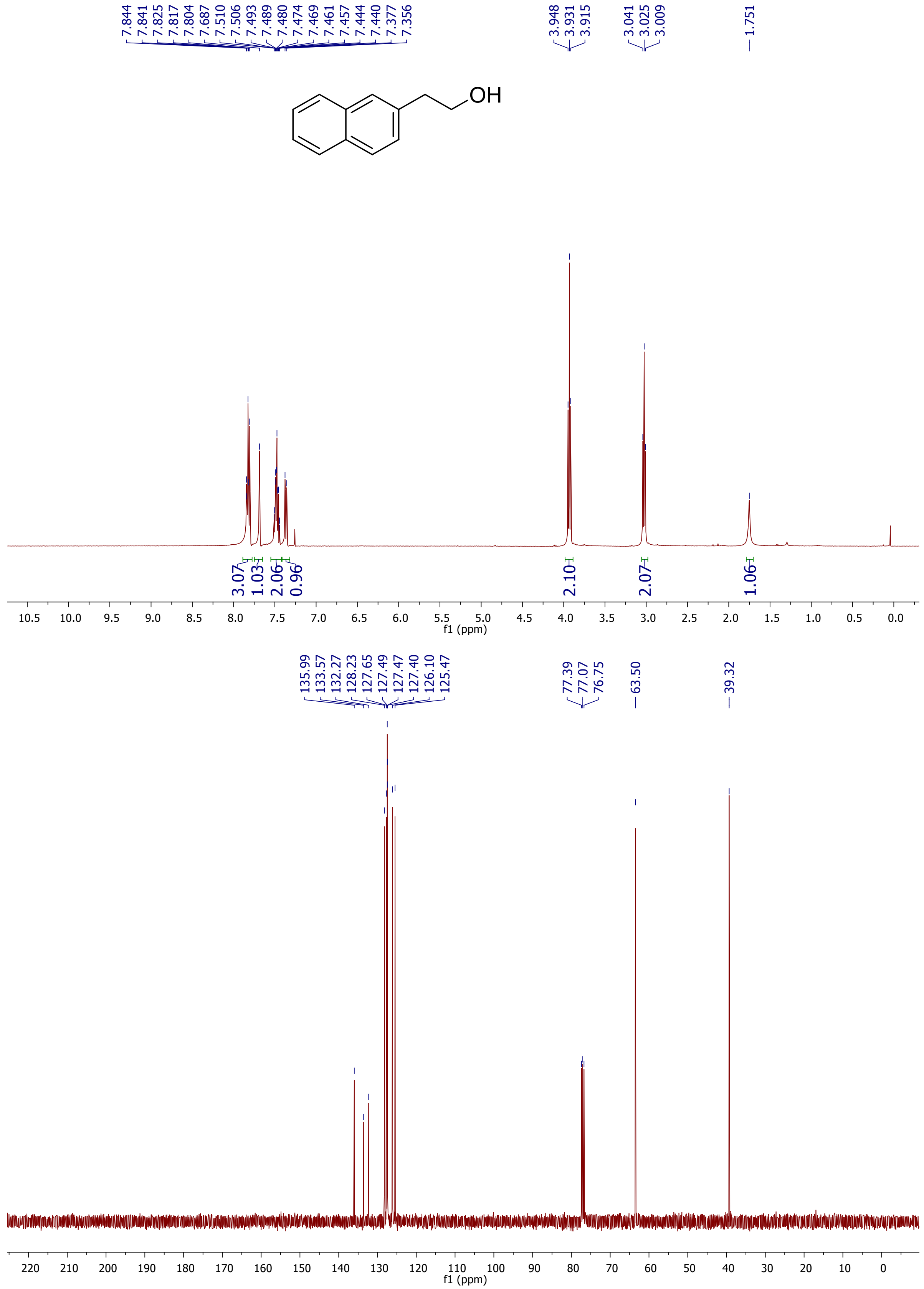

Figure S55. ^{1}H (400 MHz, $CDCl_3$) and $^{13}C\{^{1}H\}$ (100.6 MHz, $CDCl_3$) NMR spectra of **5h**

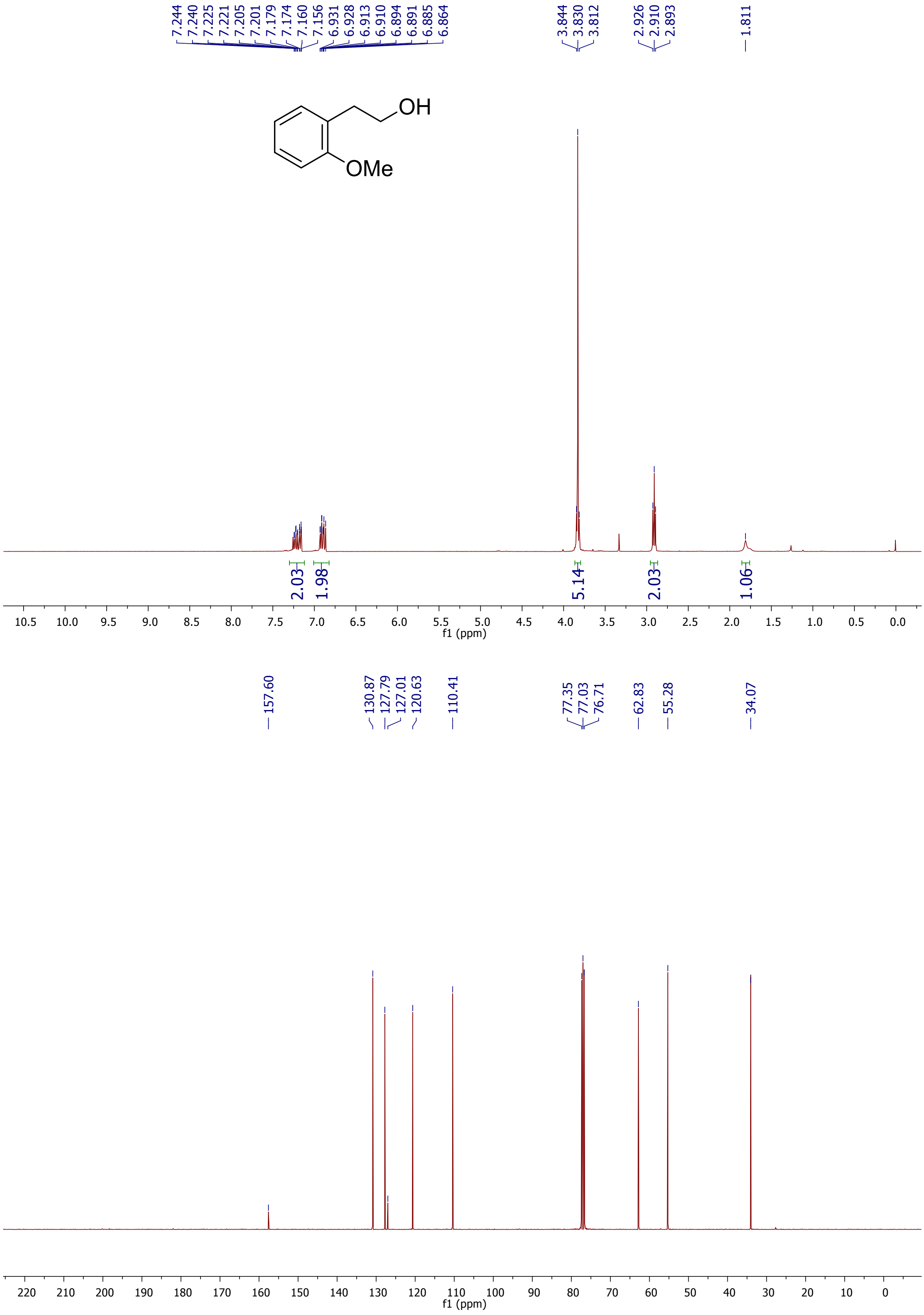

Figure S56. ^{1}H (400 MHz, $CDCl_3$) and $^{13}C\{^{1}H\}$ (100.6 MHz, $CDCl_3$) NMR spectra of **5i**

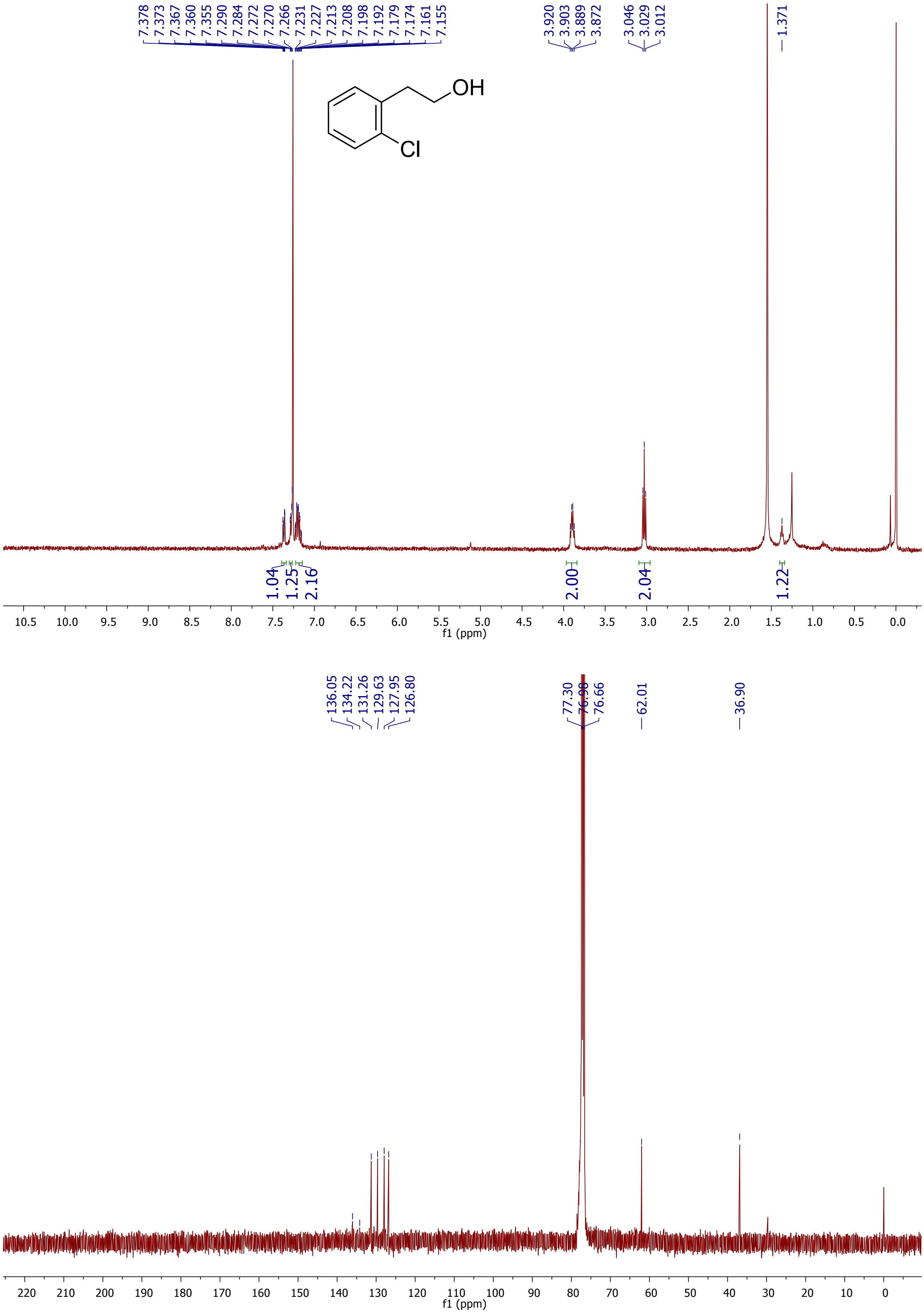

Figure S57. ^{1}H (400 MHz, $CDCl_3$) and $^{13}C\{^{1}H\}$ (100.6 MHz, $CDCl_3$) NMR spectra of **5j**

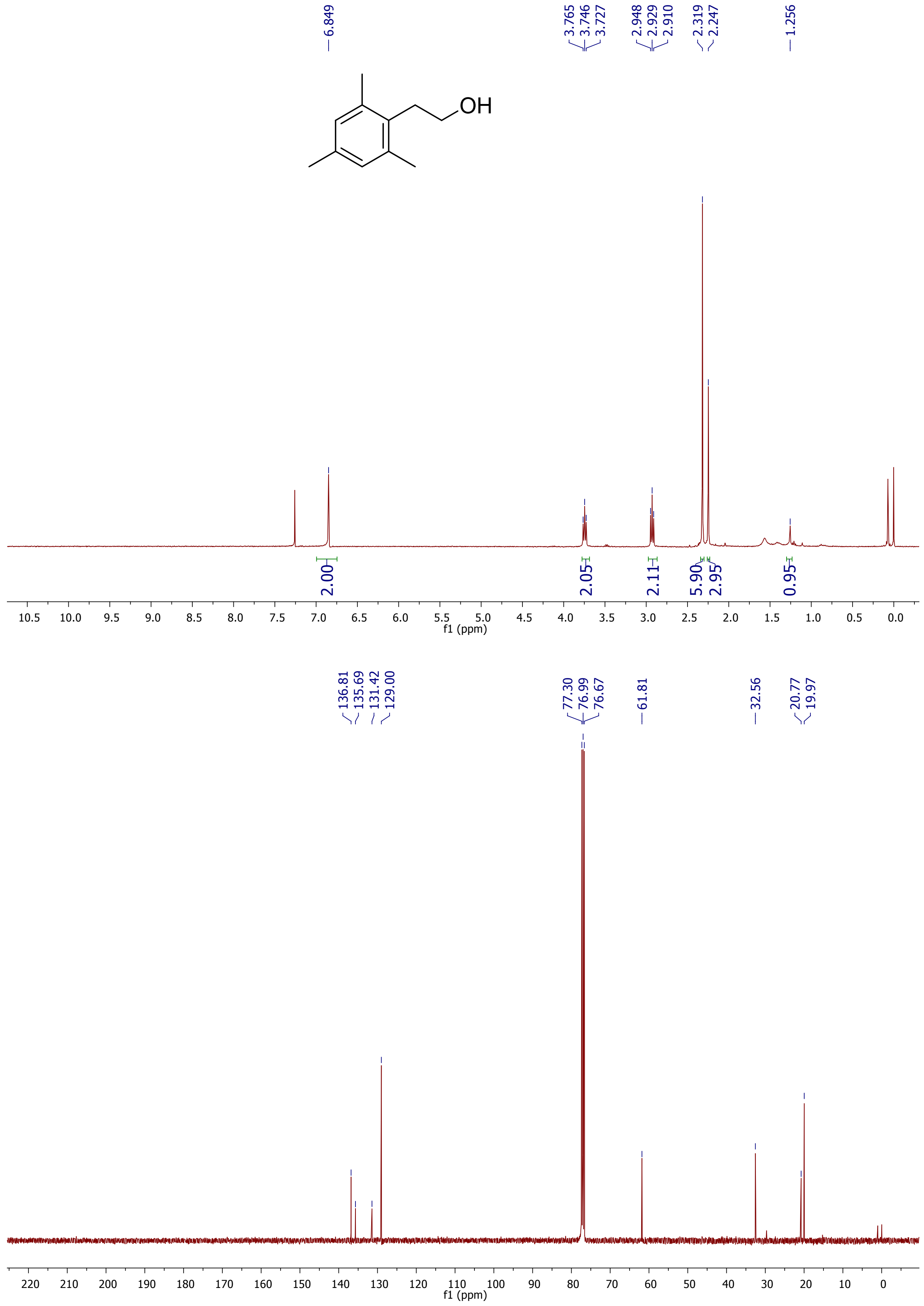

Figure S58. ^{1}H (400 MHz, $CDCl_3$) and $^{13}C\{^{1}H\}$ (100.6 MHz, $CDCl_3$) NMR spectra of **5k**

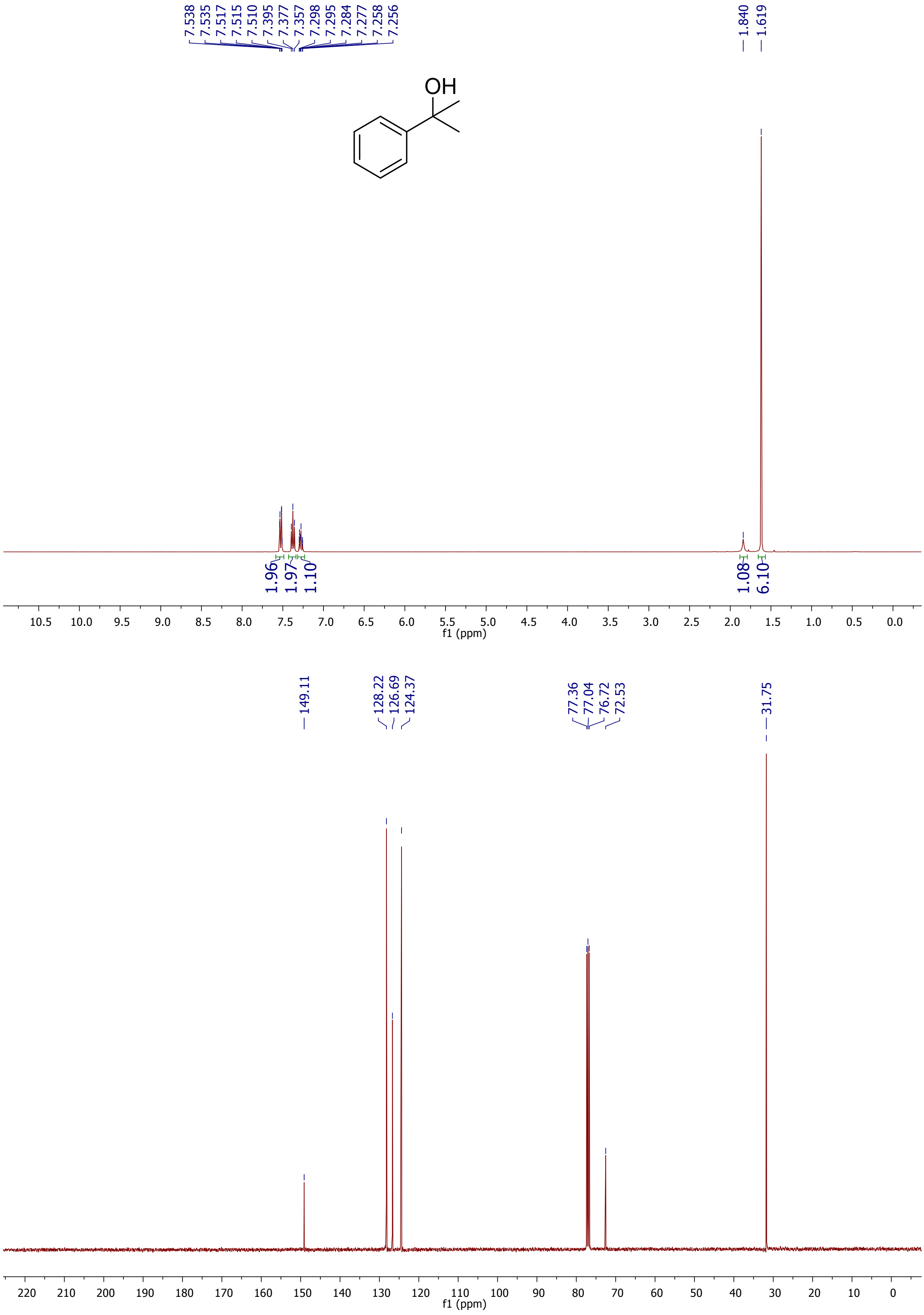

Figure S59. ^{1}H (400 MHz, $CDCl_3$) and $^{13}C\{^{1}H\}$ (100.6 MHz, $CDCl_3$) NMR spectra of **6a**

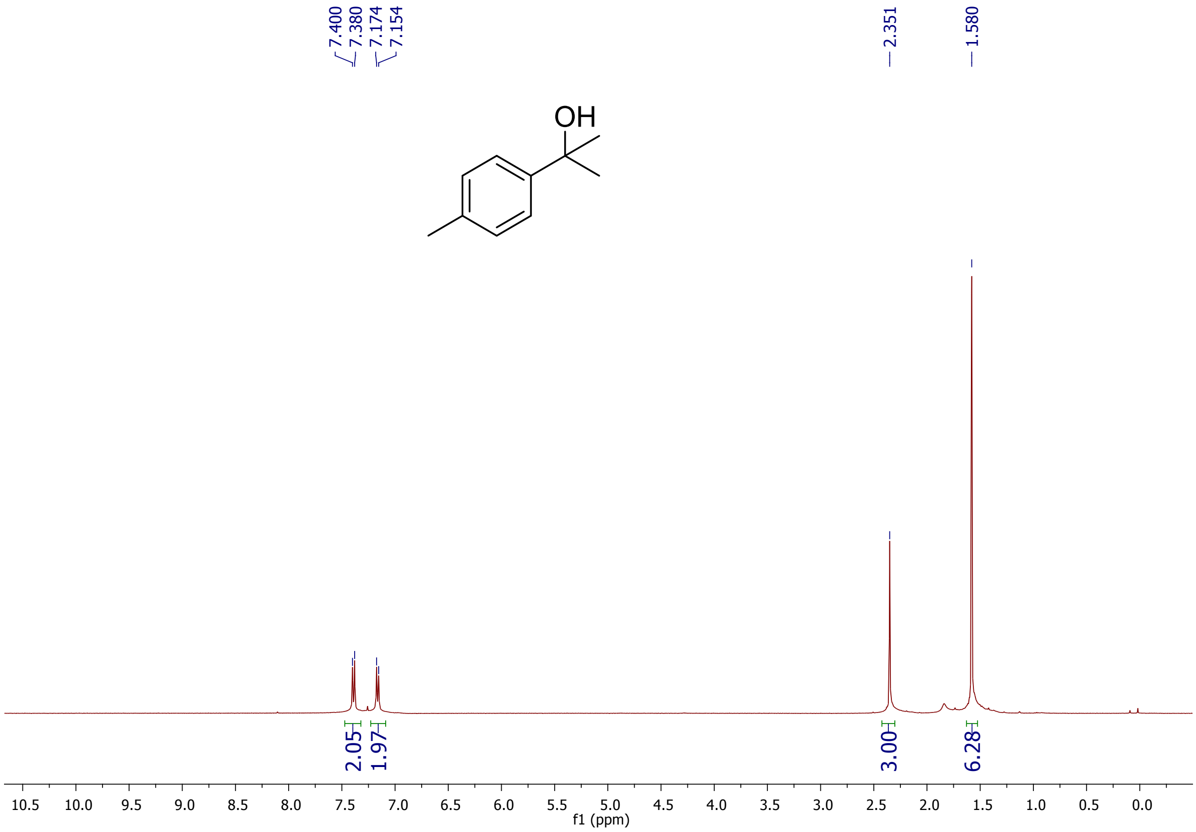

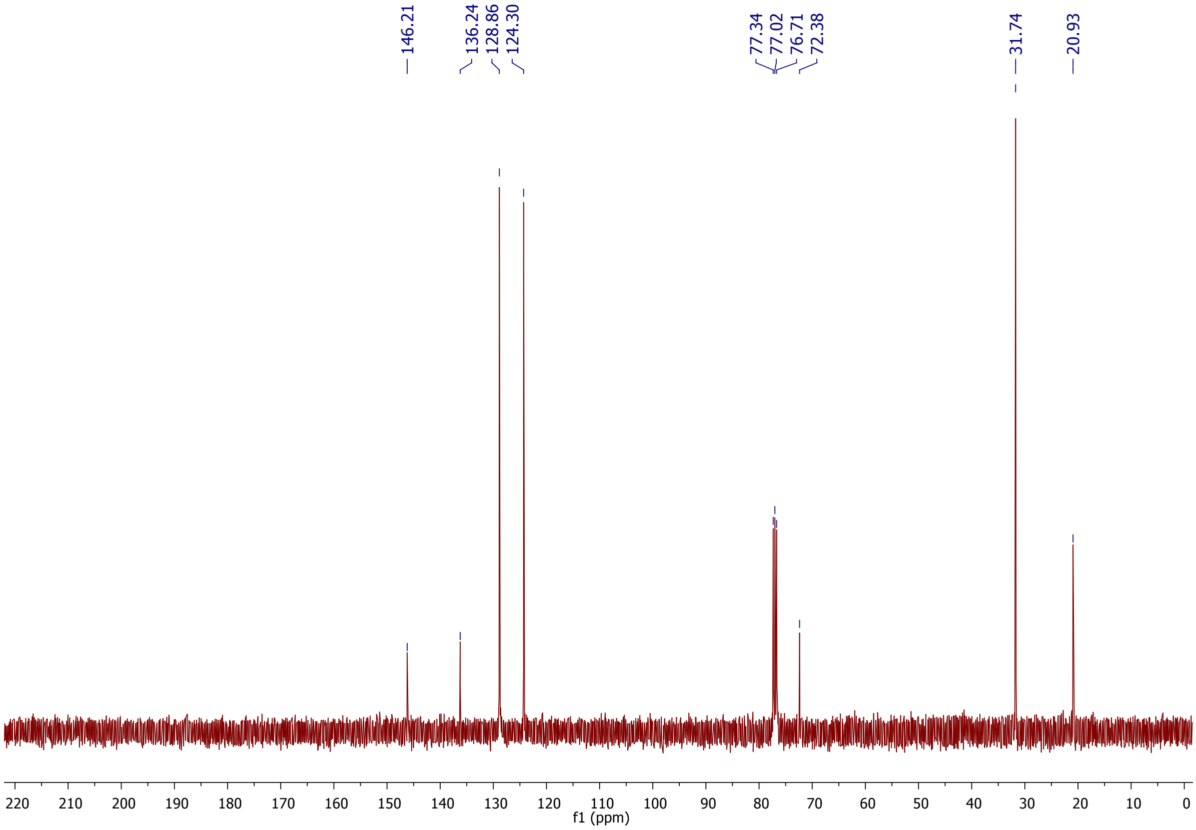

Figure S60. ^{1}H (400 MHz, $CDCl_3$) and $^{13}C\{^{1}H\}$ (100.6 MHz, $CDCl_3$) NMR spectra of **6b**

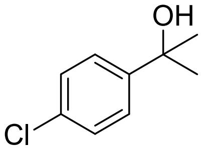

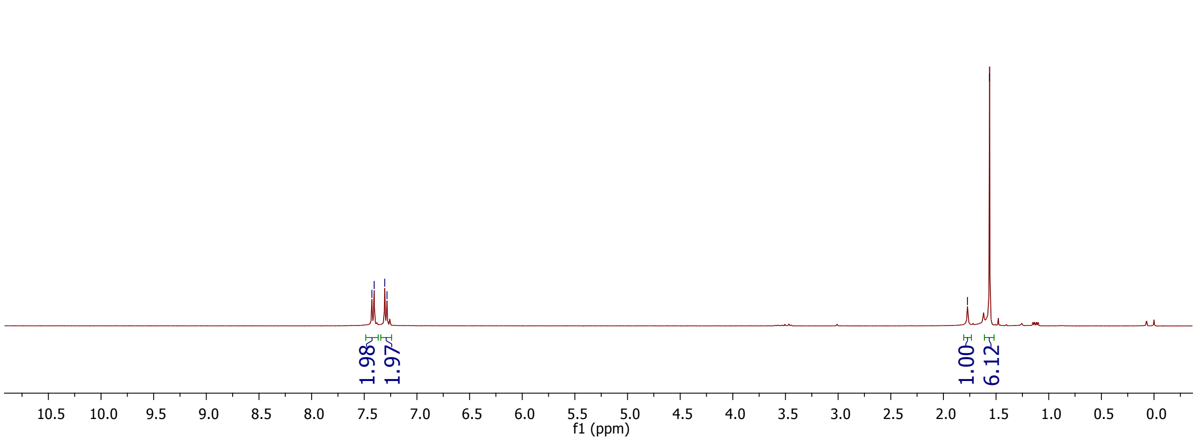

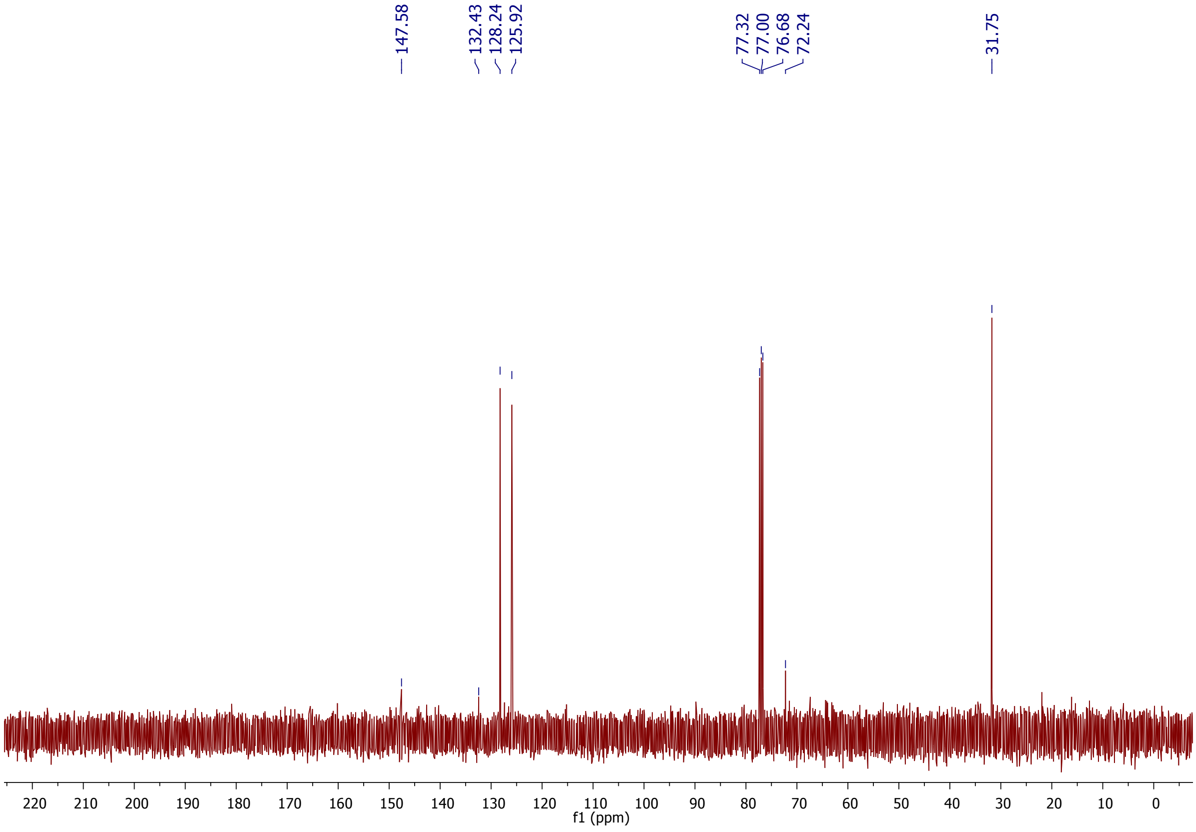

Figure S61. ^{1}H (400 MHz, $CDCl_3$) and $^{13}C\{^{1}H\}$ (100.6 MHz, $CDCl_3$) NMR spectra of **6c**

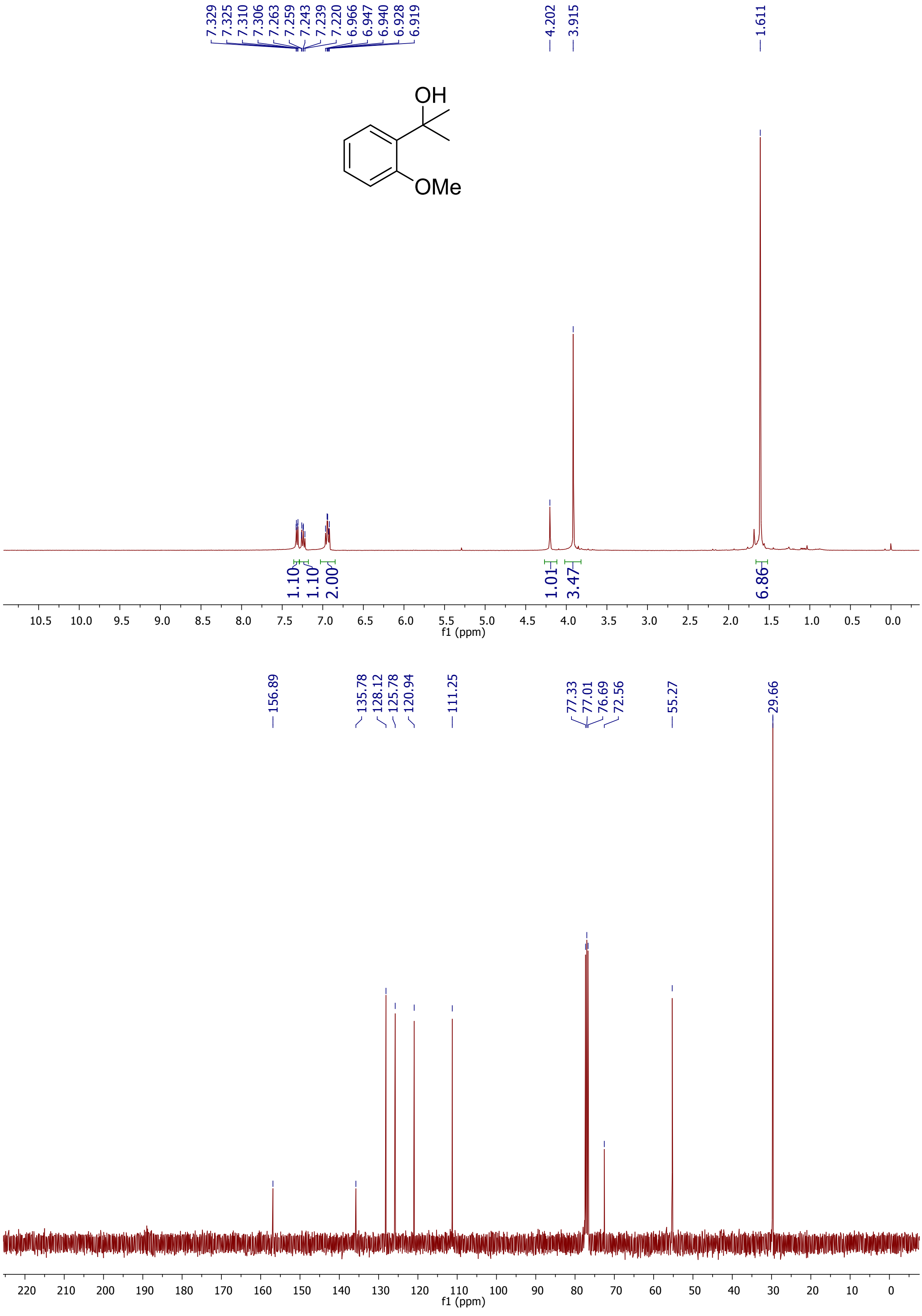

Figure S62. ^{1}H (400 MHz, $CDCl_3$) and $^{13}C\{^{1}H\}$ (100.6 MHz, $CDCl_3$) NMR spectra of **6d**

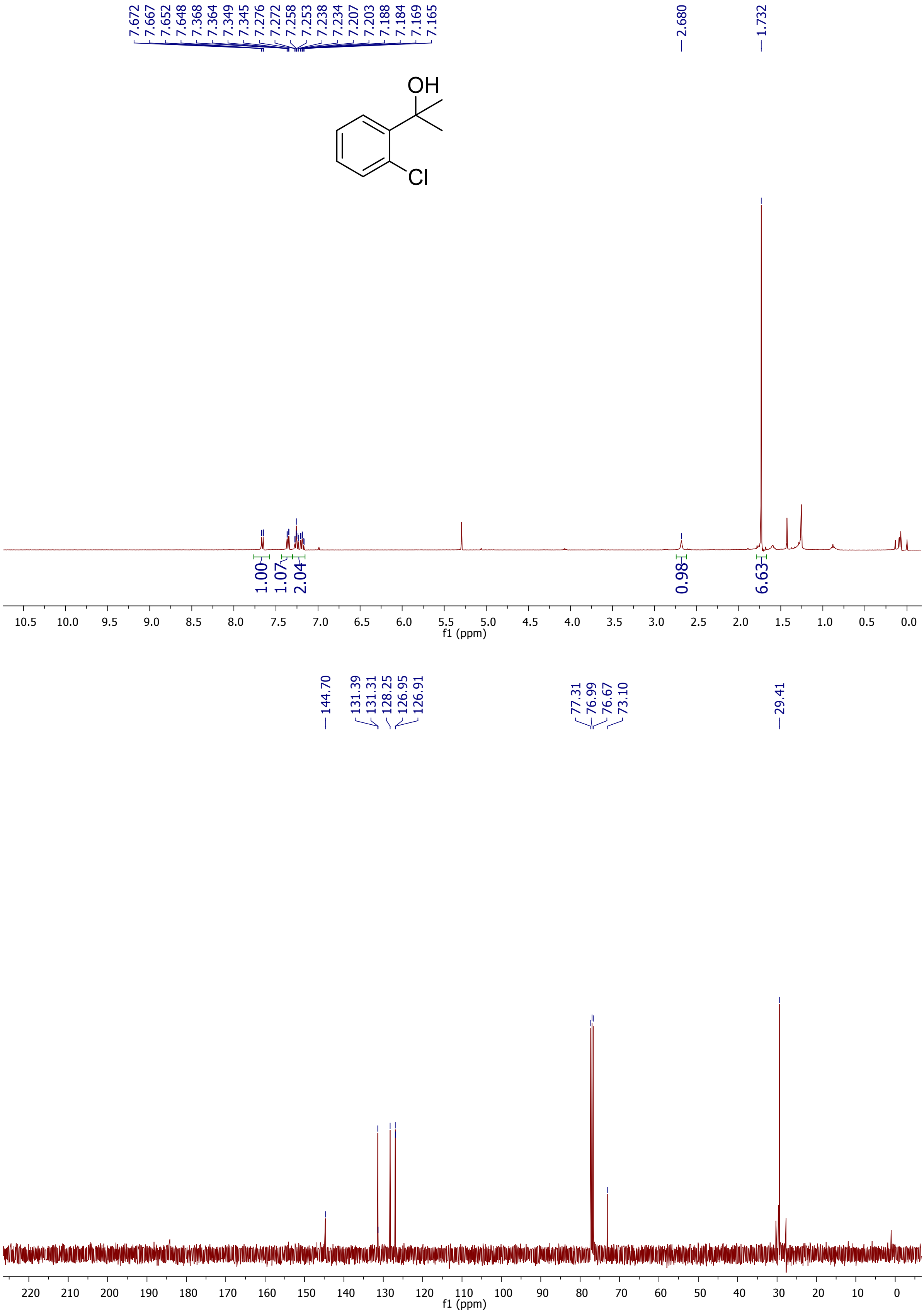

Figure S63. ^{1}H (400 MHz, $CDCl_3$) and $^{13}C\{^{1}H\}$ (100.6 MHz, $CDCl_3$) NMR spectra of **6e**

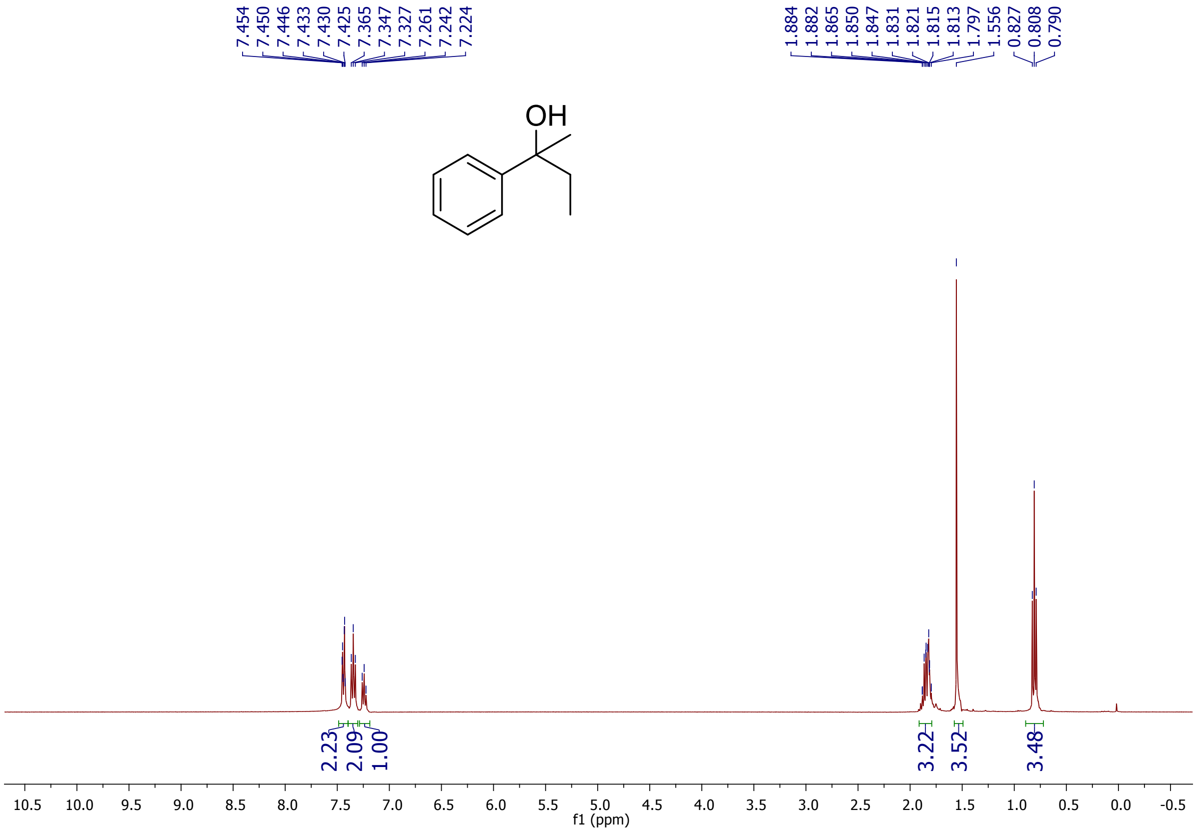

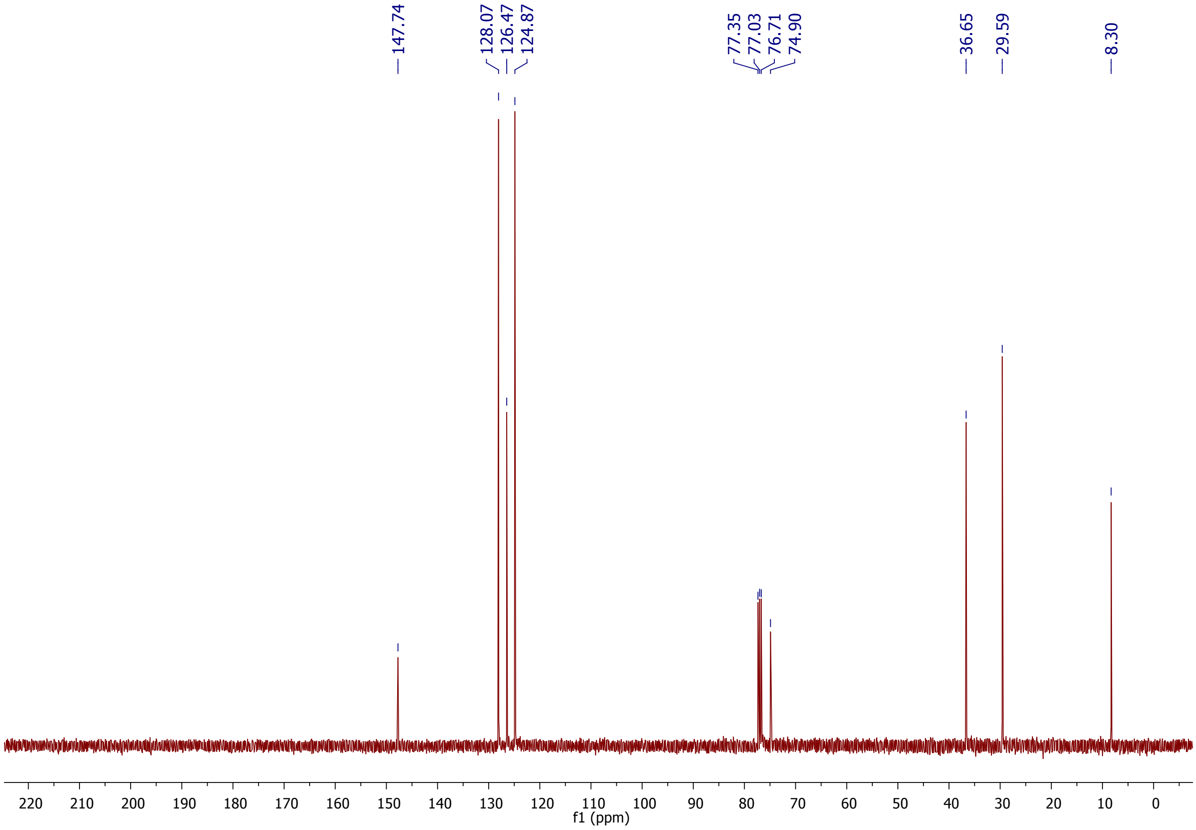

Figure S64. ^{1}H (400 MHz, $CDCl_3$) and $^{13}C\{^{1}H\}$ (100.6 MHz, $CDCl_3$) NMR spectra of **6f**

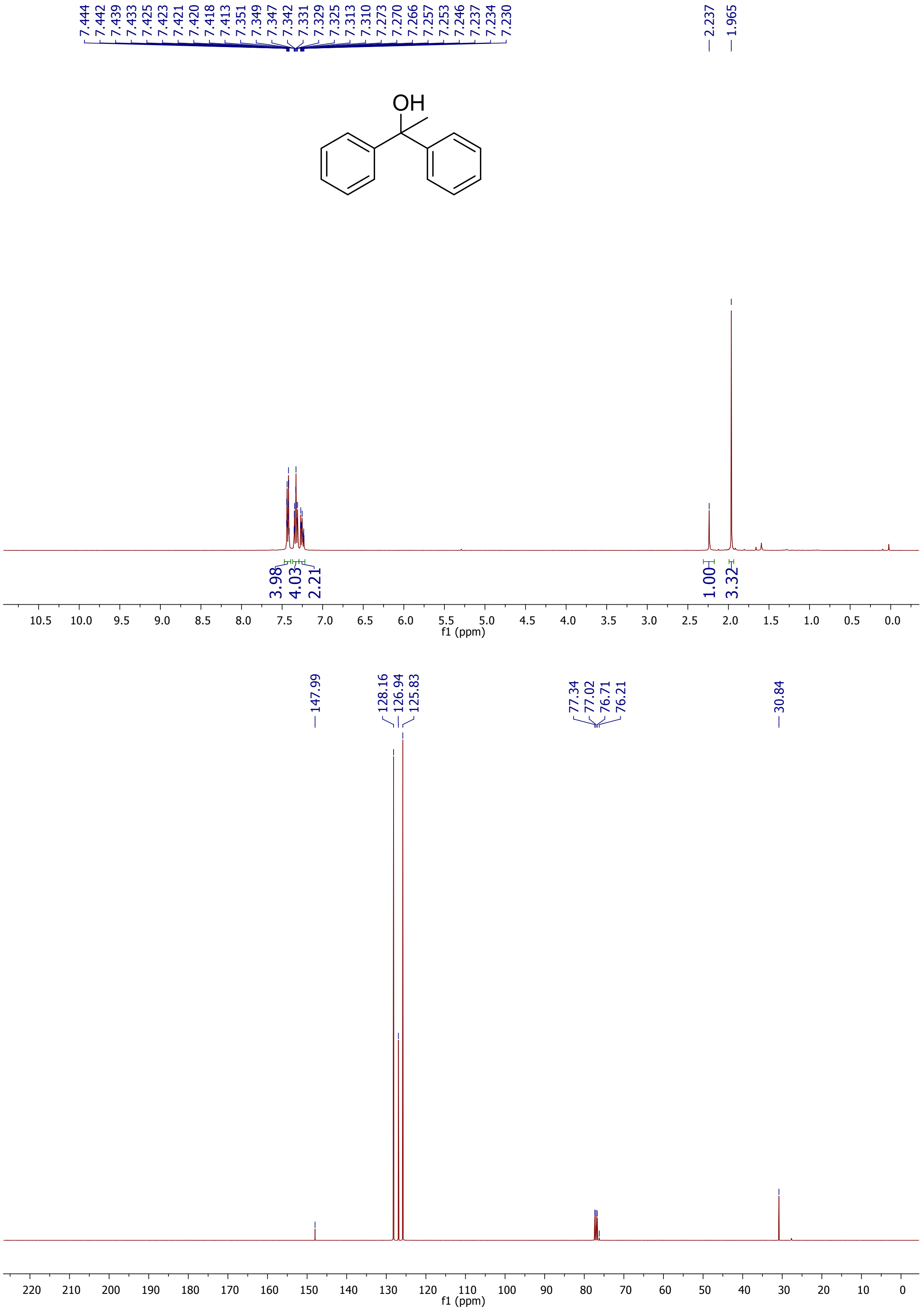

Figure S65. ^{1}H (400 MHz, CDCl$_3$) and ^{13}C{^{1}H} (100.6 MHz, CDCl$_3$) NMR spectra of **6g**

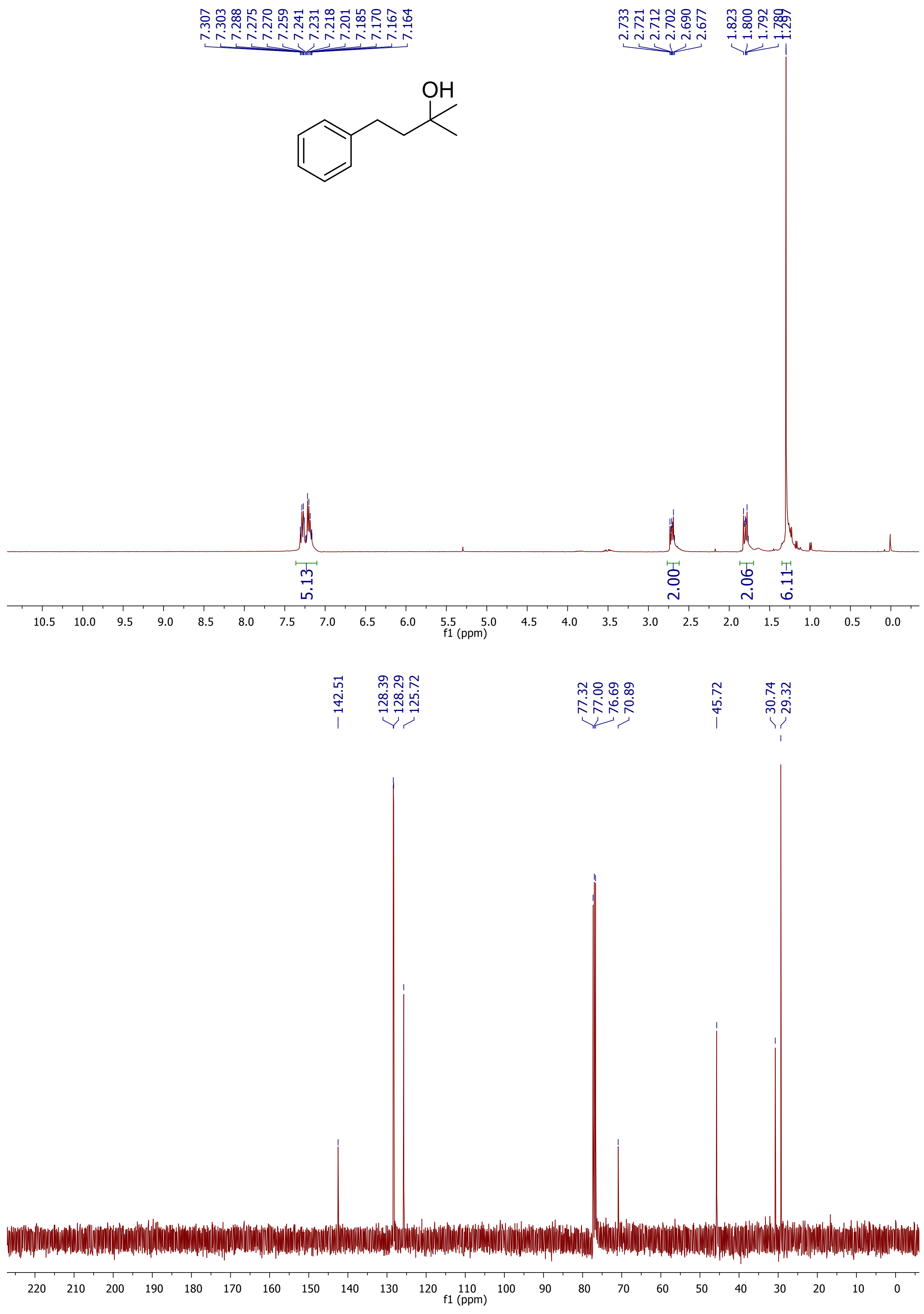

Figure S66. ^{1}H (400 MHz, $CDCl_3$) and $^{13}C\{^{1}H\}$ (100.6 MHz, $CDCl_3$) NMR spectra of **6h**

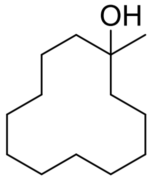

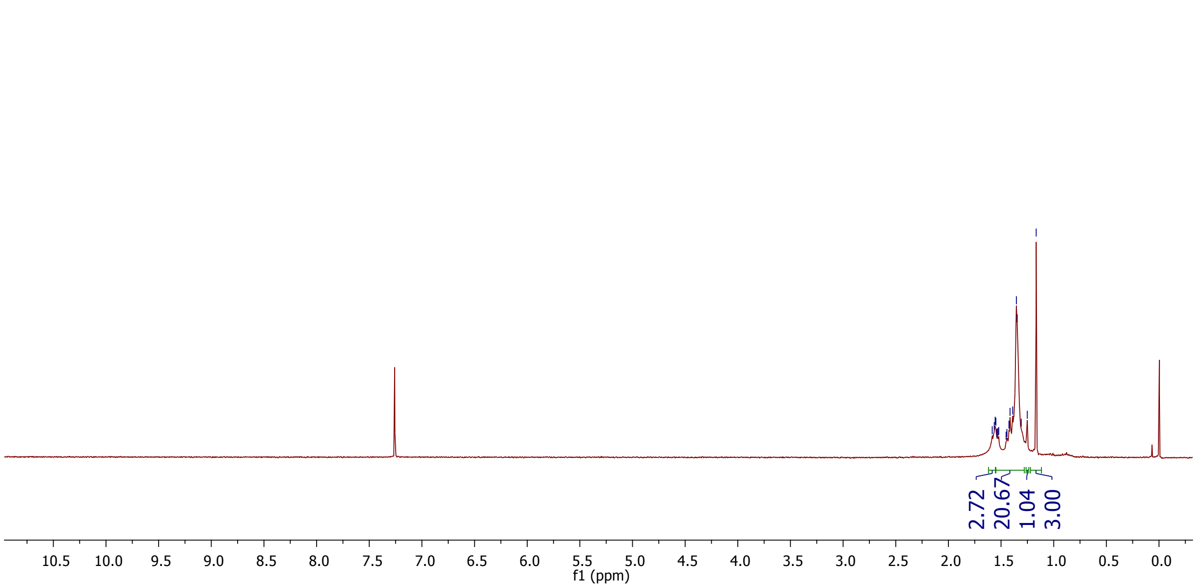

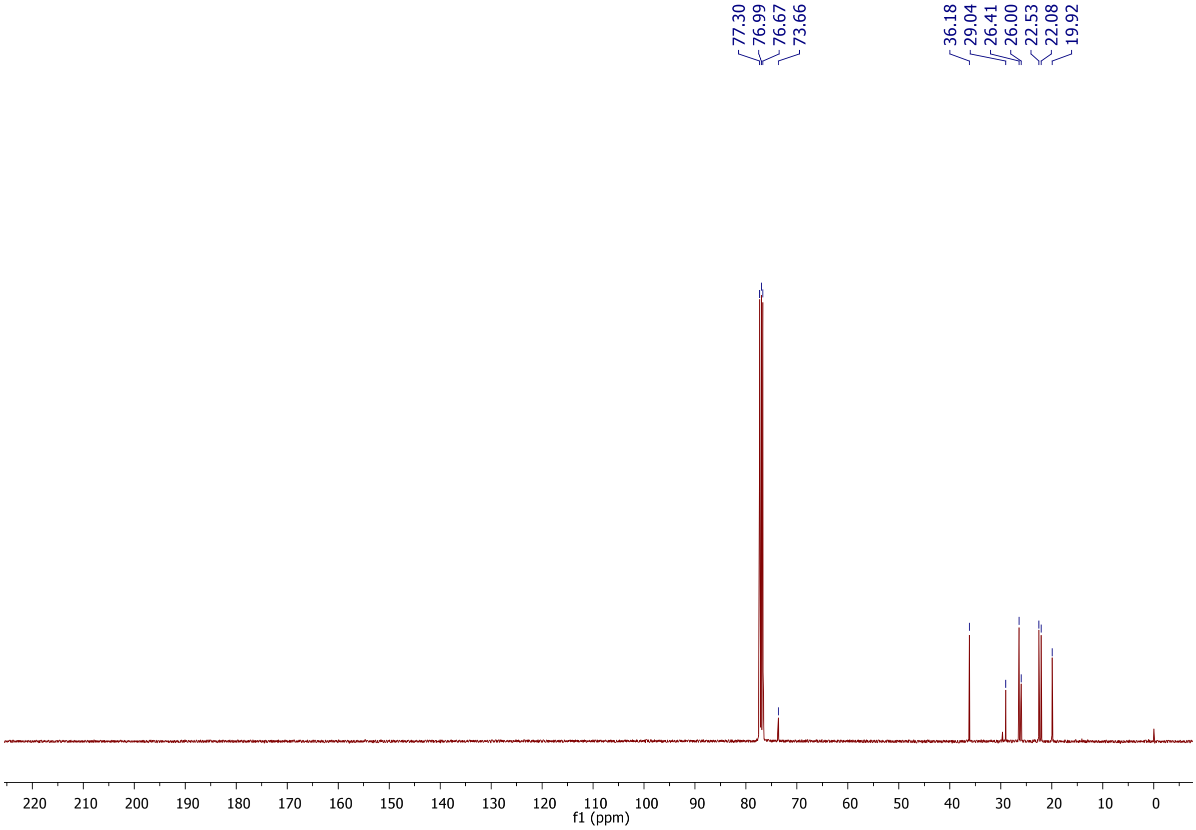

Figure S67. ^{1}H (400 MHz, $CDCl_3$) and $^{13}C\{^{1}H\}$ (100.6 MHz, $CDCl_3$) NMR spectra of **6i**

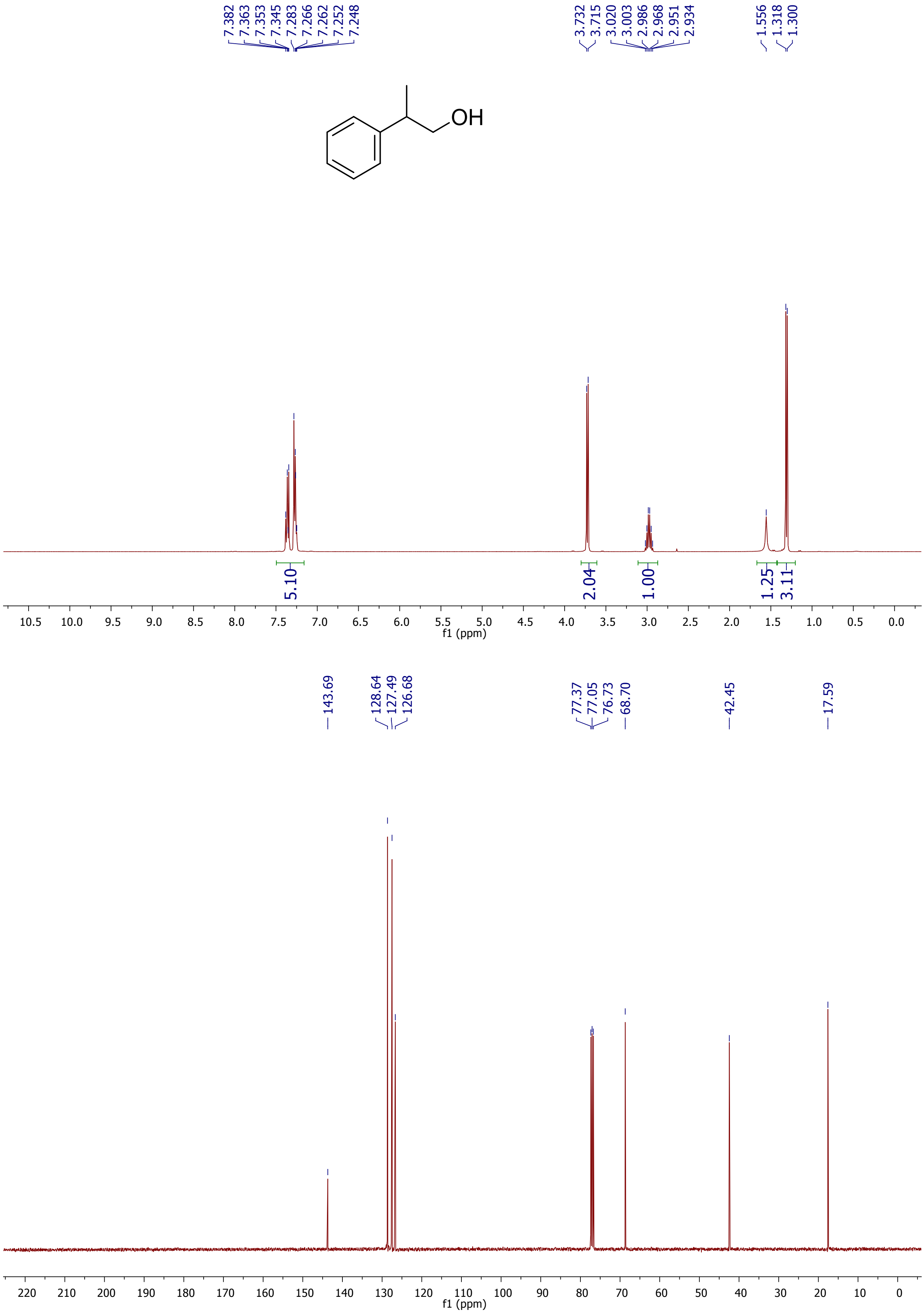

Figure S68. ^{1}H (400 MHz, $CDCl_3$) and $^{13}C\{^{1}H\}$ (100.6 MHz, $CDCl_3$) NMR spectra of **7a**

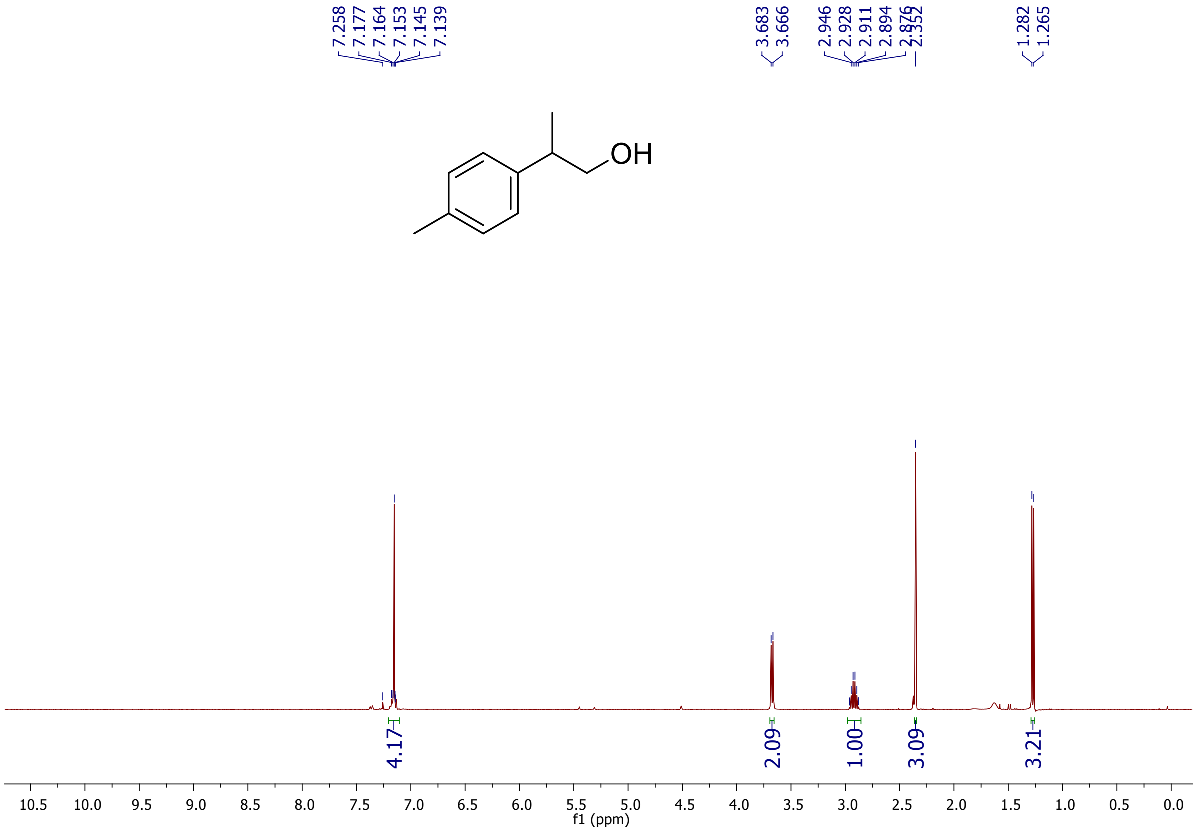

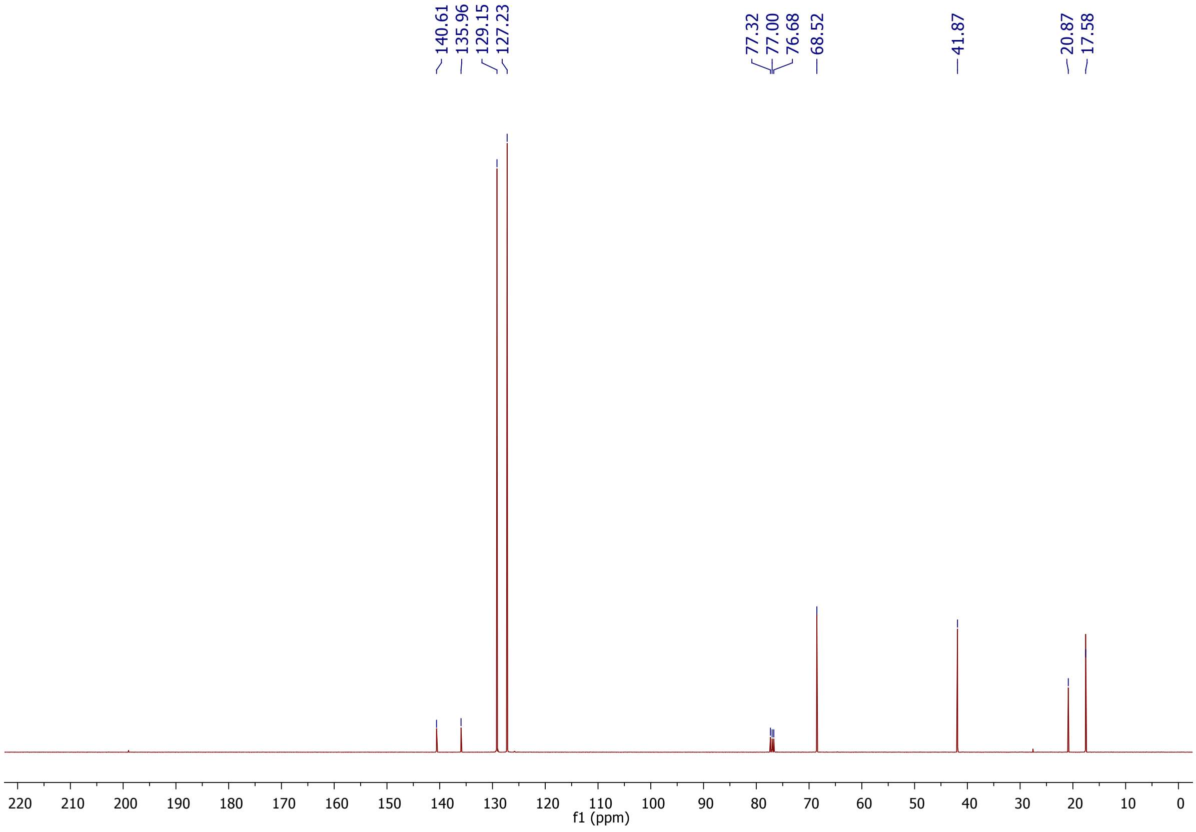

Figure S69. ^{1}H (400 MHz, $CDCl_3$) and $^{13}C\{^{1}H\}$ (100.6 MHz, $CDCl_3$) NMR spectra of **7b**

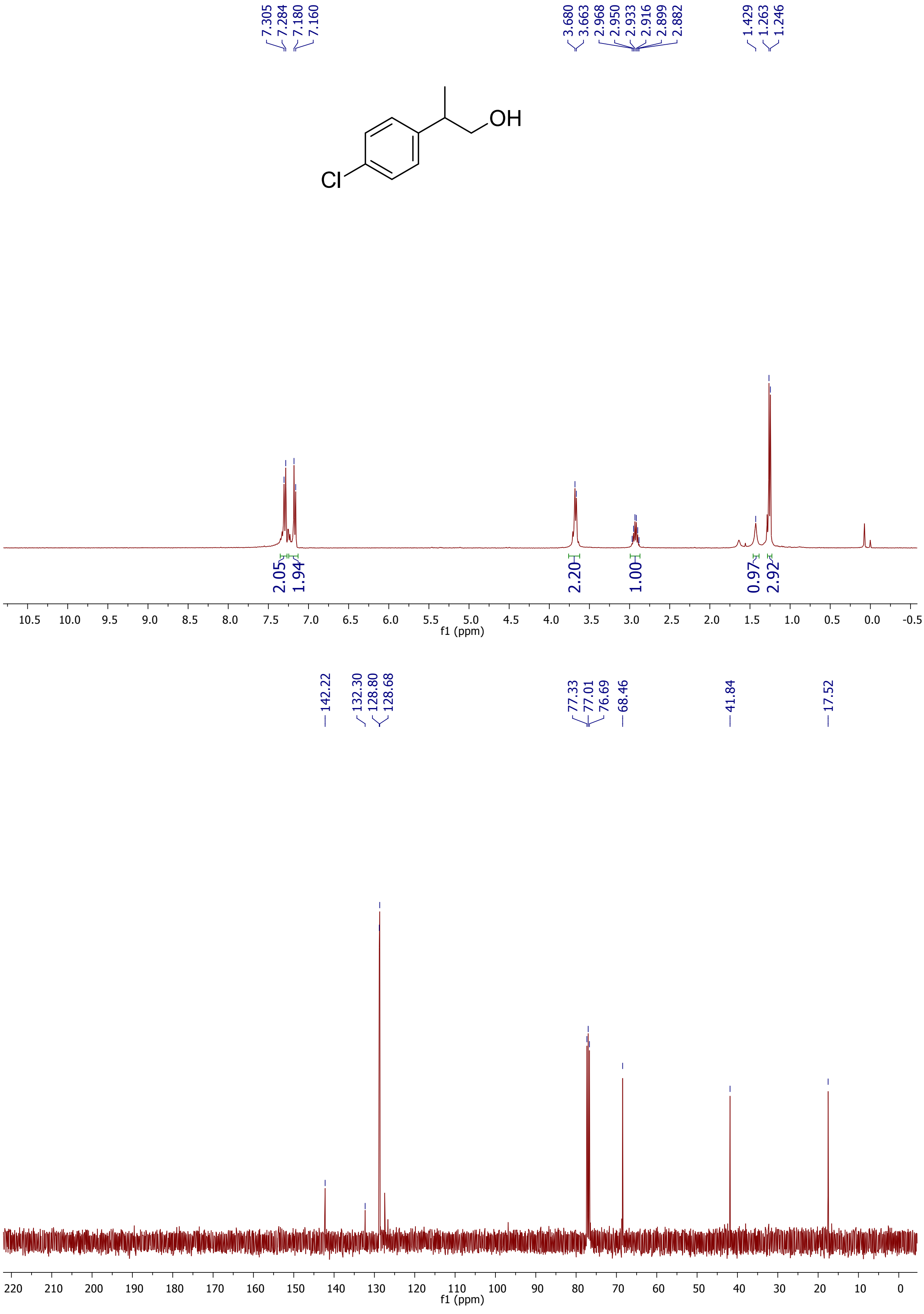

Figure S70. ^{1}H (400 MHz, $CDCl_3$) and $^{13}C\{^{1}H\}$ (100.6 MHz, $CDCl_3$) NMR spectra of **7c**

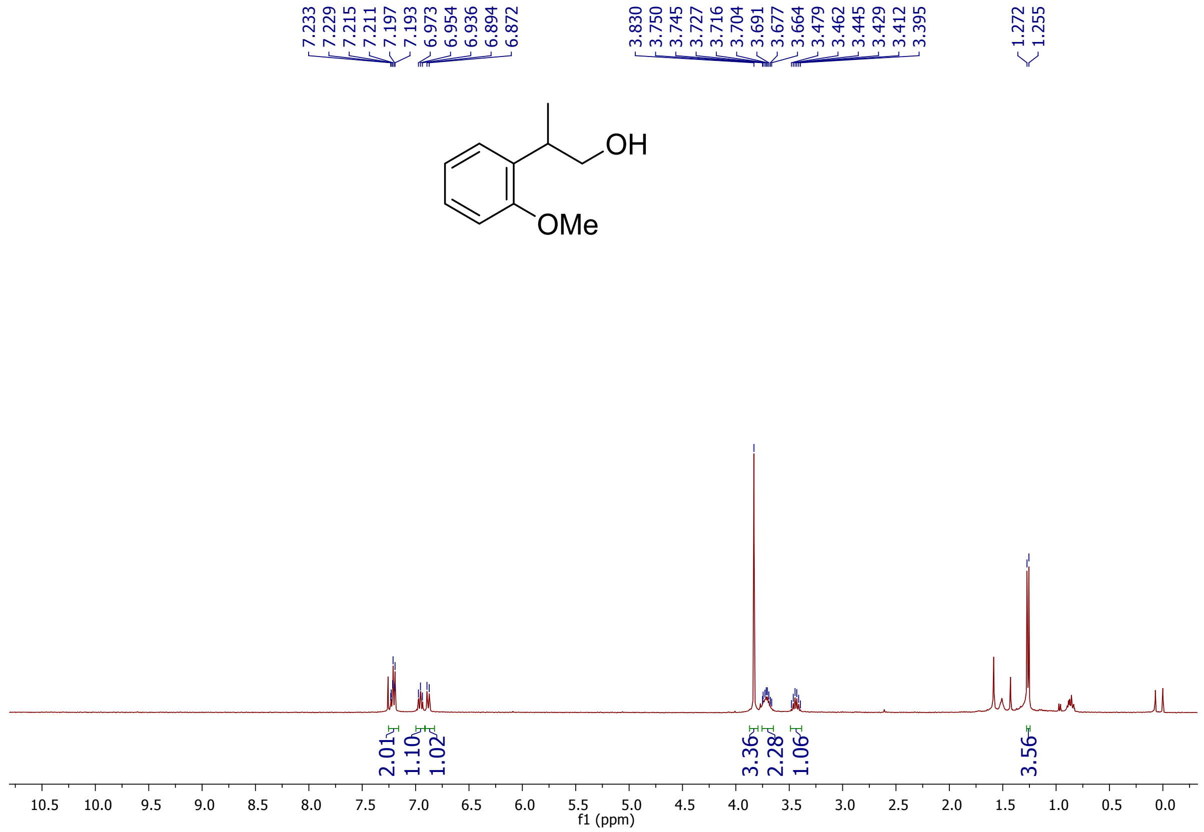

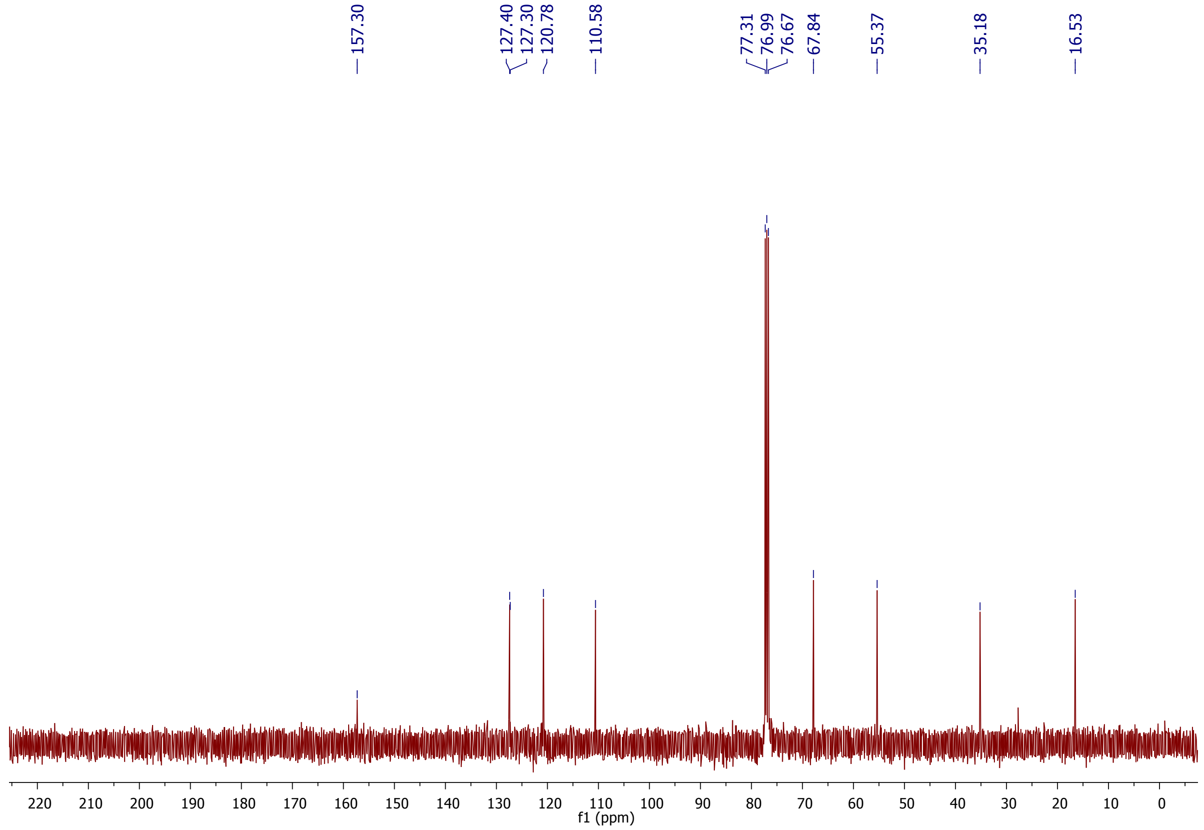

Figure S71. ^{1}H (400 MHz, $CDCl_3$) and $^{13}C\{^{1}H\}$ (100.6 MHz, $CDCl_3$) NMR spectra of **7d**

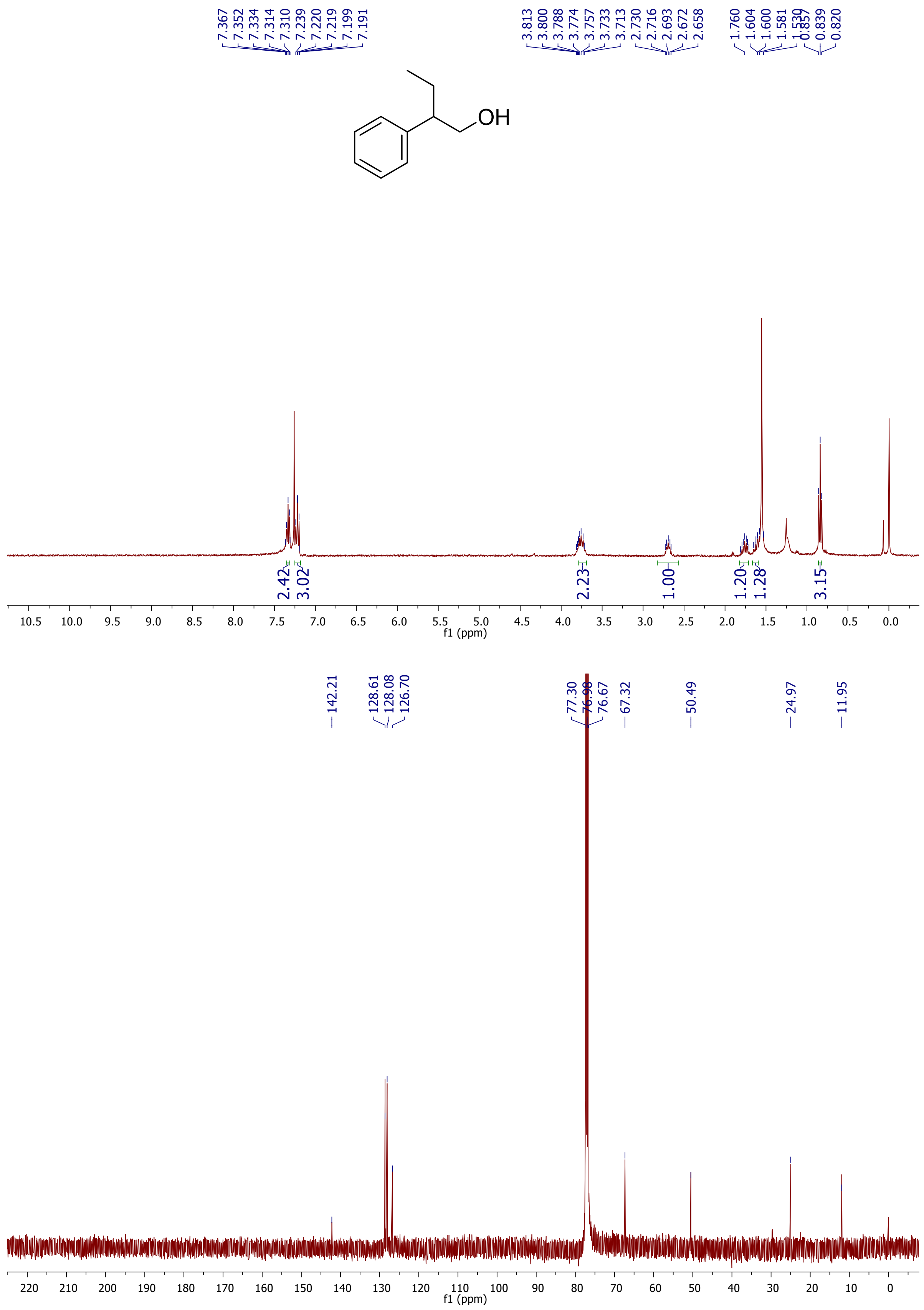

Figure S72. ^{1}H (400 MHz, $CDCl_3$) and $^{13}C\{^{1}H\}$ (100.6 MHz, $CDCl_3$) NMR spectra of **7e**

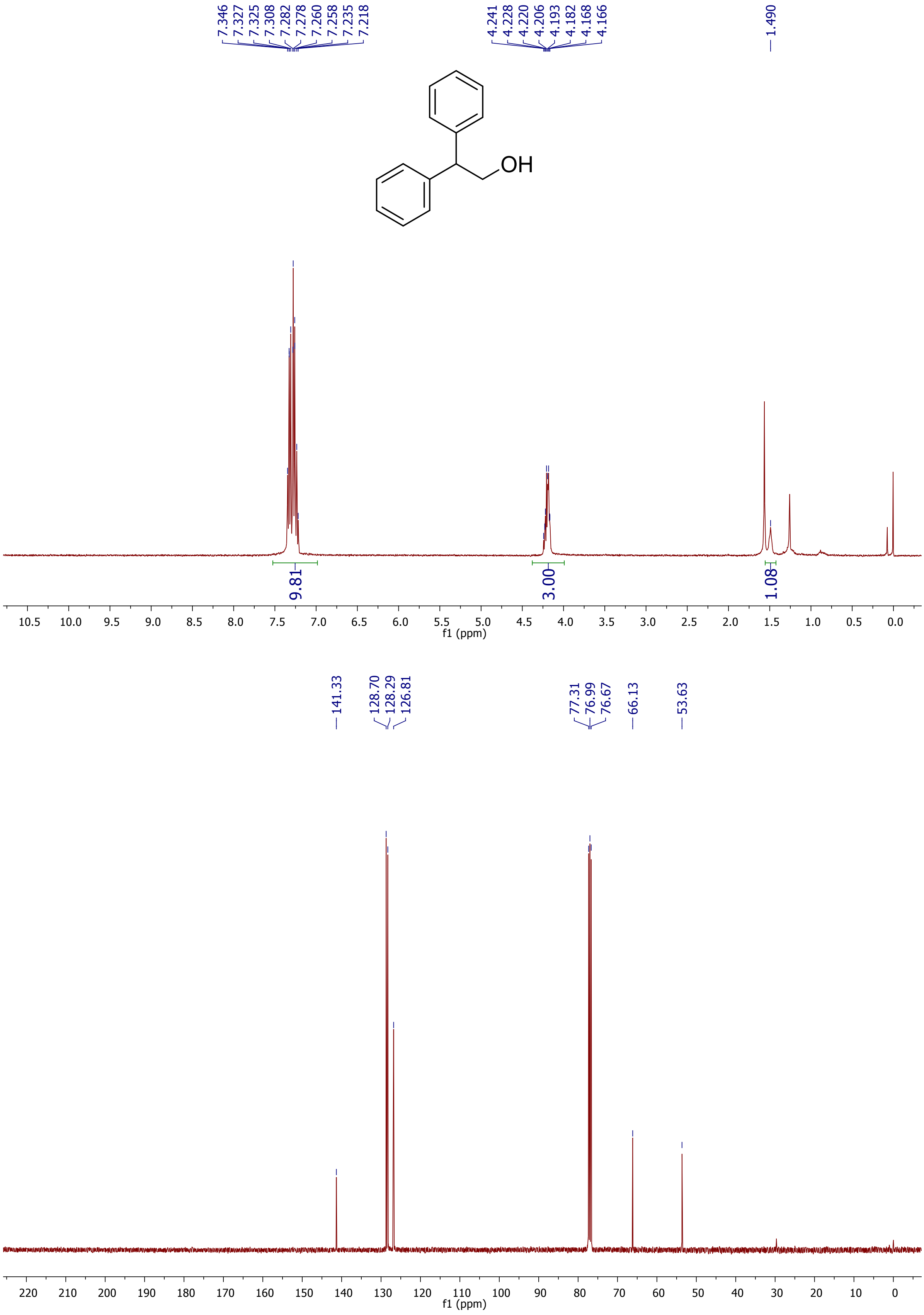

Figure S73. ^{1}H (400 MHz, $CDCl_3$) and $^{13}C\{^{1}H\}$ (100.6 MHz, $CDCl_3$) NMR spectra of **7f**

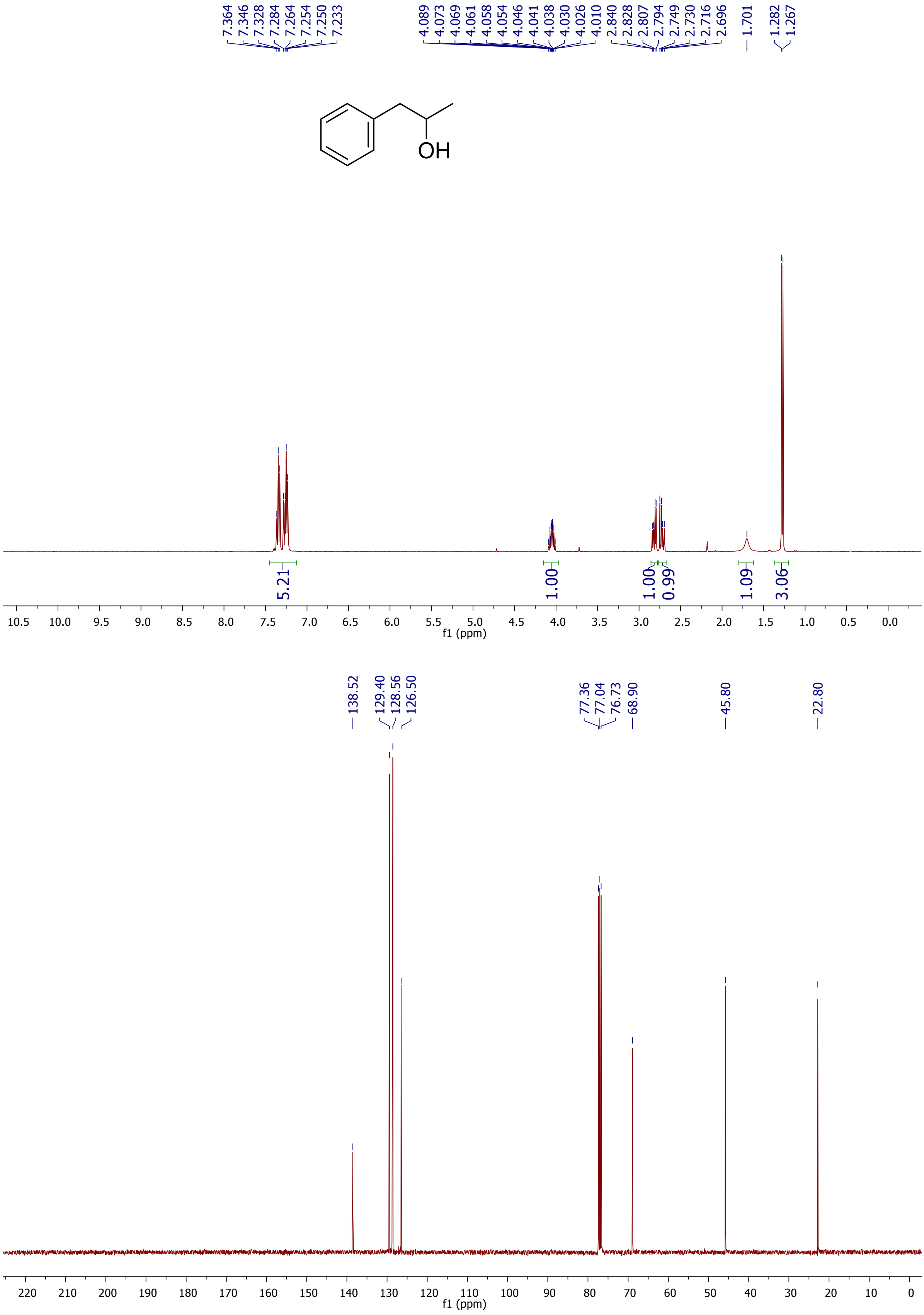

Figure S74. ^{1}H (400 MHz, $CDCl_3$) and $^{13}C\{^{1}H\}$ (100.6 MHz, $CDCl_3$) NMR spectra of **8a**

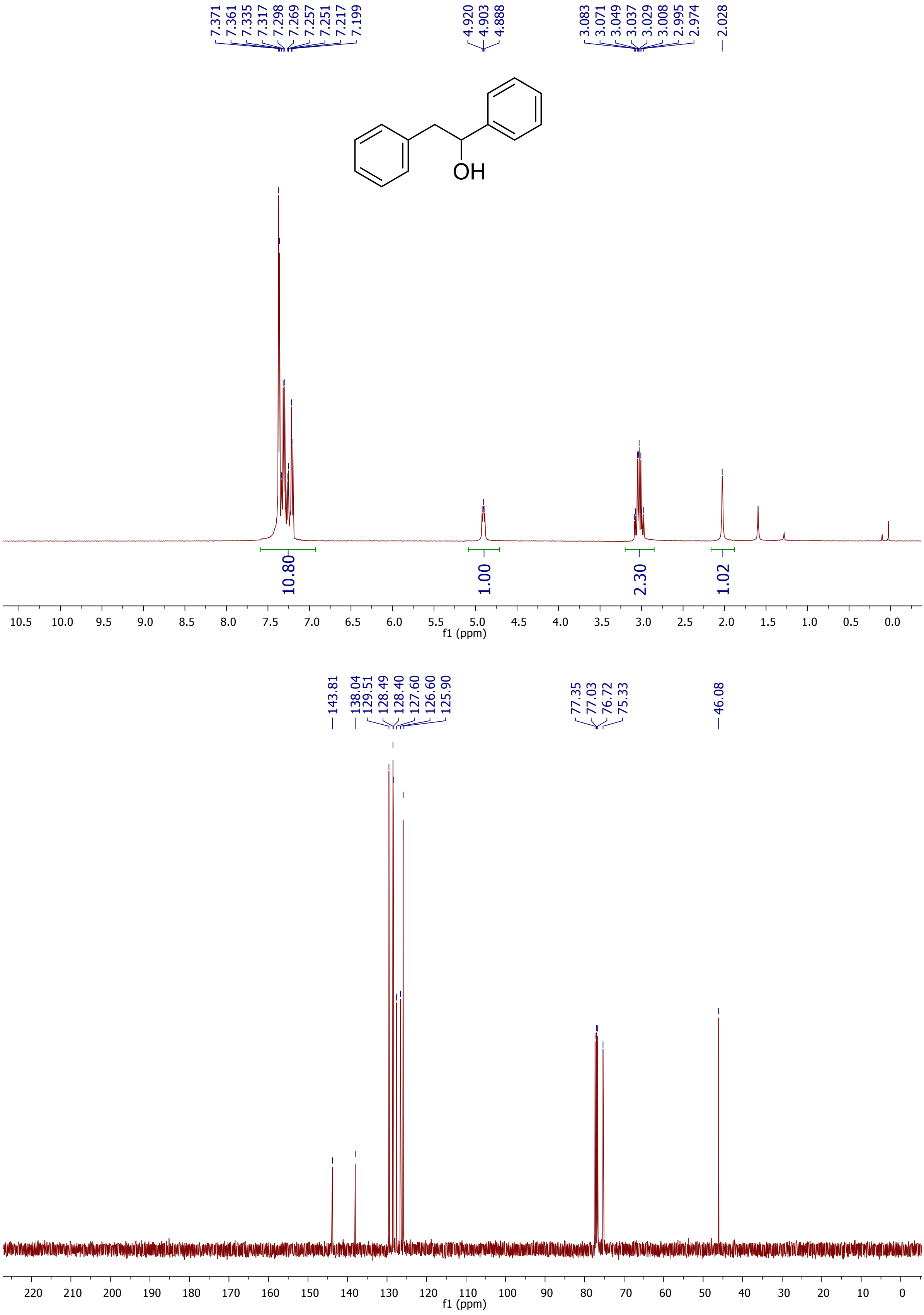

Figure S75. ^{1}H (400 MHz, $CDCl_3$) and $^{13}C\{^{1}H\}$ (100.6 MHz, $CDCl_3$) NMR spectra of **8b**

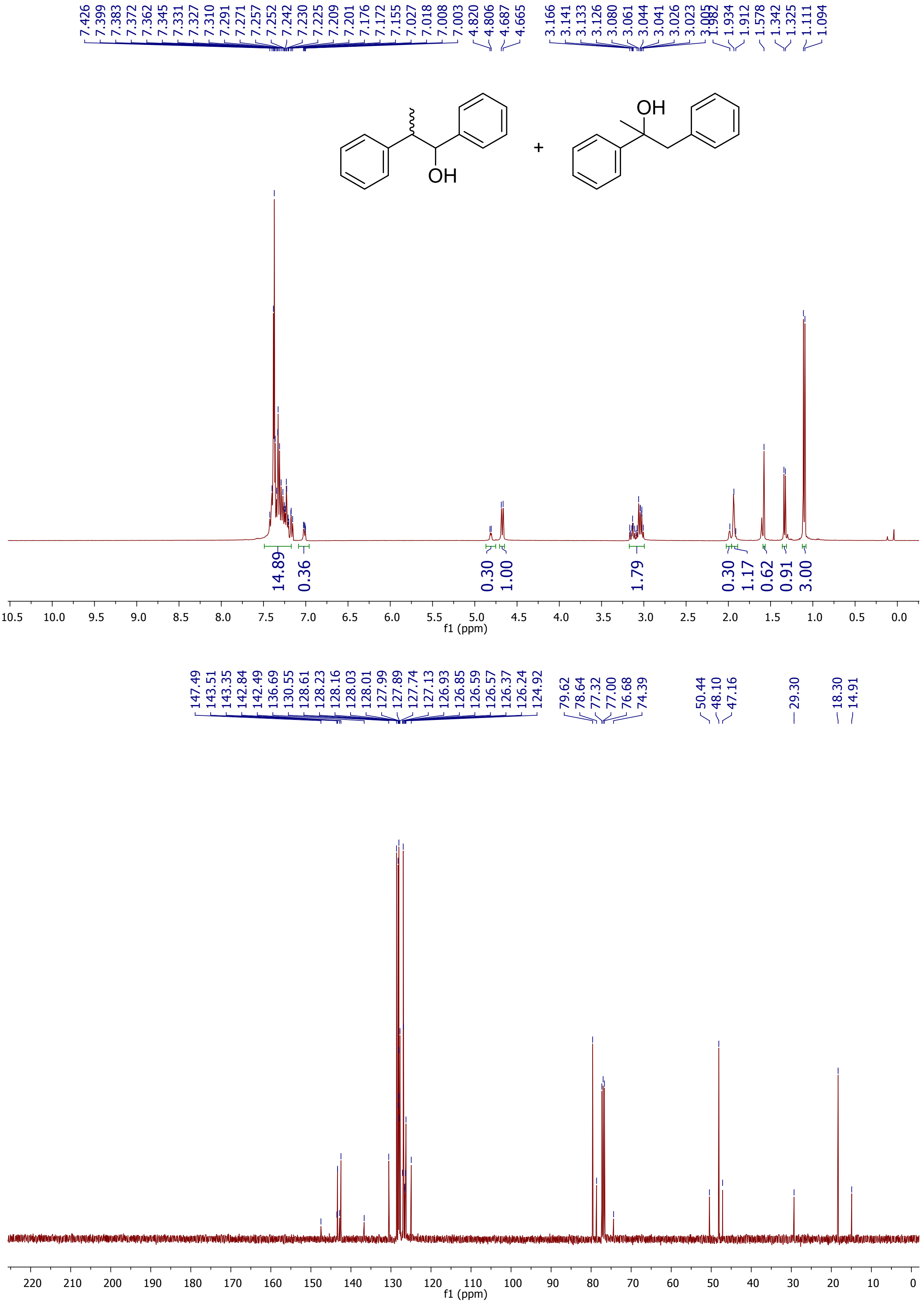

Figure S76. ^{1}H (400 MHz, $CDCl_3$) and $^{13}C\{^{1}H\}$ (100.6 MHz, $CDCl_3$) NMR spectra of **8c**

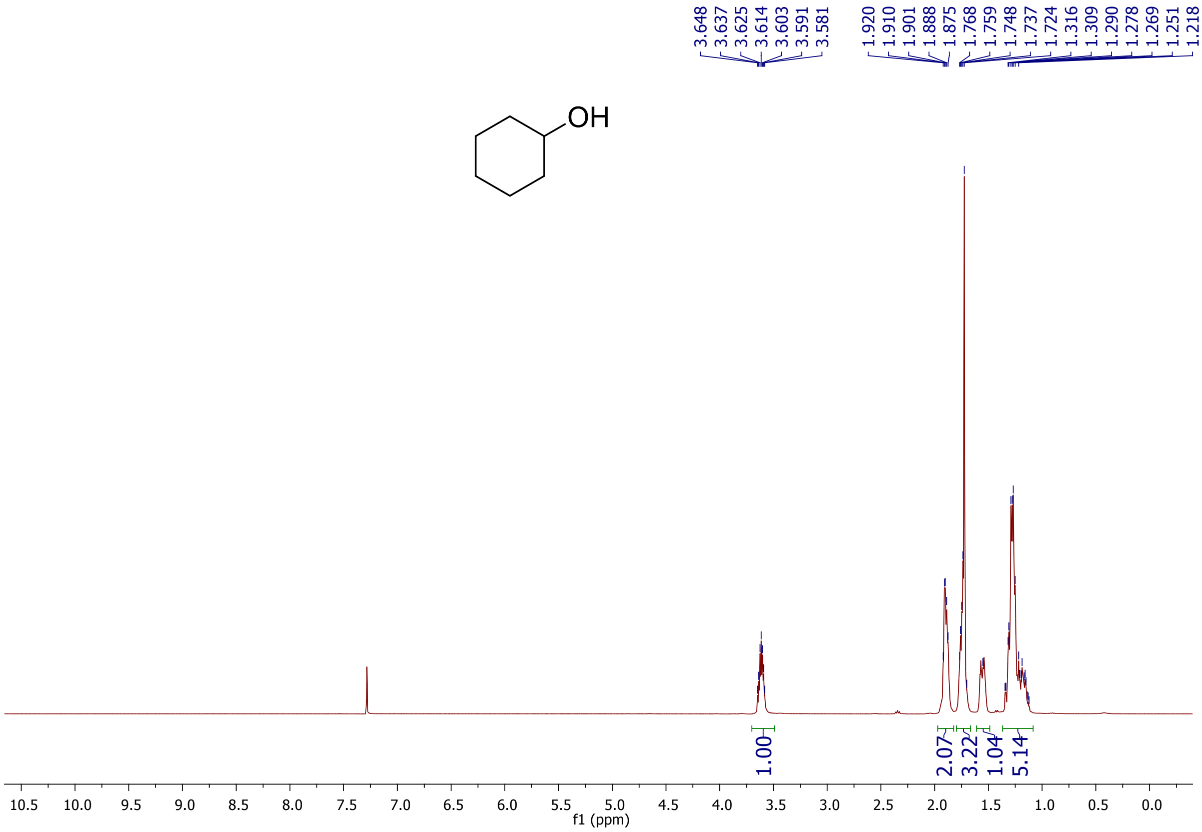

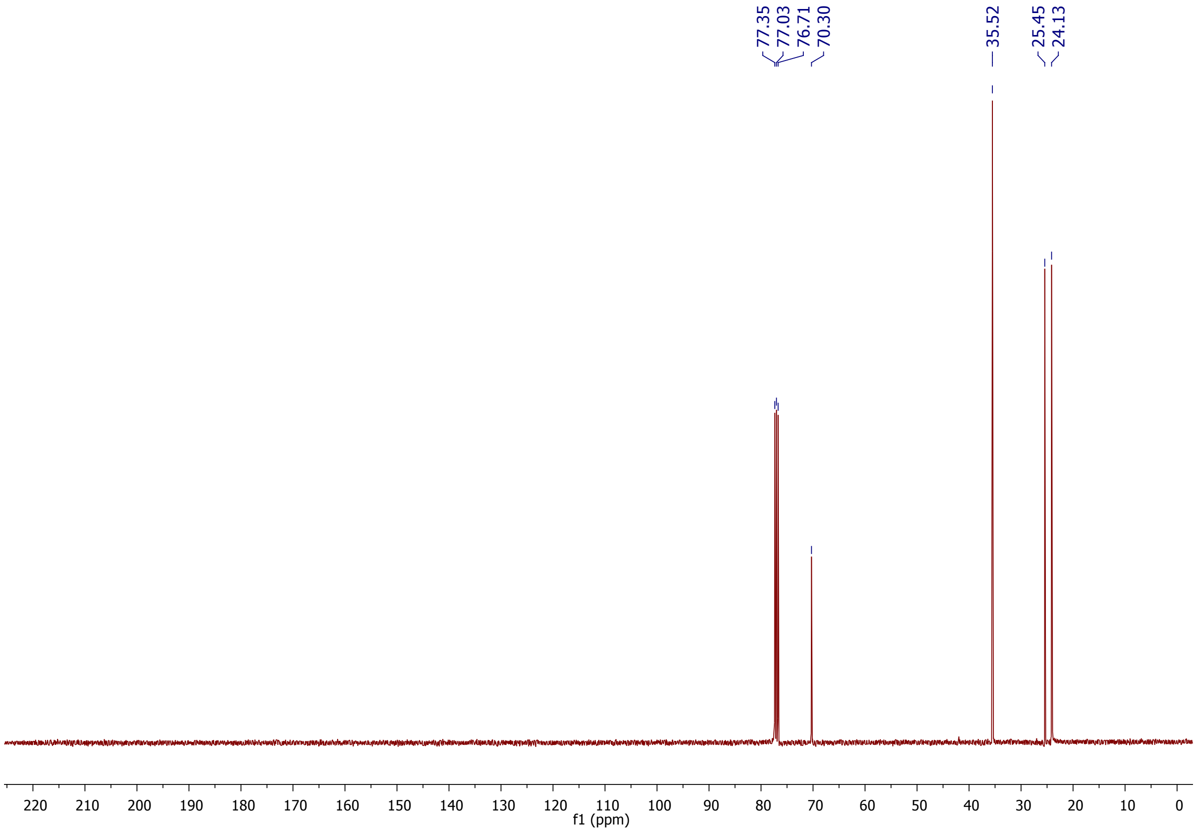

Figure S77. ^{1}H (400 MHz, $CDCl_3$) and $^{13}C\{^{1}H\}$ (100.6 MHz, $CDCl_3$) NMR spectra of **8d**
